# Borate esters: Simple catalysts for the sustainable synthesis of complex amides

**DOI:** 10.1126/sciadv.1701028

**Published:** 2017-09-22

**Authors:** Marco T. Sabatini, Lee T. Boulton, Tom D. Sheppard

**Affiliations:** 1 Christopher Ingold Laboratories, Department of Chemistry, University College London, 20 Gordon Street, London WC1H 0AJ, UK.; 2 Medicines Research Centre, GlaxoSmithKline, Gunnels Wood Road, Stevenage, Herts SG1 2NY, UK.

## Abstract

Chemical reactions for the formation of amide bonds are among the most commonly used transformations in organic chemistry, yet they are often highly inefficient. A novel protocol for amidation using a simple borate ester catalyst is reported. The process presents significant improvements over other catalytic amidation methods in terms of efficiency and safety, with an unprecedented substrate scope including functionalized heterocycles and even unprotected amino acids. The method was used to access a wide range of functionalized amide derivatives, including pharmaceutically relevant targets, important synthetic intermediates, a catalyst, and a natural product.

## Teaser

A commercially available borate ester catalyzes amide formation from carboxylic acids and amines with very high efficiency.

## INTRODUCTION

Amide linkages are at the basis of all life processes, as the key connections in proteins. Although their importance is well recognized in chemistry as a common motif in pharmaceuticals and polymeric materials (*1*), their synthesis is often overlooked as a contemporary challenge. Improved methods for the synthesis of amide functionality are key to the sustainable future of chemical synthesis and manufacturing, especially if they can offer high efficiency and reduced environmental impact. This is a consequence of the fact that amide formation is typically achieved using inefficient and often hazardous reagents, which generate large quantities of waste products leading to high disposal costs (*2*). Accordingly, there have been recent calls from numerous major pharmaceutical companies for research into methods for “amide bond formation avoiding poor atom economy reagents” (*3*).

**Fig. 1:**
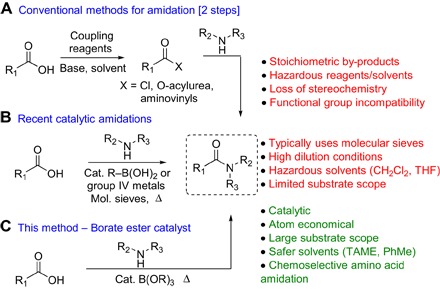
Approaches to amide bond formation. (**A**) Conventional methods for amidation proceeding via an activated carboxylic acid. (**B**) Recent catalytic amidations using group IV metal or boronic acid catalysts. THF, tetrahydrofuran. (**C**) Borate ester–catalyzed amide bond formation. TAME, *tert*-amyl methyl ether; PhMe, toluene.

Despite recent reports of new strategies for amide bond formation from alcohols, aldehydes, or alkynes (*4*–*6*), direct condensation of a carboxylic acid and amine remains the most common approach to amide bond formation, owing to the ubiquity and stability of these functional groups. Conventional methods for direct amidation (Fig. 1A) formally proceed via a two-step sequence involving activated carboxylic acid derivatives, which then undergo aminolysis (*7*, *8*). Indirect amide formations of this sort are expensive and waste-intensive and often suffer from functional group incompatibilities. The ideal approach would involve direct condensation of a carboxylic acid and amine in the presence of a catalyst because the only by-product of the reaction would be water. Direct thermal reaction without a catalyst has relatively limited scope and usually requires high temperatures (*9*, *10*). In recent years, the use of group IV metal salts or boron compounds as catalysts has enabled amidation reactions to take place at lower temperatures (*10*–*24*). However, the use of these catalytic reactions in an industrial context is rare because of their limited reactivity with functionalized substrates and the inefficient procedures used [high dilution conditions and large quantities of waste-intensive molecular sieves (0.8 to 2.5 kg/mol)] (Fig. 1B) (*10*–*24*). Only boric acid has been applied as an amidation catalyst to any great extent on an industrial scale, but it is effective only for relatively reactive acid/amine combinations (*25*).

To address this key issue of sustainability, we sought to develop an operationally simple catalytic method for direct amidation of the carboxylic acid/amine pair at high concentrations in “industrially preferred” solvents and without the need for any additives or dehydrating agents (*26*, *27*). Our interest in the application of borate esters in stoichiometric amidation reactions led us to explore the use of these compounds as amidation catalysts, with a view to their suitability for application of preparing multigram quantities of amide. Here, we disclose a novel efficient protocol for amidation using a borate ester catalyst (Fig. 1C), with an unprecedented substrate scope. This method offers significant improvements in terms of safety and ease of setup and leads to a large reduction in the overall waste generated in an amidation reaction [process mass intensity (PMI)].

## RESULTS AND DISCUSSION

### Development of a highly versatile method for amidation

**Fig. 2:**
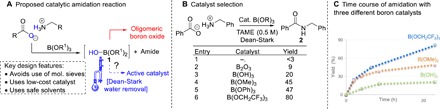
Towards a borate-catalyzed amide coupling. (**A**) Proposed catalytic cycle for amidation. (**B**) Catalyst selection. (**C**) Time course of borate amidation with different boron catalysts.

Borate esters have been demonstrated to mediate amidation reactions of functionalized carboxylic acids and amines (*28*, *29*), yet a priori it was uncertain whether effective catalytic turnover could be achieved because the partially hydrolyzed borate ester **1** could readily undergo decomposition to leave a poorly reactive oligomeric boron oxide (Fig. 2A). Pleasingly, it was observed that effective turnover could be achieved using a Dean-Stark apparatus as an economical and efficient method for water removal, hence avoiding the need for wasteful molecular sieves. Following a solvent screen, *tert*-amyl methyl ether [TAME; boiling point (bp), 86°C] and PhMe (bp, 110°C) were identified as the most effective solvents. The former was preferable in most cases because it led to improved reactivity with functionalized substrates, and the lower bp reduces the energy requirements of the process. There was no background reaction under these conditions in the absence of a catalyst, and all boron-based catalysts examined gave measurably improved yields of amide **2** (Fig. 2B). Evaluation of a series of borate esters confirmed that a commercially available borate ester B(OCH_2_CF_3_)_3_ was the most effective catalyst for this transformation. As can be seen from the time-course plot (Fig. 2C), borate esters B(OMe)_3_ and B(OCH_2_CF_3_)_3_ significantly outperform boric acid, demonstrating that the alkoxy group on the boron atom substantially enhances the catalytic activity.

**Fig. 3:**
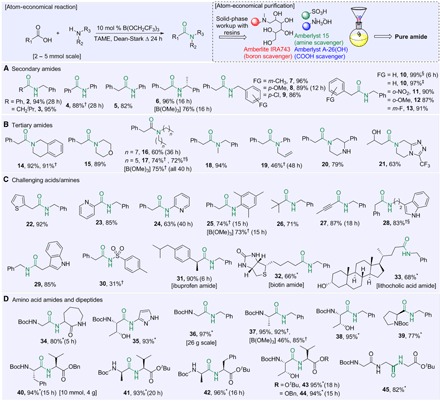
Scope of borate-catalyzed amide bond formation. (**A**) Secondary amides. (**B**) Tertiary amides. (**C**) Challenging amides. (**D**) Amino acid amides. mol %, mole percent; Boc, *tert*-butoxycarbonyl. Reactions run according to general procedure A for 24 hours unless stated otherwise. *Amide synthesized using 20 mole percent (mol %) B(OCH_2_CF_3_)_3_. †Reaction performed in PhMe instead of TAME. ‡Amide synthesized using 1 mol % B(OCH_2_CF_3_)_3_. §Amide synthesized using 5 mol % B(OCH_2_CF_3_)_3_.

The efficiency of the amidation process was further enhanced through the use of an operationally simple method for purification of the amide products using scavenger resins (Fig. 3), which remove unreacted acid and amine, as well as boron-containing impurities. This significantly reduces the solvent requirements of the process by removing the need for aqueous/organic separations and/or chromatographic purification in most cases.

### Scope of the reaction

To evaluate the reaction scope, we explored the preparation of a selection of amides (Fig. 3). A variety of primary amines were successfully coupled with simple carboxylic acids to give secondary amides **2** to **13** in excellent yields. In contrast to existing catalytic amidation reactions, a range of tertiary amides could also be prepared, including examples derived from both cyclic (**14**, **15**, **20**, and **21**) and acylic (**16** to **19**) secondary amines, the latter being particularly difficult compounds to prepare via catalytic amidation. Furthermore, challenging amides were prepared from functionalized/heterocyclic carboxylic acids or amines (**22** to **33**), demonstrating the unprecedented scope of this catalytic amidation reaction. It was even possible to acylate a poorly nucleophilic sulfonamide to give the corresponding derivative **30**, albeit in moderate yield. Naturally occurring carboxylic acids such as biotin (**32**) and lithocholic acid (**33**) were also amenable to amide bond formation. Nonsteroidal anti-inflammatory ibuprofen also smoothly reacted to form the corresponding amide **31**. Our studies also suggested that B(OMe)_3_ was a reasonably effective and very low-cost catalyst for synthesizing relatively unfunctionalized amides when PhMe is used as solvent (**6**, **17**, **25**, and **37**).

We next directed our efforts to amidation reactions of amino acids due to their importance as low-cost renewable raw materials that can be applied to the synthesis of many biologically active targets. As such, the compatibility of our methodology with this type of building block was an important aspect to examine because many of the existing catalytic amidation methods are unsuccessful with these compounds (*11*–*24*). Coupling of *N*-Boc–protected amino acids with a range of amines, including both simple aliphatic amines (**36** to **39**) and functionalized examples (**34** and **35**), gave the corresponding amides in excellent yields. The synthesis of dipeptides (**40** to **44**) and even a tripeptide (**45**) was also demonstrated successfully. Pleasingly, the free hydroxyl functionality of threonine did not require protection, and the corresponding amino amides (**35** and **38**) and dipeptides (**43** and **44**) were obtained in excellent yields. No detectable racemization was observed for any of the amino acid derivatives.

### Amidation with unprotected amino acids

**Fig. 4:**
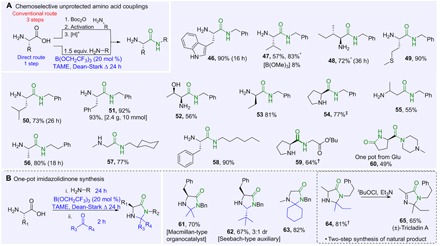
Chemoselective amide bond formation from amino acids. (**A**) Chemoselective amino acid couplings. (**B**) Sequential amidation/condensation to form imidazolidinones. dr, diastereomeric ratio. Reactions run according to general procedures B and C for 24 hours unless stated otherwise. *Using 30 mol % B(OCH_2_CF_3_)_3_. †Using 2 equiv. of amine. ‡Using 1.2 equiv. of benzylamine.

In an effort to address the unmet need for novel chemoselective strategies in chemical synthesis and thereby further prevent the generation of unnecessary waste by-products, we sought to test the application of our catalytic amidation reaction with unprotected amino acids. Protecting group manipulations and amide bond formation account for about a third of the reactions carried out in the synthesis of pharmaceutical intermediates (*30*, *31*), so overcoming these synthetic hurdles could pave the way toward greater sustainability in the chemical industry. Perhaps the most impressive feat of this methodology, catalytic amidation of unprotected amino acids, could be achieved in a chemoselective manner, thereby circumventing the need for protection/deprotection of the amino group (Fig. 4) (*32*). This represents a novel, practical, and more atom-economical route to primary amino acid derivatives, which are a well-documented class of potent anticonvulsants and agents for neuropathic pain treatment (*33*, *34*). Owing to the unreactive/insoluble nature of free amino acids, a higher catalyst loading and a slight excess of amine were necessary for this type of reaction to go to completion. This unique catalytic and chemoselective amide bond formation displayed a broad substrate scope with a variety of functionalized free amino acids and amines. Simple unfunctionalized amino amides (**47**, **48**, **50**, **51**, and **53** to **59**) were obtained in high yields. Diverse functional groups, including hydroxyl groups (**52**), heterocycles (**46**), and sulfides (**49**), were well tolerated. A β-amino acid could also be converted to the corresponding amide, albeit in lower yield (**55**). Pleasingly, glutamic acid underwent a tandem cyclization/amidation to give the corresponding pyroglutamide (**60**) in good yield. This method provides a highly efficient route for the catalytic synthesis of amino amides directly in one step from readily available free amino acids.

### One-pot sequential condensations

Given that B(OCH_2_CF_3_)_3_ has previously been shown to promote imine formation when used stoichiometrically (*35*), we also explored a one-pot unprotected amino acid amidation/condensation reaction to provide access to imidazolidinones in a single step. Using this approach, we were able to prepare a Macmillan-type organocatalyst (**61**) and a Seebach-type auxiliary (**62**), as well as cyclohexanone derivative **63**, in one-pot procedures starting from the unprotected amino acid. Furthermore, we were also able to synthesize the natural product (±)-Tricladin A (**65**) from alanine in a two-step, rather than a five-step, sequence (*36*): The one-pot sequential direct amidation/condensation with 2-butanone provided **64** in 81% yield, which could then be converted into the racemic natural product via a literature oxidation method.

### Synthetic applications of the reaction

**Fig. 5:**
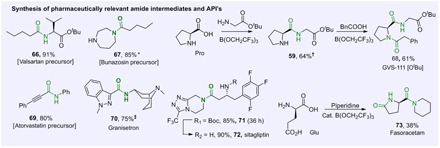
Application to the synthesis of APIs. Reactions run according to general procedures A or B for 24 hours unless stated otherwise. *Using 1.5 equiv. of homopiperazine. †Using 2 equiv. of *H*-Gly-O^*t*^*t*Bu. ‡Using 1.0 equiv. of B(OCH_2_CF_3_)_3_.

We then went on to explore the application of our methodology to the synthesis of active pharmaceutical ingredients (APIs) (Fig. 5). The amide bond formation steps within the syntheses of several top-selling pharmaceuticals, including Valsartan (Diovan) (**66**), Bunazosin (Andante) (**67**), GVS-111 (Noopept) (**68**), Atorvastatin (Lipitor) (**69**), Granisetron (Kytril) (**70**), and Sitagliptin (Januvia) (**71** and **72**), and all, proceeded well using our new amidation procedure. Similarly, Fasoracetam (**73**) was synthesized in one step from glutamic acid, a cheap and readily available chemical feedstock.

**Fig. 6:**
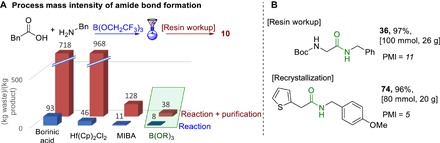
Environmental metrics for catalytic amidation reactions. (**A**) PMI calculations for a selection of catalytic amide bond formation processes. MIBA, 5-methoxy-2-iodophenylboronic acid. (**B**) Improved PMIs of large-scale borate-catalyzed amidation procedures.

Because our goal was to develop a highly efficient and scalable amidation protocol, we sought to demonstrate the efficiency of our method by benchmarking it against other known catalytic amide bond formation processes. The PMI [PMI = (raw material input)/(bulk product output)] provides a widely used measure for the efficiency of a chemical process and was used to compare a selection of recently reported catalytic amidation reactions with the present method (Fig. 6) (*37*). The synthesis of phenylacetamide **10** is ubiquitous in the amidation literature, so this compound was selected for comparison (*12*–*24*, *38*). Pleasingly, our procedure showed clear improvements over existing catalytic amidation methods with regard to the PMI for both (i) the reaction conditions (more concentrated; no additives or molecular sieves) and (ii) the workup (no liquid-phase extraction). The PMI values were also calculated for two further large-scale borate-catalyzed amide syntheses. Although the solid-phase workup procedure is still highly efficient on a multigram scale (**36**), direct crystallization of the amide product from the reaction mixture leads to a further improvement in the PMI (**74**).

### Mechanism of the reaction

Because the alkoxy group present on the borate ester exerts a significant effect on the catalytic activity (Fig. 2B), at least one of these groups must remain attached to the boron atom during the catalytic cycle. Analysis of the contents of the Dean-Stark trap by ^19^F nuclear magnetic resonance (NMR) showed that less than 1 equivalent of trifluoroethanol was removed from the reaction mixture over the course of the amidation reaction, suggesting that the active catalyst has a general structure XB(OCH_2_CF_3_)_2_.

**Fig. 7:**
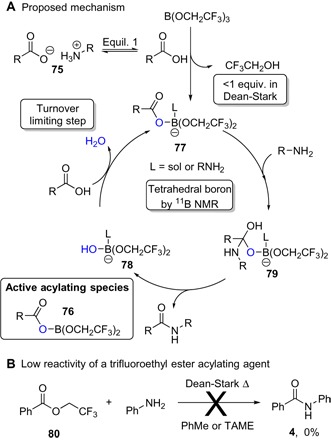
Mechanism of the amidation reaction. (**A**) Proposed mechanism. (**B**) Low reactivity of a trifluoroethyl ester acylating agent. Sol, solvent.

Using a graphical method involving variable time normalization developed by Burés (*39*, *40*), we were able to elucidate the reaction orders from concentration profiles of the reaction. As expected, analysis of the reaction kinetics for the formation of amide **6** suggests a positive dependence on the concentration of borate ester catalyst (0.8th order). This is consistent with a reaction that is first-order in catalyst, but with some competitive decomposition of the active species (*41*). The reaction rate was independent of amine concentration (0th order) but displayed a positive correlation with acid concentration (0.5th order). The noninteger reaction order with respect to the carboxylic acid could be explained by off-cycle equilibria, for example, reversible formation of the amine carboxylate salt **75** (equil. 1) (Fig. 7A) (*41*).

As a consequence of these observations, we propose that the active acylating agent is likely to be an acyloxyboron compound (**76**) bearing two trifluoroethoxy groups, related to the intermediates previously suggested for stoichiometric amidation reactions mediated by alkoxyboron compounds (*42*, *43*). The ^11^B NMR of the reaction mixture only showed that tetrahedral boron species are present, so **76** is likely present as a Lewis base adduct **77** with the amine or solvent (L = amine, trifluoroethanol, or TAME). The former is more likely because the amine is a stronger Lewis base. Note that the first-order dependence on catalyst does not preclude an active species containing two or more boron atoms with bridging carboxylate ligands (for example, [**77**]_2_) (*41*, *44*). On the basis of the kinetic data, we propose a catalytic cycle as shown (Fig. 7A), in which condensation of the carboxylic acid with the monohydroxyboron species **78**, with concomitant water removal, is the turnover limiting step.

Finally, an alternative pathway in which a trifluoroethyl ester, such as **80**, acts as the acylating species (*45*, *46*) was excluded on the basis that ester **80** is not a competent acylating agent for poorly nucleophilic amines such as aniline (Fig. 7B). Further work is under way to fully elucidate the mechanism of this amidation reaction to facilitate the design of more active catalysts.

## Conclusions

Our new borate-catalyzed amide coupling reaction has many advantages over existing methods for amidation: It uses a simple, commercially available catalyst, and the protocol proceeds with high efficiency (low PMI value), with a remarkably broad substrate scope, including application to the synthesis of many pharmaceutically relevant compounds. The procedure can easily be performed on a multigram scale, and the products can be isolated either via a filtration workup or by direct crystallization from the reaction mixture. We anticipate that this method will find many applications in the synthesis of amides in a wide range of fields.

## MATERIALS AND METHODS

### General amidation procedure A

A stirred suspension of an amine (5.0 to 5.5 mmol) and carboxylic acid (5.0 mmol) in TAME (5 ml) was heated to reflux (bp, 86°C) in Dean-Stark apparatus (side arm filled with TAME), and B(OCH_2_CF_3_)_3_ (0.5 mmol; 5 ml of a 0.1 M solution in TAME) was added into the reaction mixture through the Dean-Stark apparatus. An air condenser was fitted, and the reaction mixture was stirred for 2 to 36 hours. Upon completion, the reaction mixture was cooled down to room temperature and concentrated in vacuo. The crude mixture was dissolved in dimethyl carbonate (10 ml) and H_2_O (0.5 ml); Amberlite IRA743 (0.25 g), Amberlyst A15 (0.5 g), and Amberlyst A-26(OH) (0.5 g) resins were added; the resulting suspension was stirred for 30 min. After the disappearance of any remaining starting materials [monitored by thin-layer chromatography (TLC)], MgSO_4_ (~0.5 g) was added. The reaction was filtered, the reaction flask was washed with dimethyl carbonate (2 × 10 ml), and the combined filtrates were concentrated in vacuo to give pure amide.

### General amidation procedure B for unprotected amino acids

A stirred suspension of an amine (7.5 mmol) and unprotected amino acid (5 mmol) in TAME (2.5 ml) was heated to reflux (bp, 86°C) in a Dean-Stark apparatus (side arm filled with TAME), and B(OCH_2_CF_3_)_3_ (1 mmol; 2.5 ml of a 0.4 M solution in TAME) was added through the Dean-Stark apparatus. An air condenser was fitted, and the reaction mixture was stirred for 24 hours. Upon completion, the reaction mixture was concentrated in vacuo and dry-loaded onto silica gel for column chromatography.

### General procedure C for synthesis of imidazolidinones

Following general procedure B, after heating to reflux for 24 hours, a solution of aldehyde or ketone (10 mmol) in TAME (5 ml) was added dropwise over 10 min into the reaction mixture (fig. S2). The reaction was left to stir for 1 hour. If the reaction was not complete, as seen by the disappearance of the intermediate amino amide by TLC (revealed with ninhydrin stain) or high-performance liquid chromatography (HPLC), a further portion of aldehyde or ketone (5 mmol) in TAME (2 ml) was added dropwise over 5 min into the reaction mixture, which was left to stir for another hour. Once complete, the reaction was cooled to room temperature and concentrated in vacuo. The product was purified by flash column chromatography.

## Supplementary Material

http://advances.sciencemag.org/cgi/content/full/3/9/e1701028/DC1

## SUPPLEMENTARY MATERIALS

Supplementary material for this article is available at http://advances.sciencemag.org/cgi/content/full/3/9/e1701028/DC1 General methods Optimization of reaction parameters General procedures Resin capacity in varying solvents PMI calculations Mechanistic studies Spectroscopic data ^1^H and ^13^C NMR spectra Chiral HPLC traces for enantiopurity measurements ^1^H and ^13^C NMR spectra for enantiopurity measurements table S1. Solvent screen for general amidation. table S2. Screening of borate reagents in amino acid amidation. table S3. Varying time, catalyst loading, and equivalents of amine. table S4. Varying concentration. table S5. Reaction troubleshooting. table S6. Solvent screen for resin workup. table S7. Raw data for PMI calculations for amidation product **10**. table S8. PMI calculations for amidation product **10**. table S9. Raw data for PMI calculations. table S10. PMI calculations. table S11. Raw data for determination of order in catalyst. table S12. Raw data for determination of order in catalyst. table S13. Raw data for determination of order in catalyst. table S14. Raw data for determination of order in amine. table S15. Raw data for determination of order in amine. table S16. Raw data for determination of order in amine. table S17. Raw data for determination of order in acid. table S18. Raw data for determination of order in acid. table S19. Raw data for determination of order in acid. table S20. Raw data for determination of order in acid. table S21. Raw data for determination of order in acid. fig. S1. Representative example of a Dean-Stark reaction setup. fig. S2. Representative examples of a Dean-Stark setup with adaptor for the addition of ketone/aldehyde. fig. S3. Representative example of a resin workup. fig. S4. Green metrics for catalytic amidation protocols. fig. S5. ^19^F NMR spectra of the crude reaction mixture (top) and the Dean-Stark (bottom) with fluorobenzene as an internal standard. fig. S6. ^11^B NMR spectra of B(OCH_2_CF_3_)_3_ (top) and reaction mixture (bottom two) at 4- and 24-hour intervals. References (*47*–*66*)
