# Supplementary material for: Borate esters: Simple catalysts for the sustainable synthesis of complex amides
Source: Sci Adv. 2017 Sep 22;3(9):e1701028. doi: 10.1126/sciadv.1701028 (PMC5609808; doi:10.1126/sciadv.1701028)
Supplement: http://advances.sciencemag.org/cgi/content/full/3/9/e1701028/DC1 [file supp_3_9_e1701028__index.html]

Science Advances | Science Advances

## Supplementary Materials

**This PDF file includes:**

- General methods
- Optimization of reaction parameters
- General procedures
- Resin capacity in varying solvents
- PMI calculations
- Mechanistic studies
- Spectroscopic data
- 1H and 13C NMR spectra
- Chiral HPLC traces for enantiopurity measurements
- 1H and 13C NMR spectra for enantiopurity measurements
- table S1. Solvent screen for general amidation.
- table S2. Screening of borate reagents in amino acid amidation.
- table S3. Varying time, catalyst loading, and equivalents of amine.
- table S4. Varying concentration.
- table S5. Reaction troubleshooting.
- table S6. Solvent screen for resin workup.
- table S7. Raw data for PMI calculations for amidation product **10**.
- table S8. PMI calculations for amidation product **10**.
- table S9. Raw data for PMI calculations.
- table S10. PMI calculations.
- table S11. Raw data for determination of order in catalyst.
- table S12. Raw data for determination of order in catalyst.
- table S13. Raw data for determination of order in catalyst.
- table S14. Raw data for determination of order in amine.
- table S15. Raw data for determination of order in amine.
- table S16. Raw data for determination of order in amine.
- table S17. Raw data for determination of order in acid.
- table S18. Raw data for determination of order in acid.
- table S19. Raw data for determination of order in acid.
- table S20. Raw data for determination of order in acid.
- table S21. Raw data for determination of order in acid.
- fig. S1. Representative example of a Dean-Stark reaction setup.
- fig. S2. Representative examples of a Dean-Stark setup with adaptor for the addition of ketone/aldehyde.
- fig. S3. Representative example of a resin workup.
- fig. S4. Green metrics for catalytic amidation protocols.
- fig. S5. 19F NMR spectra of the crude reaction mixture (top) and the Dean-Stark (bottom) with fluorobenzene as an internal standard.
- fig. S6. 11B NMR spectra of B(OCH2CF3)3 (top) and reaction mixture (bottom two) at 4- and 24-hour intervals.
- References (*47–66*)

Download PDF

**Files in this Data Supplement:**

- Adobe PDF - 1701028\_SM.pdf
