# Supplementary material for: Borate esters: Simple catalysts for the sustainable synthesis of complex amides
Source: Sci Adv. 2017 Sep 22;3(9):e1701028. doi: 10.1126/sciadv.1701028 (PMC5609808; doi:10.1126/sciadv.1701028)

Science Advances

AAAS

advances.sciencemag.org/cgi/content/full/3/9/e1701028/DC1

Supplementary Materials for

Borate esters: Simple catalysts for the sustainable synthesis of complex amides

Marco T. Sabatini, Lee T. Boulton, Tom D. Sheppard

Published 22 September 2017, *Sci. Adv.* **3**, e1701028 (2017)
DOI: 10.1126/sciadv.1701028

This PDF file includes:

- General methods
- Optimization of reaction parameters
- General procedures
- Resin capacity in varying solvents
- PMI calculations
- Mechanistic studies
- Spectroscopic data
- ^{1}H and ^{13}C NMR spectra
- Chiral HPLC traces for enantiopurity measurements
- ^{1}H and ^{13}C NMR spectra for enantiopurity measurements
- table S1. Solvent screen for general amidation.
- table S2. Screening of borate reagents in amino acid amidation.
- table S3. Varying time, catalyst loading, and equivalents of amine.
- table S4. Varying concentration.
- table S5. Reaction troubleshooting.
- table S6. Solvent screen for resin workup.
- table S7. Raw data for PMI calculations for amidation product **10**.
- table S8. PMI calculations for amidation product **10.**
- table S9. Raw data for PMI calculations.
- table S10. PMI calculations.
- table S11. Raw data for determination of order in catalyst.
- table S12. Raw data for determination of order in catalyst.
- table S13. Raw data for determination of order in catalyst.
- table S14. Raw data for determination of order in amine.

- table S15. Raw data for determination of order in amine.
- table S16. Raw data for determination of order in amine.
- table S17. Raw data for determination of order in acid.
- table S18. Raw data for determination of order in acid.
- table S19. Raw data for determination of order in acid.
- table S20. Raw data for determination of order in acid.
- table S21. Raw data for determination of order in acid.
- fig. S1. Representative example of a Dean-Stark reaction setup.
- fig. S2. Representative examples of a Dean-Stark setup with adaptor for the addition of ketone/aldehyde.
- fig. S3. Representative example of a resin workup.
- fig. S4. Green metrics for catalytic amidation protocols.
- fig. S5. ^{19}F NMR spectra of the crude reaction mixture (top) and the Dean-Stark (bottom) with fluorobenzene as an internal standard.
- fig. S6. ^{11}B NMR spectra of $B(OCH_2CF_3)_3$ (top) and reaction mixture (bottom two) at 4- and 24-hour intervals.
- References (*47–66*)

General methods

All reagents and solvents were purchased and used as supplied unless otherwise stated. All reactions were carried out at atmospheric pressure with stirring and under air atmosphere unless otherwise indicated. All resins were pre-washed with EtOAc, Et_2O and CH_2Cl_2 and dried *in vacuo* prior to use*. In vacuo* is used to describe evaporation of solvent by Büchi rotary evaporator between 17 ºC and 70 ºC at a pressure of ~ 10 mmHg. All reactions were monitored by TLC or ^{1}H NMR. TLC plates used were pre-coated with silica gel 60 F254 on aluminium (Merck KGaA). The spotted TLCs were visualised by UV light (254 nm or 365 nm) or chemically stained ($KMnO_4$, or Ninhydrin). Column chromatography purification was performed using a Biotage Isolera flash purification system with either Biotage SNAP or GraceResolv flash cartridges prepacked with silica gel (40-60 µm). $[\alpha]_D$ values are given in 10^{-1} deg cm^2 g^{-1}, concentration (*c*) in g per 100 mL. ^{1}H NMR and ^{13}C NMR spectra were recorded at 300, 400, 500 MHz or 600 MHz (for ^{1}H) and 75, 100, 125 MHz or 150 MHz (for ^{13}C) on a Bruker AMX300, AMX400, AMX500 or AMX600 at ambient temperature, unless otherwise indicated. ^{11}B NMR spectra were recorded at 128 MHz on a Bruker AMX400 at ambient temperature unless otherwise indicated. Deuterated solvents for NMR detection used were $CDCl_3$, MeOD or DMSO-d_6 as stated in the spectrum. Peaks are assigned as singlet (s), doublet (d), triplet (t), quartet (q), quintet (qn) or multiplet (m). All shifts are reported in parts per million (ppm) and compared against residual solvent signals: $CDCl_3$ (δ = 7.26 ppm, s), DMSO (δ = 2.50 ppm, qn) or MeOD (δ = 3.31, qn) as the internal standard. Coupling constants (*J*) are quoted in Hertz (Hz) to one decimal place. Mass spectrometry was performed on VG70 SE (ES+, CI, ES- modes). Infra-red spectra were obtained using a Perkin-Elmer Spectrum 100 FTIR Spectrometer operating in ATR mode, all frequencies given in reciprocal centimetres (cm^{-1}). Melting points were measured with a Gallenkamp heating block and are uncorrected.

Optimization of reaction parameters

1a. Optimisation for general amidation

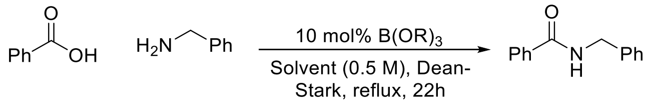

table S1. Solvent screen for general amidation.

Solvent	R	Eq of H_2NBn	Yield (%)
MeCN	CH_2CF_3	2	8
PhF	CH_2CF_3	2	26
Toluene	CH_2CF_3	2	> 95
Toluene	No cat	2	15
Xylene	CH_2CF_3	2	> 95
CPME	CH_2CF_3	2	79
TAME	CH_2CF_3	2	> 95
TAME	CH_2CF_3	1	80
TAME**	CH_2CF_3	1	52

Reaction conditions: Benzoic acid (2 mmol), benzylamine (2 or 4 mmol), 10 mol% $B(OCH_2CF_3)_3$, solvent (4 mL), reflux, 22 h. 1,4-dimethoxybenzene was used as an internal standard. TAME = *tert*-Amyl methyl ether **Reaction carried out without a Dean-Stark apparatus on a 10 mmol scale. Condensed water particles were observed on the condenser, indicating partial water removal from the reaction mixture.

1b. Optimisation for unprotected amino acid amidation

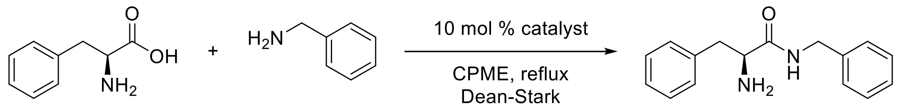

table S2. Screening of borate reagents in amino acid amidation.

Catalyst	Yield (%)
-	0
$B(OMe)_3$	66
$B(OH)_3$	34
B_2O_3	29
$B(OCH_2CF_3)_3$	92
$B(OPh)_3$	78
1:2 B_2O_3: $HOCH_2CF_3$	58

Reaction conditions: Phenylalanine (0.330 g, 2 mmol), benzylamine (0.662 mL, 6 mmol), 10 mol% $B(OCH_2CF_3)_3$, cyclopentyl methyl ether (4 mL), reflux, 15 h. 1,4-dimethoxybenzene was used as an internal standard

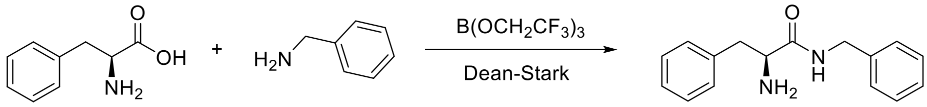

table S3. Varying time, catalyst loading, and equivalents of amine.

Solvent	Equiv of amine	Hours	cat loading (mol%)	yield
TAME	2.0	24	20	93%
TAME	1.5	24	10	90%
TAME	1.5	24	20	90%
TAME	1.5	10	20	63%
TBEE	1.5	24	20	94%

Reaction conditions: Phenylalanine (0.330 g, 2 mmol), benzylamine, $B(OCH_2CF_3)_3$, TAME or TBEE (4 mL), reflux. 1,4-dimethoxybenzene was used as an internal standard. TBEE = *tert*-Butyl ethyl ether

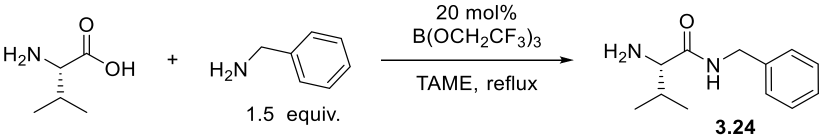

3.24

table S4. Varying concentration.

Solvent	Hours	concentration (M)	Isolated yield	er
TAME	24	0.33	30%	-
TAME	24	0.5	30%	-
TAME	24	0.66	37%	99:1
TAME	24	1.0	57%	99:1
2-MeTHF	24	1.0	16%	-

Reaction conditions: Valine (0.234 g, 2 mmol), benzylamine (0.327 mL, 3 mmol), 20 mol% $B(OCH_2CF_3)_3$, TAME , reflux, 24 h

General procedures

Regular amidation (used for substrate scope in figs. 2,3 & 5 in paper)

2a. Procedure for the synthesis of amides: ***General procedure A***

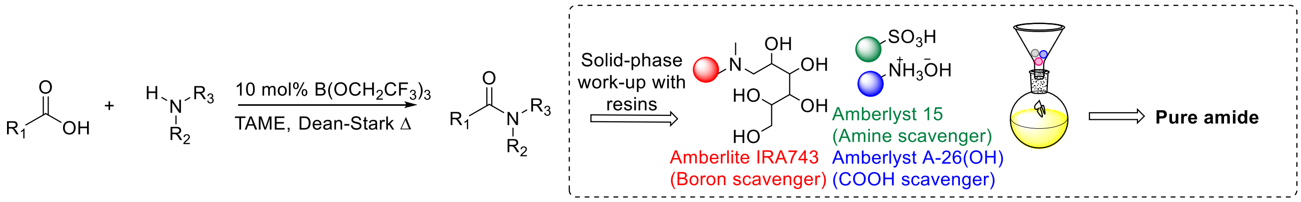

A stirred suspension of an amine (5.0-5.5 mmol) and carboxylic acid (5.0 mmol) in TAME (5 mL) with a Dean-Stark (side-arm filled with TAME) was heated to reflux (bp, 86 °C) and $B(OCH_2CF_3)_3$ (0.5 mmol, 5 mL of a 0.1 M solution in TAME) was added into the reaction mixture through the Dean-Stark. An air condenser was fitted, and the reaction mixture stirred for 2-36 hours. Upon completion, the reaction mixture was cooled down to room temperature and concentrated *in vacuo.* The crude mixture was dissolved in dimethyl carbonate (10 mL) and H_2O (0.5 mL), and Amberlite IRA-743 (0.25 g), Amberlyst A15 (0.5 g) and A-26(OH) (0.5 g) resins were added and the resulting suspension was stirred for 30 min. After disappearance of any remaining starting materials (monitored by TLC), $MgSO_4$ (~0.5 g) was added. The reaction was filtered, the reaction flask washed with dimethyl carbonate (2×10 mL), and the combined filtrates concentrated *in vacuo* to give pure amide.

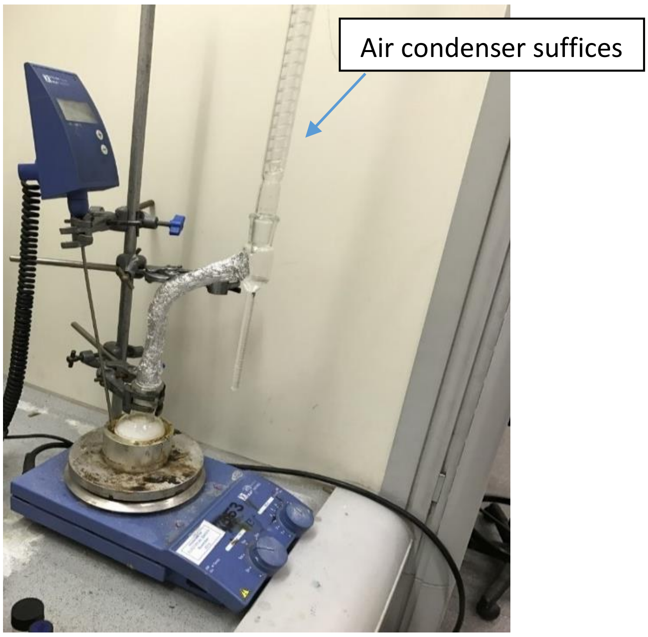

fig. S1. Representative example of a Dean-Stark reaction setup.

Several amino acid esters are not commercially available as free amines (they are instead sold as HCl salts). Where this is the case, the free amine can be obtained in quantitative yields from a liquid phase extraction with saturated Na_2CO_3. Procedure below:

2b. Procedure for the ***Preparation of Amino Acid Ester Free Amines***

The amino acid ester hydrochloride salt (10 mmol) was introduced into a separatory funnel and mixed with EtOAc, DMC or CH_2Cl_2 (20 mL). An aqueous solution saturated with Na_2CO_3 (20 mL) was added and the solution was vigorously shaken and extracted with designated solvent (3 x 20 mL). The combined organic extracts were combined, dried over anhydrous $MgSO_4$, filtered and concentrated *in vacuo* to provide pure amino acid ester amine in quantitative yield.

Unprotected amino acid amidation (used for substrate scope in fig. 4, part 1 in paper)

2c. Procedure for the synthesis of primary amino acid derivatives: ***General procedure B***

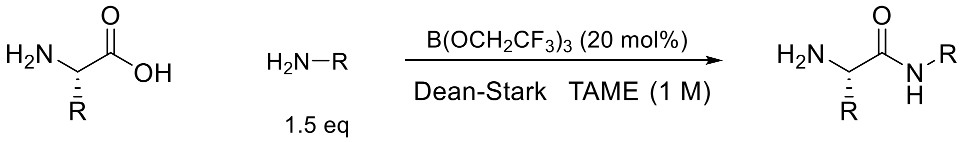

A stirred suspension of an amine (7.5 mmol) and unprotected amino acid (5 mmol) in TAME (2.5 mL) with a Dean-Stark was heated to reflux (bp, 86 °C) and $B(OCH_2CF_3)_3$ (1 mmol, 2.5 mL of a 0.4 M solution in TAME) was added through the Dean-Stark. An air condenser was fitted, and the reaction mixture stirred for 24 hours. Upon completion, the reaction mixture was concentrated *in vacuo* and dry loaded onto silica gel for column chromatography.

Note: Naturally occurring (L)-Amino acids were used, unless specified otherwise. Where amino acids were not available as fine powders, they were ground with mortar and pestle (for amino acids obtained from Alfa Aesar or Calbiochem: Valine, Leucine, Isoleucine, Sarcosine, Methionine, and Glutamic acid).

Unprotected amino acid sequential amidation-condensation (used for substrate scope in fig. 4, part 2 in paper)

2d. Procedure for the synthesis of imidazolidinones: **General procedure C**

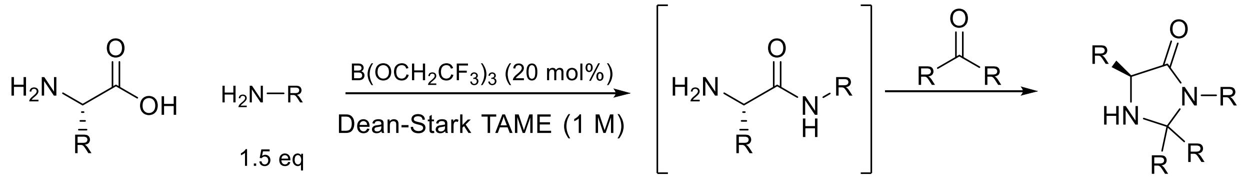

Following general procedure B, after heating to reflux for 24 h, a solution of aldehyde or ketone (10 mmol) in TAME (5 mL) was added dropwise over 10 min into the reaction mixture (SI Fig. 2). The reaction was left to stir for 1 hour. If the reaction was not complete, as seen with the disappearance of the intermediate amino amide by TLC [revealed with ninhydrin stain] or HPLC, a further portion of aldehyde or ketone (5 mmol) in TAME (2 mL) was added dropwise over 5 min into the reaction mixture which was left to stir for a further hour. Once complete, the reaction was cooled to room temperature and concentrated *in vacuo*. The product was purified by flash column chromatography.

Note: to achieve maximum yields it is advisable to use HPLC to monitor the reaction upon addition of the carbonyl, as the intermediate aminoamide can be hard to see on TLC.

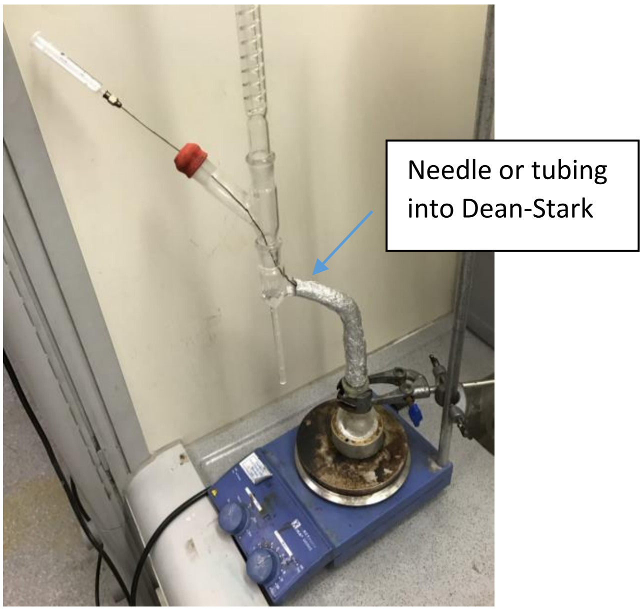

fig. S2. Representative examples of a Dean-Stark setup with adaptor for the addition of ketone/aldehyde.

table S5. Reaction troubleshooting.

Problem	Possible reason	Solution	notes
Product is not clean after resin workup	Resins scavenge amines, carboxylic acids, and boron only. Side products without these functionalities will not be removed	Further purify by crystallisation or column chromatography	Beware of products containing heterocycles or free amines: these will most likely be scavenged by the resins
Product contamination especially at reduced scale	When reactions are scaled down, below 2 mmol, the Dean-Stark apparatus becomes less effective	Dilute reaction conditions (with min. 2 mL solvent)	-
Reaction is not complete	The amine/carboxylate salt is insoluble in TAME or The amine/carboxylic acid pair is unreactive	Increase time of reaction.<br>Try changing solvent [PhMe, Xylene, CPME, in that order]<br>Increase catalyst loading [20 mol% - up to 1 eq]	Heteroatoms may coordinate to boron and alter reactivity or solubility. In this case increase cat. loading first
Reaction shows signs of racemisation	Reagents or Products coordinate with Borate	Shorten reaction time<br>Lower catalyst loading	-

2e. Procedure for enantiomeric ratio determination using Marfey's reagent

Marfeys Reagent for determination of *er*:

The *er* of some amidation reaction products was measured using 1-Fluoro-2,4,-dinitrophenyl-5-L-alaninamide, also known as Marfey's reagent.[47] This reagent was synthesised from 1,5-difluoro-2,4-dinitrobenzene (FFDNB) and L-alaninamide (65% and 68% yield for the D and the L isomers respectively) as reported previously.[28]

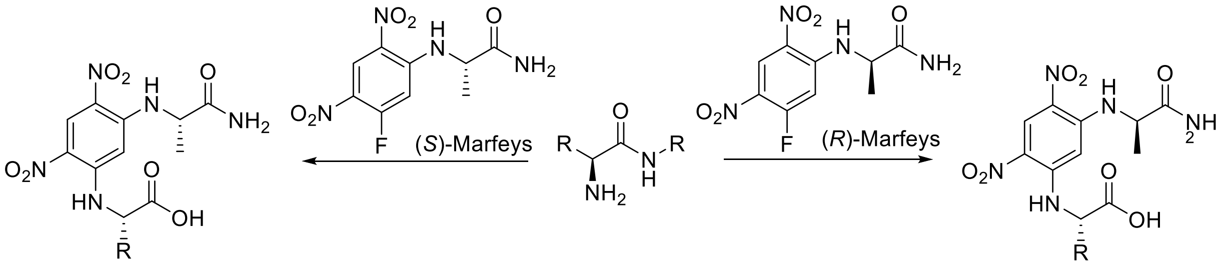

Procedure:

The chiral amide (0.04 mmol, 1 eq) was mixed with L-Marfey's reagent (16 mg, 0.06 mmol, 1.5 eq) and Et_3N (6 µL, 0.04 mmol, 1.0 eq) in 0.6 mL of DMSO-d_6 directly in an NMR tube. The mixture was heated at 40 °C for 1 h and analysed by ^1H and ^{13}C NMR.

The steps above were repeated with D-Marfey's reagent.

NMR spectra obtained from the two crude reactions were then overlaid for determination of enantiopurity.

Resin capacity in varying solvents

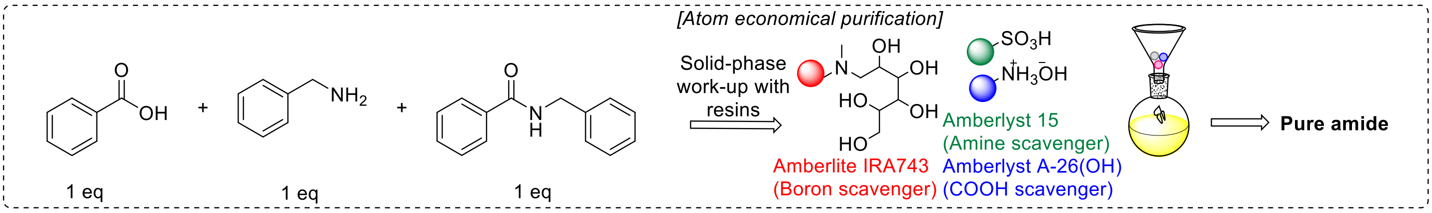

Procedure: In a 25 mL RBF, benzoic acid (0.24 g, 2.0 mmol), benzylamine (0.22 mL, 2.0 mmol), *N*-benzylbenzamide (0.42 g, 2.0 mmol) and 1,4 dimethoxybenzene (0.069 g, 0.50 mmol, internal standard) were dissolved in 4 mL of solvent (dimethyl carbonate (DMC), CH_2Cl_2, 2-MeTHF, EtOAc, or propanol) and H_2O (0.2 mL) was added. Resins IRA743 (0.25 g), A15 (0.5 g) and A26 OH (0.5 g) were added. The reaction mixture was stirred for 30 min, at which point 0.2 g $MgSO_4$ was added. The mixture was filtered, and reaction vial washed twice with 4 mL of the selected solvent.

table S6. Solvent screen for resin workup.

Solvent	Remaining Amine	Remaining Benzoic acid	Remaining Amide
**Dimethyl carbonate**	0%	60%	100%
**CH_2Cl_2**	13%	78%	100%
**Propanol**	15%	60%	100%
**2-MeTHF**	35%	67%	100%
**EtOAc**	5%	85%	100%

The crude mixture was concentrated *in vacuo* and analysed by NMR.

Discussion: The procedure above represents a simulated amidation reaction that has proceeded to 50% conversion. Despite reactions rarely proceeding with such low conversions, and therefore containing high levels of starting materials, such conditions allowed for a more accurate account of the effects of the solvent on resin scavenging: running such test on reactions with more plausible conversions would lead to full scavenging of reagents and thus not allow for differentiation of solvent efficacies. It should also be noted that benzoic acid was observed to be one of the more unreactive substrates with the scavenger resins. Notwithstanding, if reagents are not fully scavenged, one can either: 1) let the mixture stir for a longer period and/or 2) add more resins.

Conclusion: This screen shows that while dimethyl carbonate (recommended solvent on GSK's solvent selection guide) is effective for the resin workup procedure, other solvents such as CH_2Cl_2, EtOAc and propanol can also be used for the resin workup.

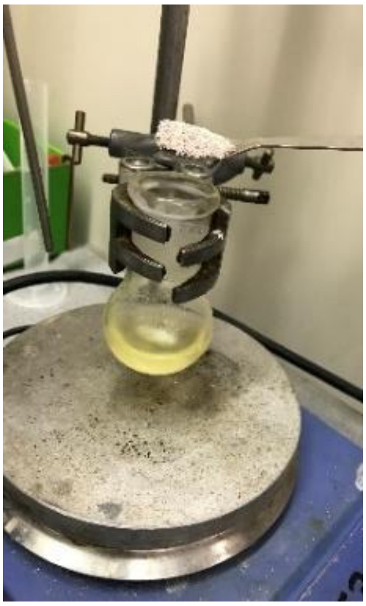

Figure S3a

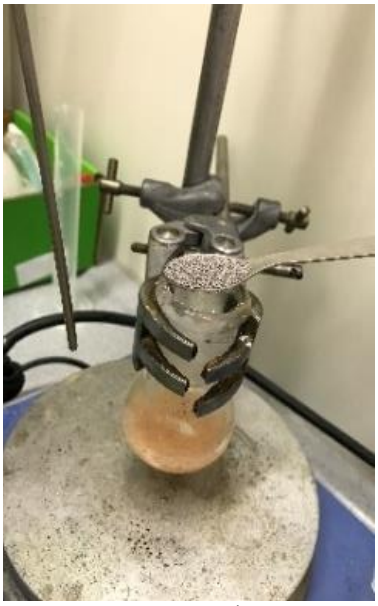

Figure S3b

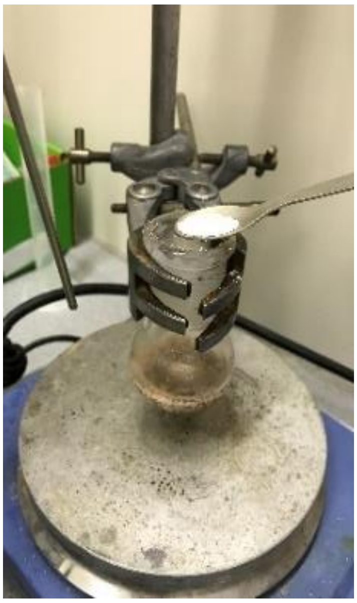

Figure S3c

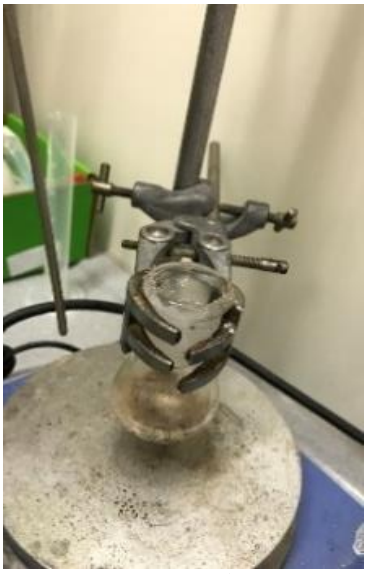

Figure S3d

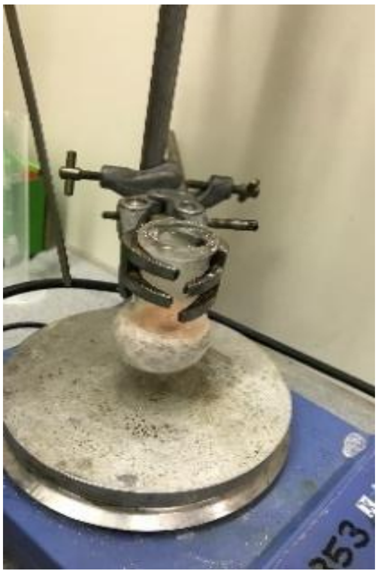

Figure S3e

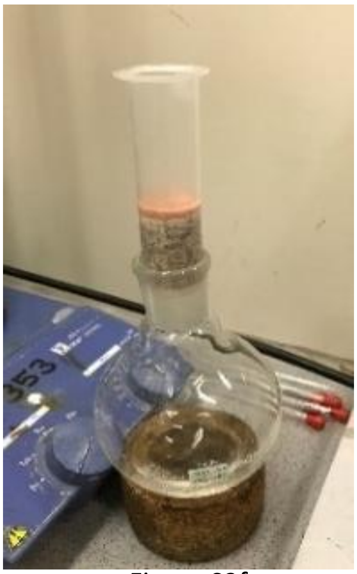

Figure S3f

Representative example of a resin workup. From left to right, top to bottom: Fig. S3a, Addition of A26 OH; fig. S3b, Addition of A15; fig. S3c, Addition of IRA743; fig. S3d, stirring of reaction for 30 min; fig. S3e, Addition of $MgSO_4$, Fig. 3f Filtration, and solvent washes.

PMI calculations

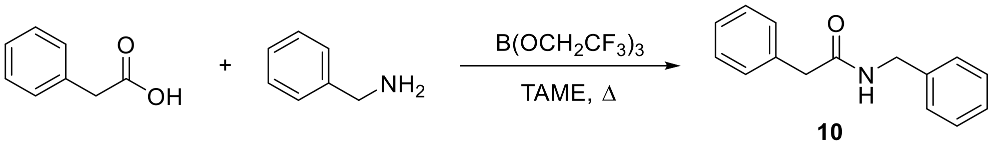

10

table S7. Raw data for PMI calculations for amidation product 10.

Entry	conditions	input reaction	input workup	yield
**1**	Solvent (0.07M), 5 A MS (2g/mmol), Catalyst (10 mol%), Acid (1.1 eq), Amine (1 eq), 48 h, 26 °C	CH_2Cl_2 (9.3 g), 5 A MS (1 g), *Borinic acid* (0.012 g), Phenylacetic acid (0.075g), Benzylamine (0.053 g) *total = 10.4 g*	CH_2Cl_2 (20.0 g), 1M HCl (2×10 g), 1M NaOH (2×10 g), Brine (10 g), *Total = 70 g*	99%, 0.112 g

Procedure for entry **1**: Under argon atmosphere phenylacetic acid (0.55 mmol, 75.0 mg, 1.1 equiv.), the borinic acid (12 mg, 0.05 mmol, 10 mol%) and 1 g of powdered of activated 5Å molecular sieves were introduced. Dry CH_2Cl_2 (7 mL) was added, and the suspension was vigorously stirred for 15 min. Then, benzylamine (0.50 mmol, 54.6 µL, 1 equiv.) was added and the resulting mixture was further stirred for 48 h at room temperature. The suspension was filtered through a pad of celite and washed with CH_2Cl_2 (3 x 5 mL). The filtrate was extracted twice with an aqueous solution of HCl (1M, 10 mL), twice with an aqueous solution of NaOH (1M, 10 mL) and brine (10 mL). The organic layer was collected, dried over anhydrous MgSO4, filtered and evaporated under reduced pressure to yield the title amide as pure product.

Entry	conditions	input reaction	input workup	yield
**2**	Solvent (0.1 M), 4 Å MS (1.5g/mmol), catalyst (5 mol%), Acid (1.0 eq), Amine (2.0 eq), 26 °C, 48h	Et_2O (3.6 g), 4 Å MS (0.75 g), *$Hf(Cp)_2Cl_2$* (9 mg), Phenyl acetic acid (0.068 g), benzylamine (0.106 g), *total = 4.5 g*	EtOAc:Et_3N , 200:1 (90 g) *total = 90 g*	87% yield, 0.0976 g

Procedure for entry **2**: Carboxylic acid (38 mg, 0.5 mmol), molecular sieves 4Å (0.75 g) and $Hf(Cp)_2Cl_2$ (5-10 mol%) were added to a 20 mL MW-vial equipped with a magnetic stirring bar and a cap with septum was crimped on. The atmosphere was evacuated and replaced with nitrogen gas. The vial was placed in an oil bath at 26 °C and dry diethylether (5 mL) was added. The amine (1 mmol) was added with a syringe and the reaction was stirred at constant temperature for the time indicated in the article. Thereafter, the mixture was filtered through a plug of silica (4 x 3.5 cm) with 100 mL of an EtOAc:Et_3N (200:1) eluent. The solvent was removed under reduced pressure affording analytically pure product unless otherwise noted.

Entry	conditions	input reaction	input workup	yield
**3**	Solvent (1M), 4 Å MS (1g/mmol), catalyst (10 mol%), Acid (1.1 eq), Amine (1.0 eq), 50 °C, 18h	PhMe (29 g), 4 Å MS (33 g), *MIBA* (0.92 g), Phenyl acetic acid (4.90 g), Benzylamine (3.56 g), *total = 71.4 g*	CH_2Cl_2 (67 g) 1M HCl (200 g) 1M NaOH (200 g) Brine (50 g) CH_2Cl_2 (266 g) *total = 783 g*	89% yield, 6.693 g

Procedure for entry **3**: Into a 250 ml round bottom flask equipped with a stir bar was added phenylacetic acid (4.90 g, 36 mmol, 1.1 equiv.), 5-methoxy-2-iodophenylboronic acid (0.92 g, 3.3 mmol, 10 mol%) and 33 g (1 g per mmol of amine substrate) of activated 4A molecular sieves. Toluene was added to maintain a concentration at 1 M and the mixture was stirred. After 10 minutes, the amine (3.56 g, 33 mmol, 1.0 equiv.) was added. The resulting mixture was stirred for 18 h at 50 °C. The reaction mixture was filtered through a pad of Celite 545, which was rinsed with

CH_2Cl_2 (50 ml). The filtrate was washed with a 1 M aqueous acidic solution (4 × 50 ml), 1 M aqueous basic solution (4 × 50 ml) and brine (50 ml). The combined aqueous solutions were back extracted with CH_2Cl_2 (4 × 50 ml), then the combined organic layers were dried over anhydrous Na_2SO_4, filtered and evaporated to dryness to yield the title amide product

**4**	Solvent (0.5 M), $B(OCH_2CF_3)_3$ (5 mol%), Acid (1.0 eq), Amine (1.0 eq), 86 °C, 8 h	TAME (7.7 g), $B(OCH_2CF_3)_3$ (0.077 g), Phenylacetic acid (0.680 g), benzylamine (0.535 g), *total = 8.99 g*	Resins (1.2 g) DMC (10.7 g) H_2O (0.5 g) DMC (21.4 g) *total*= 33.8	99% yield, 1.12 g

Entry 1: Chem. Commun., 2015,51, 16084-16087, DOI: 10.1039/C5CC06177F***;***; ***Entry 2:*** ACS Catal., 2015, 5 (6), 3271–3277, DOI: 10.1021/acscatal.5b00385; ***Entry 3***: Green Chem., 2015,17, 4016-4028, DOI: 10.1039/C5GC00659G ***Entry 4***: This work

$$\textbf{Process Mass Intensity} = \frac{\boldsymbol{total\ mass\ in\ a\ process\ or\ process\ step\ (kg)}}{\boldsymbol{mass\ of\ product\ (kg)}}$$

For a discussion on why PMI is used in the Pharmaceutical Industry see article by Jimenez-Gonzalez *et. al.*[48]

table S8. PMI calculations for amidation product 10.

Entry	(Reaction input)/(product output) [kg]	(Reaction + workup input)/(product output) [kg]
**1**	93	718
**2**	46	968
**3**	11	128
**4**	8	38

Entry 1: Chem. Commun., 2015,51, 16084-16087, DOI: 10.1039/C5CC06177F***;***; ***Entry 2:*** ACS Catal., 2015, 5 (6), 3271–3277, DOI: 10.1021/acscatal.5b00385; ***Entry 3***: Green Chem., 2015,17, 4016-4028, DOI: 10.1039/C5GC00659G ***Entry 4***: This work

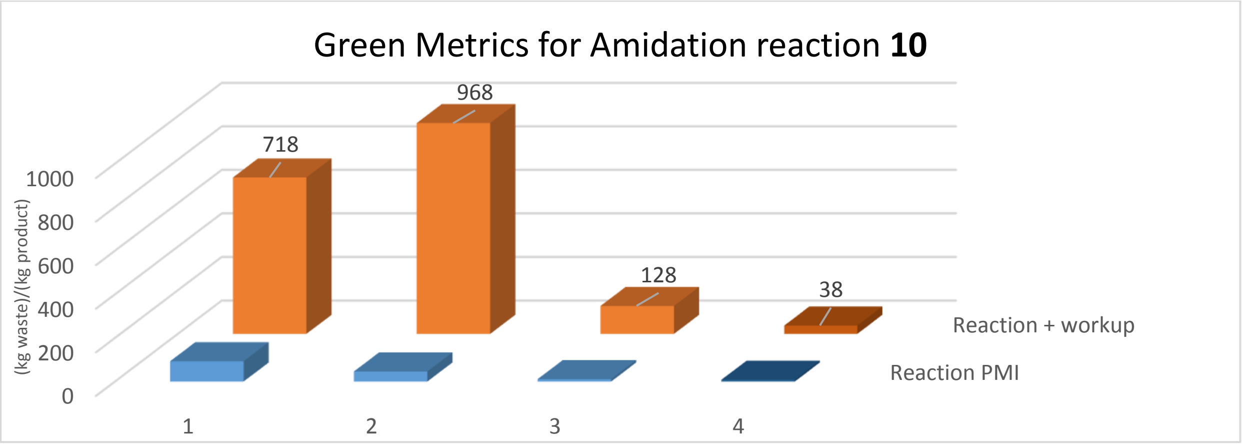

fig. S4. Green metrics for catalytic amidation protocols.

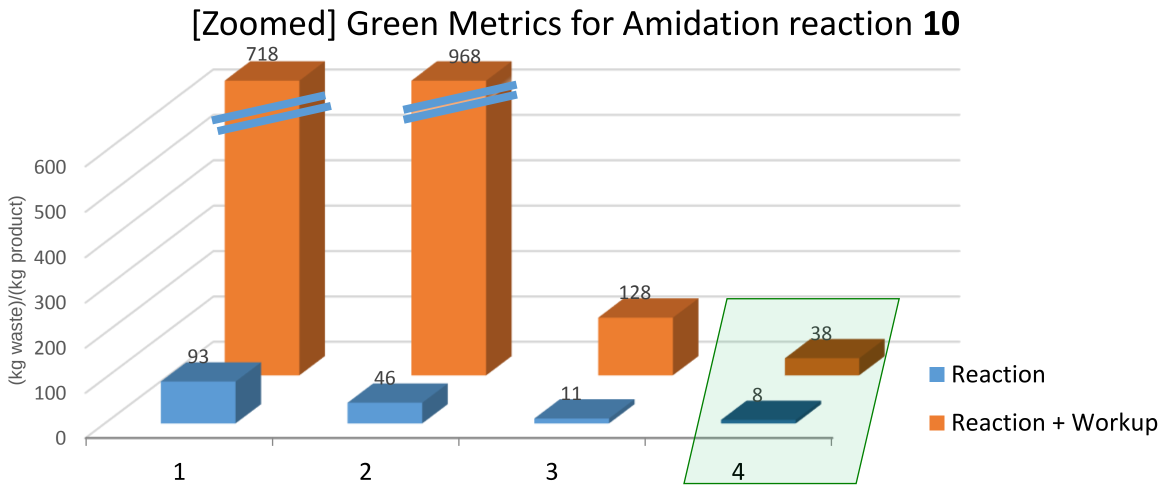

fig. S4b. [Zoomed] Green metric for amidation reaction 10.

Improved PMI's for scale-up amidation reactions

table S9. Raw data for PMI calculations.

Entry	Compound	input of reaction	input of workup	yield
**1**	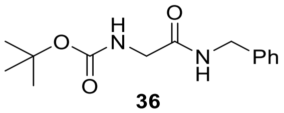 **36**	TAME (144 g) [-77 g recycled], $B(OCH_2CF_3)_3$ (3.08 g), N-BocGly (17.5 g), benzylamine (11.77 g), *total = 109.4*	*[Resin workup]* Resins (4.5 g), DMC (107 g), H_2O (1 g), $MgSO_4$ (1 g), DMC (54 g) *total= 167.5*	97% yield, 25.8 g

[*Scale up procedure*] Prepared according to general procedure A from Boc-glycine (17.5 g, 100 mmol), benzylamine (12.0 mL, 110 mmol), and $B(OCH_2CF_3)_3$ (3.08 g) in TAME (200 mL, d = 0.77g/mL), for 24 h. Upon completion, the reaction mixture was concentrated to half its volume by opening, emptying TAME from the Dean-Stark side arm trap (100 mL - recycled). The rest of the solvent was removed *in vacuo*, the product was dissolved in DMC (100 mL, d = 1.07 g/mL) and H_2O and resins IRA743 (1.5 g), A15 (2 g) and A26 OH (2 g) were added. The reaction mixture was stirred for 30 min, at which point 1 g $MgSO_4$ was added. The mixture was filtered, and the reaction flask washed twice with DMC (25 mL). The solution was concentrated *in* vacuo to yield **36** as a white solid (25.8 g, 97%). $PMI = \frac{276.9}{25.8}$*

Entry	Compound	input of reaction	input of workup	yield
**2**	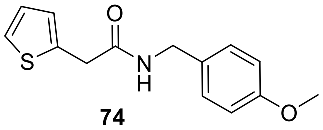 **74**	TAME (123 g) [-60 g recycled] $B(OCH_2CF_3)_3$ (2.46 g) amine (12.1 g) carboxylic acid (11.4 g) *total = 88.96*	*[Crystallisation]* 7.7 g TAME for washing filter *total = 7.7*	96% yield, 20.2 g

[*Scale up procedure*] Prepared according to general procedure A from 2-Thiopheneacetic acid (11.37 g, 80 mmol) and 4-methoxybenzylamine (12.07 g, 88 mmol) for 24 h. Upon completion, the reaction mixture was concentrated to half its volume by collecting TAME from the Dean-Stark side arm trap (80 mL – recycled). Upon cooling of the reaction mixture, the product crystallised spontaneously. The suspension was filtered and washed with DMC (7 mL) to yield **74** as a light brown scaly solid (20.20 g, 92%). $PMI = \frac{96.7}{20.20}$†

table S10. PMI calculations.

Entry	(Reaction input)/(product output) [kg]	(Reaction + workup input)/(product output) [kg]
**1**	4	11
**2**	4	5

Recycling of TAME: Recycled TAME was used for other amidation reactions without noticeable changes in yields.
* Without recycling of solvent $PMI = \frac{353.9}{25.8} = 13.7$

† Without recycling of solvent $PMI = \frac{156.7}{20.20} = 7.8$

Mechanistic studies

^{19}F NMR studies

^{19}F NMR analysis (using PhF as an internal standard) of the Dean-Stark trap and crude reaction mixture, of a reaction to prepare amide **2**, suggested that less than one equivalent of trifluoroethanol was removed from the reaction mixture over the course of the amidation reaction.

Procedure:

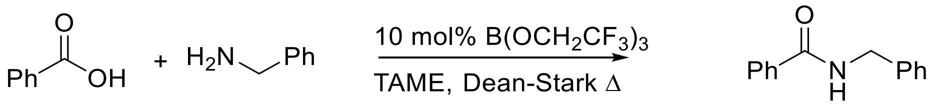

The reaction was run according to general procedure A using benzoic acid (610 mg, 5.0 mmol) and benzylamine (545 µL, 5.0 mmol) and 10 mol% $B(OCH_2CCF_3)_3$ for 24 h, at which point the reaction was brought to RT. The contents of the Dean-Stark were transferred to a 10 mL vial, to which Fluorobenzene (the internal standard, 47 µL, 0.50 mmol) was added. Fluorobenzene (the internal standard, 47 µL, 0.50 mmol) was then added to the reaction mixture. Aliquots taken from the reaction mixture and the material from the Dean Stark trap were diluted with $CDCl_3$ (50% v/v) and analysed by ^{19}F NMR (300 mHz). For improved quantification the ^{19}F NMR were run without proton decoupling and with an increased relaxation time of 20s.

Results & Discussion:

Run 1: 23% of the trifluoroethanol was in the Dean-Stark, and 77% in the reaction flask.

Run 2: 21% of the trifluoroethanol was in the Dean-Stark, and 79% in the reaction flask.

The signal for the trifluoroethanol present in the reaction mixture was broadened (top spectrum, figure SI 11). This could be interpreted as exchange between free trifluoroethanol and trifluoroethoxy groups coordinated to boron.

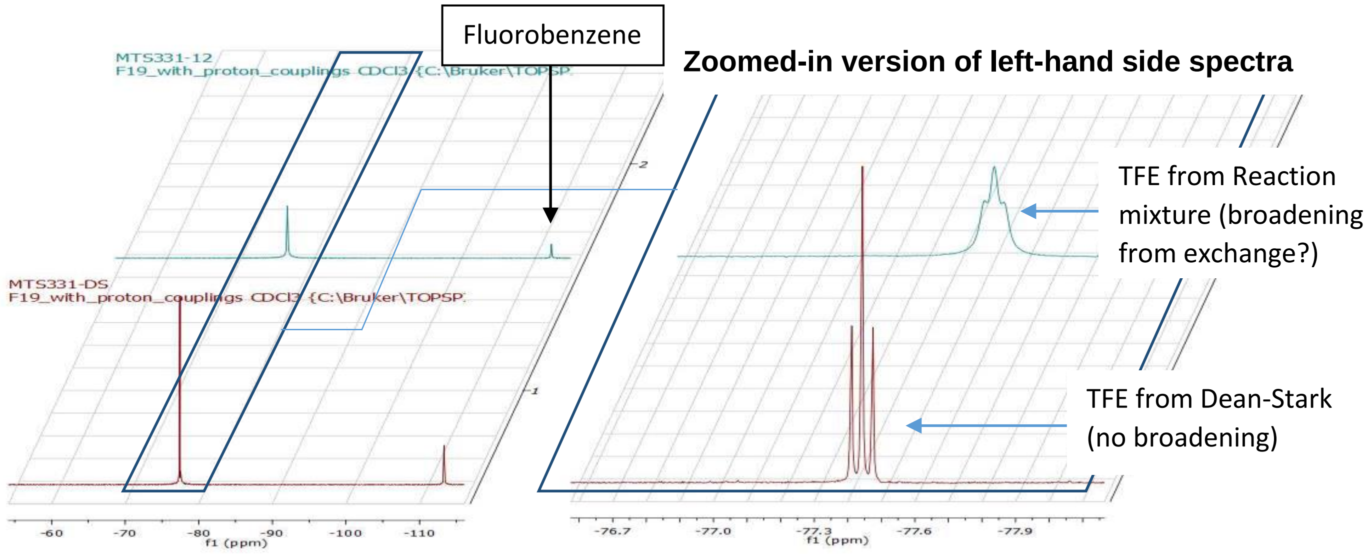

fig. S5. ^{19}F NMR spectra of the crude reaction mixture (top) and the Dean-Stark (bottom) with fluorobenzene as an internal standard.

^{11}B NMR studies

Analysis of the crude reaction mixture by ^{11}B NMR for the synthesis of amide **2**, only shows the presence of a tetrahedral boron species.

Procedure:

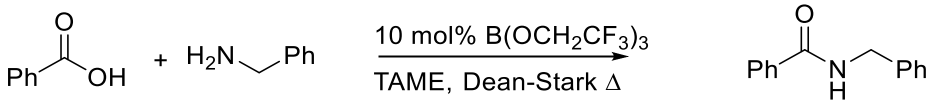

The reaction was run according to general procedure A using benzoic acid (610 mg, 5.0 mmol) and benzylamine (545 µL, 5.0 mmol) and 10 mol% $B(OCH_2CCF_3)_3$ for 24 h, at which point the reaction was brought to RT. 0.3 mL of the reaction was transferred to an NMR tube and topped up with 0.3 mL of $CDCl_3$. Spectra for $B(OCH_2CF_3)_3$ and the crude reaction mixture at 4 h and 24 h intervals are shown below.

Results & Discussion:

^{11}B NMR of $B(OCH_2CF_3)_3$ gives a signal at 17 ppm, indicating a trigonal species.

^{11}B NMR of the crude reaction mixture at various time intervals only showed the presence of tetrahedral boron species (broad peak at ~1 ppm).

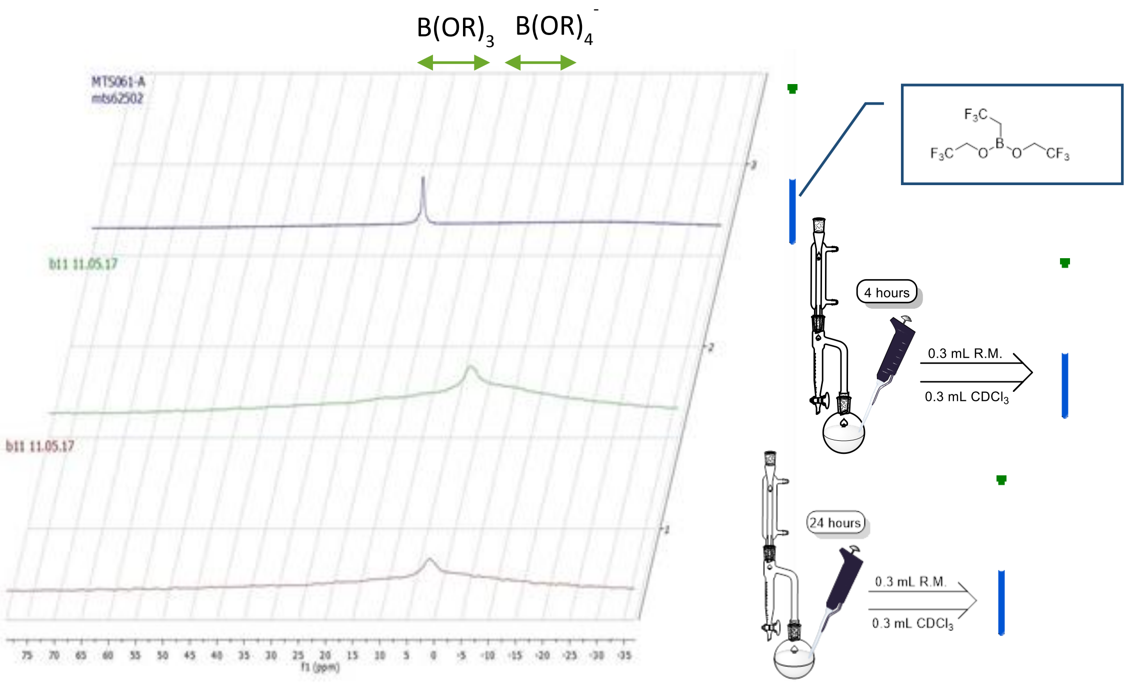

fig. S6. ^{11}B NMR spectra of $B(OCH_2CF_3)_3$ (top) and reaction mixture (bottom two) at 4- and 24-hour intervals.

Reactivity of 2,2,2-trifluoroethyl benzoate as an acylating agent

*Procedure for the synthesis of 2,2,2-trifluoroethyl benzoate **80**:*

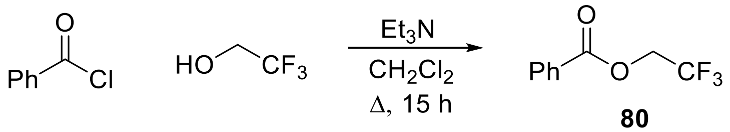

80

This method is based on a procedure described by Watson *et al.*[45]

Benzoyl chloride 3.4 mL, 30 mmol) was added dropwise to trifluoroethanol (1.4 mL, 20 mmol) and triethylamine (3.4 mL, 24 mmol) in CH_2Cl_2 (30 mL). The reaction mixture was heated to reflux for 15 h, at which point it was concentrated *in vacuo*. The resulting residue was dissolved in EtOAc (40 mL), washed with saturated $NaHCO_3$ (40 mL) and brine (40 mL), dried over anhydrous $MgSO_4$ and concentrated *in vacuo*. The crude material was purified by flash column chromatography (Pet. Ether/EtOAc, 95:5) to afford 2,2,2-trifluoroethyl benzoate **80** as a colourless liquid (3.5 g, 85%).

ν_{max} (film/cm^{-1}) 3063, 2971, 1733, 1601

^{1}H NMR (300 MHz, $CDCl_3$) δ 8.10-8.07 (m, 2H, Ar*H*), 7.62 (tt, 1H, *J* = 7.4, 1.2 Hz, Ar*H*), 7.48 (app t, *J* = 7.6 Hz, 2H, Ar*H*), 4.70 (q, *J* = 8.4 Hz, 2H, C*H*$_2$)

^{13}C NMR (75 MHz , $CDCl_3$) δ 165.1, 134.0, 130.1, 128.7, 128.6, 123.3 (q, *J* = 276 Hz), 60.9 (q, *J* = 36.5 Hz)

^{19}F NMR (282 mHz, $CDCl_3$) -73.7

LRMS (ES+): 205.0 ([M+H]$^+$, 100);

Data in agreement with the literature.[45]

*Procedure for the reaction of 2,2,2-trifluoroethyl benzoate **80*** with aniline.

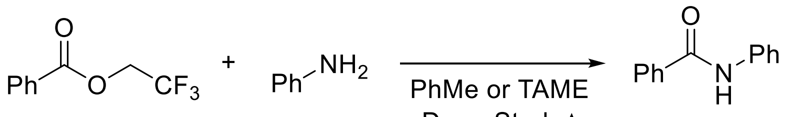

80 **4**, 0%

1) Benzoyl ester **80** (1.02 g, 5.0 mmol) and aniline (0.455 mL, 0.5 mmol) were heated at reflux in TAME (10 mL) in a round bottom flask equipped with a Dean-Stark, for 24 h. 1,4 dimethoxybenezene (0.5 mmol) was used as an internal standard.

2) Benzoyl ester **80** (1.02 g, 5.0 mmol) and aniline (0.455 mL, 0.5 mmol) were heated at reflux in Toluene (10 mL) in a round bottom flask equipped with a Dean-Stark, for 24 h. 1,4 dimethoxybenezene (0.5 mmol) was used as an internal standard.

The reactions were brought to RT and analysed by ^{1}H and ^{13}C NMR. No amide was observed in either of the two cases.

Interaction of $B(OCH_2CF_3)_3$ with methanol

The interaction was studied by the addition of MeOH (1, 2 or 3 mmol) to a solution of $B(OCH_2CF_3)_3$ (217 μL, 1 mmol) in 0.6 mL $CDCl_3$ (with 0.5 mmol 1,4 dimethoxybenzene as the internal standard).

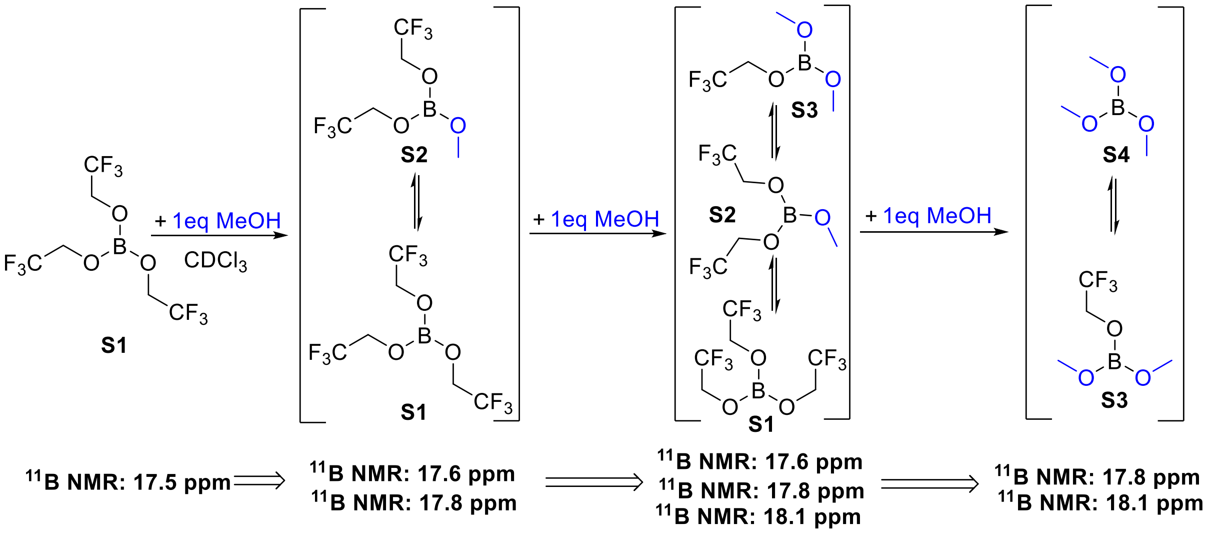

Spectroscopic analysis of these experiments indicates that with increasing amounts of methanol being added to the solution, exchange of alkoxy groups on the boron occurs to give mixed borate esters.

^{11} BNMR data

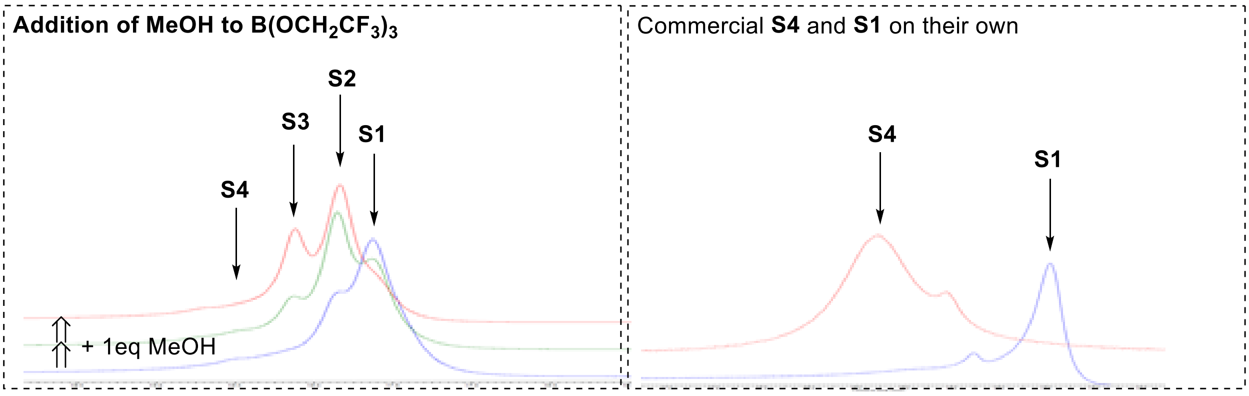

^{1}H NMR data

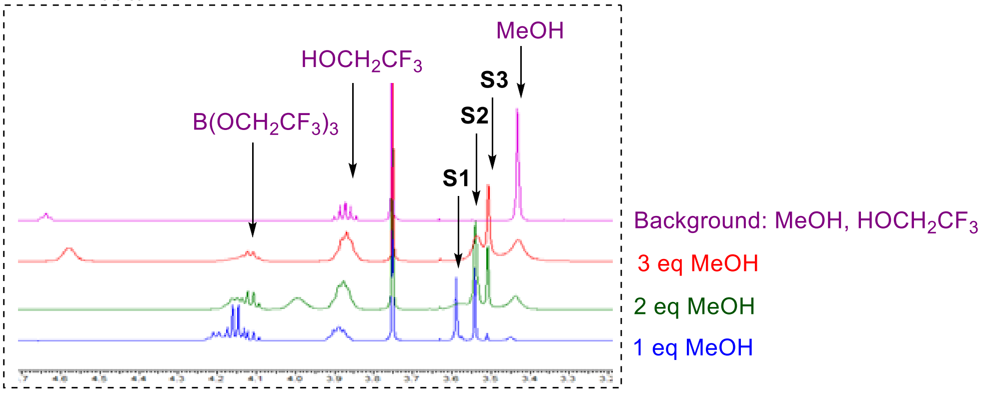

Interaction of $B(OCH_2CF_3)_3$ with H_2O

The interaction was studied by the addition of H_2O (1, 2 or 3 mmol) to a solution of $B(OCH_2CF_3)_3$ (217 μL, 1 mmol) in 0.6 mL $CDCl_3$ (with 0.5 mmol 1,4 dimethoxybenzene as the internal standard).

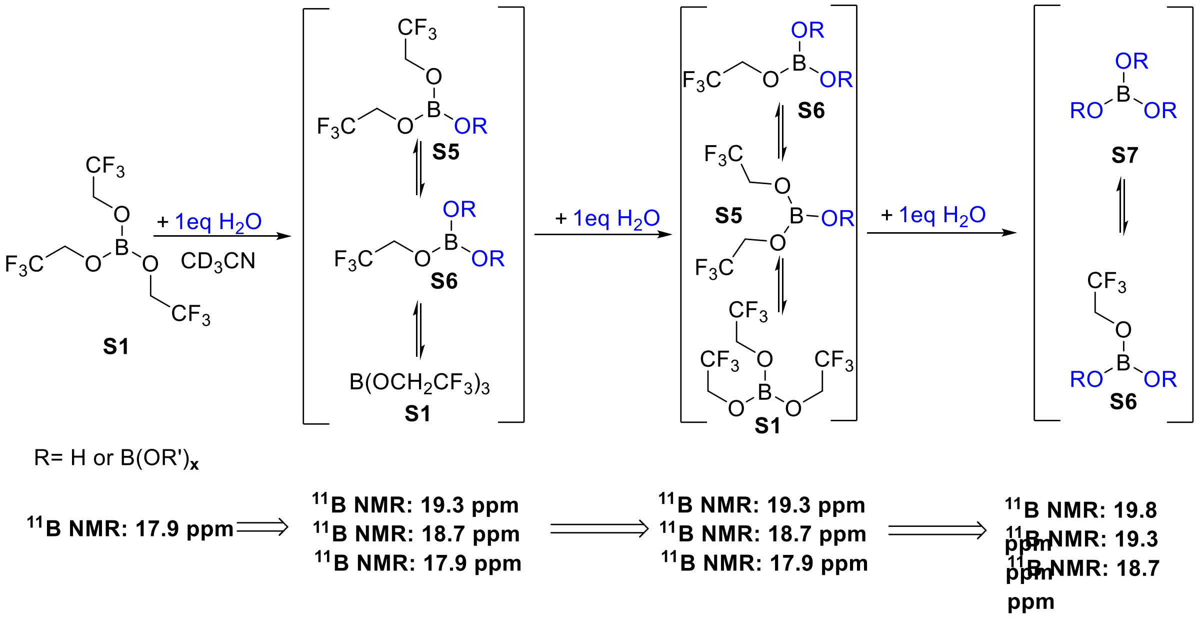

Spectroscopic analysis of these experiments indicates that with increasing amounts of water being added to the solution, partial hydrolysis occurs.

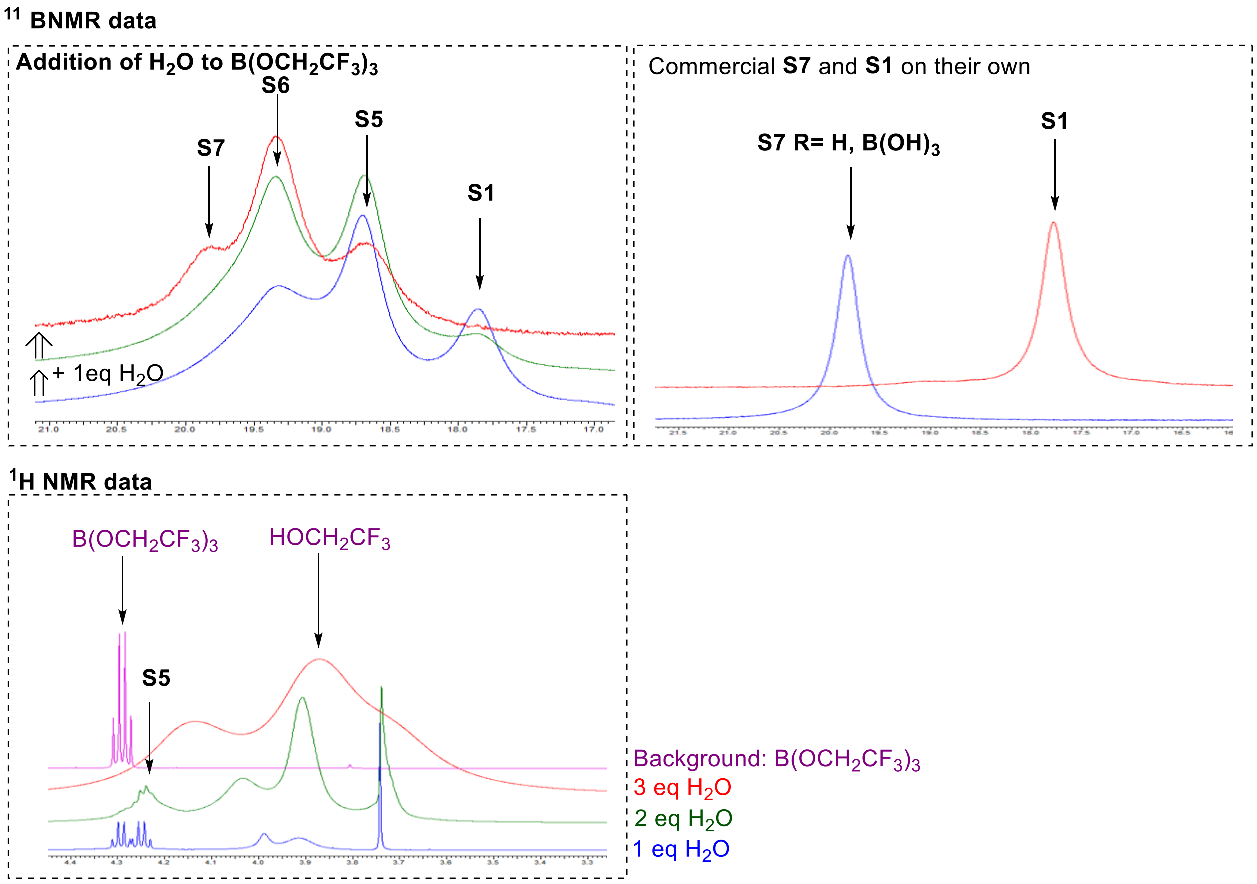

Interaction of Borate with Carboxylic Acid and Amine:

The interaction was studied by the addition of amine (1 mmol) to a solution of $B(OCH_2CF_3)_3$ (217 µL, 1 mmol) in 0.6 mL CD_3CN (with 0.5 mmol 1,4 dimethoxybenzene as the internal standard). Formation of a tetrahedral borate species was observed.

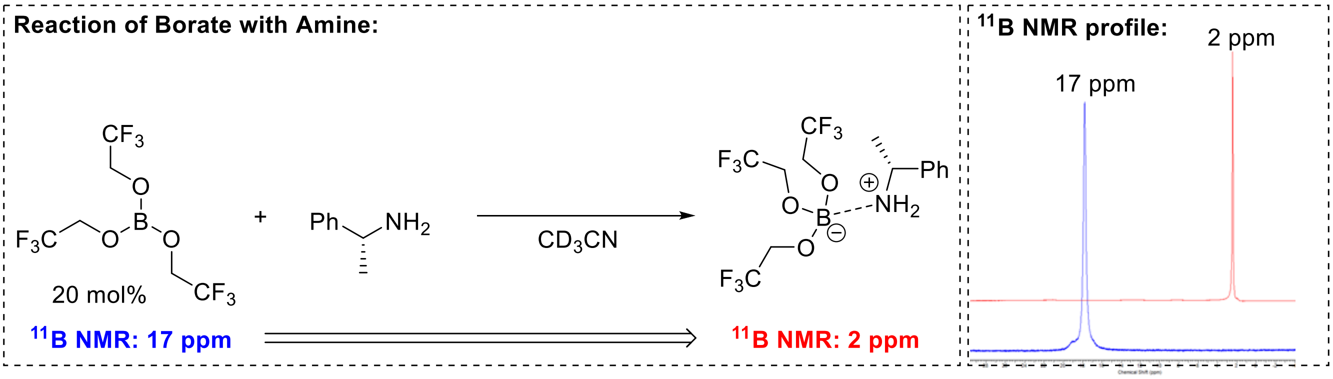

The interaction was studied by the addition of phenylacetic acid (1 mmol) to a solution of $B(OCH_2CF_3)_3$ (217 µL, 1 mmol) in 0.6 mL $CDCl_3$ (with 0.5 mmol 1,4 dimethoxybenzene as the internal standard). No change in chemical shift was observed.

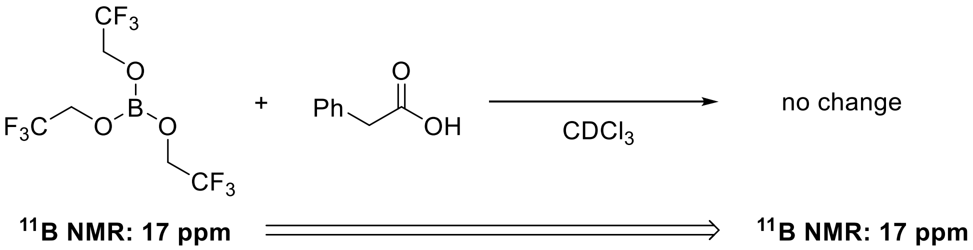

Kinetic Analysis

The graphical method developed by Burés was used to determine the reaction orders from concentration profiles. This method uses a variable normalization of the time scale to enable the visual comparison of entire concentration profiles, allowing for the order in each component of the reaction to be determined.

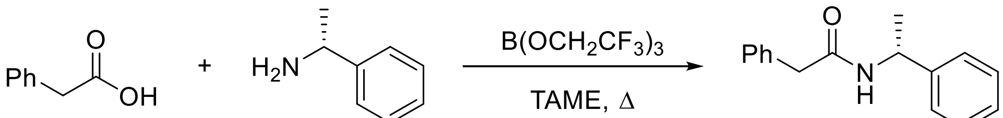

6

Representative example. A reaction was carried out following general procedure A from phenylacetic acid (0.681 g, 5.0 mmol), (S)-(−)-α-Methylbenzylamine (0.645 mL, 5.0 mmol) and 1,4 dimethoxybenzene (69 mg, 0.5 mmol) as the internal standard. The reaction was monitored by the collection of aliquots (every 40 or 60 min) until it had reached completion (~6 hours)

Raw data for determination of order of Catalyst

For determination of the order in catalyst, the reaction was run with 5 mol%, 10 mol% and 15 mol% $B(OCH_2CF_3)_3$. A plot of the concentration of product vs. time is shown below:

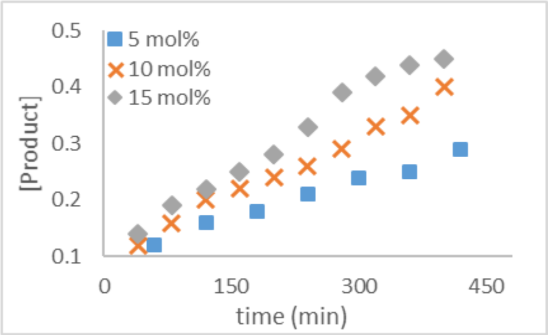

Graph S1: Effects of catalyst loading

Raw data for determination of order of Amine

For determination of the order in amine, the reaction was run with 1.0 eq, 1.6 eq and 2.0 eq of (S)-(−)-α-Methylbenzylamine. A plot of the concentration of product vs. time is shown below:

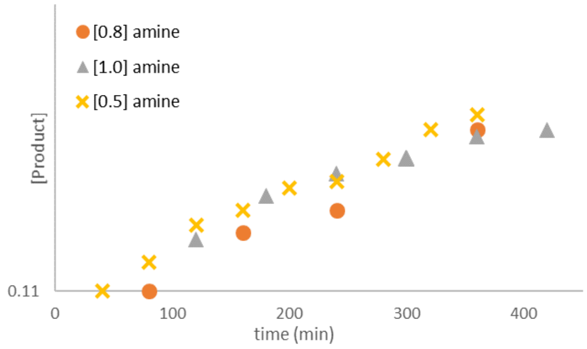

Graph S2: Effects of excess amine

Raw data for determination of order of Acid

For determination of the order in carboxylic acid, the reaction was run with 1.0 eq, 1.2 eq, 1.4 eq of phenylacetic acid. A plot of the concentration of product vs. time is shown below:

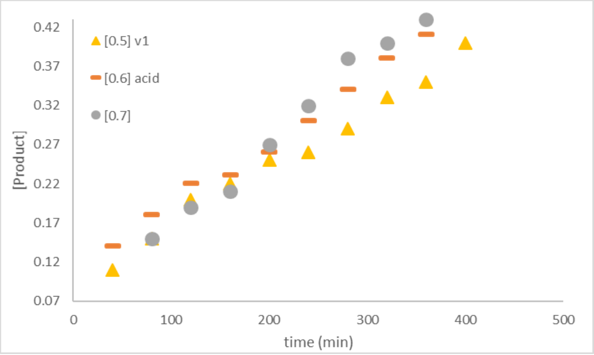

Graph S3: Effects of excess acid: Data set 1

Due to the graphical nature of the kinetic analysis, it was difficult to unambiguously determine the order of reaction with respect to acid concentration. For this reason, the reaction was repeated with 1.0 eq and 1.6 eq of phenylacetic acid to obtain further data. A plot of the concentration of product vs. time is shown below:

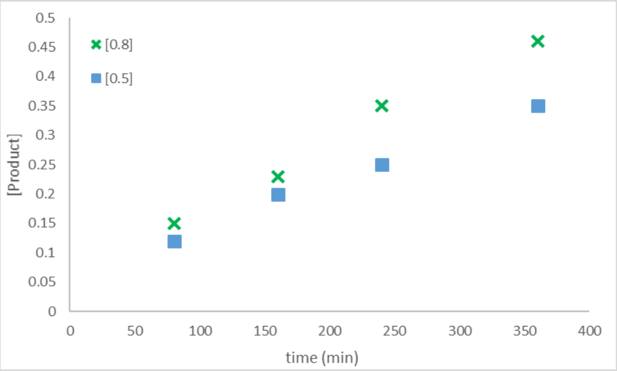

Graph S4: Effects of excess acid: Data set 2

Reproducibility of data:

Two experiments were run with varying times for collection of aliquots. The two reactions displayed excellent reproducibility.

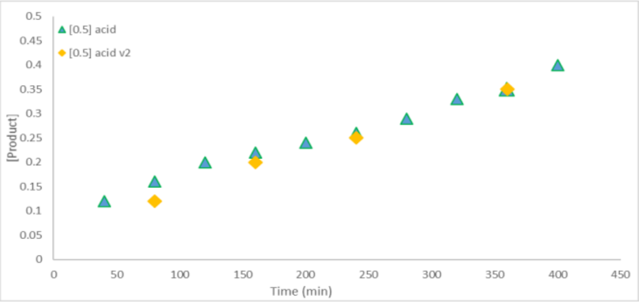

Graph S5: Reproducibility of data

Assuming the catalyst is not significantly deactivated during the course of the reaction, the order with respect to borate catalyst can be determined by plotting [product] against $t[cat]^{\alpha}$.

Order in catalyst = 0.8

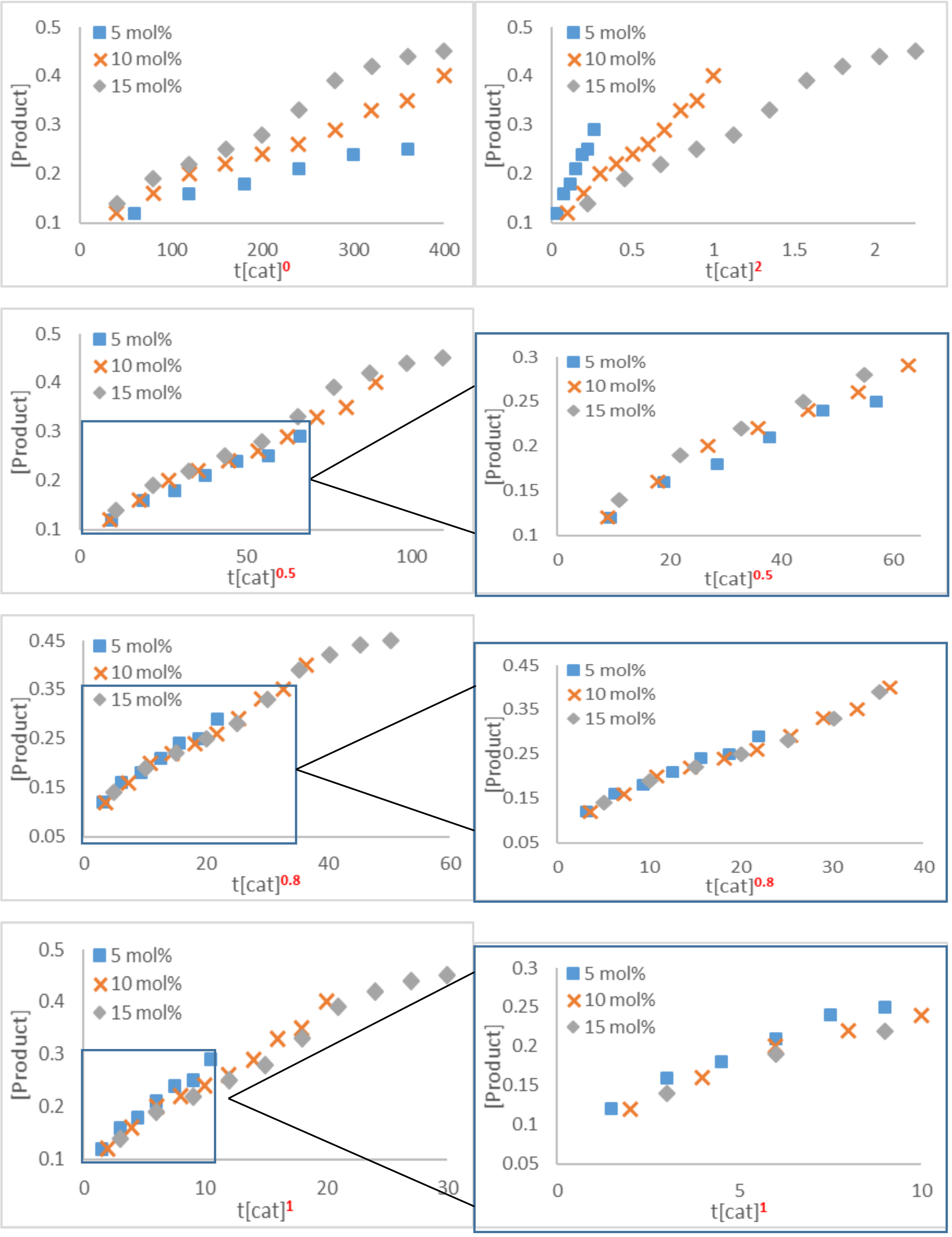

Graphs S6 a-h: Data from **tables S11-13** plotted against $t[cat]^{n}$ where **a)** n = 0 **b)** n = 2 **c)** n = 0.5 **d)** n = 0.5 [zoomed in] **e)** n = 0.8 **f)** n = 0.8 [zoomed in] **g)** n = 1.0 **g)** n = 1.0 [zoomed in]

The graphical method developed by Burés was used to plot [Product] against the variable time scale normalized in amine ($\sum[\text{Amine}]^{\alpha}\Delta t$) or carboxylic acid ($\sum[\text{Acid}]^{\alpha}\Delta t$). When α is the correct order in [Amine] or [Acid] the traces will overlay

$$\int_{t=0}^{t=n} [\mathrm{A}]^{\alpha} \mathrm{d}t = \sum_{i=1}^{n} \left(\frac{[\mathrm{A}]_i + [\mathrm{A}]_{i-1}}{2} \right)^{\alpha} (t_i - t_{i-1})$$

Order in [Amine] = 0

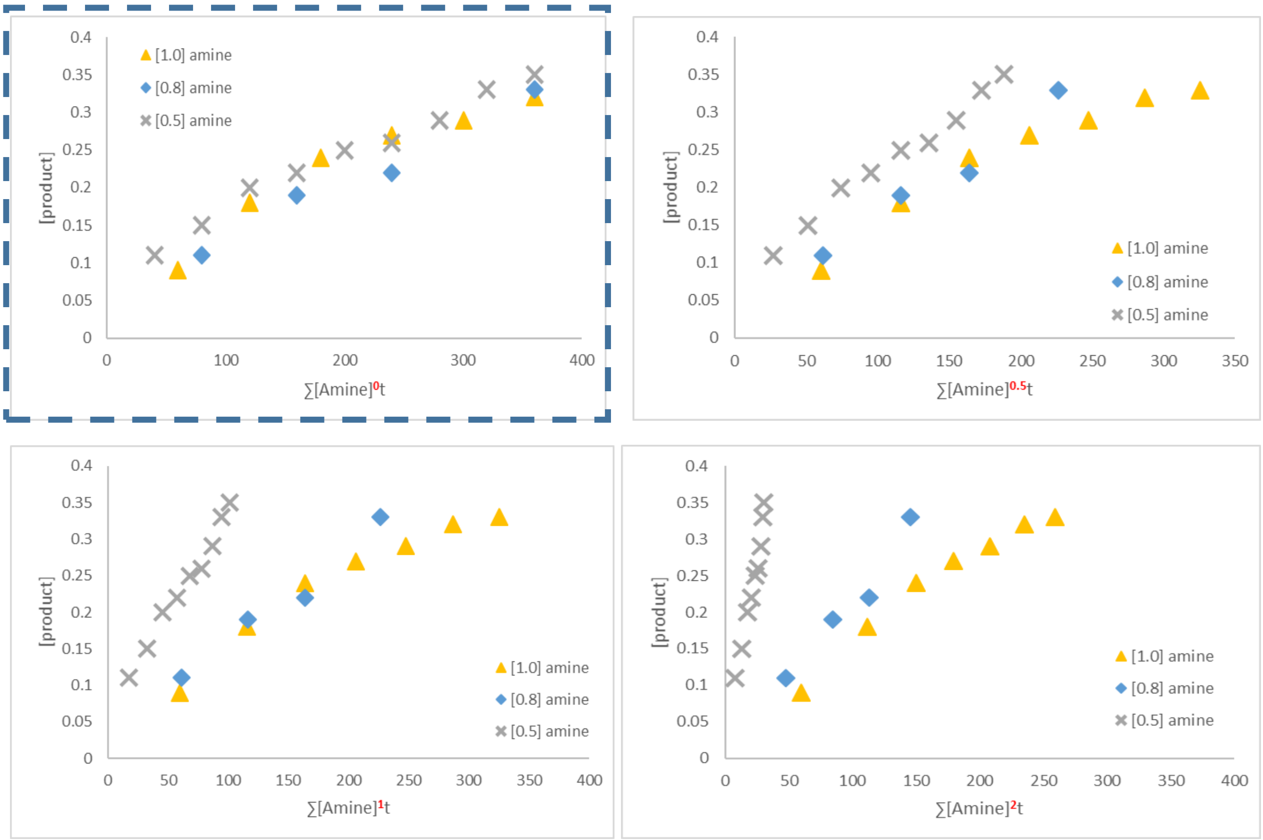

Graphs S7 a-d: Data from **tables S14-S16** plotted against ($\sum[\text{Amine}]^{n}\Delta t$) where **a)** n = 0 **b)** n = 0.5 **c)** n = 1.0 **d)** n = 2.0

Order in [Acid] = 0.5 (first set of data)

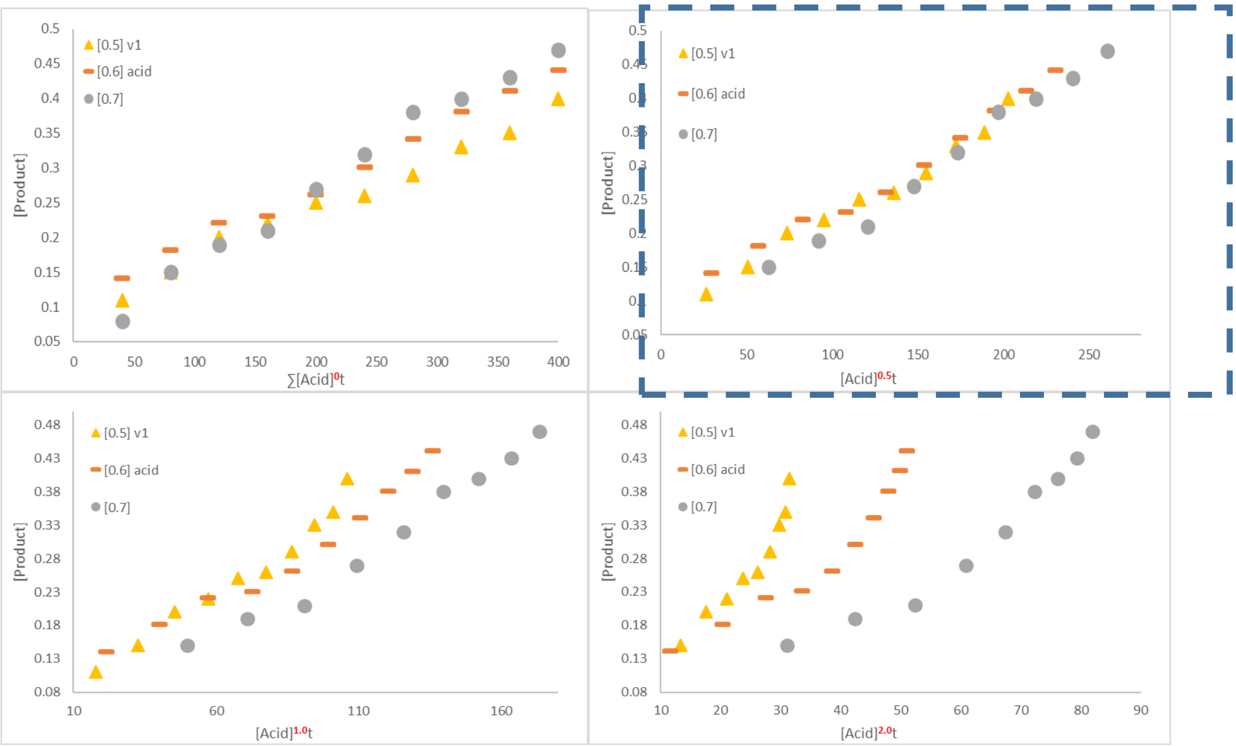

Graphs S8 a-d: Data from **tables S17-S19** plotted against ($\sum[\text{Acid}]^{n}\Delta t$) where **a)** n = 0 **b)** n = 0.5 **c)** n = 1.0 **d)** n = 2.0

Order in [Acid] = 0.5 (second set of data)

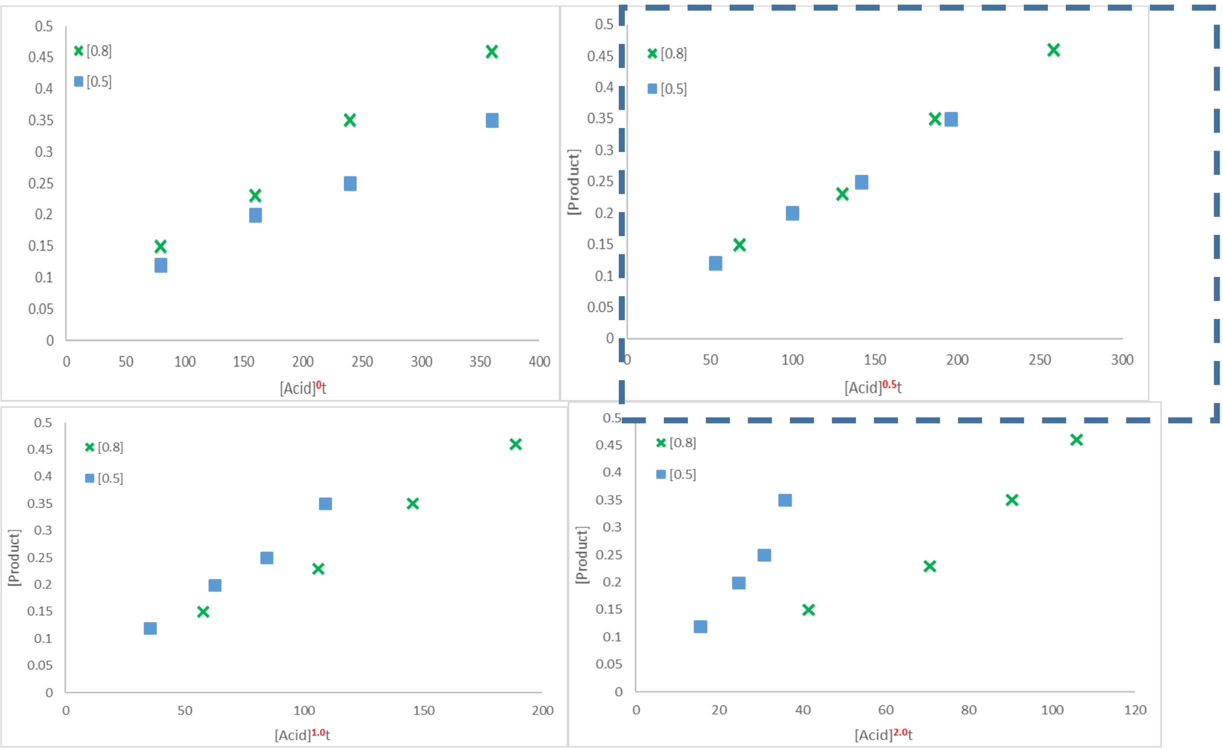

Graphs S9 a-d: Data from **tables S20-S21** plotted against ($\sum[\text{Acid}]^{n}\Delta t$) where **a)** n = 0 **b)** n = 0.5 **c)** n = 1.0 **d)** n = 2.0

For determination of order in catalyst, the reaction was run with 5 mol%, 10 mol% and 15 mol% $B(OCH_2CF_3)_3$.

Data for [acid] = 0.5, [amine] = 0.5 with either [catalyst] = 0.025, [catalyst] = 0.05 or [catalyst] = 0.075

table S11. Raw data for determination of order in catalyst.

10 mol% $B(OCH_2CF_3)_3$ **[catalyst] = 0.05 M**	
Time (h)	[Product]
0	0.12
80	0.16
120	0.20
160	0.22
200	0.24
240	0.26
280	0.29
320	0.33
360	0.35
400	0.40

table S12. Raw data for determination of order in catalyst.

5 mol% $B(OCH_2CF_3)_3$ **[catalyst] = 0.025 M**	
Time (h)	[Product]
60	0.12
120	0.16
180	0.18
240	0.21
300	0.24
360	0.25
420	0.29

table S13. . Raw data for determination of order in catalyst.

15 mol% $B(OCH_2CF_3)_3$ **[catalyst] = 0.075 M**	
Time (h)	[Product]
40	0.14
80	0.19
120	0.22
160	0.25
200	0.28
240	0.33
280	0.39
320	0.42
360	0.44
400	0.45

For determination of order in amine, the reaction was run with 1.0 eq, 1.6 eq and 2.0 eq of (S)-(−)-α-Methylbenzylamine.

Data below for [acid] = 0.5, [catalyst] = 0.05, with either [amine] = 0.5, [amine] = 0.8 or [amine] = 1.0

table S14. Raw data for determination of order in amine.

	1.0 eq amine **[amine] = 0.5 M**	
Time (h)	[Amine]	[Product]
40	0.39	0.12
80	0.35	0.16
120	0.30	0.20
160	0.28	0.22
200	0.25	0.24
240	0.24	0.26
280	0.21	0.29
320	0.18	0.33
360	0.15	0.35
400	0.10	0.40

table S15. Raw data for determination of order in amine.

1.6 eq amine **[amine] = 0.8 M**		
Time (h)	[Amine]	[Product]
80	0.74	0.11
160	0.62	0.19
240	0.58	0.22
360	0.46	0.33

table S16. Raw data for determination of order in amine.

2.0 eq amine **[amine] = 1.0 M**		
Time (h)	[Amine]	[Product]
60	0.96	0.09
120	0.90	0.18
180	0.73	0.24
240	0.70	0.27
300	0.68	0.29
360	0.65	0.32
420	0.63	0.33

Raw data for determination of order of Acid

For determination of order in carboxylic acid, the reaction was run with 1.0 eq (above), 1.2 eq, 1.4 eq of phenylacetic acid.

Data set 1:

table S17. Raw data for determination of order in acid.

Time (h)	1.0 eq acid **[acid] = 0.5 M** [Acid]	[Product]
40	0.39	0.12
80	0.35	0.16
120	0.30	0.20
160	0.28	0.22
200	0.25	0.24
240	0.24	0.26
280	0.21	0.29
320	0.18	0.33
360	0.15	0.35
400	0.10	0.40

table S18. Raw data for determination of order in acid.

	1.2 eq acid **[acid] = 0.6 M**	
Time (h)	[Acid]	[Product]
40	0.48	0.14
80	0.45	0.18
120	0.4	0.22
160	0.38	0.23
200	0.32	0.26
240	0.30	0.30
280	0.26	0.34
320	0.24	0.38
360	0.19	0.41
400	0.16	0.44

table S19. Raw data for determination of order in acid.

1.4 eq acid **[acid] = 0.7 M**	
[Acid]	[Product]
0.62	0.08
0.55	0.15
0.51	0.19
0.49	0.21
0.43	0.27
0.38	0.32
0.32	0.38
0.31	0.40
0.27	0.43
0.23	0.47

Data set 2:

table S20. Raw data for determination of order in acid.

	1.0 eq acid **[acid] = 0.5 M**	
Time (h)	[Acid]	[Product]
80	0.38	0.12
160	0.3	0.20
240	0.25	0.25
320	0.16	0.35

table S21. Raw data for determination of order in acid.

1.6 eq acid **[acid] = 0.8 M**	
[Acid]	[Product]
0.64	0.15
0.57	0.23
0.42	0.35
0.30	0.46

Spectroscopic data

Preparation of Tris(2,2,2-trifluoroethyl)borate

100 g scale: A suspension of B_2O_3 (100.0 g, 1.44 mol) in 2,2,2-trifluoroethanol (210 mL, 2.88 mol) was stirred at 80 °C for 24 h. The reaction mixture was allowed to cool and then filtered on a sinter funnel through a pad of celite (9.5 × 2 cm) into a 250 mL round bottom flask to remove excess boric anhydride. The filtrate was purified by fractional distillation (bp CF_3CH_2OH, 77-80 °C, bp of $B(OCH_2CF_3)_3$ 125-129 °C) to give $B(OCH_2CF_3)_3$ as a clear liquid (108 g, 0.19 mol, 44%); Unreacted 2,2,2-trifluoroethanol can be recovered from the distillation.

bp 125-129 °C;

ν_{max} (film/cm^{-1}) 3168, 1440, 1380;

^{1}H NMR (600 MHz, $CDCl_3$) δ 4.23 (q, *J* = 8.6 Hz, 6H);

^{13}C NMR (150 MHz , $CDCl_3$) δ 61.8 (q, *J* = 36.3 Hz), 123.3 (q, *J* = 278.4 Hz); LRMS (EI) 309 ([M]$^+$, 100);

Data in agreement with the literature.[32]

Note: The reaction should be conducted on a scale >25 g to facilitate purification by distillation.

N-Benzylbenzamide (2)

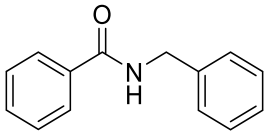

Prepared according to general procedure A from benzoic acid (610 mg, 5.0 mmol) and benzylamine (545 µL, 5.0 mmol) for 28 h, and purified using the standard resin workup procedure to yield **1** a white solid (996 mg, 94%);

mp 100-101 °C [Lit.[28] 100-101 °C];

ν_{max} (solid/cm^{-1}) 3284, 1636, 1548, 1489;

^{1}H NMR (600 MHz, $CDCl_3$) δ 7.90–7.76 (m, 2H, Ar*H*), 7.52–7.46 (m, 1H, Ar*H*), 7.45–7.39 (m, 2H, Ar*H*), 7.37–7.32 (m, 4H, Ar*H*), 7.32–7.26 (m, 1H, Ar*H*), 6.56 (br s, 1H, N*H*), 4.63 (d, *J* = 5.1 Hz, 2H, C*H*$_2$);

^{13}C NMR (150 MHz, $CDCl_3$) δ 167.5, 138.3, 134.5, 131.6, 128.9, 128.7, 128.0, 127.7, 127.1, 44.2

LRMS (ES+): 212.1 ([M+H]$^+$, 100);

HRMS (ES+): measured 212.1085, ([$C_{14}H_{13}NO$+H]$^+$), requires 212.10754.

Data in agreement with the literature.[28]

N-Benzyl-3-methylbutanamide (3)

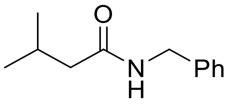

Prepared according to general procedure A from isovaleric acid (200 mg, 2.0 mmol) and benzylamine (240 µL, 2.2 mmol) for 24 h, and purified using the standard resin workup procedure to yield **3** as a white solid (364 mg, 95%).

mp 58-59 °C [Lit.[28] 58-59 °C]

ν_{max} (solid/cm^{-1}) 3288, 2957, 1633, 1545, 1543;

^{1}H NMR (500 MHz, $CDCl_3$) δ 7.34-7.26 (m, 5H, Ar*H*), 5.8 (br s, 1H, N*H*), 4.43 (d, *J* = 5.8 Hz, 2H, NHC*H*$_2$), 2.11-2.15 (m, 1H, C*H*), 2.07 (d, *J* = 7.6 Hz, 2H, CHC*H*$_2$), 0.95 (d, *J* = 6.5 Hz, 6H, (C*H*$_3$)$_2$);

^{13}C NMR (125 MHz, $CDCl_3$) δ 175.7, 141.7, 132.0, 131.1, 130.8, 49.4, 46.9, 29.5, 25.8;

LRMS (ES+): 192.1 ([M]$^+$, 50), 383.3 ([2×M]$^+$, 100);

HRMS (ES+): measured 192.1383, ([$C_{12}H_{17}NO$+H]$^+$), requires 192.1388

Data in agreement with the literature.[28]

N-Phenylbenzamide (4)

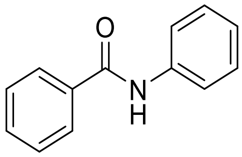

Prepared according to general procedure A from benzoic acid (244 mg, 2.0 mmol) and aniline (200 µL, 2.2 mmol) in PhMe for 28 h, and purified using the standard resin workup procedure to yield **3** as colourless crystals (349 mg, 88%);

mp 160-161 °C [Lit.[49] 158-160 °C];

ν_{max} (solid/cm^{-1}) 3341, 2995, 1653, 1597, 1523;

^{1}H NMR (600 MHz, $CDCl_3$) δ 7.9 (br s, 1H, N*H*), 7.89-7.86 (m, 2H, Ar*H*), 7.66 (d, *J* = 7.7 Hz, 2H, Ar*H*), 7.56 (tt, *J* = 7.4, 2.0 Hz, 1H, Ar*H*), 7.50-7.48 (m, 2H, Ar*H*), 7.39-7.37 (m, 2H, Ar*H*), 7.16 (t, *J* = 7.4 Hz, 1H, Ar*H*);

^{13}C NMR (150 MHz, $CDCl_3$) δ 165.9, 138.0, 135.1, 132.0, 129.2, 128.9, 127.1, 124.7, 120.3;

LRMS (ES+): 198.1 ($[M+H]^+$, 100), 220.1 ($[M+Na]^+$, 20);

HRMS (ES+): measured 198.0911, ($[C_{13}H_{11}NO+H]^+$), requires 198.0913.

Data in agreement with the literature.[49]

N,2-Diphenylacetamide (5)

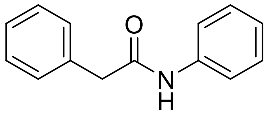

Prepared according to general procedure A from phenylacetic acid (272 mg, 2.0 mmol) and aniline (200 µL, 2.2 mmol) for 24 h, and purified using the standard resin workup procedure to yield **5** as an off-white solid (347 mg, 82%);

mp 119-120 °C [Lit.[29] 116-118 °C];

ν_{max} (solid/cm^{-1}) 3252, 1655, 1597, 1543;

^1H NMR (600 MHz, $CDCl_3$) δ 7.44-7.39 (m, 4H, Ar*H*), 7.35-7.33 (m, 3H, Ar*H*), 7.27 (t, *J* = 7.6 Hz, 3H, Ar*H*, N*H*), 7.09 (t, *J* = 7.4 Hz, 1H, Ar*H*), 3.74 (s, 2H, CH_2);

^{13}C NMR (150 MHz, $CDCl_3$) δ 169.3, 137.7, 134.6, 129.7, 129.4, 129.1, 127.8, 124.6, 120.0, 44.9;

LRMS (ES+): 212.1 ($[M+H]^+$, 100); 234.1 ($[M+Na]^+$, 100);

HRMS (ES+): measured 212.1076 ($[C_{14}H_{13}NO+H]^+$), requires 212.1076.

Data in agreement with the literature. [29]

(*R*)-2-Phenyl-N-(1-phenylethyl)acetamide (6)

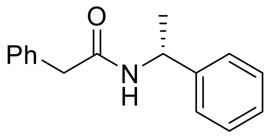

Prepared according to general procedure A from phenylacetic acid (680 mg, 5.0 mmol) and (*R*)-(−)-α-Methylbenzylamine (645 µL, 5.0 mmol) for 16 h, and purified using the standard resin workup procedure to yield **6** as a white solid (1.15 g, 96%).

$[\alpha]_D^{20}$+1.1 (*c* 1.0, $CHCl_3$) [Lit.[28] $[\alpha]_D^{25}$+3.3 (*c* 1.0, $CHCl_3$)];

mp 120-121 °C [Lit.[24] 116-117 °C];

ν_{max} (solid/cm^{-1}) 3305, 3063, 3028, 2973, 2927, 1638, 1536

^{1}H NMR (500 MHz, $CDCl_3$) δ 7.37-7.34 (m, 2H, Ar*H*), 7.31-7.28 (m, 3H, Ar*H*), 7.26-7.23 (m, 3H, Ar*H*), 7.19-7.18 (m, 2H, Ar*H*), 5.6 (br s, 1H, N*H*), 5.12 (q, *J* = 7.3 Hz, 1H, C*H*CH_3), 3.58 (s, 2H, CH_2CO), 1.39 (d, *J* = 6.9 Hz, 3H, CH_3)

^{13}C NMR (125 MHz, $CDCl_3$) δ 170.1, 143.1, 135.0, 129.5, 129.1, 128.7, 127.4, 127.4, 126.0, 48.8, 44.0, 21.9

LRMS (ES+): 240.1 ($[M+H]^+$, 50), 479.31 ($[M+H]^+$, 100);

HRMS (ES+): measured 240.1388, ($[C_{16}H_{17}NO+H]^+$), requires 240.1388

Enantiopurity was determined by chiral HPLC to be >99:1 er : Chiral HPLC OD1 column, 90:10 hexane:^{i}PrOH, flow 0.8 mL/min, λ = 218 nm, 20 °C, t_R= 17.1 min (R enantiomer), and 21.0 min (S enantiomer).

Compound was also prepared according to general procedure A, using $B(OMe)_3$ as the catalyst with phenylacetic acid (680 mg, 5.0 mmol) and (R)-(−)-α-Methylbenzylamine (645 µL, 5.0 mmol) for 16 h, to yield **6** as a white solid (0.93 g, 76%). Spectroscopic data and chiral purity were identical.

Data in agreement with the literature.[28]

***N*-(2-Methylbenzyl)-2-phenylacetamide (7)**

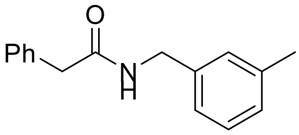

Prepared according to general procedure A from phenylacetic acid (680 mg, 5.0 mmol) and *3*-methylbenzylamine (689 µL, 5.5 mmol) for 24 h, and crystallised in reaction flask upon cooling of reaction mixture to yield **7** as a white solid (1.20 g, 96%).

mp 85-86 °C [Lit.[50] 86-88 °C];

ν_{max} (solid/cm^{-1}) 3232, 3062, 3030, 29.13, 1626, 1548

^{1}H NMR (500 MHz, $CDCl_3$) δ 7.36-7.33 (m, 2H, Ar*H*), 7.30-7.26 (m, 3H, Ar*H*), 7.18 (t, *J* = 7.4 Hz, 1H, Ar*H*), 7.05 (d, *J* = 7.3 Hz, 1H, Ar*H*), 6.98-6.96 (m, 2H, Ar*H*), 5.7 (br s, 1H, N*H*), 4.37 (d, *J* = 5.8 Hz, 2H, CH_2NH), 3.63 (s, 2H, CH_2CO), 2.31 (s, 3H, CH_3)

^{13}C NMR (125 MHz, $CDCl_3$) δ 170.9, 138.5, 138.1, 134.9, 129.5, 129.1, 128.6, 128.3, 128.2, 127.5, 124.6, 43.9, 43.6, 21.4

LRMS (ES+): 240.1 ([M+H]$^+$, 20), 487.2 ([2×M+H]$^+$, 100)

Data in agreement with the literature. [50]

***N*-(4-Methoxybenzyl)-2-phenylacetamide (8)**

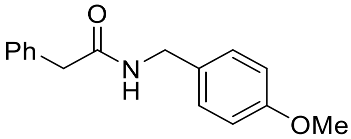

Prepared according to general procedure A from phenylacetic acid (680 mg, 5.0 mmol) and *p*-methoxybenzylamine (685 µL, 5.0 mmol) for 12 h, and crystallised in reaction flask upon cooling of reaction mixture to yield **8** as a fluffy white solid (1.28 g, 89%).

mp 143-144 °C [Lit.[29] 139-141 °C];

ν_{max} (solid/cm^{-1}) 3234, 3062, 3031, 2841, 1649, 1610, 1544

^{1}H NMR (400 MHz, DMSO-d_6, *[note: rotamers observed in $CDCl_3$]*) δ 8.46 (br t, *J* = 5.5 Hz, 1H, N*H*), 7.31-7.22 (m, 5H, Ar*H*), 7.15 (d, *J* = 8.8 Hz, 2H, Ar*H*), 6.86 (d, *J* = 8.5 Hz, 2H, Ar*H*), 4.20 (d, *J* = 5.8 Hz, 2H, CH_2NH), 3.72 (s, 3H, OCH_3); 3.45 (s, 2H, CH_2CO)

^{13}C NMR (125 MHz, $CDCl_3$) δ 170.8, 159.0, 134.9, 130.3, 129.3, 129.1, 129.0, 127.5, 114.1, 55.4, 43.9, 43.2

LRMS (ES+): 256.1 ([M+H]$^+$, 100);

HRMS (ES+): measured 256.1336, $[C_{16}H_{17}NO_2+H]^+$, requires 256.1338

Data in agreement with the literature.[29]

_N_-(4-Chlorobenzyl)-2-phenylacetamide (9):

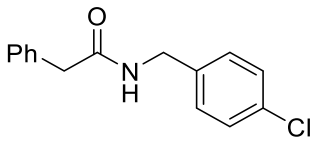

Prepared according to general procedure A from phenylacetic acid (680 mg, 5.0 mmol) and *4*-Chlorobenzylamine (668 µL, 5.5 mmol) for 24 h, and crystallised in reaction flask upon cooling of reaction mixture to yield **9** as an off-white solid (1.12 g, 86%).

mp 135-136 °C

ν_{max} (solid/cm^{-1}) 3232, 3062, 2968, 1649, 1632

^{1}H NMR (500 MHz, $CDCl_3$) δ 7.36-7.33 (m, 2H, Ar*H*), 7.29 (d, *J* = 7.4 Hz, 1H, Ar*H*), 7.26-7.24 (m, 4H, Ar*H*), 7.09 (d, *J* = 8.4 Hz, 2H, Ar*H*), 5.8 (br s, 1H, N*H*), 4.35 (d, *J* = 6.0 Hz, 2H, CH_2N), 3.61 (s, 2H, CH_2CO)

^{13}C NMR (125 MHz, $CDCl_3$) δ 171.1, 136.8, 134.7, 133.3, 129.5, 129.2, 128.9, 128.8, 127.6, 43.8, 42.9

LRMS (ES+): 260.1 (^{35}Cl[M+H]$^+$, 100), 262.1 (^{37}Cl [M+H]$^+$, 20);

HRMS (ES+): measured 260.0848, ($[C_{15}H_{14}ClNO+H]^+$), requires 260.0842

Data in agreement with the literature.[50]

***N*-Benzyl-2-phenylacetamide (10)**

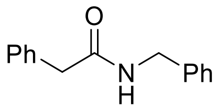

Prepared according to general procedure A (5 mol% $B(OCH_2CF_3)_3$) from phenylacetic acid (681 mg, 5.0 mmol) and benzylamine (546 µL, 5.0 mmol) for 6 h, and purified using the standard resin workup procedure to yield **10** as a white solid (1.12 g, 99%).

mp 120-121 °C [Lit.[51] 119-120 °C];

ν_{max} (solid/cm^{-1}) 3284, 3030, 1635, 1544

^{1}H NMR (400 MHz, $CDCl_3$) δ 7.37-7.23 (m, 8H, Ar*H*), 7.19-7.17 (m, 2H, Ar*H*), 5.8 (br s, 1H, N*H*), 4.41 (d, *J* = 6.0 Hz, 2H, C*H*$_2$NH), 3.62 (s, 2H, COC*H*$_2$);

^{13}C NMR (100 MHz, $CDCl_3$) δ 171.0, 138.2, 134.9, 129.5, 129.2, 128.7, 127.6, 127.5, 127.5, 43.9, 43.6

LRMS (ES+): 226.1 ([M+H]$^+$, 40), 451.2 ([2×M+H]$^+$, 100);

HRMS (ES+): measured 226.1231, ([$C_{15}H_{15}NO$+H]$^+$), requires 226.1232

Data in agreement with the literature.[51]

Procedure was also conducted with 1 mol% on a 5 mmol scale for 24 h, to yield **10** as a white solid (1.10 g, 97%).

***N*-Benzyl-2-(2-nitrophenyl)acetamide (11)**

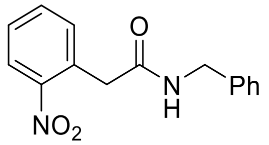

Prepared according to general procedure A from 2-nitrophenylacetic acid (906 mg, 5.0 mmol) and benzylamine (599 µL, 5.5 mmol) for 24 h, and crystallised in reaction flask upon cooling of reaction mixture to yield **11** as a brown solid (1.21 g, 90%).

mp 135-136 °C [Lit.[52] 136-138 °C];

ν_{max} (solid/cm^{-1}) 3292, 3067, 3029, 1641, 1550, 1520

^{1}H NMR (500 MHz, $CDCl_3$) δ 8.04 (dd, *J* = 8.2, 1.1 Hz, 1H, Ar*H*), 7.60 (t, *J* = 7.6 Hz, 1H, Ar*H*), 7.55-7.44 (m, 2H, Ar*H*), 7.33-7.25 (m, 5H, Ar*H*), 6.1 (br s, 1H, N*H*), 4.45 (d, *J* = 5.7 Hz, 2H, C*H*$_2$NH), 3.88 (s, 2H, C*H*$_2$CO);

^{13}C NMR (125 MHz, $CDCl_3$) δ 169.0, 149.0, 138.1, 133.8, 133.7, 130.4, 128.9, 128.7, 127.9, 127.7, 125.3, 44.0, 41.1;

LRMS (ES+): 271.1 ($[M+H]^+$, 100), 541.1 ($[2\times M+H]^+$, 60)

Data in agreement with the literature.[52]

_N_-Benzyl-2-(2-methoxyphenyl)acetamide (12)

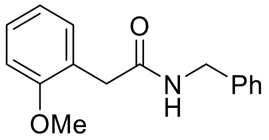

Prepared according to general procedure A from 2-methoxyphenylacetic acid (831 mg, 5.0 mmol) and benzylamine (599 µL, 5.5 mmol) for 24 h, and crystallised in reaction flask upon cooling of reaction mixture to yield **12** as a white needles (1.11 g, 87%).

mp 70-71 °C [Lit.[53] 77-78 °C];

ν_{max} (solid/cm^{-1}) 3255, 3055, 3028, 2831, 1660, 1632, 1595

^{1}H NMR (500 MHz, $CDCl_3$) δ 7.31-7.25 (m, 4H, Ar*H*), 7.19-7.17 (m, 2H, Ar*H*), 6.84 (d, *J* = 7.4 Hz, 1H, Ar*H*), 6.83-6.81 (m, 2H, Ar*H*), 5.8 (br s, 1H, N*H*), 4.41 (d, *J* = 6.0 Hz, 2H, CH_2NH), 3.78 (s, 3H, OCH_3), 3.59 (s, 2H, $COCH_2$);

^{13}C NMR (125 MHz, $CDCl_3$) δ 170.8, 160.1, 138.2, 136.3, 130.2, 128.7, 127.6, 127.5, 121.8, 115.1, 113.0, 55.3, 43.9, 43.6

LRMS (ES+): 256.2 ($[M+H]^+$, 100), 511.3 ($[2\times M+H]^+$, 20)

Data in agreement with the literature.[53]

N-Benzyl-2-(3-fluorophenyl)acetamide (13)

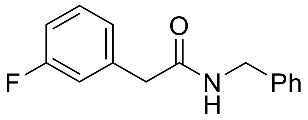

Prepared according to general procedure A from 3-fluorophenylacetic acid (770 mg, 5.0 mmol) and benzylamine (599 µL, 5.5 mmol) for 24 h, and crystallised in reaction flask upon cooling of reaction mixture to yield **13** as a white solid (1.10 g, 91%).

mp 96-97 °C [Lit.[51] 94-96 °C];

ν_{max} (solid/cm^{-1}) 3235, 3061, 3026, 1648, 1623, 1553

^{1}H NMR (500 MHz, $CDCl_3$) δ 7.33-7.24 (m, 4H, Ar*H*), 7.20-7.19 (m, 2H, Ar*H*), 7.05 (d, 1H, *J* = 7.6 Hz, Ar*H*), 7.01-6.96 (m, 2H, Ar*H*), 5.8 (br s, 1H, N*H*), 4.41 (d, *J* = 5.7 Hz, 2H, CH_2NH), 3.59 (s, 2H, COCH_2);

^{13}C NMR (125 MHz, $CDCl_3$) δ 170.2, 163.1 (d, *J* = 247.0 Hz) 138.1, 137.3 (d, *J* = 7.7 Hz), 130.6 (d, *J* = 8.2 Hz), 128.9, 127.7, 127.7, 125.2 (d, *J* = 2.7 Hz), 116.7 (d, *J* = 21.4 Hz), 114.7, (d, *J* = 20.9 Hz), 43.9, 43.5

δ_F (282 MHz, $CDCl_3$) -112.3;

LRMS (ES+): 244.1 ($[M+H]^+$, 100), 245.1 ($[M+H]^+$, 20), 487.2 ($[2\times M+H]^+$, 100)

Data in agreement with the literature.[51]

1-(3,4-Dihydroisoquinolin-2(1H)-yl)-2-phenylethan-1-one (14)

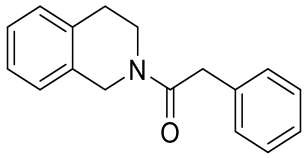

Prepared according to general procedure A from phenylacetic acid (272 mg, 2.0 mmol) and tetrahydroisoquinoline (275 µL, 2.2 mmol) for 24 h, purified using the standard resin workup procedure, to yield **14** as colourless crystals (460 mg, 92%).

Rf: 0.3 (CH_2Cl_2:MeOH, 99:1)

mp 95-96 °C [Lit.[54] 95-97 °C];

ν_{max} (solid/cm^{-1}) 2996, 1634, 1448, 1438;

^{1}H NMR (600 MHz, $CDCl_3$, *mixture of rotamers in a ratio of 10†:6‡*) δ 7.35-7.13‡† (m, 8H, Ar*H*), 7.09† (d, *J* = 6.1 Hz, 0.6H, Ar*H*), 7.01‡ (d, *J* = 6.1 Hz, 0.4H, Ar*H*), 4.79† (s, 1.3H, CH_2Ph), 4.62‡ (s,

0.7H, CH_2Ph), 3.87‡ (t, J = 5.9 Hz, 0.7H, CH_2CH_2), 3.82‡† (s, 2H, CH_2N), 3.67† (t, J = 5.9 Hz, 1.3H, CH_2CH_2), 2.87‡ (t, J = 5.9 Hz, 0.7H, CH_2CH_2), 2.69† (t, J = 5.9 Hz, 1.3H, CH_2CH_2);

^{13}C NMR (150 MHz, $CDCl_3$, *mixture of rotamers in a ratio of 10:6*) δ 170.2, 170.0, 135.2, 135.0, 134.1, 134.1, 133.5, 132.6, 129.0, 128.9, 128.9, 128.8, 128.8, 128.3, 127.0, 127.0, 126.9, 126.8, 126.6, 126.5, 126.1, 47.9, 44.6, 43.9, 41.6, 41.5, 40.1, 29.4, 28.6;

LRMS (ES+): 252.2 ([M+H]$^+$, 100), 503.2 ([2×M+H]$^+$, 10)

HRMS (ES+): Measured 252.1390, ([$C_{17}H_{17}NO$+H]$^+$), requires 252.1388.

Procedure was also conducted in PhMe, on a 5 mmol scale, to yield **14** as colourless crystals (1.14 g, 91%)

Data in agreement with the literature.[54]

1-Morpholino-2-phenylethan-1-one (15)

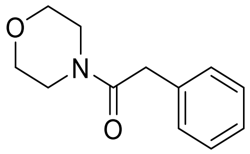

Prepared according to general procedure A from phenylacetic acid (272 mg, 2.0 mmol) and morpholine (190 µL, 2.2 mmol) for 24 h, and purified using the standard resin workup procedure to yield **15** as an off-white solid (410 mg, 89%).

mp 63-64 °C [Lit.[29] 65-67 °C];

ν_{max} (solid/cm^{-1}) 2855, 1636, 1495, 1453, 1427, 1300;

^{1}H NMR (600 MHz, $CDCl_3$) δ 7.33-7.31 (m, 2H, ArH), 7.26-7.23 (m, 3H, ArH), 3.73 (s, 2H, CH_2Ph), 3.64 (s, 4H, OCH_2CH_2N), 3.48-3.42 (m, 4H, OCH_2CH_2N);

^{13}C NMR (150 MHz, $CDCl_3$) δ 169.7, 134.9, 128.9, 128.6, 127.0, 66.9, 66.6, 46.6, 42.2, 41.0;

LRMS (ES+): 206.1 ([M+H]$^+$, 100), 412 ([2×M+H]$^+$, 30)

HRMS (ES+): measured 206.1185, ([$C_{12}H_{15}NO_2$+H]$^+$), requires 206.1181.

Data in agreement with the literature.[29]

***N,N*-Dioctyl-2-phenylacetamide (16)**

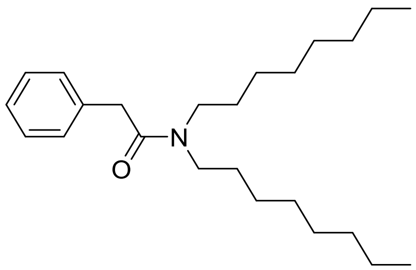

Prepared according to general procedure A from phenylacetic acid (272 mg, 2.0 mmol) and dioctylamine (665 µL, 2.2 mmol) in PhMe for 36 h, and purified using the standard resin workup procedure to yield **17** as a colourless oil (430 mg, 60%).

ν_{max} (film/cm^{-1}) 2954, 2923, 1636, 1454, 1423;

^{1}H NMR (600 MHz, $CDCl_3$) δ 7.31-7.29 (m, 2H, Ar*H*), 7.25-7.21 (m, 3H, Ar*H*), 3.69 (s, 2H, CH_2CON), 3.30 (t, *J* = 7.6 Hz, 2H, NCH_2), 3.18 (t, *J* = 7.9 Hz, 2H, NCH_2), 1.53-1.50 (m, 2H, NCH_2CH_2), 1.50-1.45 (m, 2H, NCH_2CH_2), 1.30-1.22 (m, 20H, 2×CH_2CH_2CH_2CH_2C$H_2$$CH_3$), 0.89 (t, *J* = 7.1 Hz, 3H, C$H_3$$CH_2$), 0.87 (t, *J* = 7.1 Hz, 3H, C$H_3$$CH_2$),

^{13}C NMR (150 MHz, $CDCl_3$) δ 170.5, 135.7, 128.8, 128.7, 126.7, 48.4, 46.0, 41.2, 31.9, 31.9, 29.5, 29.4, 29.4, 29.3, 29.1, 27.7, 27.2, 26.9, 22.8, 22.8, 14.2, 14.2

LRMS (ES+): 360.3 ([M+H]$^+$, 100), 719.6 ([2×M+H]$^+$, 40)

HRMS (ES+): measured 360.3268, ([$C_{24}H_{42}NO$+H]$^+$), requires 360.3266.

***N,N*-Dihexyl-2-phenylacetamide (17)**

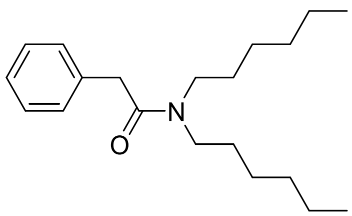

Prepared according to general procedure A from phenylacetic acid (272 mg, 2.0 mmol) and dihexylamine (511 µL, 2.2 mmol) for 40 h in PhMe, purified using the standard resin workup procedure and further purified by column chromatography (CH_2Cl_2:MeOH, 96:4), to yield **16** as a colourless oil (447 mg, 74%).

Rf: 0.6 (CH_2Cl_2:MeOH, 99:1)

ν_{max} (film/cm^{-1}) 2954, 2926, 2857, 1638,1454, 1422;

^{1}H NMR (600 MHz, $CDCl_3$) δ 7.31-7.29 (m, 2H, Ar*H*), 7.25-7.21 (m, 3H, Ar*H*), 3.69 (s, 2H, CH_2CON), 3.30 (t, *J* = 7.6 Hz, 2H, NCH_2), 3.18 (t, *J* = 7.9 Hz, 2H, NCH_2), 1.53-1.48 (m, 2H, NCH_2CH_2), 1.47-1.43 (m, 2H, NCH_2CH_2), 1.30-1.22 (m, 12H, 2 × CH_2CH_2C$H_2$$CH_3$), 0.88 (t, *J* = 7.1 Hz, 3H, C$H_3$$CH_2$), 0.87 (t, *J* = 7.0 Hz, 3H, C$H_3$$CH_2$);

^{13}C NMR (150 MHz, $CDCl_3$) δ 170.6, 135.7, 128.8, 128.7, 126.7, 48.5, 46.0, 41.2, 31.7, 31.6, 29.1, 27.7, 26.8, 26.6, 22.7, 22.7, 14.1, 14.1;

LRMS (ES+): 304.3 ($[M+H]^+$, 100), 629.5 ($[2{\times}M+H]^+$, 20)

HRMS (ES+): measured 304.2637, ($[C_{20}H_{33}NO+H]^+$), requires 304.2635.

Procedure was also conducted in PhMe employing $B(OMe)_3$ to yield **14** as colourless crystals (1.13 g, 75%)

Procedure was also conducted in PhMe employing 5 mol%,$B(OCH_2CF_3)_3$ to yield **14** as colourless crystals (1.10 g, 72%)

***N*-Benzyl-*N*-methyl-2-phenylacetamide (18)**

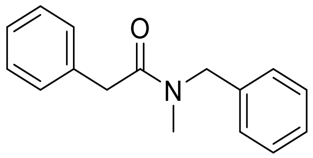

Prepared according to general procedure A from phenylacetic acid (681 mg, 5.0 mmol) and methylbenzylamine (644 µL, 5.0 mmol) for 24 h, and purified using the standard resin workup procedure to yield **18** as a pale yellow oil (1.12 g, 94%).

ν_{max} (film/cm^{-1}) 3061, 3029, 1637, 1583;

^1H NMR (600 MHz, $CDCl_3$, *mixture of rotamers in a ratio of* $3^{\dagger}:2^{\ddagger}$) δ 7.36-7.25‡† (m, 8H, Ar*H*), 7.23† (d, *J* = 7.3 Hz, 1.2H, Ar*H*), 7.10‡ (d, *J* = 7.3 Hz, 0.8H, Ar*H*), 4.63† (s, 1.2H, CH_2), 4.54‡ (s, 0.8H, CH_2), 3.80† (s, 1.2H, CH_2), 3.77‡ (s, 0.8H, CH_2), 2.97‡ (s, 1.3H, CH_3), 2.91† (s, 1.7H, CH_3);

^{13}C NMR (150 MHz, $CDCl_3$, *mixture of rotamers in a ratio of 3:2*) δ 171.3, 171.3, 137.4, 136.6, 135.2, 135.1, 129.1, 129.0, 128.9, 128.9, 128.8, 128.7, 128.2, 127.8, 127.5, 127.0, 126.9, 126.5, 53.8, 51.1, 41.4, 41.0, 35.4, 34.2

LRMS (ES+): 240.2 ($[M+H]^+$, 100), 480.3 ($[2{\times}M+H]^+$, 50)

HRMS (ES+): measured 240.1389, ($[C_{16}H_{18}NO+H]^+$), requires 240.1388.

Data in agreement with literature.[55]

N-Allyl-N,2-diphenylacetamide (19)

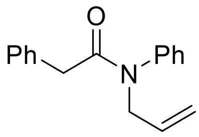

Prepared according to general procedure A from phenylacetic acid (680 mg, 5.0 mmol) and N-allylaniline (678 µL, 5.0 mmol) in PhMe for 48 h, and purified using the standard resin workup procedure to yield **19** as a pale yellow oil (580 mg, 46%).

ν_{max} (film/cm^{-1}) 3303, 3029, 2973, 1691, 1496

^{1}H NMR (600 MHz, $CDCl_3$) δ 7.40-7.35 (m, 3H, ArH), 7.25-7.18 (m, 3H, ArH), 7.08 (d, J = 7.2 Hz, 2H, ArH), 7.05 (d, J = 7.3 Hz, 2H, ArH), 5.86 (ddt J = 17.0, 10.3, 6.3 Hz, 1H, NCH_2CHCH_2), 5.10 (d, J = 10.2 Hz, 1H, NCH_2CHCHH), 5.04 (d, J = 17.0 Hz, 1H, NCH_2CHCHH), 4.30 (d, J = 6.3 Hz, 2H, NCH_2), 3.45 (s, 2H, $PhCH_2$)

^{1}H NMR (150 MHz, $CDCl_3$) δ 170.7, 142.5, 135.5, 133.1, 129.6, 129.2, 128.8, 128.4, 128.2, 126.7, 118.1, 52.6, 41.3

LRMS (ES+): 252.2 ([M+H]$^+$, 30); 503.2 ([2×M+H]$^+$, 100);

HRMS (ES+): measured 252.1390, ([$C_{17}H_{17}NO$+H]$^+$), requires 252.1388

2-Phenyl-1-(3-phenylpiperazin-1-yl)ethan-1-one (20)

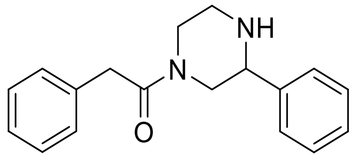

Prepared according to general procedure A from phenylacetic acid (272 mg, 2.0 mmol) and 2-phenylpiperazine (356 mg, 2.2 mmol) in TAME (more dilute due to solubility issues, at 0.25 M) for 24 h. Product was purified by column chromatography, to yield **20** as a colourless oil (465 mg, 79%);

ν_{max} (film/cm^{-1}) 3300, 1631, 1602, 1494, 1450;

^{1}H NMR (600 MHz, $CDCl_3$, *mixture of rotamers in a ratio of 0.57†:0.43‡*) δ 7.44- 7.21‡† (m, 9H), 7.15-7.12‡† (m, 1H), 4.68-4.65‡† (m, 1H), 3.81- 3.75‡† (m, 3H), 3.25† (dd, J = 10.5, 2.8 Hz, 0.57H), 3.17‡ (td, J = 12.8, 3.1 Hz, 0.43H), 3.11-3.08† (m, 0.57H), 3.02-3.00‡ (m, 0.43H, NHCHH), 2.96† (dd, J = 12.9, 10.6 Hz, 0.57H), 2.84-2.75† (m, 1.14H), 2.66-2.58‡ (m, 0.86H), 1.2‡† (br s, 1H, NH);

^{13}C NMR (150 MHz, $CDCl_3$) δ 169.6, 169.5, 141.3, 140.9, 135.4, 135.3, 129.0, 128.9, 128.8, 128.8, 128.7, 128.7, 128.1, 128.0, 127.1, 127.0, 127.0, 126.9, 60.9, 60.1, 53.9, 49.1, 46.6, 46.4, 46.0, 42.1, 41.5, 41.3;

LRMS (ES+): 281.2 ($[M]^+$, 100).

HRMS (ES+): measured 281.1652, ($[C_{18}H_{20}N_2O+H]^+$), requires 281.1654

Data in agreement with literature.[51]

3-Hydroxy-1-(3-(trifluoromethyl)-5,6-dihydro-[1,2,4]triazolo[4,3-a]pyrazin-7(8H)-yl)butan-1-one (21)

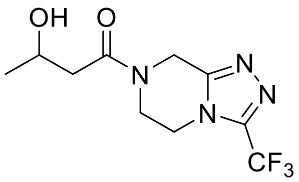

Prepared according to general procedure A, from 3-Hydroxybutyric acid (208 mg, 2 mmol, highly hygroscopic solid – turns into an oil over time) and 3-(trifluoromethyl)-5,6,7,8-tetrahydro-[1,2,4]triazolo[4,3-a]pyrazine (422 mg, 2.2 mmol, see note on next page about amine purity). Product was purified by column chromatography (CH_2Cl_2:MeOH, 95:5) to yield **21** as a colourless oil (339 mg, 63%).

Rf: 0.3 (CH_2Cl_2:MeOH, 95:5)

ν_{max} (film/cm^{-1}) 3321, 2977, 2936, 2929, 2894, 1714, 1615, 1504

^{1}H NMR (600 MHz, MeOD, mixture of rotamers in a ratio of 2‡:3†) δ 5.13‡ (d, J = 17.0 Hz, 0.4H, C*H*H), 5.07† (d, J = 17.5 Hz, 0.6H, C*H*H), 5.03‡ (d, J = 17.0 Hz, 0.4H, C*H*H), 4.95† (d, J = 17.5 Hz, 0.6H, C*H*H), 4.63 (br s, 0.5H, O*H*), 4.31† (t, J = 5.4 Hz, 1.2H, C*H*HCH$_2$), 4.23-4.04‡† (m, 3.8H), 2.74-2.68‡† (m, 1H), 2.57† (d, J = 15.1 Hz, 0.6H), 2.56‡ (d, J = 15.1 Hz, 0.4H) ,1.26-1.24‡† (m, 3H, C*H*$_3$)

^{13}C NMR (600 MHz, MeOD, mixture of rotamers in a ratio 2‡:3†) δ 173.3‡, 173.2†, 152.8†, 152.5‡, 114.7‡† (q, J = 40.0 Hz), 119.2‡† (q, J = 270.0 Hz), 66.1‡, 66.0†, 45.3†, 44.6‡, 43.7‡, 43.0†, 43.0‡, 42.8†, 39.8†, 39.1‡, 2 × 25.5‡†

LRMS (ES+): 279.1 ($[M]^+$, 100), 301.1 ($[M+Na]^+$, 30)

HRMS: (ES+): measured 279.1068 ($[C_{10}H_{13}F_3N_4O_2+H]^+$), requires 279.1063

Note on purity of amine: 3-(trifluoromethyl)-5,6,7,8-tetrahydro-[1,2,4]triazolo[4,3-a]pyrazine is described as a colourless oil or yellow oil in literature. The purchased material was a dark brown solid, and resulted in the formation of a dense layer of insoluble oil at the bottom of the reaction flask. Liquid-liquid extraction with EtOAc/$NaHCO_3$ resulted in a pale yellow oil as described in

literature and successful amidation reaction. Alternatively, the commercially available HCl salt of the amine can also be washed with EtOAc/$NaHCO_3$ to give free amine as a colourless oil.

_N_-Benzyl-2-(thiophen-2-yl)acetamide (22)

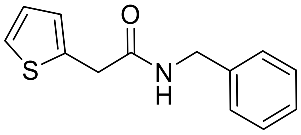

Prepared according to general procedure A from 2-Thiopheneacetic acid (284 mg, 2.0 mmol) and benzylamine (240 µL, 2.2 mmol) for 24 h, and purified using the standard resin workup procedure to yield **22** as light brown solid (420 mg, 92%).

mp 104-105 °C;

ν_{max} (solid/cm^{-1}) 3264, 3074, 3056, 3024, 2961, 2920, 1645, 1551

^{1}H NMR (600 MHz, $CDCl_3$) δ 7.32-7.29 (m, 2H, Ar*H*), 7.27-7.24 (t, *J* = 7.3 Hz, 1H, Ar*H*), 7.23-7.20 (m, 3H, Ar*H*), 6.97 (dd, *J* = 5.2, 3.3 Hz, 1H, Ar*H*), 6.93 (d, *J* = 2.8 Hz, 1H, Ar*H*), 6.3 (br s, 1H, N*H*), 4.40 (d, *J* = 5.8 Hz, 2H, CH_2N), 3.79 (s, 2H, CH_2O)

^{13}C NMR (150 MHz, $CDCl_3$) δ 170.0, 138.2, 136.3, 128.8, 127.7, 127.6, 127.5, 127.5, 125.7, 43.7, 37.6

LRMS (ES+): 232.1 ($[M]^+$, 100), 463.2 ($[2\times M]^+$, 20)

HRMS (ES+): measured 232.0795, ($[C_{13}H_{13}NO_2S+H]^+$), requires 232.0796

Data in agreement with literature.[18]

_N_-Benzylpicolinamide (23)

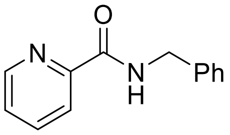

Prepared according to general procedure A from picolinic acid (620 mg, 5.0 mmol) and benzylamine (610 µL, 5.5 mmol) for 24 h and purified by column chromatography, to yield **23** as colourless crystals (900 mg, 85%).

Rf: 0.5 (CH_2Cl_2:MeOH, 99:1)

mp 82-83 °C [Lit.[51] 85-87 °C];

ν_{max} (solid/cm^{-1}) 3297, 3080, 2923, 1654, 1584

^{1}H NMR (600 MHz, $CDCl_3$) δ 8.50 (d, *J* = 4.7 Hz, 1H, Ar*H*), 8.4 (br s, 1H, N*H*), 8.23 (d, *J* = 7.8 Hz, 1H, Ar*H*), 7.81 (td, *J* = 7.7, 1.7 Hz, 1 H, Ar*H*), 7.38 (ddd, *J* = 7.6, 4.8, 1.1 Hz, 1H, Ar*H*), 7.36-7.31 (m, 4H, Ar*H*), 7.26 (t, *J* = 7.2 Hz, 1H, Ar*H*), 4.66 (d, *J* = 6.1 Hz, 2H, CH_2N);

^{13}C NMR (150 MHz, $CDCl_3$) δ 164.4, 150.0, 148.2, 138.4, 137.5, 128.8, 127.9, 127.6, 126.3, 122.5, 43.6

LRMS 213 ([M]$^+$, 100), 425 ([2×M]$^+$, 50)

HRMS: (ES+): measured 213.1027 ([$C_{13}H_{12}N_2O$+H]$^+$), requires 213.1028.

Data in agreement with literature. [51]

2-Phenyl-_N_-(pyridin-2-yl)acetamide (24)

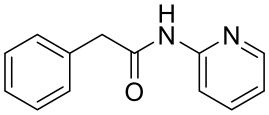

Prepared according to general procedure A, from phenylacetic acid (272 mg, 2 mmol) and excess pyridin-2-amine (282 mg, 3.0 mmol) for 40 h. Product was purified without use of resins Amberlyst A15 during the resin workup and further purified by column chromatography to yield **24** as a white solid (260 mg, 63%).

Rf: 0.6 (CH_2Cl_2:MeOH, 99:1)

mp 122-123 °C [lit.[29] 121-122°C];

ν_{max} (film/cm^{-1}) 3234 (broad), 3043, 1656, 1535;

^{1}H NMR (600 MHz, $CDCl_3$) δ 8.30 (br s, 1H, N*H*), 8.24 (d, *J* = 8.4 Hz, 1H, Ar*H*), 8.22 (d, *J* = 4.9 Hz, 1H, Ar*H*), 7.69 (dd *J* = 7.0, 1.9 Hz, 1H, Ar*H*), 7.39-7.36 (m, 2H, Ar*H*), 7.32-7.30 (m, 3H, Ar*H*), 7.02 (dd, *J* = 7.3, 4.9 Hz, 1H, Ar*H*), 3.75 (s, 2H, CH_2);

^{13}C NMR (150 MHz, $CDCl_3$) δ 169.7, 151.4, 147.8, 138.6, 134.1, 129.6, 129.3, 127.6, 120.1, 114.2, 45.0;

LRMS (ES+): 213.1 ($[M]^+$, 100);

HRMS: (ES+): 213.1025 ($[C_{13}H_{12}N_2O+H]^+$), requires 213.1028.

Data in agreement with literature.[29]

***N*-Mesityl-2-phenylacetamide (25)**

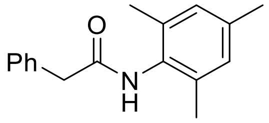

Prepared according to general procedure A from phenylacetic acid (680 mg, 5.0 mmol) and 2,4,6 trimethylaniline (700 µL, 5.0 mmol) in PhMe for 15 h, and purified using the standard resin workup procedure to yield **25** as a fluffy white solid (930 mg, 74%).

mp 165-166 °C [Lit.[56] 152-153 °C];

ν_{max} (solid/cm^{-1}) 3250, 3029, 2915, 2854, 1654, 1517

^{1}H NMR (600 MHz, DMSO-d_6, *[note: rotamers observed in CDCl$_3$]*) δ 9.39 (br s, 1H, N*H*), 7.42-7.25 (m, 5H, Ar*H*), 6.85 (s, 2H, Ar*H*), 3.65 (s, 2H, CH_2), 2.22 (s, 3H, *p*-CH_3), 2.06 (s, 6H, 2×*o*-CH_3)

^{13}C NMR (150 MHz, DMSO-d_6) δ 168.9, 136.5, 136.4, 134.9, 132.5, 129.1, 128.3, 128.3, 126.5, 42.6, 20.5, 18.0

LRMS (ES+): 254.2 ($[M+H]^+$, 100), 507.3 ($[2×M+H]^+$, 50)

HRMS (ES+): measured 254.1549, ($[C_{17}H_{19}NO+H]^+$), requires 254.1545

Data in agreement with literature. [56]

Procedure was also conducted with $B(OMe)_3$ in PhMe for 15 h, on a 5 mmol scale, to yield **25** as colourless crystals (923 g, 73%)

***N*-Benzylpivalamide (26)**

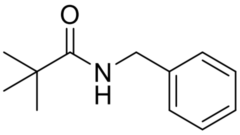

Prepared according to general procedure A from pivalic acid (204 mg, 2.0 mmol) and benzylamine (240 µL, 2.2 mmol) for 24 h and purified using the standard resin workup procedure to yield **26** as white crystals (273 mg, 71%);

mp 86-87 °C [Lit.[28] 80-81 °C];

ν_{max} (solid/cm^{-1}) 3298, 2967, 1633, 1539, 1493;

^{1}H NMR (600 MHz, $CDCl_3$) δ 7.35-7.25 (m, 5H, Ar*H*), 5.9 (br s, 1H, N*H*), 4.44 (d, *J* = 5.5 Hz, 2H, CH_2), 1.23 (s, 9H, 3 × CH_3);

^{13}C NMR (150 MHz, $CDCl_3$) δ 178.4, 138.7, 128.8, 127.7, 127.5, 43.6, 38.8, 27.7

LRMS (ES+): 192.1 ([M+H]$^+$, 100), 214.1 ([M+Na+H]$^+$, 10), 405.3 ([2×M+Na+H]$^+$, 20),

HRMS (ES+): measured 192.1384 ([$C_{12}H_{17}NO$+H]$^+$), requires 192.1383.

Data in agreement with literature.[28]

***N*-Benzylbut-2-ynamide (27)**

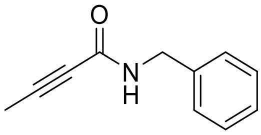

Prepared according to general procedure A from 2-butynoic acid (168 mg, 2.0 mmol) and benzylamine (240 µL, 2.2 mmol) for 18 h, and purified using the standard resin workup procedure to yield **27** as colourless crystals (300 mg, 87%);

mp 127-128 °C; [Lit.[28] 114-115 °C];

ν_{max} (solid/cm^{-1}) 3209, 3027, 2252, 2216, 1615, 1538, 1493;

^{1}H NMR (600 MHz, $CDCl_3$, *mixture of rotamers in a ratio of 10†:1‡*) δ 7.37-7.31‡† (m, 2H, ArH), 7.31-7.26‡† (m, 3H, ArH), 6.02† (br s, 0.9H, CON*H*), 5.95‡ (br s, 0.1H, CON*H*), 4.60‡ (d, *J* = 6.6 Hz, 0.2H, CH_2Ph) 4.47† (d, *J* = 5.8 Hz, 1.8H, CH_2Ph), 2.00‡ (s, 0.3H, CH_3), 1.93† (s, 2.7H, CH_3);

^{13}C NMR (150 MHz, $CDCl_3$) δ 153.4, 137.5, 128.9, 128.8, 127.9, 127.8, 127.2, 83.8, 74.8, 43.9, 3.7;

LRMS (ES+): 174.1 (100 [M+H]$^+$), 196.1 (10, [M+Na]$^+$)

Data in agreement with literature.[28]

N-(2-(1H-Indol-3-yl)ethyl)-2-phenylacetamide (28)

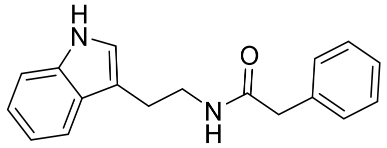

Prepared according to general procedure A (5 mol% $B(OCH_3CF_3)_3$) from phenylacetic acid (681 mg, 5.0 mmol) and tryptamine (801 mg, 5.0 mmol) in PhMe for 24 h. Product was purified by column chromatography, to yield **28** as a brown solid (1.156 g, 83%);

mp 148-149 °C [Lit.[29] 145-146 °C];

ν_{max} (solid/cm^{-1}) 3391, 3263, 1635, 1550 ;

^{1}H NMR (600 MHz, $CDCl_3$) δ 8.07 (br s, 1H, ArN*H)*, 7.54 (d, *J* = 8.2 Hz, 1H, Ar*H*), 7.35 (d, *J* = 8.1 Hz, 1H, Ar*H*), 7.31-7.26 (m, 3H, Ar*H*), 7.21 (td, *J* = 8.0, 1.0 Hz, 1H, Ar*H*), 7.16-7.14 (m, 2H, ArH), 7.11 (t, *J* = 7.0 Hz, 1H, ArH), 6.77 (d, *J* = 2.3 Hz, 1H, ArH), 5.47 (br s, 1H, CON*H*), 3.54 (dt, *J* = 6.5, 6.4 Hz, 2H, CH_2CH_2), 3.52 (s, 2H, CH_2Ph), 2.90 (t, *J* = 6.5 Hz, 2H, CH_2CH_2);

^{13}C NMR (125 MHz, $CDCl_3$) δ 171.1, 136.4, 135.1, 129.6, 129.1, 127.3, 127.3, 122.3, 122.1, 119.6, 118.8, 112.8, 111.3, 44.1, 39.9, 25.2

LRMS (ES+): 279.1 ($[M+H]^+$, 60), 557.2 ($[2\times M+H]^+$, 100)

HRMS (ES+): measured 279.1494, ($[C_{18}H_{18}ON_2+H]^+$), requires 279.1497.

Data in agreement with literature.[29]

N-Benzyl-2-(1H-indol-3-yl)acetamide (29)

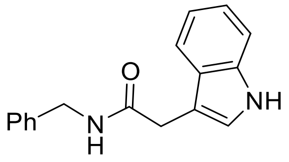

Prepared according to general procedure A, from 3-indole acetic acid (350 mg, 2.0 mmol) and benzylamine (240 µL, 2.2 mmol) for 24 h. Product was purified by crystallisation with hot dioxane/water (1:1) to give sharp off-white crystals (451 mg, 85%)

Rf: 0.2 (CH_2Cl_2:MeOH, 99:1)

mp 155-156 °C; [Lit.[57] 156-158 °C];

ν_{max} (solid/cm^{-1}) 3268, 2925, 1627, 1616, 1555, 1493

^{1}H NMR (600 MHz, DMSO-d_6) δ 10.9 (br s, 1H, ArN*H*), 8.4 (br t, *J* = 5.7 Hz, 1H, CON*H*), 7.55 (d, *J* = 7.9 Hz, 1H, Ar*H*), 7.35 (d, *J* = 8.1 Hz, 1H, Ar*H*), 7.29-7.27 (m, 2H, Ar*H*), 7.23-7.20 (m, 4H, Ar*H*), 7.07 (td, *J* = 7.1, 0.8 Hz, 1H, Ar*H*), 6.97 (td, *J* = 7.0, 0.8 Hz, 1H, Ar*H*), 4.27 (d, *J* = 5.9 Hz, 2H, CH_2N), 3.58 (s, 2H, CH_2CO);

^{13}C NMR (150 MHz, DMSO-d_6) δ 170.8, 139.7, 136.2, 128.2, 127.3, 127.2, 126.7, 123.9, 121.0, 118.8, 118.3, 111.3, 108.9, 42.2, 32.7;

LRMS (ES+): 265.1 ($[M]^+$, 100), 529.3 ($[2\times M]^+$, 40);

HRMS (ES+): measured 265.1345, ($[C_{17}H_{16}N_2O+H]^+$), requires 265.1341

Data in agreement with literature.[57]

2-Phenyl-*N*-tosylacetamide (30)

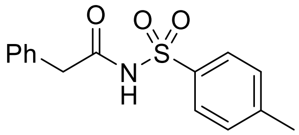

Prepared according to general procedure A from phenylacetic acid (680 mg, 5.0 mmol) and *p*-toluenesulfonamide (855 mg, 5.0 mmol) in PhMe for 24 h. The product was purified by column chromatography followed by recrystallisation from hot CPME, to yield **30** as a white solid (450 mg, 31%).

mp 151-152 °C; [Lit.[56] 148-149 °C];

ν_{max} (solid/cm^{-1}) 3089, 3061, 2879, 1677, 1596, 1455, 1341

^{1}H NMR (600 MHz, $CDCl_3$) δ 8.25 (br s, 1H, N*H*), 7.88 (d, *J* = 8.4 Hz, 2H, Ar*H*), 7.33-7.31 (m, 5H, Ar*H*), 7.16-7.14 (m, 2H, Ar*H*), 3.59 (s, 2H, CH_2), 2.45 (s, 3H, CH_3);

^{13}C NMR (600 MHz, $CDCl_3$) δ 168.6, 145.4, 135.3, 132.3, 129.7, 129.5, 129.4, 128.6, 128.2, 43.8, 21.9

LRMS (ES+): 290.1 ($[M+H]^+$, 100), 579.1 ($[2\times M+H]^+$, 50)

HRMS (ES+): measured 290.0855, ($[C_{15}H_{15}NO_3S+H]^+$), requires 290.0851

Data in agreement with literature.[56]

***(S)-N*-Benzyl-2-(4-isobutylphenyl)propanamide (31)**

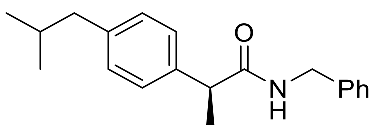

Prepared according to general procedure A, from (S)-Ibuprofen (412 mg, 2.0 mmol) and benzylamine (240 µL, 2.2 mmol) for 6 h and purified using the standard resin workup procedure to yield **31** as a white solid (533 mg, 90%).

mp 76-77 °C [Lit.[29] 77-78 °C];

$[\alpha]_D^{20}$ +7.4 (*c* 0.88, CH_2Cl_2) [Lit.[29] $[\alpha]_D^{20}$ +7.2 (*c* 0.88, CH_2Cl_2)];

ν_{max} (solid/cm^{-1}) 3304, 2949, 2866, 1638;

^{1}H NMR (600 MHz, $CDCl_3$) δ 7.29−7.19 (m, 5H, Ar*H*), 7.14−7.11 (m, 4H, Ar*H*), 6.3 (br s, 1H, N*H*), 4.35 (d, *J* = 5.8 Hz, 2H, CH_2Ph), 3.58 (q, *J* = 7.2 Hz, 1H, C*H*CON), 2.45 (d, *J* = 7.1 Hz, 2H, CH_2), 1.85 (app nonet, *J* = 6.8 Hz, 1H, C*H*(CH_3)$_2$), 1.55 (d, *J* = 7.2 Hz, 3H, CH_3), 0.90 (d, *J* = 6.6 Hz, 6H, (CH_3)$_2$)

^{13}C NMR (150 MHz, CDCl3) δ 174.7, 140.7, 138.8, 138.7, 129.7, 128.6, 127.5, 127.4, 127.3, 46.7, 45.1, 43.5, 30.3, 22.5, 18.6

LRMS (ES+) 296.2 ([M+H]$^+$, 100)

Commercially available ibuprofen was used, from Alfa Aesar. The enantiopurity for the starting material was measured by HPLC to be 99.2:0.8 er. The corresponding amide, had an enantiopurity of 98.5:1.5, implying less than 1% racemisation [previous literature of the synthesis of this compound suggests that is it prone to racemisation: Compound was reported to have an ee of 83% in H. Lundberg, F. Tinnis, H. Adolfsson, *Synlett* **23**, 2201-2204 (2012); and one of >95% in N. Gernigon, R. M. Al-Zoubi, D. G. Hall, *J. Org. Chem.* ***77,*** 8386-8400 (2012).]: Chiral HPLC ADH column, 90:10 hexane:^{i}PrOH, flow 0.8 mL/min, λ = 220 nm, 20 °C, t_R= 12.6 min (S enantiomer), and 21.0 (R enantiomer).

Data in agreement with literature.[29]

***N*-Benzyl-5-((3aS,4S,6aR)-2-oxohexahydro-1H-thieno[3,4-d]imidazol-4-yl)pentanamide (32)**

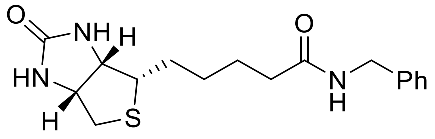

Prepared according to general procedure A using 20 mol% $B(OCH_2CF_3)_3$, from Biotin (1.22 g, 5.0 mmol) and benzylamine (546 µL, 5.0 mmol) for 24 h and purified by column chromatography (CH_2Cl_2:MeOH, 92:8) to yield **32** as a white solid (1.11 g, 66%).

Rf: 0.1 (CH_2Cl_2:MeOH, 99:1)

mp 171-172 °C

$[\alpha]_D^{20}$+78.8 (*c* 0.25, MeOH);

ν_{max} (solid/cm^{-1}) 3656, 3420, 3273, 2967, 2939, 2867, 1689, 1671, 1640, 1543

^{1}H NMR (600 MHz, DMSO-d_6) δ 8.29 (br t, *J* = 6.0 Hz, 1H, N*H*), 7.32-7.30 (m, 2H, Ar*H*), 7.24-7.21 (m, 3H, Ar*H*), 6.42 (br s, 1H, N*H*), 6.35 (br s, 1H, N*H*), 4.31-4.29 (m, 1H, C*H*NHCONH), 4.25 (d, *J* = 5.9 Hz, 2H, CH_2Ph), 4.13-4.11 (m, 1H, C*H*CHS), 3.11-3.08 (m, 1H, C*H*S), 2.83 (dd, *J* = 12.4, 5.1 Hz, 1H, C*H*HS), 2.58 (d, *J* = 12.4 Hz, 1H, C*H*HS), 2.14 (t, *J* = 7.4 Hz, 2H, CH_2CONHBn), 1.65-1.44 (m, 4H, 2×CH_2), 1.36-1.27 (m, 2H, CH_2)

^{13}C NMR (150 MHz, DMSO-d_6) δ 172.1, 162.7, 139.8, 128.3, 127.2, 126.7, 61.1, 59.2, 55.5, 42.0, 40.1, 35.2, 28.3, 28.1, 25.4

LRMS (ES+): 334.2 ([M+H]$^+$, 100])

HRMS (ES+): measured 334.1581, ([$C_{17}H_{23}N_3O_2S$+H]$^+$), requires 334.1589

(4R)-N-Benzyl-4-((3R,8R,9S,10S,13R,14S,17R)-3-hydroxy-10,13-dimethylhexadecahydro-1H-cyclopenta[a]phenanthren-17-yl)pentanamide (33)

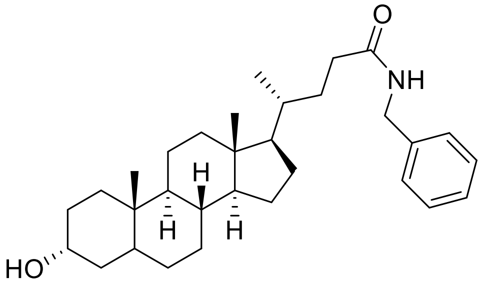

Prepared according to general procedure A using 20 mol% $B(OCH_2CF_3)_3$, from lithocholic acid (1.88 g, 5.0 mmol) and benzylamine (600 µL, 5.5 mmol) for 24 h and purified by column chromatography (CH_2Cl_2:MeOH, 95:5) to yield **33** as a white solid (1.59 g, 68%).

Rf: 0.3 (CH_2Cl_2:MeOH, 99:1)

mp 195-196 °C

$[\alpha]_D^{20}$+26.9 (*c* 1.0, MeOH);

ν_{max} (solid/cm^{-1}) 3670, 3405, 3329, 2971, 2922, 2857, 1649, 1537

^{1}H NMR (600 MHz, DMSO-d_6) δ 8.3 (br s, 1H, N*H*), 7.31-7.29 (m, 2H, Ar*H*), 7.27-7.21 (m, 3H, Ar*H*), 4.43 (d, *J* = 4.6 Hz, 1H), 4.24 (dd, *J* = 5.9, 2.1 Hz, 2H), 3.38-3.33 (m, 1H), 2.18-2.12 (m, 1H), 2.07-2.02 (m, 1H), 1.92-1.90 (m, 1H), 1.81-1.75 (m, 2H), 1.70-1.68 (m, 2H), 1.68 (q, *J* = 12.2 Hz, 1H), 1.51-1.47(m, 2H), 1.35-1.30 (m, 7H), 1.20-1.00 (m, 11H), 0.92-0.88 (m, 1H), 0.88 (d, *J* = 6.6 Hz, 3H), 0.86 (s, 3H), 0.59 (s, 3H)

^{13}C NMR (150 MHz, DMSO-d_6) δ 173.5, 138.6, 128.8, 128.0,127.6, 72.0, 56.6, 56.1, 43.7, 42.9, 42.2, 40.5, 40.3, 36.6, 35.9, 35.6, 35.5, 34.7, 33.8, 31.9, 30.7, 28.4, 27.3, 26.5, 24.3, 23.5, 20.9, 18.5, 12.2

LRMS (ES+): 466.4 ([M+H]$^+$, 100])

HRMS (ES+): measured 466.3681, ([$C_{31}H_{47}NO_2$+H]$^+$), requires 466.3685

tert-Butyl (2-oxo-2-((2-oxoazepan-3-yl)amino)ethyl)carbamate (34)

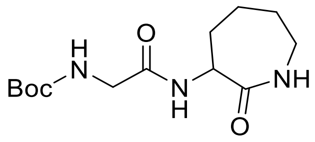

Prepared according to general procedure A with 20 mol% B(OCH_2CF_3)$_3$ from Boc-glycine (875 mg, 5.0 mmol) and DL-α-Amino-ε-caprolactam (902 mg, 5.5 mmol) for 5 h, and crystallised from hot (propanol:H_2O, 2:1) to yield **34** as pale yellow crystals (1.14 g, 80%)

mp 154-155 °C

ν_{max} (solid/cm^{-1}) 3349, 3296, 2972, 2924, 1700, 1645, 1525

^{1}H NMR (600 MHz, $CDCl_3$) δ 8.01 (br t, *J* = 6.0 Hz, 1H, N*H*), 6.20 (br t, *J* = 5.2 Hz, 1H, BocN*H*), 3.90 (d, *J* = 11.1 Hz, 1H, NHC*H*), 3.32 (d, *J* = 5.2 Hz, 2H, NHCH_2CON), 3.15-3.03 (m, 2H, NHCH_2CH$_2$), 1.87 (app d, *J* = 10.3 Hz, 2H, CHCH$_2$CH_2) 1.74-1.71 (m, 1H, 1×NHCH$_2$*CH*H), 1.63-1.57 (m, 1H, 1×CHC*H*H), 1.46-1.40 (m, 1H, 1×CHC*H*H), 1.36 (s, 9H, (CH_3)$_3$), 1.22-1.16 (m, 1H, 1×NHCH$_2$*CH*H)

^{13}C NMR (125 MHz, $CDCl_3$) δ 173.9, 172.1, 155.3, 77.4, 52.0, 43.9, 40.4, 30.4, 28.7, 28.3, 27.3

LRMS (ES+): 286.2 ([M+H]$^+$, 10]), 304.2 ([M+Na]$^+$, 25])

HRMS: (ES+): measured 308.1585, ([$C_{13}H_{23}N_3O_4$+Na]$^+$), requires 308.1581

tert-Butyl ((2S,3R)-1-((1H-pyrazol-3-yl)amino)-3-hydroxy-1-oxobutan-2-yl)carbamate (35)

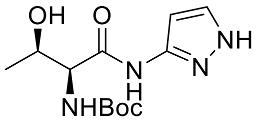

Prepared according to general procedure A with 20 mol% $B(OCH_2CF_3)_3$ from Boc-threonine (438 mg, 2.0 mmol) and 1H-pyrazol-3-amine (183 mg, 2.2 mmol) for 24 h, and purified by column chromatography (CH_2Cl_2:MeOH, 95:5), to yield **35** as a white solid (530 mg, 93%)

$[\alpha]_D^{20}$ -80.8 (*c* 1.0, $CHCl_3$);

mp 157-158 °C

ν_{max} (solid/cm^{-1}) 3290, 3249, 2979, 1685, 1666, 1601, 1493

^{1}H NMR (500 MHz, $CDCl_3$) δ 11.9 (br s, 2H, CON*H*, ArN*H*), 7.54 (d, *J* = 2.3 Hz, 1H, Ar*H*), 6.8 (br d, *J* = 1.5 Hz, 1H, Ar*H*), 5.74 (d, *J* = 10.0 Hz, 1H, N*H*), 4.26-4.24 (m, 2H, 2×C*H*), 4.1 (br s, 1H, O*H*), 1.48 (s, 9H, $(CH_3)_3$), 1.18 (d, *J* = 6.2 Hz, 3H, CH_3)

^{13}C NMR (125 MHz, $CDCl_3$) δ 170.1, 157.1, 147.2, 129.4, 97.4, 81.3, 67.8, 57.2, 28.4, 18.6

LRMS (ES+): 285.2 ($[M]^+$, 50), 569.3 ($[2\times M]^+$, 100),

HRMS: (ES+): measured 285.1563, ($[C_{12}H_{20}N_4O_4+H]^+$), requires 285.1565

tert-Butyl (2-(benzylamino)-2-oxoethyl)carbamate (36)

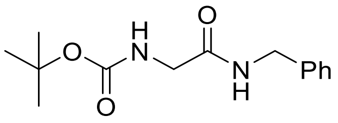

[*Scale up procedure*] Prepared according to general procedure A from Boc-glycine (17.5 g, 100 mmol), benzylamine (12.0 mL, 110 mmol), and $B(OCH_2CF_3)_3$ (3.08 g, 10 mmol) in TAME (200 mL), for 24 h. Upon completion, the reaction mixture was concentrated to half its volume by collecting TAME from the Dean-Stark side arm trap (100 mL recovered). The rest of the solvent was removed *in vacuo*, product was dissolved in DMC (100 mL) and H_2O (1 mL) and resins IRA743 (2 g), A15 (2 g) and A26 OH (1.5 g) were added. The reaction mixture was stirred for 30 min, at which point $MgSO_4$ (1 g) was added. The mixture was filtered, and reaction mixture washed twice with DMC (25 mL). Solution was concentrated *in* vacuo to yield **36** as a white solid (25.8 g, 97%).

mp 75-76 °C;

ν_{max} (solid/cm^{-1}) 3337, 3313, 3064, 2985, 1702, 1655, 1512

^{1}H NMR (500 MHz, $CDCl_3$) δ 7.29-7.26 (m, 2H, Ar*H*), 7.24-7.21 (m, 3H, Ar*H*), 6.9 (br s, 1H, N*H*), 5.5 (br t, *J* = 4.9 Hz, 1H, N*H*), 4.38 (d, *J* = 5.7 Hz, 2H, PhCH_2), 3.78 (s, 2H, BocNHCH_2), 1.39 (s, 9H, (CH_3)$_3$)

^{13}C NMR (125 MHz, $CDCl_3$) δ 169.5, 156.3, 138.0, 128.8, 127.8, 127.7, 80.4, 44.6, 43.5, 28.4

LRMS (ES+): 265.1 ($[M]^+$, 100), 165.1 ($[M\text{-}Boc]^+$, 60);

HRMS (ES+): measured 265.15470, ($[C_{14}H_{20}N_2O_3+H]^+$), requires 265.15467.

Data in agreement with literature.[14]

tert-Butyl (_S_)-(1-(benzylamino)-1-oxopropan-2-yl)carbamate (37)

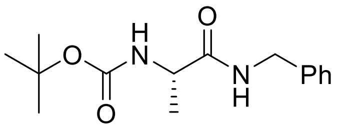

Prepared according to general procedure A from Boc-alanine (945 mg, 5.0 mmol) and benzylamine (546 µL, 5.0 mmol) for 24 h, and purified using the standard resin workup procedure to yield **37** as a white solid (1.33 g, 95%)

mp 105-106 °C; [Lit.[28] 100-102 °C];

$[\alpha]_D^{20}$-21.8 (*c* 1.0, $CHCl_3$); [Lit.[28] $[\alpha]_D^{20}$ –23.9 (*c* 1.9, $CHCl_3$)];

ν_{max} (solid/cm^{-1}) 3378, 2982, 1687, 1651, 1515

^{1}H NMR (500 MHz, $CDCl_3$) δ 7.31-7.29 (m, 2H, Ar*H*), 7.26-7.23 (m, 3H, Ar*H*), 6.7 (br s, 1H, N*H*), 5.1 (br s, 1H, N*H*), 4.42 (br s, 2H, PhCH_2), 4.21 (br s, 4.21, 1H, C*H*CH_3), 1.39 (s, 9H, (CH_3)$_3$), 1.36 (d, *J* = 7.1 Hz, 3H, CH_3)

^{13}C NMR (125 MHz, $CDCl_3$) δ 172.6, 155.7, 138.2, 128.7, 127.6, 127.5, 80.2, 50.2, 43.4, 28.4, 18.4

LRMS (ES+): 279.2 ($[M+H]^+$, 30), 557.3 ($[2\times M+H]^+$, 100)

HRMS (ES+): measured 279.1715, ($[C_{15}H_{22}N_2O_3+H]^+$), requires 279.1709

Enantiopurity was determined by chiral HPLC to be >99:1 er : Chiral HPLC OD1 column, 90:10 hexane:^{i}PrOH, flow 0.8 mL/min, λ = 254 nm, 20 °C, t_R= 9.8 min (S enantiomer), and 11.6 (R enantiomer).

Compound was also prepared according to general procedure A using $B(OMe)_3$ as the catalyst from, Boc-alanine (0.945 g, 5.0 mmol) and benzylamine (0.546 mL, 5.0 mmol) in both TAME and PhMe for 24 h, and purified using the standard resin workup procedure to yield **37** as a white solid (0.64 g, 46% in TAME and 85% in PhMe). Spectroscopic data and chiral purity was identical.

Data in agreement with literature.[28]

tert-Butyl ((2S,3R)-1-(benzylamino)-3-hydroxy-1-oxobutan-2-yl)carbamate (38)

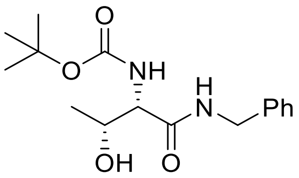

Prepared according to general procedure A with 20 mol% $B(OCH_2CF_3)_3$ from Boc-threonine (1.095 g, 5.0 mmol) and benzylamine (600 µL, 5.5 mmol) for 24 h, and purified using the standard resin workup procedure to yield **38** as a white solid (1.46 g, 95%)

mp 91-92 °C

$[\alpha]_D^{20}$-45.2 (*c* 1.0, $CHCl_3$);

ν_{max} (solid/cm^{-1}) 3332, 2952, 1680

^{1}H NMR (500 MHz, DMSO-d_6, 60 °C) δ 8.10 (br s, 1H, N*H*), 7.29-7.26 (m, 2H, Ar*H*), 7.24-7.21 (m, 3H, Ar*H*), 6.2 (br s, 1H, N*H*), 4.65 (d, *J* = 6.0 Hz, 1H, C*H*NH), 4.34 (dd, *J* = 15.3, 5.9 Hz, 1H, CH*H*Ph), 4.27 (dd, *J* = 15.3, 5.9 Hz, 1H, C*H*HPh), 3.99-3.88 (m, 2H, C*HOH*), 1.40 (s, 9H, $(CH_3)_3$), 1.07 (d, *J* = 6.3 Hz, 3H, $CHCH_3$)

^{13}C NMR (125 MHz, $CDCl_3$) δ 170.7, 155.5, 139.4, 128.2, 127.1, 126.7, 78.3, 66.7, 60.4, 42.0, 28.2, 20.3

LRMS (ES+): 309.2 ($[M+H]^+$, 50), 617.3 ($[2\times M+H]^+$, 50),

HRMS: (ES+): measured 309.1813, ($[C_{16}H_{24}N_2O_4+H]^+$) requires 309.1814

tert-Butyl (S)-2-(benzylcarbamoyl)pyrrolidine-1-carboxylate (_39_)

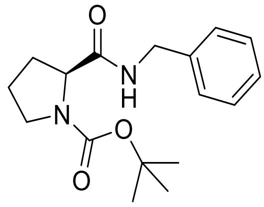

Prepared according to general procedure A with 20 mol% $B(OCH_2CF_3)_3$ from Boc-proline (430 mg, 2.0 mmol) and benzylamine (240 µL, 2.2 mmol) for 24 h, purified using the standard resin workup procedure and further purified by column chromatography (CH_2Cl_2:MeOH, 95:5) to yield **39** as a white solid (471 mg, 77%).

Rf: 0.4 (CH_2Cl_2:MeOH, 99:1)

mp 127-128 °C [Lit.[29]124-125 °C];

$[\alpha]_D^{25}$-75.9 (*c* 1.0, CH_2Cl_2) [Lit.[25] $[\alpha]_D^{25}$-77 (*c* 1.0, CH_2Cl_2)] ;

ν_{max} (solid/cm^{-1}) 3305, 2993, 1682, 1653, 1527, 1392;

^{1}H NMR (400 MHz, $CDCl_3$, 60 °C) δ 8.2 (br s, 1H, N*H*), 7.32-7.22 (m, 5H, ArH), 4.37 (dd, *J* = 15.1, 6.3 Hz, 1H, CH*H*Ph), 4.26 (dd, *J* = 15.1, 5.8 Hz, 1H, C*H*HPh), 4.12 (d, *J* = 6.5 Hz, 1H, C*H*), 3.41-3.29 (m, 2H, BocNCH_2), 2.0 (br s, 1H, CHC*H*H), 1.87-1.75 (m, 3H, CHC*H*HCH_2), 1.34 (s, 9H, 3×CH_3);

^{13}C NMR (150 MHz, $CDCl_3$, mixture of rotamers) δ 172.5, 172.1, 156.0, 154.9, 138. 5, 138.2, 128.8, 127.9, 127.8, 127.5, 80.8, 80.6, 61.5, 60.2, 47.3, 43.4, 31.2, 28.4, 28.2, 24.8, 23.9;

LRMS (ES+): 205.1([M+H-Boc]$^+$, 100), 305.2 ([M+H]$^+$, 10), 327.2 ([M+Na]$^+$, 20);

Enantiopurity was determined by chiral HPLC to be >99:1 er: Chiral HPLC OD1 column, 90:10 hexane:^{i}PrOH, flow 0.8 mL/min, λ = 218 nm, 20 °C, t_R= 10.1 min (R enantiomer), and 17.2 (S enantiomer).

Data in agreement with literature.[29]

Benzyl (*tert*-butoxycarbonyl)-L-phenylalanyl-L-valinate (40)

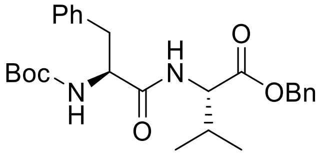

Prepared according to general procedure A with 20 mol% $B(OCH_2CF_3)_3$ from L-Boc-Phe (2.65 g, 10 mmol) and L-H-Val-OBn (2.28 g, 11 mmol) for 15 h, to yield **40** as a white solid (4.1 g, 94%).

$[\alpha]_D^{20}$ – 7.2 (*c* 1.0, $CHCl_3$);

mp 63-64 °C

ν_{max} (solid/cm^{-1}) 3308, 2968, 2931, 1734, 1658

^{1}H NMR (300 MHz, $CDCl_3$) δ 7.38-7.33 (m, 5H, Ar*H*), 7.27-7.25 (m, 2H, Ar*H*), 7.22-7.19 (m, 3H, Ar*H*), 6.56 (br d, *J* = 7.7 Hz, 1H, N*H*), 5.18 (d, *J* = 7.8 Hz, 1H, N*H*), 5.13 (s, 2H, OCH_2), 4.53 (dd, *J* = 8.6, 4.9 Hz, 1H, C*H*CH(CH_3)$_2$), 4.4 (br d, *J* = 6.3 Hz, 1H, C*H*CHPh), 3.06 (m, 2H, CHCH_2), 2.13 (sext, *J* = 6.8, 1H, C*H*(CH_3)$_2$), 1.41 (s, 9H, (CH_3)$_3$), 0.86 (d, *J* = 6.9 Hz, 3H, CHCH_3CH$_3$), 0.80 (d, *J* = 6.9 Hz, 3H, CHCH$_3$CH_3)

^{13}C NMR (75 MHz, $CDCl_3$) δ 171.7, 171.5, 155.6, 136.8, 135.4, 129.5, 128.7, 128.6, 128.6 128.5, 127.1, 127.0, 80.2, 67.1, 57.3, 55.9, 38.1, 31.4, 28.4, 19.0, 17.8, 14.3

LRMS (ES+): 455.3 ([M+H]$^+$, 90), 909.5 ([2×M+H]$^+$, 100)

HRMS (ES+): Measured 455.2546, ([$C_{26}H_{34}N_2O_5$+H]$^+$), requires 455.2546

Overlay of spectra of Boc-L-Phe-L-Val-OBn (**40**) and Boc-D-Phe-L-Val-OBn in section 6.a. of the SI confirms diastereopurity of dipeptide **40**.

***tert*-Butyl (*tert*-butoxycarbonyl)-D-alanyl-L-valinate (41)**

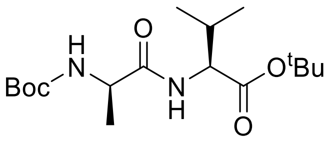

Prepared according to general procedure A with 20 mol% $B(OCH_2CF_3)_3$ from D-Boc-Ala-OH (380 mg, 2.0 mmol) and H-L-Val-O^{t}Bu (380 mg, 2.2 mmol) for 20 h, and purified using the standard resin workup procedure to yield **41** as a white solid (640 mg, 93%)

mp 132-133 °C

$[\alpha]_D^{20}$ +32.0 (*c* 1.0, $CHCl_3$);

ν_{max} (solid/cm^{-1}) 3350, 3327, 2926, 1701, 1648

^{1}H NMR (400 MHz, $CDCl_3$, 60 °C) δ 6.51 (br s, 1H, N*H*), 4.90 (br s, 1H, N*H*), 4.40 (dd, *J* = 8.8, 4.5 Hz, 1H, C*H*CH$(CH_3)_2$), 4.18 (app q, *J* = 7.0 Hz, 1H, C*H*CH_3), 2.18-2.11 (m, 1H, C*H*$(CH_3)_2$), 1.47 (s, 9H, $(CH_3)_3$), 1.45 (s, 9H, $(CH_3)_3$), 1.37 (d, *J* = 7.0 Hz, 3H, CHC*H*$_3$), 0.94 (d, *J* = 6.9 Hz, 3H, CHC*H*$_3$CH$_3$), 0.91 (d, *J* = 6.9 Hz, 3H, CHCH$_3$C*H*$_3$)

^{13}C NMR (125 MHz, $CDCl_3$) 172.5, 171.0, 155.5, 82.1, 80.2, 57.3, 50.4, 31.6, 28.4, 28.2, 19.0, 18.5, 17.5

LRMS (ES+): 345.2 ([M]$^+$, 60), 689.4 ([2×M]$^+$, 100)

HRMS: (ES+): measured 345.2390, ($[C_{17}H_{32}N_2O_5+H]^+$)requires 345.2390

Overlay of spectra of Boc-D-Ala-L-Val-O^{t}Bu (**41**) and Boc-DL-Ala-L-Val-O^{t}Bu in section 6.a. of the SI confirms diastereopurity of dipeptide **41**.

***tert*-Butyl (*tert*-butoxycarbonyl)-D-alanyl-L-phenylalaninate (42)**

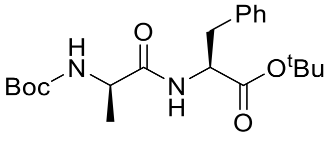

Prepared according to general procedure A with 20 mol% $B(OCH_2CF_3)_3$ from D-Boc-Ala-OH (946 mg, 5.0 mmol) and L-H-Phe-O^{t}Bu (1.217 g, 5.5 mmol) for 16 h, and purified using the standard resin workup procedure to yield **42** as a white solid (1.880 g, 96%)

mp 114-115 °C

$[\alpha]_D^{20}$ +45.5 (*c* 1.0, $CHCl_3$);

ν_{max} (solid/cm^{-1}) 3329, 2973, 2930, 1657, 1510

^{1}H NMR (600 MHz, $CDCl_3$) δ 7.28-7.26 (m, 2H, Ar*H*), 7.24-7.21 (m, 1H, Ar*H*), 7.16-7.14 (m, 2H, Ar*H*), 6.6 (br s, 1H, N*H*), 5.0 (br s, 1H, N*H*), 4.73 (q, *J* = 6.0 Hz, 1H, C*H*CH_3), 4.18 (br t, *J* = 6.1 Hz, 1H, C*H*CH_2Ph), 3.09 (dd, *J* = 5.8, 2.5 Hz, 2H, CH_2), 1.43 (s, 9H, $(CH_3)_3$), 1.40 (s, 9H, $(CH_3)_3$), 1.30 (d, *J* = 7.1 Hz, 3H, CHCH_3)

^{13}C NMR (125 MHz, $CDCl_3$) δ 172.1, 170.5, 155.4, 136.1, 129.7, 128.5, 127.1, 82.5, 80.2, 53.6, 50.2, 38.2, 28.4, 28.1, 18.8

LRMS (ES+): 393.3 ([M]$^+$, 100), 410.3 ([M+17]$^+$, 30)

HRMS: (ES+): measured 393.2385, ([$C_{21}H_{32}N_2O_5$+H]$^+$), requires 393.2384

Overlay of spectra of Boc-D-Ala-L-Phe-O^{t}Bu (**42**) and Boc-DL-Ala-L-Phe-O^{t}Bu in section 6.a. of the SI confirms diastereopurity of dipeptide **42**.

***tert*-Butyl (*tert*-butoxycarbonyl)-L-threonyl-L-valinate (43)**

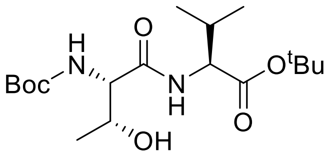

Prepared according to general procedure A with 20 mol% $B(OCH_2CF_3)_3$ from L-Thr-Ala-OH (436 mg, 2.0 mmol) and L-H-Val-O^{t}Bu (380 mg, 2.2 mmol) for 18 h, and purified using the standard resin workup procedure to yield **43** as a white solid (724 mg, 95%)

$[\alpha]_D^{20}$ -40.7 (*c* 1.0, $CHCl_3$);

mp 115-116 °C

ν_{max} (solid/cm^{-1}) 3526, 3342, 3267, 2971, 2932, 2893, 1734, 1664, 1639, 1529

^{1}H NMR (500 MHz, $CDCl_3$) δ 7.1 (br d, *J* = 8.7 Hz, 1H, N*H*), 5.6 (br d, *J* = 7.8 Hz, 1H, N*H*), 4.34 (dd, *J* = 8.7, 4.7 Hz, 1H, C*H*$CH(CH_3)_2$), 4.26 (dq, *J* = 6.3, 3.4 Hz, 1H, C*H*OH), 4.08 (dd, *J* = 7.8, 2.0 Hz, 1H, C*H*CHOH), 3.87 (br d, *J* = 3.4 Hz, 1H, O*H*), 2.10 (m, 1H, C*H*$(CH_3)_2$), 1.40 (s, 9H, $(CH_3)_3$), 1.39 (s, 9H, $(CH_3)_3$), 1.12 (d, *J* = 6.3 Hz, 3H, CHOHCH_3), 0.87 (d, *J* = 6.9 Hz, 3H, $CHCH_3CH_3$), 0.84 (d, *J* = 6.9 Hz, 3H, $CHCH_3CH_3$)

^{13}C NMR (125 MHz, $CDCl_3$) δ 171.5, 170.7, 156.4, 82.0, 80.1, 66.9, 58.0, 57.7, 31.1, 28.3, 28.0, 19.0, 18.1, 17.5;

LRMS (ES+): 375.2 ([M+H]$^+$, 100), 766.5 ([2×M+H]$^+$, 60);

HRMS (ES+): measured 375.2499, ([$C_{18}H_{34}N_2O_6$+H]$^+$), requires 375.2495

Benzyl (*tert*-butoxycarbonyl)-L-threonyl-L-valinate (44)

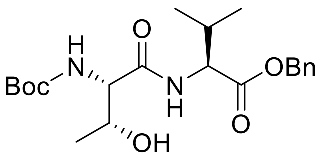

Prepared according to general procedure A with 20 mol% $B(OCH_2CF_3)_3$ from L-Thr-Ala-OH (390 mg, 1.78 mmol) and L-H-Val-OBn (405 mg, 1.96 mmol) for 15 h, to yield **43** as a viscous oil (686 mg, 94%)

$[\alpha]_D^{20}$ – 54.3 (*c* 1.0, $CHCl_3$);

ν_{max} (film/cm^{-1}) 3326, 2970, 2933, 2877, 1737, 1657

^{1}H NMR (600 MHz, $CDCl_3$) δ 7.34-7.30 (m, 5H, Ar*H*), 7.23 (d, *J* = 8.6 Hz, 1H, N*H*), 5.66 (d, *J* = 7.8 Hz, 1H, N*H*), 5.19 (d, *J* = 12.2 Hz, 1H, OC*H*HPh), 5.12 (d, *J* = 12.2 Hz, 1H, OC*H*HPh), 4.54 (dd, *J* = 8.6, 4.8 Hz, 1H, C*H*CH$(CH_3)_2$), 4.29 (m, 1H, C*H*OH), 4.12 (d, *J* = 6.1 Hz, 1H, C*H*CHOH), 3.78 (br s, 1H, O*H*), 2.21 (oct, *J* = 6.8 Hz, 1H, C*H*$(CH_3)_2$), 1.43 (s, 9H, $(CH_3)_3$), 1.16 (d, *J* = 6.4 Hz, 3H, CHOHC*H*$_3$), 0.90 (d, *J* = 6.9 Hz, 3H, $CHCH_3CH_3$), 0.84 (d, *J* = 6.9 Hz, 3H, $CHCH_3CH_3$);

^{13}C NMR (125 MHz, $CDCl_3$) δ 171.8, 171.5, 156.6, 135.4, 128.7, 128.6, 128.5, 80.4, 67.2, 66.9, 57.9, 57.4, 31.0, 28.4, 19.2, 18.11, 17.6

LRMS (ES+): 409.2 ([M+H]$^+$, 100), 309.2 ([M-Boc+H]$^+$, 20),

HRMS: (ES+): measured 409.2331, ($[C_{21}H_{32}N_2O_6+H]^+$), requires 409.2339

***tert*-Butyl (*tert*-butoxycarbonyl)glycylglycylglycinate (45)**

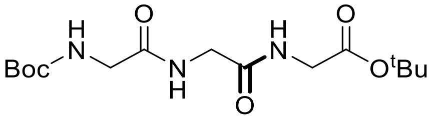

Prepared according to general procedure A with 20 mol% $B(OCH_2CF_3)_3$ from Boc-Gly-Gly (464 mg, 2.0 mmol) and H-L-Val-O^{t}Bu (232 mg, 2.0 mmol) for 24 h, purified using the standard resin workup procedure and further purified by column chromatography, to yield **45** as a white solid (569 mg, 82%)

mp 118-119 °C

ν_{max} (solid/cm^{-1}) 3333, 2973, 2931, 1732, 1698, 1662

^{1}H NMR (500 MHz, $CDCl_3$, 60 °C) δ 6.7 (br s, 1H, N*H*), 6.5 (br s, 1H, N*H*), 5.1 (br s, 1H, N*H*), 4.00 (d, *J* = 5.5 Hz, 2H, C*H*$_2$), 3.92 (d, *J* = 5.3 Hz, 2H, C*H*$_2$), 3.84 (d, *J* = 5.8 Hz, 2H, C*H*$_2$), 1.47 (s, 9H, $(CH_3)_3$), 1.46 (s, 9H, $(CH_3)_3$)

^{13}C NMR (125 MHz, $CDCl_3$) δ 170.8, 169.6, 169.0, 156.6, 82.2, 80.1, 44.2, 42.9, 41.9, 28.4, 28.1

LRMS (ES+): 346.2 ($[M]^+$, 60), 691.4 ($[2\times M]^+$, 100)

HRMS: (ES+): measured 346.1979, ($[C_{15}H_{27}N_3O_6+H]^+$), requires 346.1978

***(S)*-2-Amino-*N*-benzyl-3-(1H-indol-3-yl)propanamide (46)**

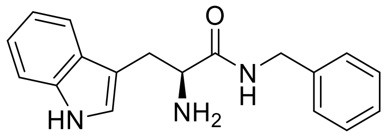

Prepared according to general procedure B, from tryptophan (408 mg, 2.0 mmol) and benzylamine (327 µL, 3.0 mmol) for 16 h. Product was purified by column chromatography (CH_2Cl_2:MeOH, 96:4) to yield an off white solid (529 mg, 90%)

Rf: 0.3 (CH_2Cl_2:MeOH, 95:5)

$[\alpha]_D^{20}$ +20.6 (*c* 1.0, MeOH);

mp 134-135 °C;

ν_{max} (solid/cm^{-1}) 3204, 1640, 1518, 1455;

^1H NMR (600 MHz, $CDCl_3$) δ 8.69 (s, 1H, N*H*), 7.71 (t, *J* = 5.8 Hz, 1H, Ar*H*), 7.67 (d, *J* = 7.9 Hz, 1H, Ar*H*), 7.38 (d, *J* = 8.1 Hz, 1H, Ar*H*), 7.34-7.26 (m, 3H, Ar*H*), 7.20-7.23 (m, 3H, Ar*H*), 7.13 (m, 1H, Ar*H*), 6.99 (d, *J* = 2.1 Hz, 1H, N*H*), 4.46 (d, *J* = 5.9 Hz, 2H, CONHCH_2), 3.77 (dd, *J* = 8.8, 4.1 Hz, 1H, C*H*CH_2), 3.43 (dd, *J* = 14.5, 4.1 Hz, 1H, CHCH_2), 2.99 (dd, *J* = 14.5, 8.8 Hz, 1H, CHCH_2), 1.52 (br s, 2H, NH_2);

^{13}C NMR (150 MHz, $CDCl_3$) δ 175.2, 138.5, 136.7, 128.8, 127.8, 127.5, 127.5, 123.6, 122.3, 119.6, 119.0, 111.6, 111.4, 55.8, 43.3, 31.1;

HRMS (ES+): measured 294.1601, ($[C_{18}H_{19}N_3O+H]^+$), requires 294.1601;

Enantiomeric ratio was determined using Marfey's reagent to be >95:5

***(S)*-2-Amino-*N*-benzyl-3-methylbutanamide (47)**

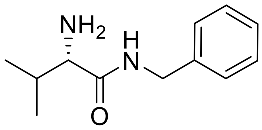

Prepared according to general procedure B, from valine (234 mg, 2.0 mmol) and benzylamine (327 µL, 3.0 mmol) for 24 h. Product was purified by column chromatography (CH_2Cl_2:MeOH, 95:5) to yield a white solid (230 mg, 57%).

Rf: 0.3 (CH_2Cl_2:MeOH, 95:5)

$[\alpha]_D^{20}$+8.5 (*c* 1.0, MeOH);

mp 117-118 °C [lit.[32] 110-112 °C];

ν_{max} (solid/cm^{-1}) 3286 (br), 2955, 2925, 2870, 1640, 1540.86, 1494, 1426, 1345;

^{1}H NMR (600 MHz, $CDCl_3$) δ 7.65 (br s, 1H, N*H*), 7.36-7.31 (m, 2H, Ar*H*), 7.30-7.26 (m, 3H, Ar*H*), 4.48 (dd, *J* = 14.7, 6.0 Hz, 1H, CONHCH*H*), 4.43 (dd, *J* = 14.7, 6.0 Hz, 1H, CONHCH*H*), 3.29 (d, *J* = 3.3 Hz, 1H, C*H*NH_2), 2.37 (m, 1H, C*H*CH_3), 1.41 (br s, 2H, NH_2), 1.01 (d, *J* = 6.9 Hz, 3H, CHCH_3), 0.84 (d, *J* = 6.9 Hz, 3H, CHCH_3);

^{13}C NMR (150 MHz, $CDCl_3$) δ 174.4, 138.7, 128.7, 127.9, 127.5, 60.3, 43.2, 30.9, 19.9, 16.1;

LRMS (ES+): 207.2 ([M+H]$^+$, 100);

HRMS (ES+): measured 207.1499, ([$C_{12}H_{18}N_2O$+H]$^+$), requires 207.1497.

Enantiomeric ratio was determined using Marfey's reagent to be >95:5

Compound (**47**) was also, prepared according to general procedure B using 30 mol% $B(OCH_2CF_3)_3$, from valine (586 mg, 5.0 mmol) and benzylamine (820 µL, 7.5 mmol) for 24 h. The product was purified by column chromatography (CH_2Cl_2:MeOH, 95:5) to yield a white solid (855 mg, 83%); Spectroscopic data is identical to that above. Enantiomeric ratio was determined using Marfey's reagent to be 95:5

Compound (**47**) was also, prepared according to general procedure B using 20 mol% $B(OMe)_3$, from Valine (234 mg, 2.0 mmol) and benzylamine (327 µL, 3.0 mmol) for 24 h. The product was purified by column chromatography (CH_2Cl_2:MeOH, 95:5) to yield a white solid (35 mg, 8%); Spectroscopic data is identical to that above. Enantiomeric ratio was determined using Marfey's reagent to be >95:5.

Data in agreement with literature.[32]

***(2S,3S)*-2-Amino-*N*-benzyl-3-methylpentanamide (48)**

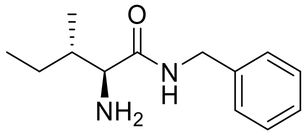

Prepared according to general procedure B using 30 mol% $B(OCH_2CF_3)_3$, from Isoleucine (656 mg, 5.0 mmol) and benzylamine (818 µL, 7.5 mmol) for 36 h. Product was purified by column chromatography (CH_2Cl_2:MeOH, 96:4) to yield a clear oil (788 mg, 72%);

R_f: 0.3 (CH_2Cl_2:MeOH, 95:5)

$[\alpha]_D^{20}$+3.2 (*c* 1.0, MeOH);

ν_{max} (film/cm^{-1}) 3310, 2962, 2930, 2875, 1647, 1516, 1498;

^{1}H NMR (600 MHz, $CDCl_3$) δ 7.67 (br s, 1H, N*H*), 7.23-7.31 (m, 5H, Ar*H*), 4.41 (d, *J* = 4.4 Hz, 2H, CONHCH_2), 3.27 (d, *J* = 3.8 Hz, 1H, C*H*NH_2), 1.96-2.03 (m, 1H, CH_3C*H*), 1.34-1.42 (m, 1H, CH_3C*H*H), 1.31 (br s, 2H, NH_2), 1.04-1.10 (m, 1H, CH_3C*H*H), 0.94 (d, *J* = 6.9 Hz, 3H, CH_3CH), 0.88 (t, *J* = 7.4 Hz, 3H, C$H_3$$CH_2$);

^{13}C NMR (150 MHz, $CDCl_3$) δ 174.4, 138.7, 128.7, 127.8, 127.4, 60.0, 43.1, 38.1, 23.8, 16.3, 12.0;

HRMS (ES+): measured 221.1648 ($[C_{13}H_{20}N_2O+H]^+$), requires 221.1648.

(*S*)-2-Amino-*N*-benzyl-4-(methylthio)butanamide (49)

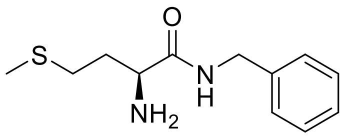

Prepared according to general procedure B, from methionine (746 mg, 5.0 mmol) and benzylamine (818 µL, 7.5 mmol) for 24 h. Product was purified by column chromatography (CH_2Cl_2:MeOH, 95:5) to yield a pale yellow oil (1.07 g, 90%);

Rf: 0.3 (CH_2Cl_2:MeOH, 95:5)

$[\alpha]_D^{20}$ -2.7 (*c* 1.0, MeOH);

ν_{max} (solid/cm^{-1}) 3287, 2965, 2912, 1738, 1659

^{1}H NMR (500 MHz, $CDCl_3$) δ 7.69 (br s, 1H, N*H*), 7.29-7.27 (m, 2H, Ar*H*), 7.23-7.20 (m, 3H, Ar*H*), 4.37 (d, *J* = 6.0 Hz, 2H, CH_2Ph), 3.46 (dd, *J* = 8.3, 4.6 Hz, 1H, C*H*NH_2), 2.55-2.53 (m, 2H, CH_2S), 2.15-2.09 (m, 1H, SCH_2C*H*H), 2.04 (s, 3H, SCH_3), 1.75-1.69 (m, 1H, SCH_2C*H*H), 1.5 (br s, 2H, NH_2)

^{1}H NMR (125 MHz, $CDCl_3$) δ 174.6, 138.5, 128.8, 127.8, 127.6, 54.4, 43.3, 34.2, 30.8, 15.4

LRMS (ES+): 239.1 ($[M+H]^+$, 100), 477.2 ($[2\times M+H]^+$, 30)

HRMS (ES+): measured 239.1220 ($[C_{12}H_{18}N_2OS+H]^+$), requires 239.1218.

Enantiomeric ratio was determined using Marfey's reagent to be >95:5

Spectroscopic data in agreement with literature of opposite enantiomer [(R)-**49**].[34]

(S)-2-Amino-N-benzyl-4-methylpentanamide (50)

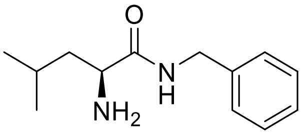

Prepared according to general procedure B, from leucine (262 mg, 2.0 mmol) and benzylamine (327 µL, 3.0 mmol) for 26 h. Product was purified by column chromatography (CH_2Cl_2:MeOH, 96:4) to yield a white solid (320 mg, 73%);

Rf: 0.4 (CH_2Cl_2:MeOH, 95:5)

$[\alpha]_D^{20}$+3.1 (*c* 1.0, MeOH);

mp 40-41 °C;

ν_{max} (solid/cm^{-1}) 3307 (br), 2950, 2933, 2867,1646, 1521, 1497;

^1H NMR (600 MHz, $CDCl_3$) δ 7.79 (br s, 1H, N*H*), 7.26-7.22 (m, 2H, ArH), 7.18-7.16 (m, 3H, ArH), 4.48 (dd, *J* = 14.7, 6.0 Hz, 1H, CONHCH*H*), 4.32 (dd, *J* = 6.0 Hz, 1H, CONHC*H₂*), 3.31 (dd, *J* = 9.8, 4.0 Hz, 1H, C*H*NH_2), 1.68-1.60 (m, 4H, CHCH_2, NH_2), 1.28 (m, 1H, $(CH_3)_2$C*H*), 0.88 (d, *J* = 6.4 Hz, 3H, CH_3), 0.84 (d, *J* = 6.3 Hz, 3H, CH_3),

^{13}C NMR (150 MHz, $CDCl_3$) δ 175.9, 138.7, 128.7, 127.6,127.3, 53.6, 44.2, 43.0, 24.9, 23.5, 21.5;

HRMS (ES+): measured 221.1653, ($[C_{13}H_{20}N_2O+H]^+$), requires 221.1648;

Enantiomeric ratio was determined using Marfey's reagent to be >95:5

(S)-2-Amino-N-benzyl-3-phenylpropanamide (51)

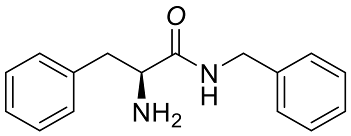

[Scale-up experiment] Prepared according to general procedure B using, phenylalanine (1.65 g, 10 mmol) and benzylamine (1.64 mL, 15 mmol) for 24 h. Product was purified by column chromatography (CH_2Cl_2:MeOH, 96:4) to yield a white solid (2.36 g, 93%)

Rf: 0.3 (CH_2Cl_2:MeOH, 95:5)

$[\alpha]_D^{20}$+12.8 (*c* 1.0, MeOH); [Lit.[32], 94:6 er, $[\alpha]_D^{25}$+9.8 (*c* 1.2, MeOH)];

mp 68-69 °C [lit.[32] 68-69 °C];

ν_{max} (solid/cm^{-1}) 3314 (br), 3062, 3028, 2981, 2926, 1733, 1651, 1603;

^1H NMR (600 MHz, $CDCl_3$) δ 7.59 (br s, 1H, N*H*), 7.37-7.20 (m, 10H, Ar*H*), 4.47 (dd, *J* = 14.8, 6.0 Hz, 1H, PhCH*H*), 4.43 (dd, *J* = 14.8, 5.8 Hz, 1H, PhC*H*H), 3.67 (dd, *J* = 9.1, 4.1 Hz, 1H, C*H*CO), 3.31 (dd, *J* = 13.7, 4.1 Hz, 1H, PhC*H*H), 2.77 (dd, *J* = 13.8, 9.2 Hz 1H, PhCH*H*), 1.37 (br s, 2H, NH_2);

^{13}C NMR (150 MHz, $CDCl_3$) δ 174.2, 138.5, 138.0, 129.5, 128.8, 128.8, 127.9, 127.5, 126.9, 56.6, 43.3, 41.2;

LRMS (ESI+): 255.1 ($[M+H]^+$, 100);

HRMS (ES+): measured 255.1500, ($[C_{16}H_{18}N_2O+H]^+$), requires 255.1497.

Enantiomeric ratio was determined using Marfey's reagent to be >95:5

Data in agreement with literature.[32]

(2S,3R)-2-Amino-*N*-benzyl-3-hydroxybutanamide (52)

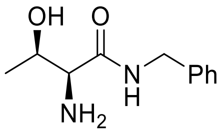

Prepared according to general procedure B, from Threonine (238 mg, 2.0 mmol) and benzylamine (327 µL, 3.0 mmol) for 24 h. Product was purified by column chromatography (CH_2Cl_2:MeOH, 95:5) to yield a light brown solid (235 mg, 56%);

Rf: 0.2 (CH_2Cl_2:MeOH, 95:5)

$[\alpha]_D^{20}$ +10.2 (*c* 0.5, MeOH);

mp 75-76 °C;

ν_{max} (solid/cm^{-1}) 3328, 3264 (br), 2890, 1647, 1605, 1549,

^{1}H NMR (600 MHz, $CDCl_3$) δ 7.85 (br s, 1H, N*H*), 7.33-7.30 (m, 2H, ArH), 7.27-7.24 (m, 3H, ArH), 4.43 (dd, *J* = 15.0, 6.0 Hz, 1H, CONHC*H*H), 4.41 (dd, *J* = 15.0, 6.0 Hz, 1H, CONHCH*H*), 4.29 (dd, *J* = 6.5, 3.4 Hz, 1H, C*H*OH), 3.28 (d, *J* = 3.3 Hz, 1H, C*H*NH_2), 2.51 (br s, 3H, O*H*, NH_2), 1.18 (d, *J* = 6.5 Hz, 3H, CH_3);

^{13}C NMR (150 MHz, $CDCl_3$) δ 173.6, 138.2, 128.7, 127.7, 127.5, 67.9, 59.7, 43.3, 19.1;

HRMS (ES+): measured 209.1279 ($[C_{11}H_{16}N_2O_2+H]^+$), requires 209.1285.

(R)-2-Amino-*N*-benzylbutanamide (53)

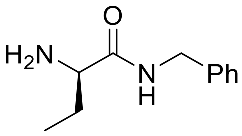

Prepared according to general procedure B, from (R) α-ethylglycine (206 mg, 2.0 mmol) and benzylamine (327 µL, 3.0 mmol) for 24 h. Product was purified by column chromatography (CH_2Cl_2:MeOH, 96:4) to yield a clear oil (311 mg, 81%);

Rf: 0.4 (CH_2Cl_2:MeOH, 95:5)

$[\alpha]_D^{20}$ -8.4 (*c* 1.0, MeOH); [Lit.[32], 94:6 er, $[\alpha]_D^{25}$-2.7 (*c* 1.0, MeOH)];

ν_{max} (film/cm^{-1}) 3291 (br, NH), 3063, 2964, 2875, 1646 (C=O), 1523, 1538;

^{1}H NMR (600 MHz, $CDCl_3$) δ 7.67 (br s, 1H, N*H*), 7.33-7.30 (m, 2H, Ar*H*), 7.27-7.24 (m, 3H, Ar*H*), 4.43 (dd, *J* = 5.9, 3.7 Hz, 2H, CH_2Ph), 3.34 (dd, *J* = 7.8, 4.4 Hz, 1H, C*H*), 1.88 (dqd, *J* = 13.9, 7.6, 4.4 Hz, 1H, CHC*H*H), 1.73 (br s, 2H, NH_2), 1.62-1.54 (m, 1H, CHC*H*H), 0.96 (t, *J* = 7.6 Hz, 3H, CH_3)

^{13}C NMR (150 MHz, $CDCl_3$) δ 175.0, 138.7, 128.8, 127.8, 127.5, 56.5, 43.2, 28.2, 10.2

LRMS (ESI+) 193.2 ($[M+H]^+$, 20), 385.3 ($[2×M+H]^+$, 20)

HRMS (ES+): measured 193.1345, ($[C_{11}H_{16}N_2O+H]^+$), requires 193.1341

Enantiomeric ratio was determined using Marfey's reagent to be >95:5

Data in agreement with literature.[32]

***(S)-N*-Benzylpyrrolidine-2-carboxamide (54)**

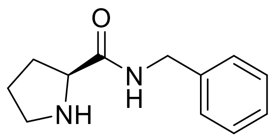

Prepared according to general procedure B, from proline (445 mg, 5.0 mmol) and benzylamine (655 µL, 6.0 mmol [using fewer equivalents of benzylamine aids purification]) for 24 h. Product was purified by column chromatography (CH_2Cl_2:MeOH:AcOH, 96:4:0.5) to yield a faint yellow oil (781 mg, 77%);

Rf: 0.5 (CH_2Cl_2:MeOH, 95:5)

$[\alpha]_D^{20}$ -39.7 (*c* 1.0, $CHCl_3$) [Lit.[59] $[\alpha]_D^{21}$ -45.7 (*c* 1.0, $CHCl_3$)]

ν_{max} (solid/cm^{-1}) 3302, 2965, 2868, 1647, 1518

^1H NMR (400 mHz, $CDCl_3$) δ 7.9 (br s, 1H, CON*H*), 7.34-7.31 (m, 2H, Ar*H*), 7.27-7.25 (m, 3H, Ar*H*), 4.44 (d, *J* = 5.9 Hz, 2H, CH_2Ph), 3.78 (dd, *J* = 9.0, 5.4 Hz, 1H, C*H*), 2.99 (dt, *J* = 10.1, 6.8 Hz, 1H, CHNHC*H*H), 2.88 (dt, *J* = 10.1, 6.3 Hz, 1H, CHNHCH*H*), 2.20-2.12 (m, 1H, CHC*H*H), 2.02-1.93 (m, 2H, 1×CHC*H*H, 1×N*H*), 1.75-1.67 (m, 2H, NHCH_2CH_2)

^{13}C NMR (150 MHz, 100 mHz) δ 175.1, 138.7, 128.6, 127.6, 127.3, 60.7, 47.3, 42.9, 30.8, 26.2

LRMS (ES+): 205.1 ($[M+H]^+$, 35), 409 ($[2×M+H]^+$, 100)

HRMS (ES+): measured 205.1336 ($[C_{12}H_{16}N_2O+H]^+$), requires 205.1335

Enantiomeric ratio was determined using Marfey's reagent to be >95:5

Data in agreement with literature.[59]

3-Amino-*N*-benzylbutanamide (55)

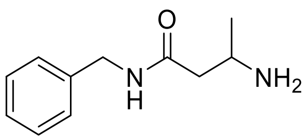

Prepared according to general procedure B, from 3-aminobutananoic acid (412 mg, 4.0 mmol) and benzylamine (818 µL, 7.5 mmol) for 24 h. Product was purified by column chromatography (CH_2Cl_2:MeOH, 90:10) to yield a white solid (424 mg, 55%);

Rf: 0.4 (CH_2Cl_2:MeOH, 95:5)

mp 122-123 °C;

ν_{max} (film/cm^{-1}) 3286 (br), 1631, 1584, 1543;

^{1}H NMR (600 MHz, DMSO-d_6) δ 7.94 (br s, 1H, N*H*), 7.32-7.30 (m, 2H, ArH), 7.26-7.21 (m, 3H, ArH), 4.27 (d, *J* = 3.6 Hz, 2H, CH_2Ph), 3.17 (sx, *J* = 6.3 Hz, 1H, C*H*), 2.16 (d, *J* = 6.3 Hz, 2H, CHCH_2), 1.67 (br s, 2H, NH_2), 0.99 (d, *J* = 6.3 Hz, 3H, CH_3);

^{13}C NMR (150 MHz, DMSO-d_6) δ 171.2, 139.7, 128.3, 127.2, 126.7, 45.3, 44.4, 41.9, 23.5;

LRMS (ES+) 193.2 ([M+H]$^+$, 60), 385.3 ([2M+H]$^+$, 100)

HRMS (ES+): measured 193.1339, ([$C_{11}H_{16}N_2O$+H]$^+$), requires 193.1341

(S)-2-Amino-*N*-phenethylpropanamide (56)

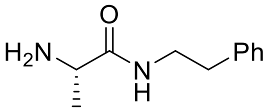

Prepared according to general procedure B, from alanine (445 mg, 5.0 mmol) and 2-phenylethan-1-amine (0.908 mg, 7.5 mmol) for 18 h. Product was purified by column chromatography (CH_2Cl_2:MeOH, 96:4) to yield a clear oil (768 mg, 80%);

Rf: 0.4 (CH_2Cl_2:MeOH, 95:5)

$[\alpha]_D^{20}$ +6.4 (*c* 1.0, $CHCl_3$) [Lit[36] $[\alpha]_D^{20}$ +5.9 (*c* 1.1, $CHCl_3$)];

ν_{max} (film/cm^{-1}) 3357, 3280, 3083, 3026, 2870, 1614, 1540

^{1}H NMR (600 MHz, $CDCl_3$) δ 7.30 (br s, 1H, N*H*), 7.29-7.26 (m, 2H, Ar*H*), 7.21-7.17 (m, 3H, Ar*H*), 3.47 (ddd, *J* = 13.2, 7.2, 2.4 Hz, 2H, NHCH_2), 3.40 (q, *J* = 7.0 Hz, 1H, C*H*CH$_3$), 2.79 (t, *J* = 7.2 Hz, 2H, CH_2Ph), 1.35 (br s, 2H, NH_2), 1.25 (d, *J* = 7.0 Hz, 3H, CHCH_3);

^{13}C NMR (150 MHz, $CDCl_3$) δ 175.8, 139.2, 128.9, 128.7, 126.5, 50.9, 40.3, 35.9, 21.9

LRMS (ESI+): 193.1 ([M+H]$^+$, 100), 385.2 ([2×M+H]$^+$, 80),

HRMS (ES+): measured 193.1335, ([$C_{11}H_{16}N_2O$+H]$^+$), requires 193.1336

Enantiomeric ratio was determined using Marfey's reagent to be >95:5

Data in agreement with literature.[36]

N-(Cyclohexylmethyl)-2-(methylamino)acetamide (57)

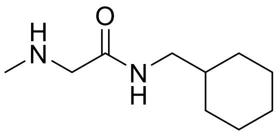

Prepared according to general procedure B, from sarcosine (445 mg, 5.0 mmol) and cyclohexylmethanamine (976 µL, 7.5 mmol) for 24 h. Product was purified by column chromatography (CH_2Cl_2:MeOH, 96:4) to yield a pale yellow oil (704 mg, 77%);

Rf: 0.4 (CH_2Cl_2:MeOH, 95:5)

ν_{max} (film/cm^{-1}) 3305, 2964, 1648, 1610, 1509

^{1}H NMR (600 MHz, DMSO-d_6) δ 7.3 (br s, 1H, N*H*), 3.20 (s, 2H, CH_3NHCH_2), 3.09 (t, *J* = 6.5 Hz, 2H, CONCH_2), 2.39 (s, 3H, CH_3), 1.70-1.62 (m, 6H, Cy*H*), 1.47-1.41 (m, 1H, Cy*H*), 1.24-1.10 (m, 3H, Cy*H*), 0.95-0.89 (m, 2H, Cy*H*)

^{13}C NMR (150 MHz, DMSO-d_6) δ 171.5, 54.7, 45.2, 38.0, 36.9, 30.9, 26.5, 25.9

LRMS (ES+): 185.2 ([M+H]$^+$, 100)

HRMS (ES+): measured 185.1651, ([$C_{10}H_{20}N_2O$+H]$^+$), requires 185.1654

***(S)*-2-Amino-*N*-hexyl-3-phenylpropanamide, (58)**

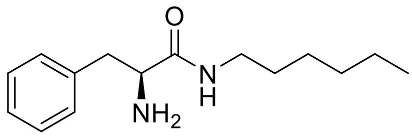

Prepared according to general procedure B, from phenylalanine (826 mg, 5.0 mmol) and hexylamine (991 µL, 7.5 mmol) for 24 h. Product was purified by column chromatography (CH_2Cl_2:MeOH, 96:4) to yield a clear oil (1.11 g, 90%);

Rf: 0.3 (CH_2Cl_2:MeOH, 95:5)

$[\alpha]_D^{20}$ −62.7 (*c* 1.0, $CHCl_3$);

ν_{max} (solid/cm^{-1}) 3299, 2953, 2920, 2855, 1636, 1544

^{1}H NMR (400 mHz, $CDCl_3$) δ 7.31-7.20 (m, 3H, 2 × ArH, N*H*), 7.19-7.24 (m, 3H, ArH), 3.59 (dd, *J* = 9.3, 4.2 Hz, 1H, C*H*), 3.22-3.19 (m, 3 H, NHC*H*2, CH*H*Ph), 2.70 (dd, *J*= 13.7, 9.3 Hz, 1 H, CH*H*Ph), 1.46 (t, *J* = 7.1 Hz, 2H, NHC*H*2CH_2), 1.28-1.34 (m, 8H, NH_2, CH_2CH_2CH_2CH_3), 0.88 (t, *J* = 7.0 Hz, 3H, CH_3)

^{13}C NMR (150 MHz, $CDCl_3$) δ 174.0, 138.1, 129.3, 128.6, 126.7, 56.5, 41.1, 39.1, 31.5, 29.5, 26.6, 22.5, 14.0

LRMS (ES+): 249.3 ([M+H]$^+$, 100), 120.0 (25).

HRMS (ES+): measured 249.1970, [$C_{15}H_{24}N_2O+H$]$^+$, requires 249.1967

Enantiomeric ratio was determined using Marfey's reagent to be >95:5

Tert-Butyl (S)-prolylglycinate (59)

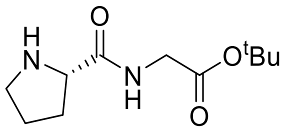

Prepared according to general procedure B, from proline (230 mg, 2.0 mmol) and H-Gly-O^{t}Bu (524 µL, 4.0 mmol) for 24 h. Product was purified by column chromatography (CH_2Cl_2:MeOH, 9:1) to yield a colourless oil (290 mg, 64%);

Rf: 0.3 (CH_2Cl_2:MeOH, 9:1)

$[\alpha]_D^{20}$ −38.1 (*c* 1.0 MeOH); [Lit.[32] 94:6 er, $[\alpha]_D^{20}$ −35.9 (*c* 1.0 MeOH)]

ν_{max} (film/cm^{-1}) 3309 (br), 2975, 2874, 1738, 1650, 1524, 1454;

^{1}H NMR (600 MHz, $CDCl_3$) δ 8.08 (br s, 1H, CONH), 3.91 (m, 2H, C*H*$_2$COO), 3.79 (dd, *J* = 9.2, 5.3 Hz, 1H, C*H)*, 3.02 (dt, *J* = 10.3, 6.8 Hz, 1H, C*H*HNH), 2.95 (dt, *J* = 10.3, 6.3 Hz, 1H, C*H*HNH), 2.42 (br s, 1H, CHN*H*), 2.16-2.12 (m, 1H, CHC*H*H), 1.94-1.89 (m, 1H, CHC*H*H), 1.76-1.69 (m, 2H, CHCH$_2$C*H*$_2$), 1.45 (s, 9H, ^{t}Bu);

^{13}C NMR (150 MHz, $CDCl_3$) δ 175.4, 169.3, 82.1, 60.5, 47.4, 41.6, 30.8, 28.2, 26.2;

LRMS (ES+) 228.10, 155.08 ([M+H]$^+$, 10, 100);

HRMS (ES+): measured 228.1467, ([$C_{11}H_{20}N_2O_3+H$]$^+$), requires 228.1468.

Enantiomeric ratio was determined using Marfey's reagent to be >95:5

Data in agreement with literature.[32]

(*R*)-5-(4-Methylpiperazine-1-carbonyl)pyrrolidin-2-one (60)

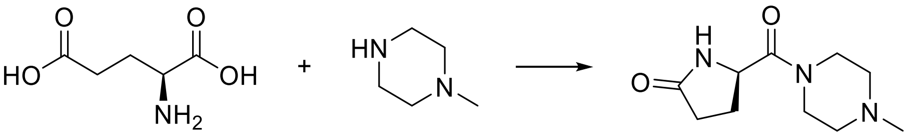

Prepared according to general procedure B, from glutamic acid (735 mg, 5.0 mmol) and *N*-methylpiperazine (833 µL, 7.5 mmol) for 24 h. Product was purified by column chromatography (CH_2Cl_2:MeOH, 9:1) to yield a colourless oil (524 mg, 49%);

Rf: 0.5 (CH_2Cl_2:MeOH, 9:1)

$[\alpha]_D^{20}$ +26.2 (*c* 1.0, MeOH) [Lit.[32] $[\alpha]_D^{20}$ +26.2 (*c* 1.0, MeOH)];

ν_{max} (film/cm^{-1}) 3244 (br, NH), 2939, 2798, 1683, 1657, 1457;

^{1}H NMR (600 MHz, $CDCl_3$) δ 6.79 (br s, 1H, N*H*), 4.46 (dd, *J* = 8.2, 5.4 Hz, 1H, C*H*), 3.60-3.63 (m, 2H, CH_2NCO), 3.44-3.46 (m, 2H, CH_2NCO), 2.30-2.44 (m, 7H, CH*H*CON, CHCH_2, $CH_3N(CH_2)_2$), 2.28 (s, 3H, CH_3), 2.08-2.10 (m, 1H, C*H*HCON);

^{13}C NMR (150 MHz, $CDCl_3$) δ 178.3, 169.9, 54.9, 54.6, 54.1, 46.0, 44.9, 42.2, 29.6, 25.4;

LRMS: (ES+): 212.2 ($[M+H]^+$, 20), 423.3 ($[2\times M+H]^+$, 50), 634.4 ($[3\times M+H]^+$,100);

HRMS (ES+): measured 212.1401, ($[C_{10}H_{17}N_3O_2+H]^+$), requires 212.1399.

Enantiomeric ratio was determined using a chiral shift reagent as described previously[32], to be >95:5.

Data in agreement with literature.[32]

(*S*)-3,5-Dibenzyl-2,2-dimethylimidazolidin-4-one (61)

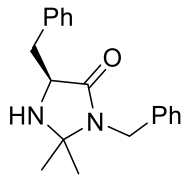

Prepared according to general procedure C, from phenylalanine (826 mg, 5.0 mmol) and benzylamine (818 µL, 7.5 mmol) for 24 h, at which point acetone (745 µL, 10 mmol) was added. Product was purified by column chromatography (CH_2Cl_2:MeOH, 95:5) to give product as a pale yellow oil (1.47 g, 70%).

$[\alpha]_D^{20}$ -54.2 (*c* 1.0, $CHCl_3$);

Rf: product 0.6 (CH_2Cl_2:MeOH, 95:5) [Rf of intermediate: 0.3]

ν_{max} (solid/cm^{-1}) 3319, 3024, 2973, 2920, 2862, 1679, 1493

^{1}H NMR (400 MHz, $CDCl_3$) δ 7.33-7.20 (m, 8H, Ar*H*), 7.11-7.09 (m, 2H, Ar*H*), 4.67 (d, *J* = 15.6 Hz, 1H, NC*H*HPh), 4.11 (d, *J* = 15.5 Hz, 1H, NC*H*HPh), 3.88 (t, *J* = 5.3 Hz, 1H, C*H*), 3.21 (dd, *J* = 14.2, 5.9 Hz, 1H, CHC*H*H), 3.14 (dd, *J* = 14.2, 4.7 Hz, 1H, CHC*H*H), 1.8 (br s, 1H, N*H*), 1.21 (s, 3H, CH_3), 0.98 (s, 3H, CH_3)

^{13}C NMR (100 MHz, $CDCl_3$) δ 174.2, 138.0, 136.7, 129.9, 128.6, 128.4, 127.5, 127.2, 127.0, 76.4, 58.9, 43.6, 36.6, 28.0, 26.2

LRMS (ES+) 295.2 ($[M+H]^+$, 80), 589.4 ($[2\times M+H]^+$, 100)

HRMS (ES+): measured 295.1813, ([$C_{19}H_{22}N_2O$+H]$^+$), requires 295.1810

Enantiopurity was determined by chiral HPLC to be >96:4 er : Compound was analysed by Chiral HPLC ADH column, 4:1 heptane:^{i}PrOH [0.1% isopropylamine], flow 1.0 mL/min, λ = 235 nm, 20 °C, t_R= 7.0 min *(S)* enantiomer, and 8.4 *(R)* enantiomer.

3,5-Dibenzyl-2-(*tert*-butyl)imidazolidin-4-one (62)

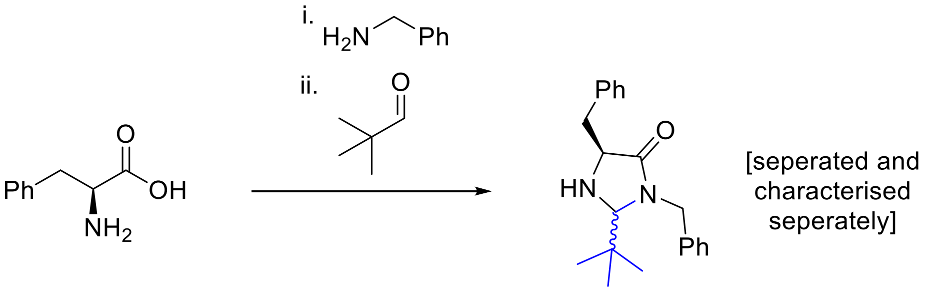

Prepared according to general procedure C, from phenylalanine (826 mg, 5.0 mmol) and benzylamine (818 µL, 7.5 mmol) for 24 h, at which point pivaldehyde (0.860 mL, 10 mmol) was added. Product was purified by column chromatography (CH_2Cl_2:MeOH, 95:5) to give the two diastereomers of product (1.77 g total, 67%). The spectroscopic data for the two separated and isolated diastereoisomers is given below.

(*2R,5S*)-3,5-Dibenzyl-2-(*tert*-butyl)imidazolidin-4-one (62 A)

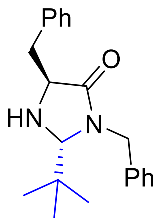

Major product, obtained as a pale yellow oil (0.79 g, 49%)
Rf: product 0.7 (CH_2Cl_2:MeOH, 95:5) [Rf of intermediate: 0.3]

$[\alpha]_D^{20}$ +71.4 (*c* 1.0, $CHCl_3$);

ν_{max} (solid/cm^{-1}) 3393, 3358, 2915, 1684, 1602

^{1}H NMR (400 MHz, $CDCl_3$) δ 7.33-7.29 (m, 5H, Ar*H*), 7.19-7.15 (m, 3H, Ar*H*), 6.76-6.74 (m, 2H, Ar*H*), 5.25 (d, *J* = 15.4 Hz, 1H, NC*H*HPh), 4.00 (d, *J* = 15.4 Hz, 1H, NC*H*HPh), 3.94 (br t, *J* = 5.0 Hz, 1H, C*H*CH$_2$), 3.72 (br d, *J* = 1Hz, 1H, C*H*CCH$_3$), 3.18 (dd, *J* = 14.2, 5.4 Hz, 1H, CHC*H*H), 3.08 (dd, *J* = 14.2, 4.9 Hz, 1H, CHC*H*H), 1.90 (br s, 1H, N*H*), 0.90 (s, 9H, (C*H*$_3$)$_3$)

^{13}C NMR (100 MHz, $CDCl_3$) δ 175.6, 136.6, 135.7, 130.3, 128.7, 128.6, 127.6, 127.3, 126.9, 79.2, 59.5, 46.7, 38.6, 37.2, 26.1

LRMS (ES+) 323.2 ($[M+H]^+$, 100)

HRMS (ES+): measured 323.2125, ($[C_{21}H_{26}N_2O+H]^+$), requires 323.2123

Enantiopurity was determined by chiral HPLC to be 96:4 er: Compound was analysed by Chiral HPLC ADH column, 4:1 heptane:^{i}PrOH [0.1% isopropylamine], flow 1.0 mL/min, λ = 235 nm, 20 °C, t_R= 5.1 min (*SR* enantiomer), and 6.0 (*RS* enantiomer).

This compound has been seen in literature to be prone to racemisation. As reported by Koskinen *et al*.[60] "production of the trans cyclo-adduct [12b] was accompanied by racemization". No further data is provided in that paper. S. A. Selkala, A. M. P. Koskinen, *Eur. J. Org. Chem*. **8**, 1620-1624 (2005)

(*2S,5S*)-3,5-Dibenzyl-2-(*tert*-butyl)imidazolidin-4-one (62 B)

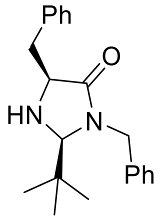

Minor product, obtained as a white solid (0.29 g, 18%)
Rf: product 0.7 (CH_2Cl_2:MeOH, 95:5) [Rf of intermediate: 0.3]

$[\alpha]_D^{20}$ -71.2 (*c* 0.5, $CHCl_3$);

ν_{max} (solid/cm^{-1}) 3393, 3358, 2915, 1684, 1602

^1H NMR (400 MHz, $CDCl_3$) δ 7.29-7.26 (m, 6H, Ar*H*), 7.23-7.19 (m, 2H, Ar*H*), 7.13-7.11 (m, 2H, Ar*H*), 5.03 (d, *J* = 15.9 Hz, 1H, NC*H*HPh), 4.22 (d, *J* = 15.7 Hz, 1H, NC*H*HPh), 4.09 (br s, 1H, C*H*CH_2), 3.82 (br s, 1H, C*H*CCH_3), 3.19 (dd, *J* = 13.7, 3.9 Hz, 1H, CHC*H*H), 3.00 (dd, *J* = 13.7, 7.6 Hz, 1H, CHC*H*H), 1.7 (br s, 1H, N*H*), 0.74 (s, 9H, $(CH_3)_3$)

^{13}C NMR (100 MHz, $CDCl_3$) δ 176.3, 137.9, 136.4, 129.7, 128.6 (2), 127.5, 127.3, 126.7, 79.9, 59.5, 46.4, 38.3, 35.2, 25.6

LRMS (ES+) 323.2 ($[M+H]^+$, 100)

HRMS (ES+): measured 323.2124, ($[C_{21}H_{26}N_2O+H]^+$), requires 323.2123

Enantiopurity was determined by chiral HPLC to be 98:2 er : Compound was analysed by Chiral HPLC ADH column, 4:1 heptane:^{i}PrOH [0.1% isopropylamine], flow 1.0 mL/min, λ = 235 nm, 20 °C, t_R= 6.2 min (RR enantiomer), and 7.4 (SS enantiomer).

Data in agreement with literature.[60]

1-Benzyl-4-methyl-1,4-diazaspiro[4.5]decan-2-one, (63)

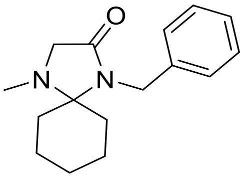

Prepared according to general procedure C, from sarcosine (445 mg, 5.0 mmol) and benzylamine (818 µL, 7.5 mmol) for 24 h, at which point cyclohexanone (2.07 mL, 20 mmol) was added in two equal portions, 1 h apart. Product was purified by column chromatography (CH_2Cl_2:MeOH, 95:5) to give product as a pale yellow oil (1.05 g, 82%).

Rf: product 0.6 (CH_2Cl_2:MeOH, 96:4) [Rf of intermediate: 0.5]

ν_{max} (film/cm^{-1}) 3345, 2931, 2856, 2790, 1687, 1605

^{1}H NMR ($CDCl_3$, 400 mHz) δ 7.31-7.24 (m, 5H, Ar*H*) 4.38 (2H, CH_2Bn), 3.39 (s, 2H, CH_2NMe), 2.32 (s, 3H, CH_3), 1.49-1.68 (m, 7H, Cy*H*), 1.83-1.40 (m, 2H, Cy*H*), 1.09-1.13 (m, 1H, Cy*H*)

^{13}C NMR (150 MHz, $CDCl_3$) δ 173.6, 138.7, 128.5, 127.3, 127.2, 83.7, 58.0, 43.1, 32.1, 39.1, 25.1, 22.8

LRMS (ES+) 259.2 ([M+H]$^+$, 100), 516.4 ([2×M+H]$^+$, 40)

HRMS (ES+): measured 259.1815, ([$C_{16}H_{22}N_2O$+H]$^+$), requires 259.1810

(5S)-2-Ethyl-2,5-dimethyl-3-phenethylimidazolidin-4-one (64)

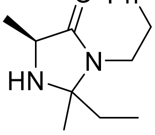

Prepared according to general procedure C, from alanine (445 mg, 5.0 mmol) and benzylamine (1.32 mL, 10.0 mmol) for 24 h, at which point 2-butanone (1.72 mL, 20 mmol) was added in two portions, 1 h apart. Product was purified by column chromatography (CH_2Cl_2:MeOH, 95:5) to a mixture of two diastereoisomers of the product **64** (991 mg, 81%).

Rf: product 0.7 (CH_2Cl_2:MeOH, 95:5) [Rf of intermediate: 0.3]

ν_{max} (solid/cm^{-1}) 3324, 2970, 2930, 1678

^{1}H NMR (600 MHz, $CDCl_3$, Mixture of diastereoisomers in a ratio of 2†:1‡]) δ 7.31-7.22 (m, 5H, Ar*H*), 3.60-3.50†‡ (m, 1.33H, 0.33×NCH*H*‡, 0.33×C*H*‡, 0.66×C*H*†), 3.30† (ddd, *J* = 9.9, 6.6, 3.4 Hz, 1.32H, NCH_2), 3.11-3.06‡ (m, 0.33H, NCH*H*), 2.99-2.89†‡ (m, 2H, CH_2Ph), 1.71-1.50†‡ (m, 3H, 2×C$H_2$$CH_3$, 1×N*H*), 1.35†‡ (d, *J* = 6.9 Hz, 3H, CHCH_3),1.26† (s, 2H, CH_3), 1.19‡ (s, 1H, CCH_3), 0.88† (t, J = 7.4 Hz, 2H, CH_2CH_3), 0.87† (t, J = 7.4 Hz, 1H, CH_2CH_3)

^{13}C NMR (150 MHz, $CDCl_3$, [Mixture of diastereoisomers in a ratio of 2†:1‡]) δ 176.1‡, 176.0†, 139.2†, 139.2‡, 129.1‡, 129.0†, 128.7†, 128.6‡, 126.6†‡, 78.8‡, 78.6†, 54.5‡, 53.6†, 42.8†, 42.8‡, 35.0‡, 35.0†, 32.4‡, 32.2†, 26.9‡, 25.1†, 18.7‡, 17.8†, 8.4‡, 7.6†;

LRMS (ES+) 247.2 ([M+H]$^+$, 100)

HRMS (ES+): measured 247.1806, ([$C_{15}H_{22}N_2O$+H]$^+$), requires 247.1806

Data in agreement with literature.[36]

2-Ethyl-2,5-dimethyl-3-phenethyl-2,3-dihydro-4H-imidazol-4-one (65)

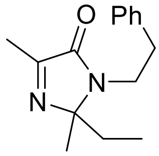

This method is based on a procedure described by Zhao *et. al.*[36]. (5S)-2-ethyl-2,5-dimethyl-3-phenethylimidazolidin-4-one (**65**) (120 mg, 0.48 mmol) was dissolved in CH_2Cl_2 (12 mL) and cooled to 0 °C. Freshly prepared ^{t}BuOCl (see preparation below, 104 mg, 0.96 mmol) was added dropwise, the reaction brought to room temperature and stirred for 2 hours. The mixture was cooled to 0 °C and Et_3N (156 mg, 1.44 mmol) was added dropwise. The reaction mixture was brought back to room temperature and stirred for 18 h, concentrated *in vacuo* and directly purified by column chromatography, to yield a colourless oil (76 mg, 65%).

Preparation of ^{t}BuOCl. This method is based on a procedure described by Mintz *et. al*.[57]
Caution! To avoid vigorous decomposition, the product should be handled only in dim light and should not be heated above its boiling point or be exposed to rubber. Commercial bleach solution (5%, 50 mL) was cooled in an ice bath to ~5 °C. A solution of ^{t}BuOH (3.7 mL, 39 mmol) and glacial acetic acid (2.45 mL, 43 mmol) was added in a single portion to the rapidly stirred bleach solution, and stirring was continued for about 3 min. The reaction mixture was poured into a 250 mL separating funnel and the lower aqueous layer discarded. The oily yellow organic layer was washed first with 10% aqueous sodium carbonate (5 mL) and then with water (5 mL). The product was dried over calcium chloride (0.1 g) and filtered to give a colourless liquid (3.0 g) which was directly used in the oxidation of imidazolidinone (**65**).

ν_{max} (oil/cm^{-1}) 3561, 2976, 2933, 2880, 1694, 1641

^{1}H NMR (600 MHz, $CDCl_3$) δ 7.32-7.30 (m, 2H, Ar*H*), 7.26-7.22 (m, 3H, Ar*H*), 3.61 (ddd, *J* = 13.9, 10.2, 5.8 Hz, 1H, C*H*HCH$_2$), 3.37 (ddd, *J* = 13.8, 10.1, 6.6 Hz, 1H, CH*H*CH$_2$), 3.01 (ddd, *J* = 13.3, 10.1, 5.8 Hz, 1H, C*H*HCH$_2$), 2.96 (ddd, *J* = 13.3, 10.2, 6.6 Hz, 1H, CH*H*CH$_2$), 2.25 (s, 3H, C*H*$_3$), 1.99 (dq, *J* = 14.5, 7.3 Hz, 1H, CH*H*CH$_3$), 1.71 (dq, *J* = 14.5, 7.3 Hz, 1H, CH*H*CH$_3$), 1.33 (s, 3H, C*H*$_3$), 0.55 (t, *J* = 7.3 Hz, 3H, CH_2CH_3);

^{13}C NMR (100 MHz, $CDCl_3$) δ 166.6, 164.2, 138.6, 128.9, 128.8, 126.8, 87.0, 43.0, 34.1, 30.0, 24.4, 14.6, 7.1

Data in agreement with literature.[36]

tert-Butyl pentanoyl-L-valinate (66)

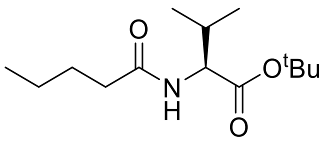

Prepared according to general procedure A, from valeric acid (218 µL, 2.0 mmol) and H-Val-O^{t}Bu (381 µL, 2.2 mmol) for 24 h, and purified using the standard resin workup procedure to yield **66** as a pale yellow oil (468 mg, 91%).

$[\alpha]_D^{20}$ -23.8 (*c* 1.0, $CHCl_3$);

ν_{max} (film/cm^{-1}) 3295, 2958, 2929, 2871, 1732, 1643

^{1}H NMR (600 MHz, $CDCl_3$) δ 6.0 (br s, 1H, N*H*), 4.46 (dd, *J* = 8.8, 4.6 Hz, 1H, CONC*H*), 2.21 (t, *J* = 7.6 Hz, 2H, C*H*$_2$CON), 2.15-2.08 (m, 1H, C*H*(CH$_3$)$_3$), 1.64-1.57 (m, 2H, C*H*$_2$CH$_2$CON), 1.45 (s, 9H, (C*H*$_3$)$_3$), 1.36-1.31 (m, 2H, C*H*$_2$CH$_2$CH$_2$CON), 0.92-0.87 (m, 9H, 6×CH(CH$_3$)$_3$, 3×CH$_2$C*H*$_3$)

^{13}C NMR (150 MHz, $CDCl_3$) δ 173.1, 171.6, 82.0, 57.2, 36.6, 31.6, 28.1, 27.9, 22.5, 19.0, 17.8, 13.9

LRMS (ES+): 258.2 ([M+H]$^+$, 20), 515.4 ([2×M+H]$^+$, 100)

HRMS (ES+): measured 258.2071, ([$C_{14}H_{27}NO_3$+H]$^+$) requires 258.2069

Enantiopurity was determined by chiral HPLC to be >99:1 er: Chiral HPLC OD1 column, 97:3 hexane:^{i}PrOH, flow 0.8 mL/min, λ = 218 nm, 20 °C, t_R= 6.6 min (R enantiomer), and 28.5 (S enantiomer).

1-(1,4-Diazepan-1-yl)butan-1-one (67)

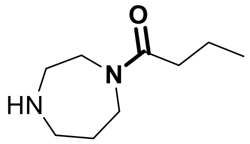

Prepared according to general procedure A, from butyric acid (460 µL, 5.0 mmol) and homopiperazine (750 mg, 7.5 mmol) for 24 h, and purified by column chromatography (EtOAc:MeOH:Et_3N , 8:2:1) to give **67** as a yellow viscous oil (725 mg, 85%).

Rf 0.3 (EtOAc:MeOH:Et_3N, 7:3:1);

ν_{max} (solid/cm^{-1}) 3328, 3283, 2940, 1733

^1H NMR (400 MHz, $CDCl_3$, mixture of rotamers) δ 3.65-3.64 (m, 2H), 3.58-3.54 (m, 1H), 3.51-3.49 (m, 1H), 2.98-2.93 (m, 2 H), 2.89-2.84 (m, 2H), 2.34-2.27 (m, 2H), 2.2 (br s, 1H, N*H*), 1.85-1.78 (m, 2H), 1.71-1.65 (m, 2H), 0.98 and 0.97 (2 × t, *J* = 7.4 Hz, 3H)

^{13}C NMR (100 MHz, $CDCl_3$, mixture of rotamers) δ 172.6, 172.5, 50.9, 50.2, 49.0, 48.6, 48.5, 47.7, 46.9, 44.6, 35.3, 35.1, 30.9, 29.6, 18.7, 18.7, 14.0

LRMS (ES+): 171.2 ($[M+H]^+$, 100)

Data in agreement with literature.[62]

***tert*-Butyl (2-phenylacetyl)-L-prolylglycinate (68)**

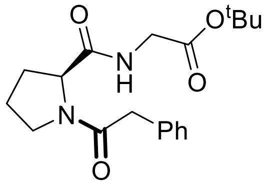

Prepared according to general procedure A, from phenylacetic aicd (136 mg, 2.0 mmol) and *tert*-Butyl (S)-prolylglycinate (**59**) (251 µL, 2.2 mmol) for 24 h. Product was purified by column chromatography (Hexane:EtOAc) to yield a colourless oil (290 mg, 64%);

$[\alpha]_D^{20}$ – 87.0 (*c* 1.0, $CHCl_3$);

ν_{max} (solid/cm^{-1}) 3296, 2975, 2933, 2877, 1739, 1628

^1H NMR (400 MHz, DMSO-d_6, 120 °C) δ 7.7 (br s, 1H, N*H*), 7.30-7.20 (m, 5H, Ar*H*), 4.45 (dd, *J* = 5.0, 2.0 Hz, 1H, NC*H*), 3.75 (d, *J* = 5.8 Hz, 2H, CH_2CO), 3.67-3.53 (m, 4H, 2×CH_2), 2.10-1.86 (m, 4H, 2×CH_2),1.43 (m, 9H, (CH_3)$_3$)

^{13}C NMR (150 MHz, $CDCl_3$, *mixture of rotamers*) δ 171.5, 171.4, 168.8, 134.3, 129.1, 128.8, 127.1, 82.0, 59.9, 47.8, 42.2, 42.1, 28.2, 27.5, 25.1

LRMS (ES+) 347.2 ($[M+H]^+$, 100), 291 ($[M+H-{}^tBu]^+$, 20)

HRMS (ES+): measured 347.1975, ($[C_{19}H_{26}N_2O_4+H]^+$), requires 347.1971

_N_,3-Diphenylpropiolamide (69)

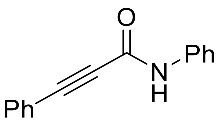

Prepared according to general procedure A, from phenylpropiolic acid (730 mg, 5.0 mmol) and aniline (455 µL, 5.0 mmol) for 24 h, purified using the standard resin workup procedure and recrystallised with hot propanol:H_2O (1:1), to yield **69** as off white crystals (885 mg, 80%).

mp 125-126 °C [Lit.[59] 124-125 °C];

ν_{max} (solid/cm^{-1}) 3210, 3179, 3017, 2210, 1618, 1591

^1H NMR (600 MHz, $CDCl_3$) δ 7.9 (br s, 1H, N*H*), 7.60 (d, *J* = 7.6 Hz, 2H, Ar*H*), 7.56-7.54 (m, 2H, Ar*H*), 7.43 (tt, *J* = 7.5, 1.2 Hz, 1H, Ar*H*), 7.37-7.33 (m, 4H, Ar*H*), 7.15 (t, *J* = 7.4 Hz, 1H, Ar*H*)

^1H NMR (150 MHz, $CDCl_3$) δ 151.3, 137.5, 132.8, 130.5, 129.3, 128.7, 125.1, 120.1, 120.0, 85.9, 83.6;

LRMS (ES+): 222.1 ($[M+H]^+$, 90)

HRMS (ES+): measured 222.0921, ($[C_{15}H_{11}NO+H]^+$), requires 222.0919

Data in agreement with literature.[63]

1-Methyl-_N_-9-methyl-9-azabicyclo[3.3.1]nonan-3-yl)-1H-indazole-3-carboxamide (Granisetron, 70)

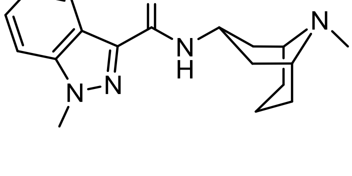

Prepared according to general procedure A with 1 eq of $B(OCH_2CF_3)_3$, from the 1-Methylindazole-3-carboxylic acid (881 mg, 5.0 mmol) and Endo-3-Amine-9-methyl-9-azabicyclo[3,3,1]nonane (770 mg, 5.0 mmol) in PhMe for 24 h, and purified by column chromatography (CH_2Cl_2:MeOH, 9:1) to give **70** as a pale orange viscous oil (1.17 g, 75%) .

ν_{max} (film/cm^{-1}) 3296, 2938, 2827, 1638, 1539

^{1}H NMR (400 MHz, $CDCl_3$) δ 8.38 (d, *J* = 8.1 Hz, 1H, Ar*H*), 7.40-7.35 (m, 2H, Ar*H*), 7.24 (ddd, *J* = 8.1, 6.5, 1.3 Hz, 1H, Ar*H*), 6.81 (br d, *J* = 8.4 Hz, 1H, N*H*), 4.62-4.50 (m, 1H, NHC*H*), 4.04 (s, 3H, ArNCH_3), 3.08 (app d, *J* = 11.0 Hz, 2H, CH_2), 2.56-2.49 (m, 5H CHNCH_3 and CH_2), 2.00-1.89 (m, 3H), 1.53-1.49 (m, 1H), 1.40-1.33 (m, 2H), 1.04-1.01 (m, 2H)

^{13}C NMR (100 MHz, $CDCl_3$) δ 161.9, 141.2, 137.5, 126.7, 122.9, 122.7, 122.4, 109.0, 51.3, 40.8, 40.6, 35.9, 33.1, 24.7, 14.3

LRMS (ES+): 313.2 ($[M+H]^+$, 100)

HRMS (ES+): measured 313.2031, ($[C_{18}H_{24}N_4O+H]^+$), requires 313.2029

Data in agreement with literature.[64]

tert-Butyl _(R)_-(4-oxo-4-(3-(trifluoromethyl)-5,6-dihydro-[1,2,4]triazolo[4,3-a]pyrazin-7(8H)-yl)-1-(2,4,5-trifluorophenyl)butan-2-yl)carbamate (71)

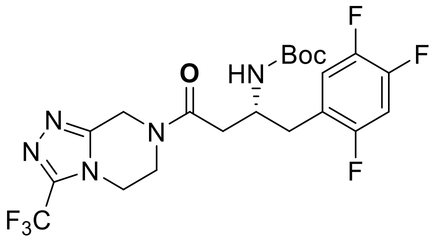

Prepared according to general procedure A, from (*R*)-3-((*tert*-butoxycarbonyl)amino)-4-(2,4,5-trifluorophenyl)butanoic acid (832 mg, 2.5 mmol) and 3-(trifluoromethyl)-5,6,7,8-tetrahydro-[1,2,4]triazolo[4,3-a]pyrazine (528 mg, 2.75 mmol, see note on amine purity on page 42 for the synthesis of **21**) for 36 h, concentrated *in vacuo* and recrystallised with hot propanol:H_2O (2:1), to yield **71** as white crystals (1.08 g, 85%).

Rf: 0.3 (CH_2Cl_2:MeOH, 99:1)

mp 186-187 °C [Lit.[65] 190-191 °C]

$[\alpha]_D^{20}$+22.0 (*c* 1.0, $CHCl_3$) [Lit.[65] $[\alpha]_D^{20}$+23.1 (*c* 1.0, $CHCl_3$)];

ν_{max} (solid/cm^{-1}) 3366, 3052, 2982, 1684, 1634

^{1}H NMR (400 MHz, DMSO-d_6, 80 °C) δ 7.31 (br s, 2H, Ar*H*), 6.5 (br s, 1H, N*H*), 4.89 (br s, 2H, NCH_2), 4.17 (br s, 2H, NCH_2), 4.07 (br s, 1H, NC*H*), 3.96 (br s, 2H, NCH_2), 2.87-2.59 (m, 4H, CH_2CHCH_2), 1.24 (s, 9H, $(CH_3)_3$)

^{13}C NMR (100 MHz, DMSO-d_6, mixture of rotamers]) δ 172.2, 169.4, 156.0 (d, *J* = 241 Hz), 154.8, 151.0, 150.9, 147.8 (d, *J* = 245 Hz), 145.6 (dd, *J* = 243, 13 Hz), 142.5 (d, *J* = 39 Hz), 122.8 (d, *J* =

19 Hz), 119.2 (dd, J = 18.2, 6 Hz), 118.5 (q, J = 270 Hz), 105.4 (dd, J = 29, 21 Hz), 77.7, 77.6, 62.1, 47.7, 47.5, 47.3, 43.6, 43.0, 42.1, 41.2, 40.1, 38.4, 38.0, 37.5, 33.1, 28.1, 28.0, 27.7, 25.5

LRMS (ES+) 508.1 ($[M+H]^+$, 100)

HRMS (ES+): measured 508.1786, ($[C_{21}H_{23}F_6N_5O_3+H]^+$), requires 508.1783

Enantiopurity was measured after the next step of the reaction sequence (Boc-deprotection)

Data in agreement with literature.[65]

(R)-3-Amino-1-(3-(trifluoromethyl)-5,6-dihydro-[1,2,4]triazolo[4,3-a]pyrazin-7(8H)-yl)-4-(2,4,5-trifluorophenyl)butan-1-one (Sitagliptin, 72)

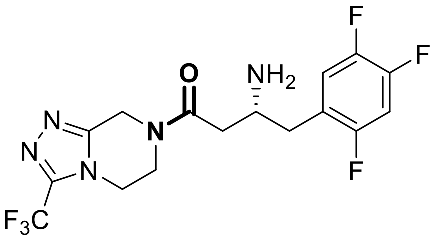

[Boc-deprotection] This method is based on a procedure described by Zhou *et al.*[61]. (R)-**70** (510 mg, 1.01 mmol) was dissolved in 10 mL of 4M HCl in dioxane at room temperature. After being stirred at room temperature for 1 h, the solution was concentrated to give a white foamy solid. The above solid was partitioned between ethyl acetate (20 mL) and 1N aqueous NaOH solution (20 mL). The aqueous layer was extracted with ethyl acetate (3× 20 mL). The combined organic phases were washed with brine, dried over anhydrous $MgSO_4$ and concentration *in vacuo.* The resulting crude was purified by flash chromatography (CH_2Cl_2:MeOH, 10:1) to give (R)-**72** as a white solid (367 mg, 90%).

Rf: 0.3 (CH_2Cl_2:MeOH, 95:5)

$[\alpha]_D^{20}$- 23.7 (c 1.0, $CHCl_3$) [Lit.[65] $[\alpha]_D^{20}$-22.8 (c 1.0, $CHCl_3$)];

mp 114-115 °C [Lit.[65] 114-115 °C]

ν_{max} (solid/cm^{-1}) 3359, 3281, 3250, 2956, 1643, 1590, 1505

^{1}H NMR (600 MHz, $CDCl_3$, mixture of rotamers in a ratio of 6†:4‡) δ 7.07†‡ (ddd, J = 10.0, 8.8, 7.0 Hz, 1H, ArH), 6.93-6.88†‡ (m, 1H, ArH), 5.10-4.98†‡ (m, 2H), 4.25-3.92†‡ (m, 4H), 3.57†‡ (br s, 1H, NH), 2.80-2.76†‡ (m, 1H), 2.70-2.65†‡ (m, 1H), 2.55-2.42†‡ (m, 2H), 1.65 (br s, 2H)

^{13}C NMR (150 MHz, $CDCl_3$) δ 170.6, 170.2, 156.2 (ddd, J = 243, 9, 2 Hz), 150.4, 149.6, 149.1 (td, J = 251, 13 Hz), 146.7 (ddd, J = 245, 13, 3 Hz), 143.9 (t, J = 40 Hz), 121.7 (td, J = 19, 5 Hz), 119.0 (dd, J = 19, 6 Hz), 118.5 (q, J = 269 Hz), 105.7 (dd, J = 29, 20 Hz), 48.6, 43.7, 43.7, 43.3, 42.6, 41.7, 40.4, 40.3, 39.3, 38.1, 36.4

LRMS (ES+): 408.1 ($[M+H]^+$, 100)

HRMS (ES+): measured 408.1262, ($[C_{16}H_{15}F_6N_5O+H]^+$), requires 408.1259

Enantiomeric ratio was determined using Marfey's reagent to be >95:5

Data in agreement with literature. [65]

(R)-5-(Piperidine-1-carbonyl)pyrrolidin-2-one (73, Fasoracetam)

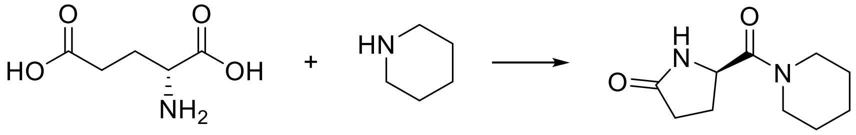

Prepared according to general procedure B, from D-Glutamic acid (735 mg, 5.0 mmol) and piperidine (740 µL, 7.5 mmol) for 24 h. Product was purified by column chromatography (EtOAc:MeOH, 85:15) to yield a colourless oil (375 mg, 38%);

Rf: 0.6 (CH_2Cl_2:MeOH, 95:5)

$[\alpha]_D^{20}$ +26.5 (*c* 1.0, MeOH);

ν_{max} (film/cm^{-1}) 3010, 2942, 2860, 1695, 1638

^{1}H NMR (400 MHz, $CDCl_3$) δ 6.49 (br s, 1H, N*H*), 4.47-4.51 (m, 1H, C*H*), 3.39-3.59 (m, 4H, 2 × NC*H*$_2$), 2.35-2.43 (m, 3H, COC*H*H, $COCH_2CH_2$), 2.08-2.10 (m, 1H, COC*H*H), 1.57-1.66 (m, 6H, $NCH_2CH_2CH_2CH_2$),

^{13}C NMR (100 MHz, $CDCl_3$) δ 178.3, 169.7, 54.0, 45.9, 43.3, 29.5, 26.4, 25.3, 25.3, 24.3;

LRMS (ES+) 197.2, ($[M+H]^+$, 100), 393.4 ($[2M+H]^+$, 20).

HRMS (ES+): measured 197.1294 ($[C_{10}H_{16}N_2O_2+H]^+$), requires 197.1290

Enantiopurity was determined by chiral HPLC to be 96:4 er : Compound was analysed by Chiral HPLC IA column, 5:2 heptane:^{i}PrOH [0.1% isopropylamine], flow 1.0 mL/min, λ = 235 nm, 20 °C, t_R= 13.2 min (R enantiomer), and 18.1 (S enantiomer).

Data in agreement with literature.[66]

***N*-(4-Methoxybenzyl)-2-(thiophen-2-yl)acetamide (74)**

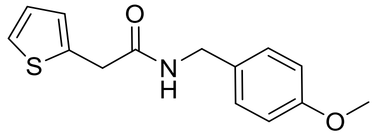

[*Scale up procedure*] Prepared according to general procedure A from 2-Thiopheneacetic acid (11.37 g, 80 mmol) and 4-methoxybenzylamine (12.07 g, 88 mmol) for 24 h, at which point reaction mixture was concentrated to half its volume by emptying solvent from the Dean-Stark side arm trap. Upon cooling of the reaction mixture, the product crystallised spontaneously to yield **74** as light brown solid (20.20 g, 92%).

mp 101-102 °C;

ν_{max} (solid/cm^{-1}) 3265, 2962, 2921, 1645, 1542

^{1}H NMR (600 MHz, $CDCl_3$) δ 7.22 (dd, *J* = 5.2, 1.2 Hz, 1H, Ar*H*), 7.13 (d, *J* = 8.7 Hz, 2H, Ar*H*), 6.97 (dd, *J* = 5.1, 3.5 Hz, 1H, Ar*H*), 6.92 (m, 1H, Ar*H*), 6.82 (dt, *J* = 8.7, 2.5 Hz, 2H, Ar*H*), 6.0 (br s, 1H, N*H*), 4.35 (d, *J* = 5.7 Hz, 2H, CH_2N), 3.79 (s, 2H, CH_2O), 3.78 (s, 3H, CH_3)

^{13}C NMR (150 MHz, $CDCl_3$) δ 169.8, 159.1, 136.3, 130.2, 129.0, 127.5, 127.5, 125.7, 114.2, 55.4, 43.3, 37.7

LRMS (ES+): 262.1 ([M]$^+$, 50), 523.2 ([2×M]$^+$, 100)

HRMS (ES+): measured 262.0908, ([$C_{14}H_{15}NO_2S$+H]$^+$, requires 262.0902

^{1}H and ^{13}C NMR spectra

***N*-benzylbenzamide (2)**

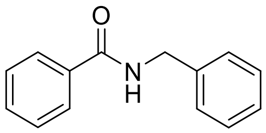

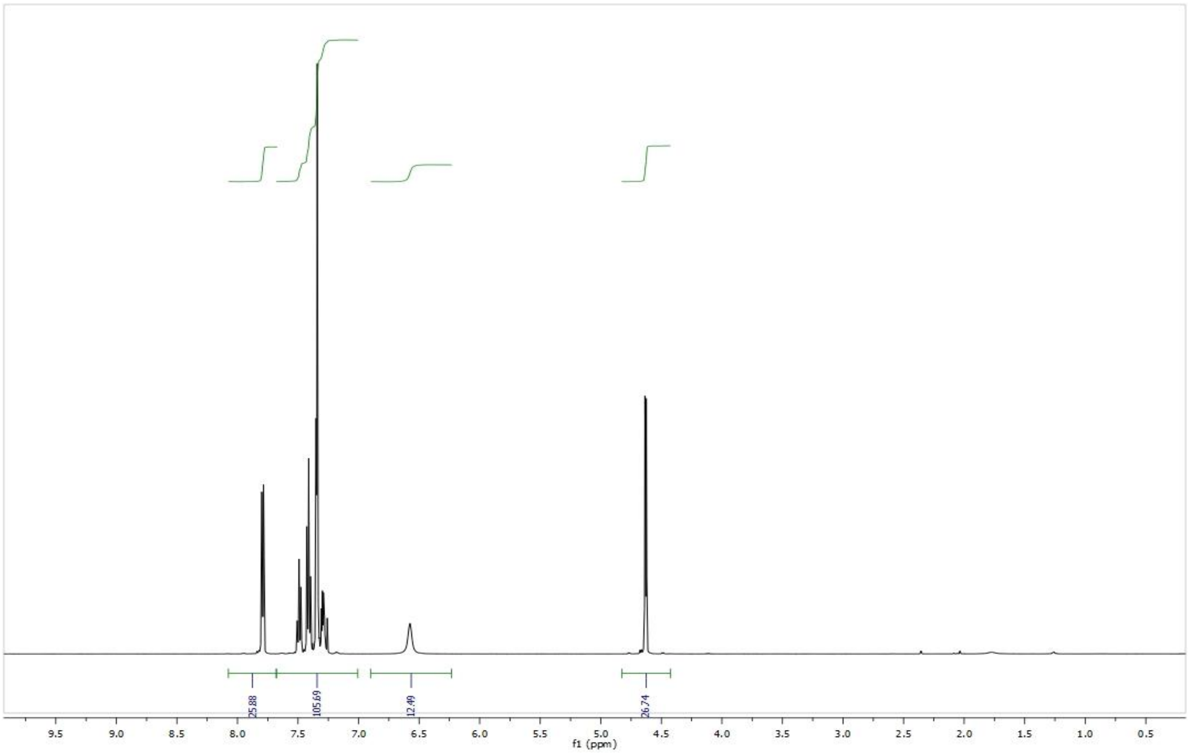

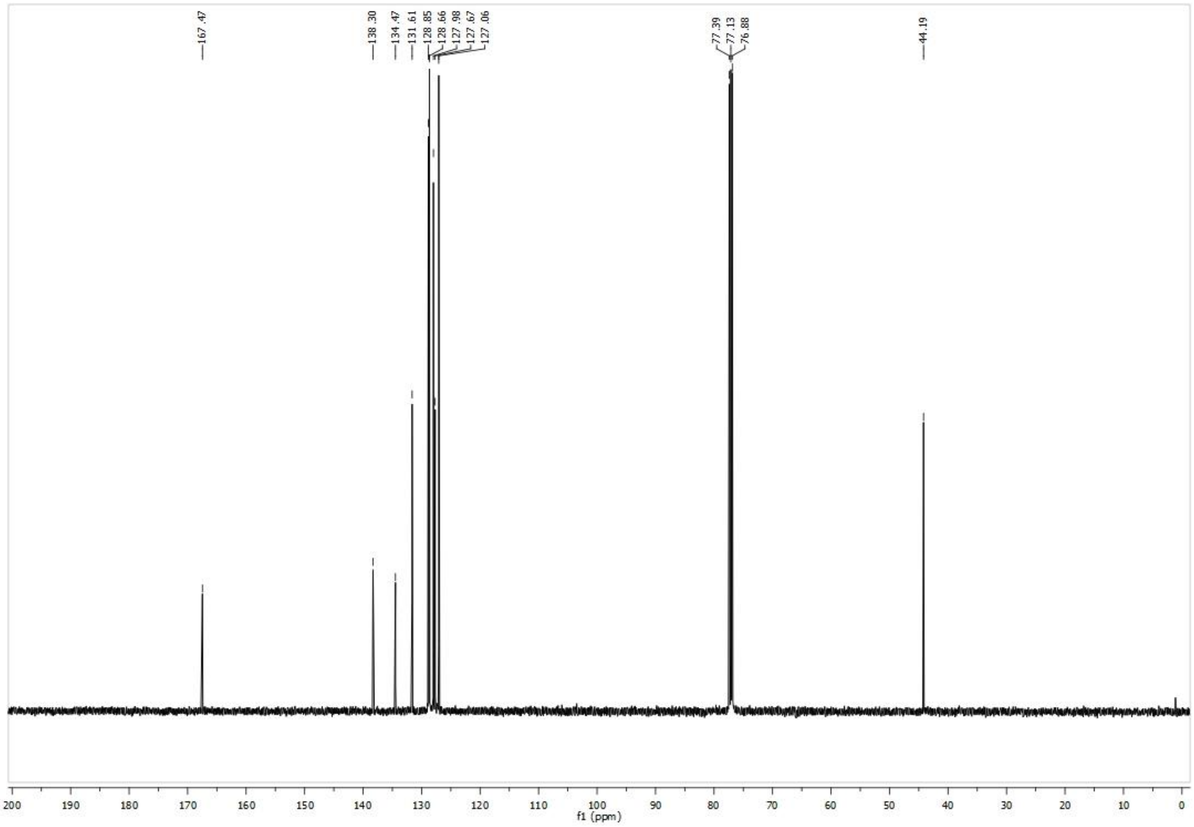

_N_-benzyl-3-methylbutanamide (3)

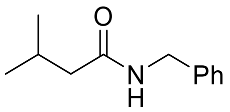

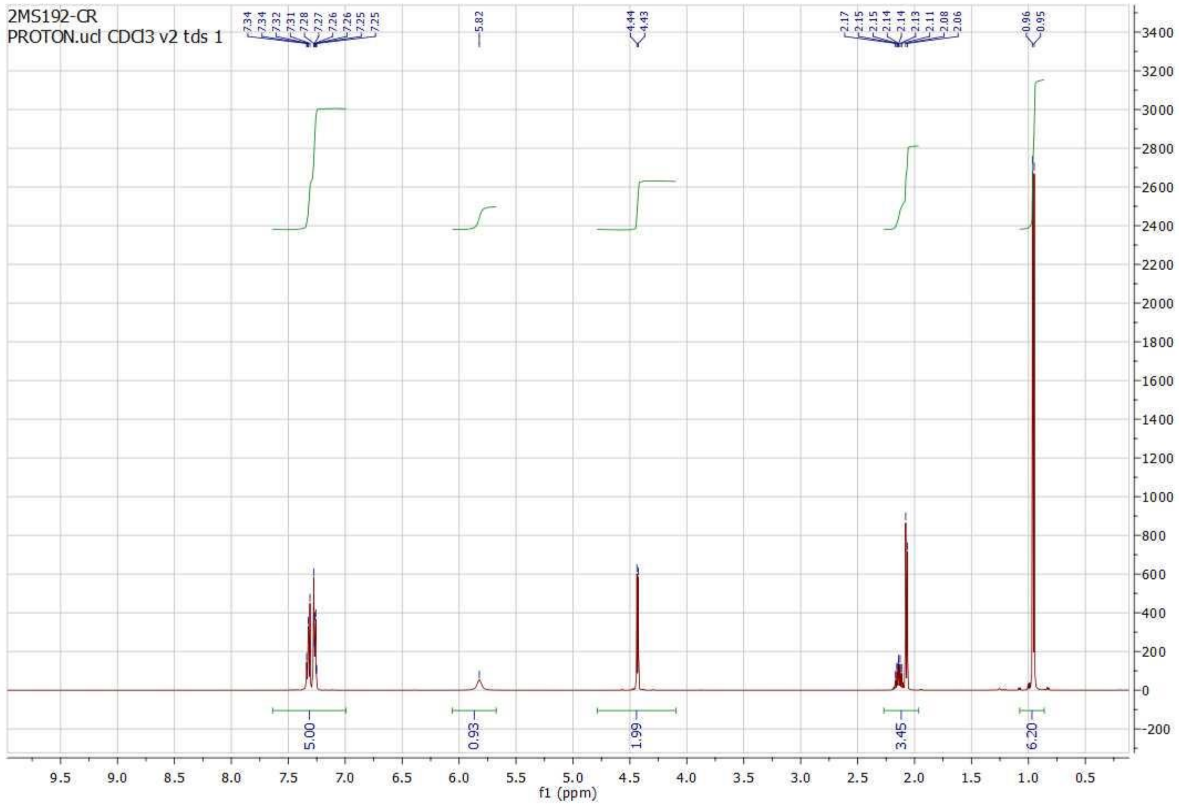

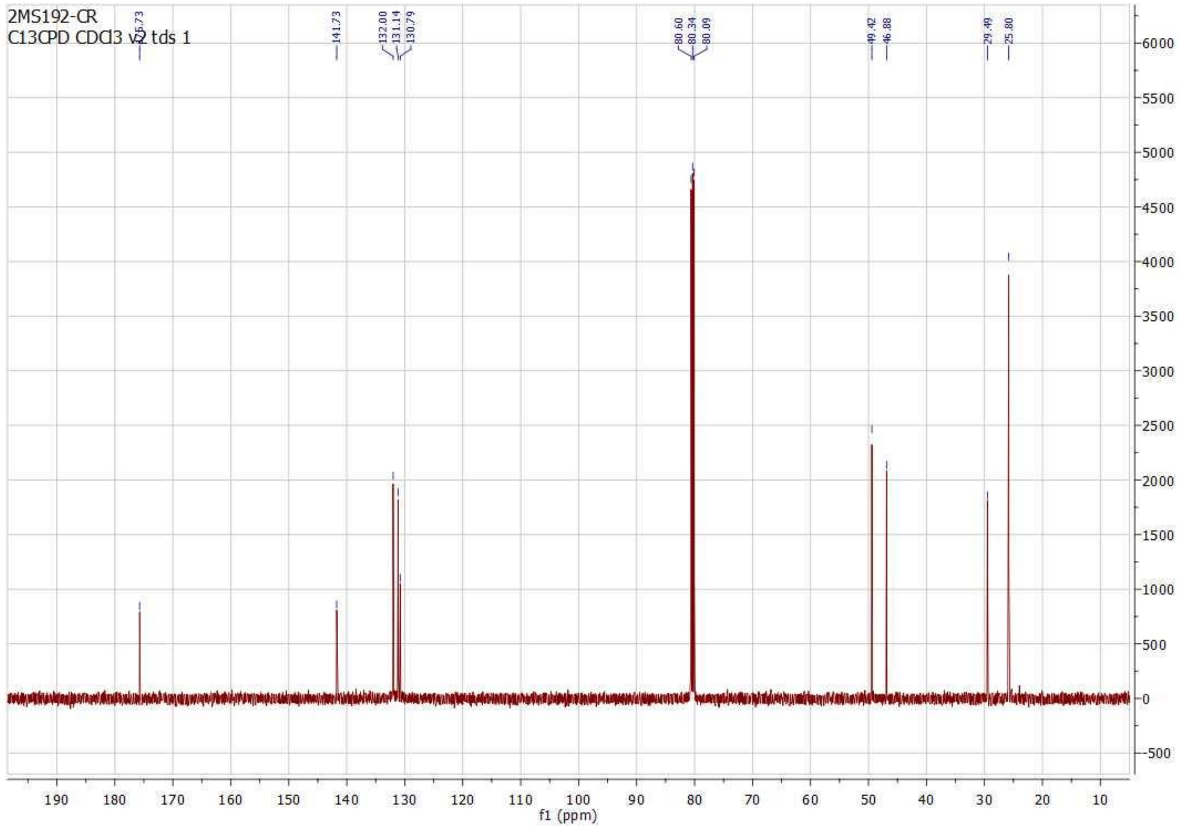

***N*-phenylbenzamide (4)**

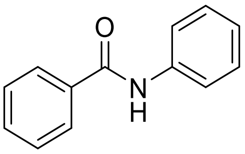

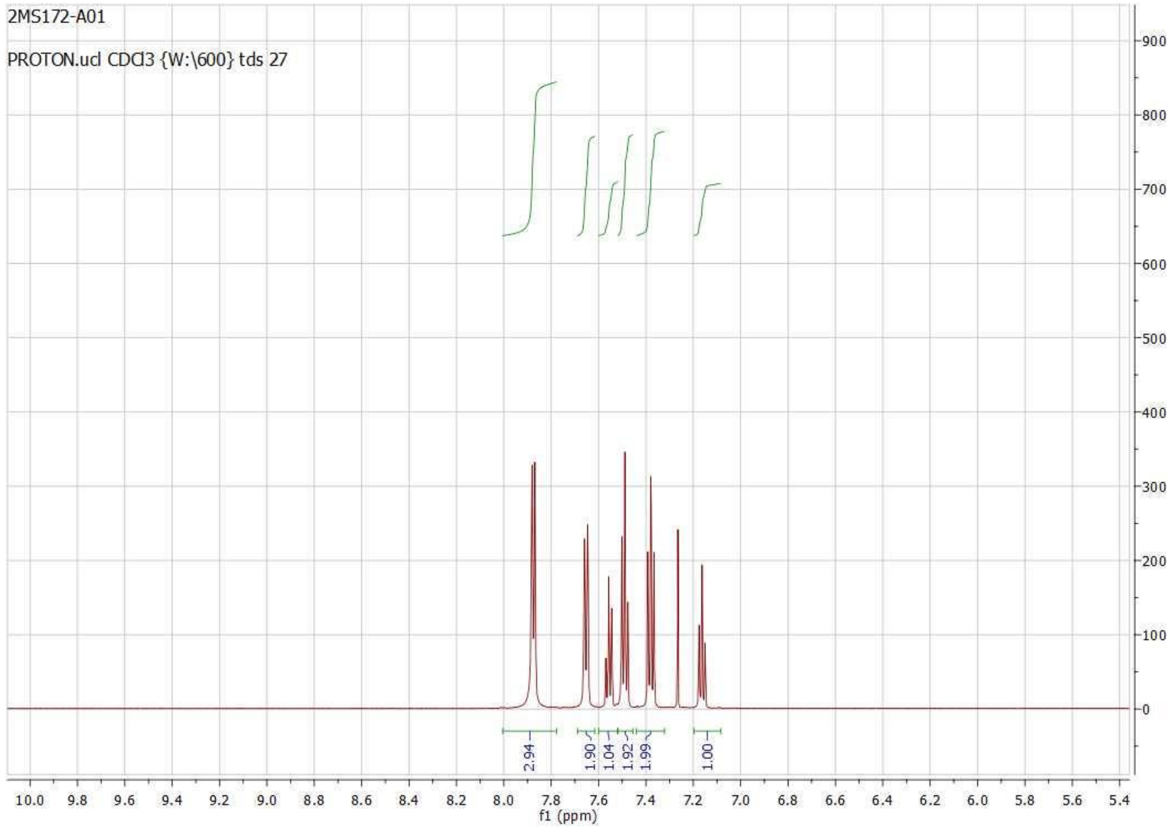

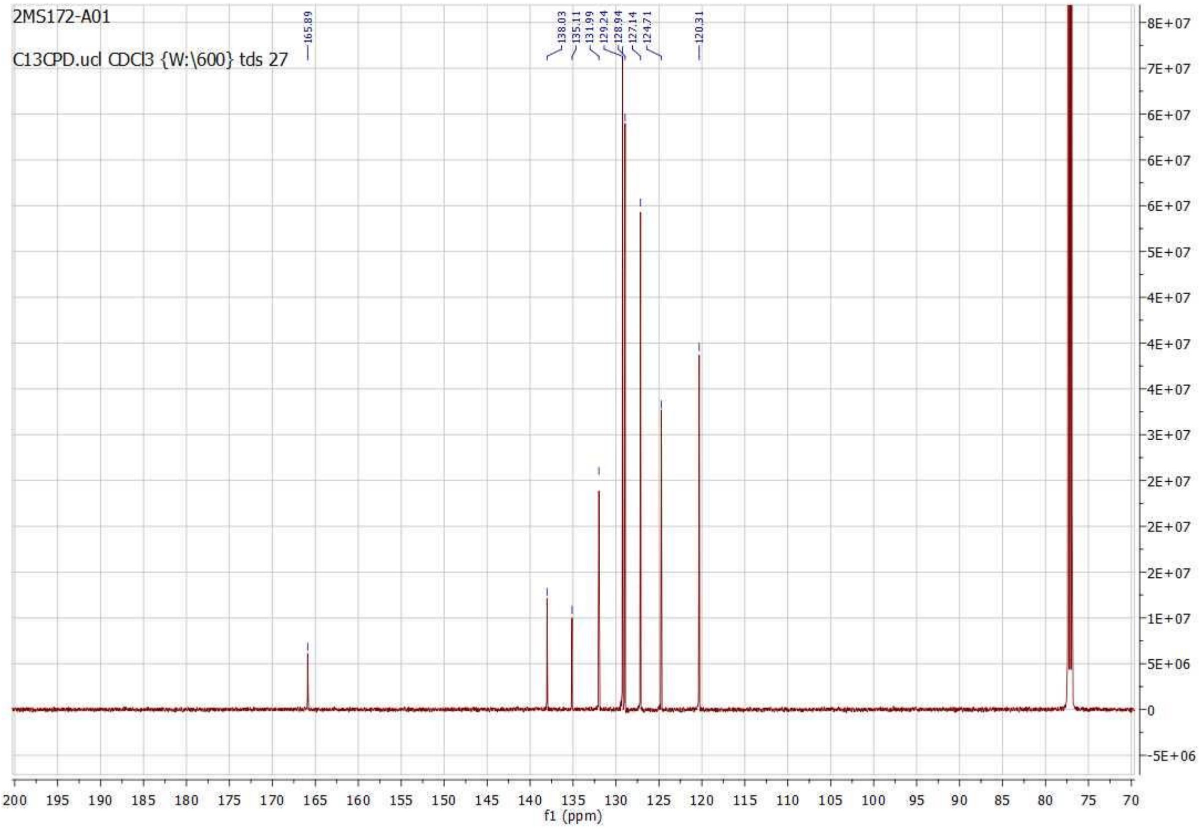

***N*,2-diphenylacetamide (5)**

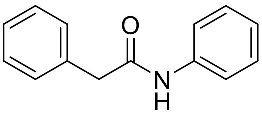

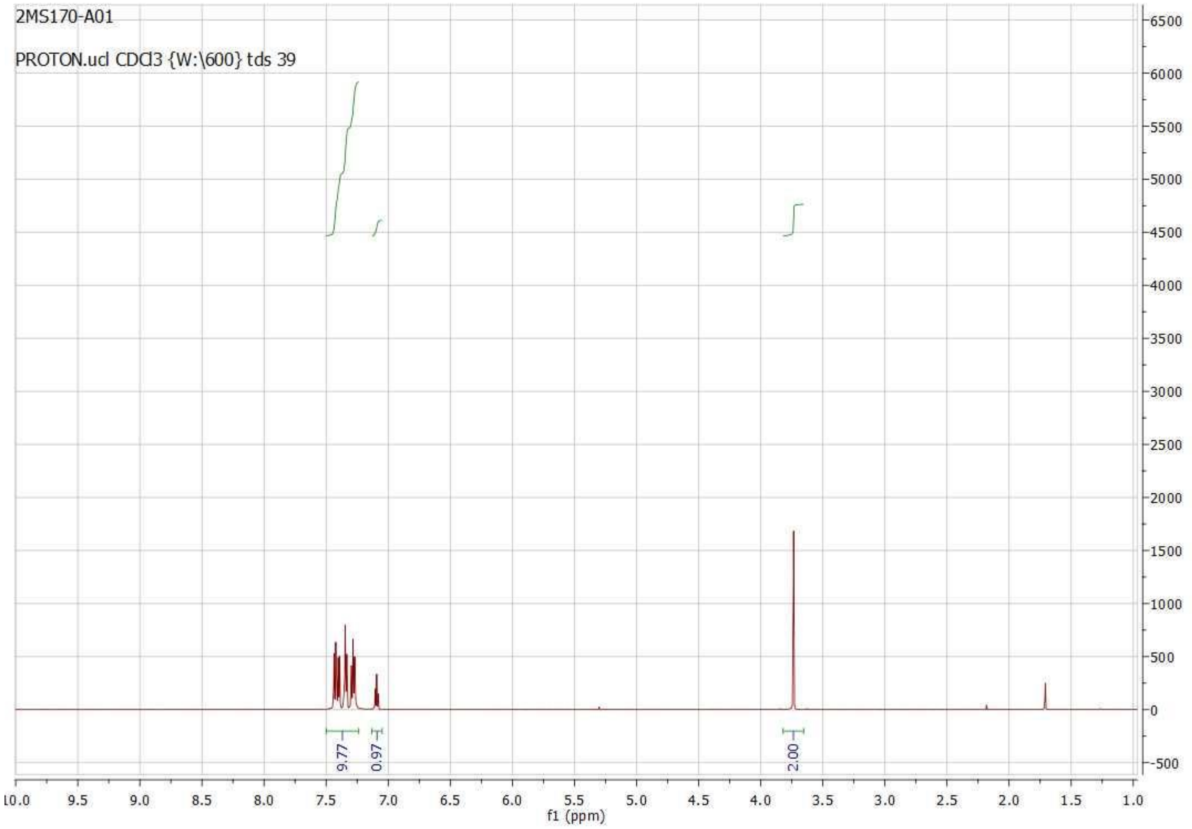

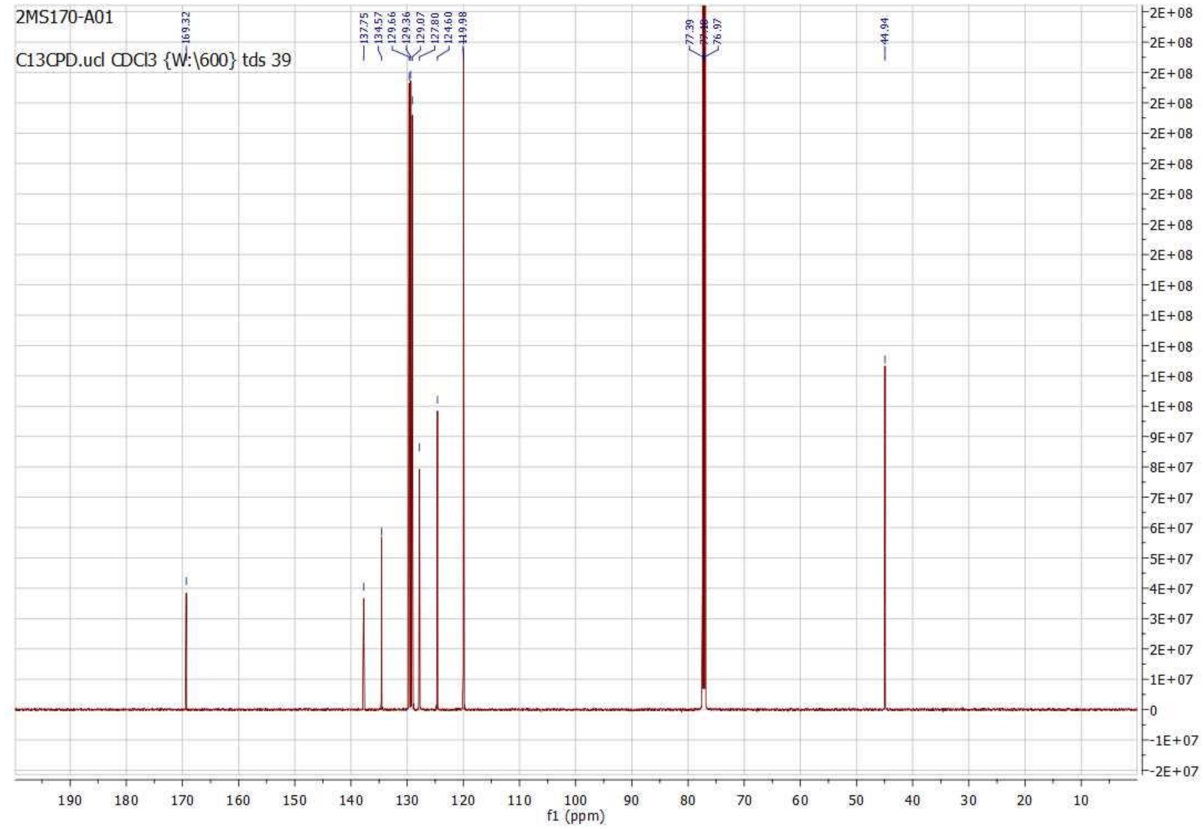

(*R*)-2-phenyl-N-(1-phenylethyl)acetamide (6)

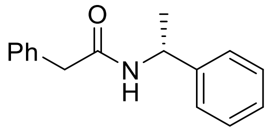

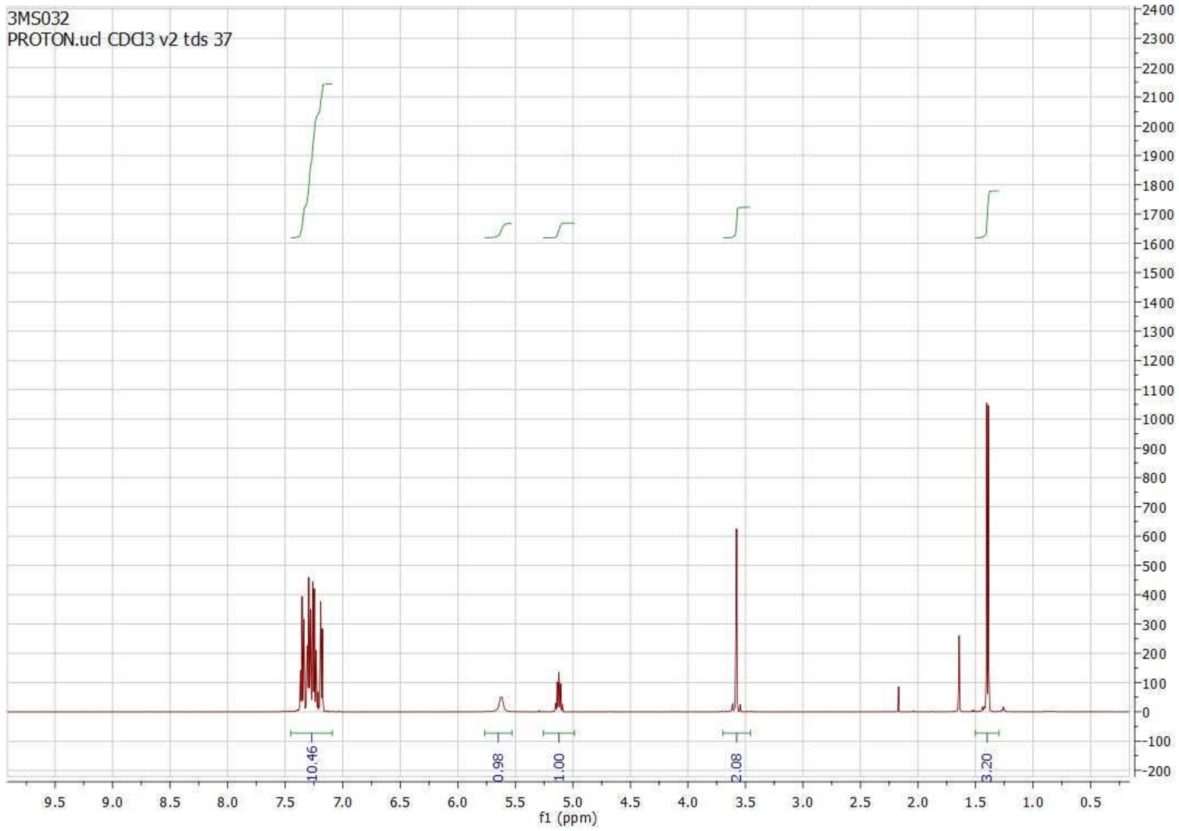

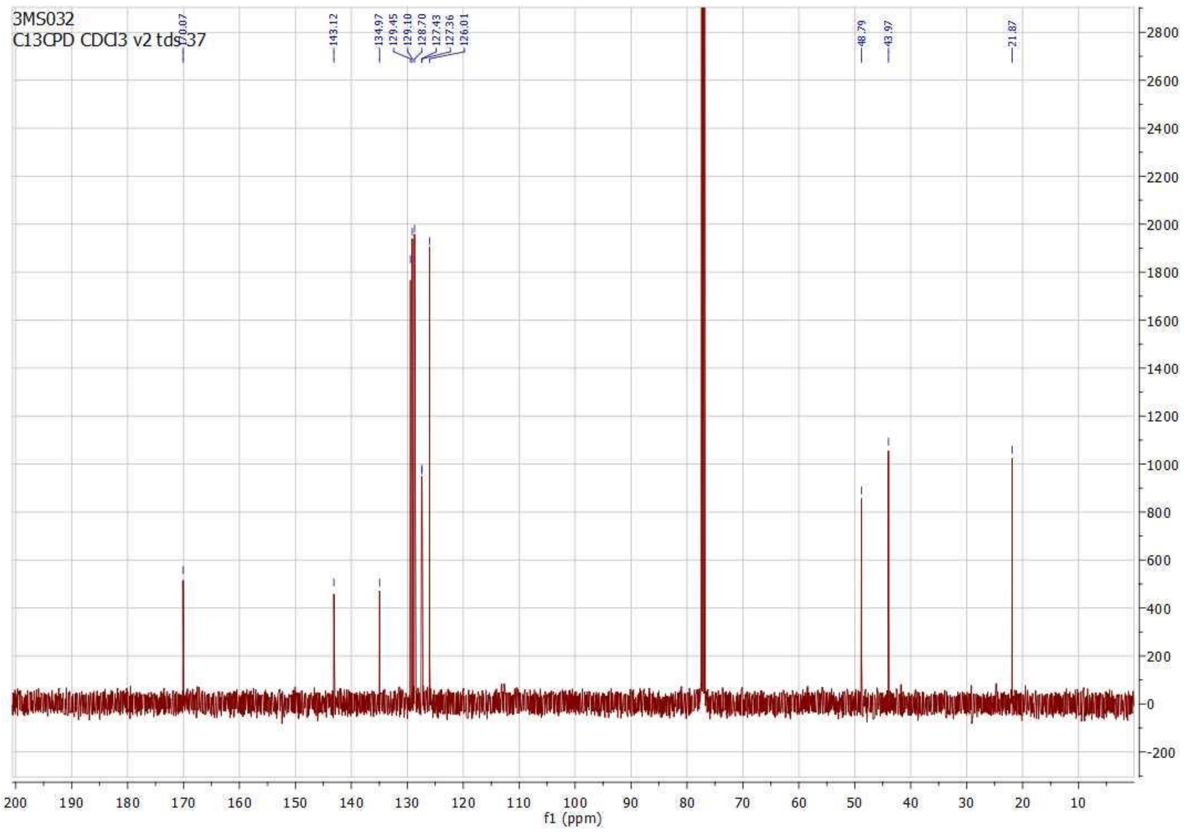

***N*-(2-methylbenzyl)-2-phenylacetamide (7)**

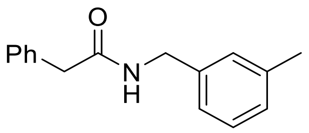

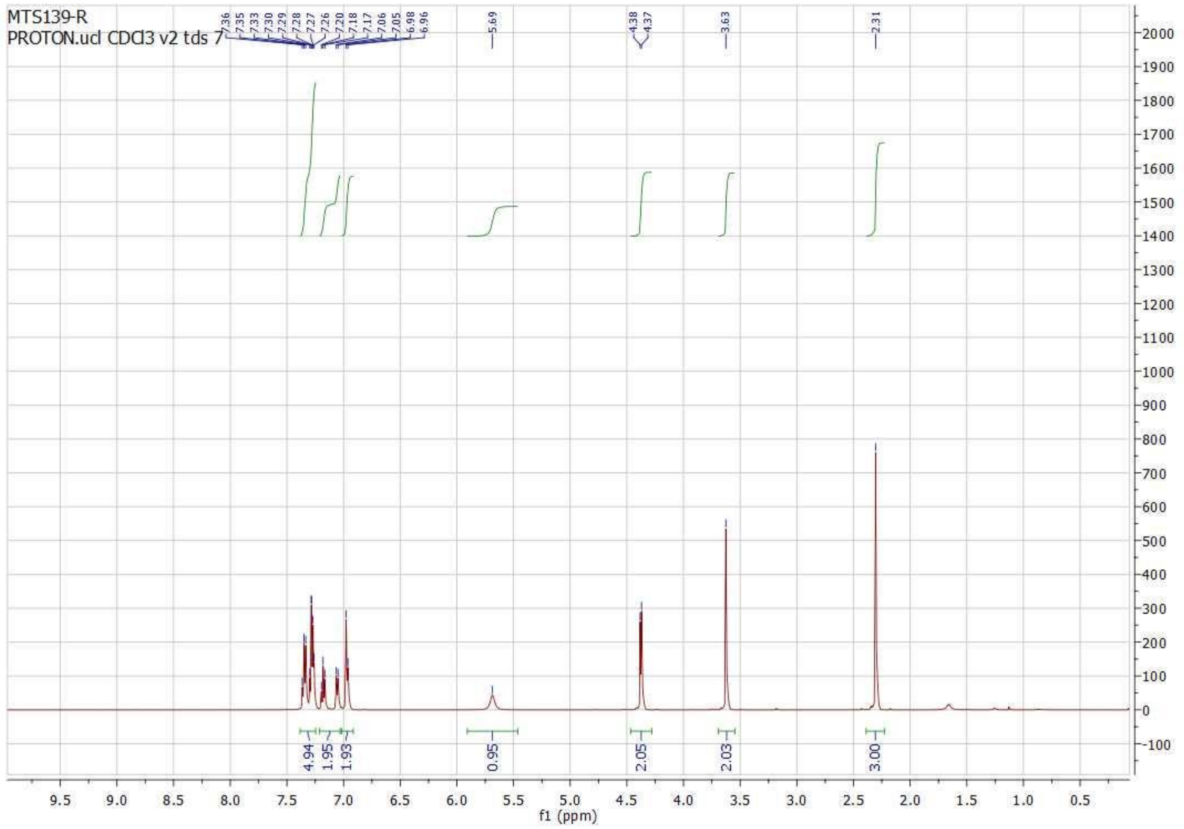

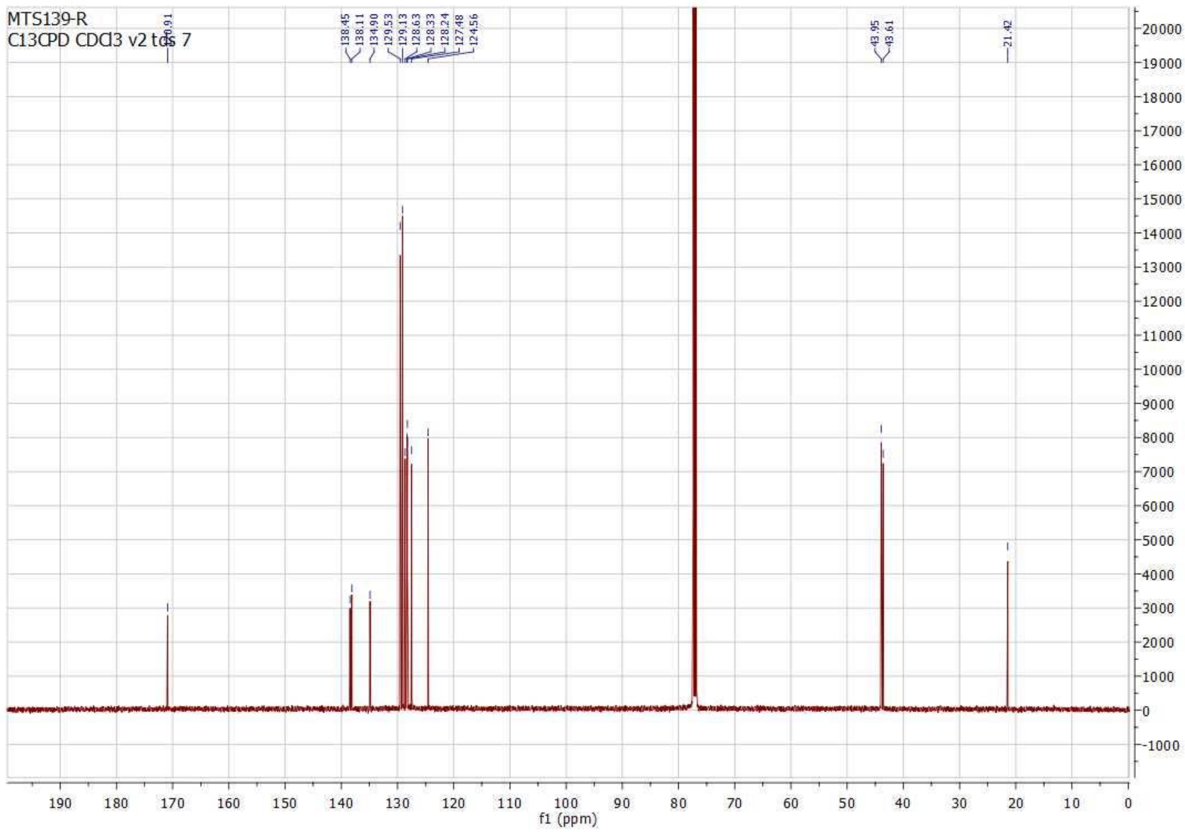

N-benzyl-2-(3-fluorophenyl)acetamide (8)

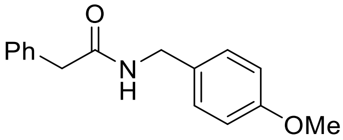

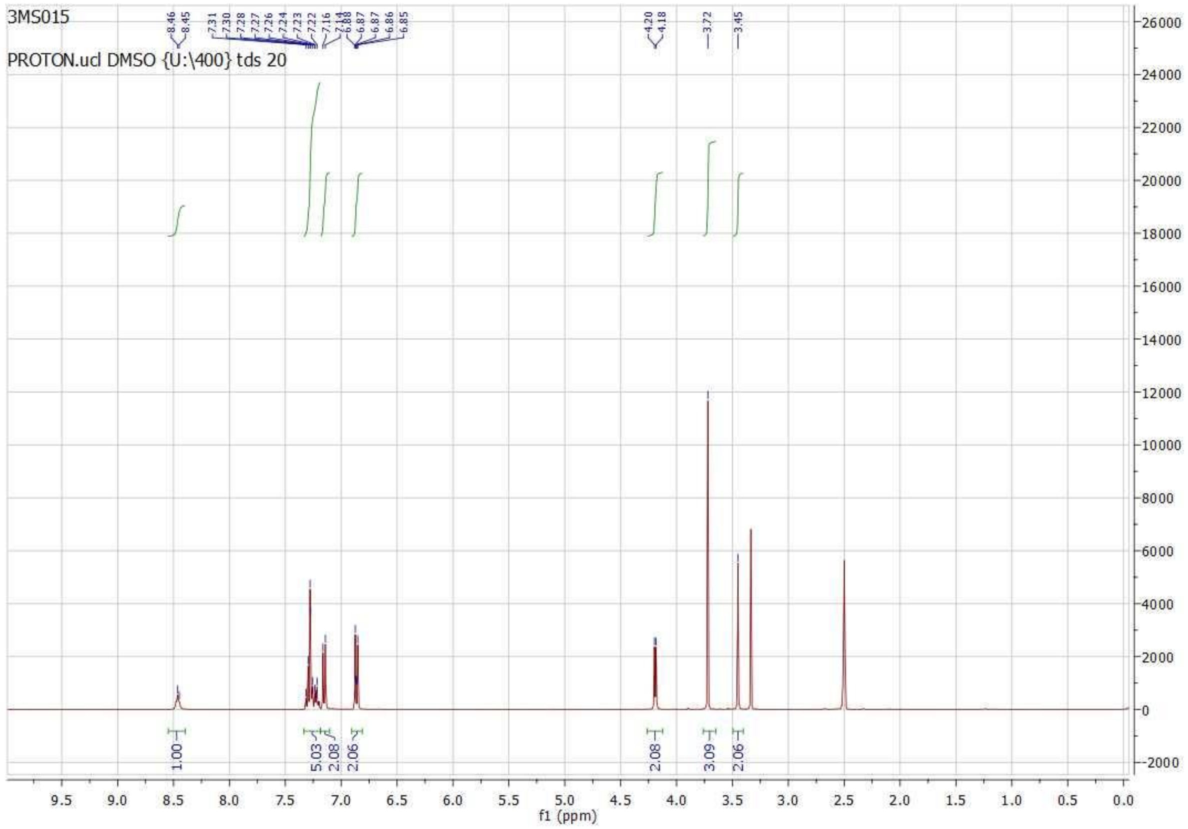

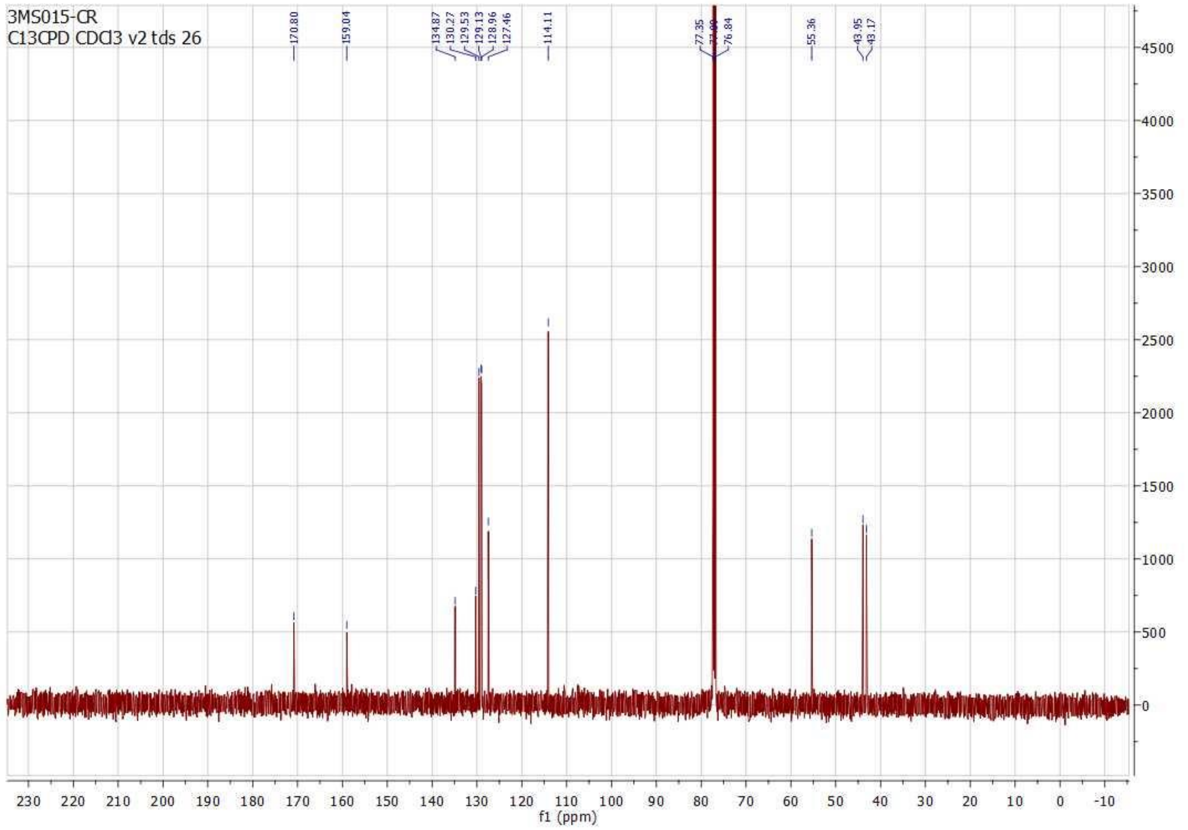

_N_-(4-chlorobenzyl)-2-phenylacetamide (9):

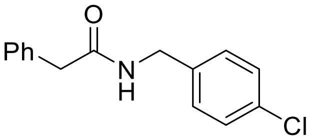

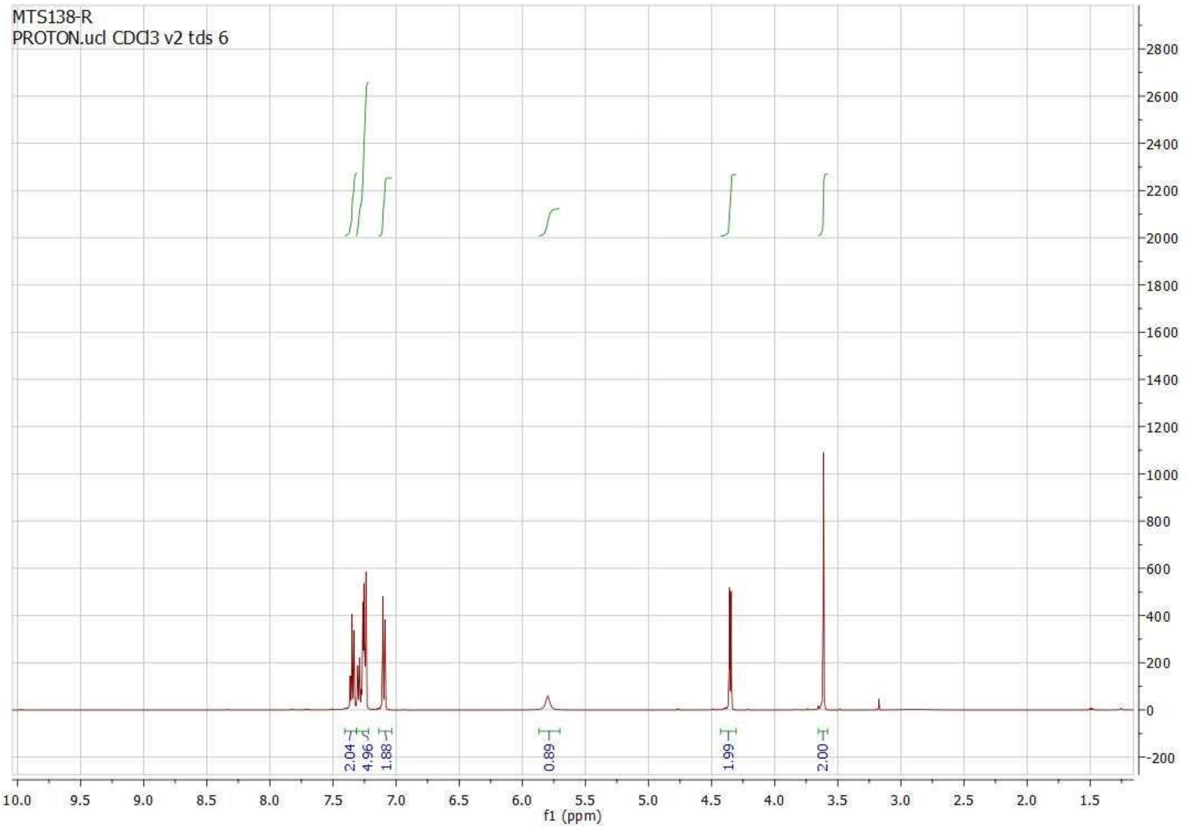

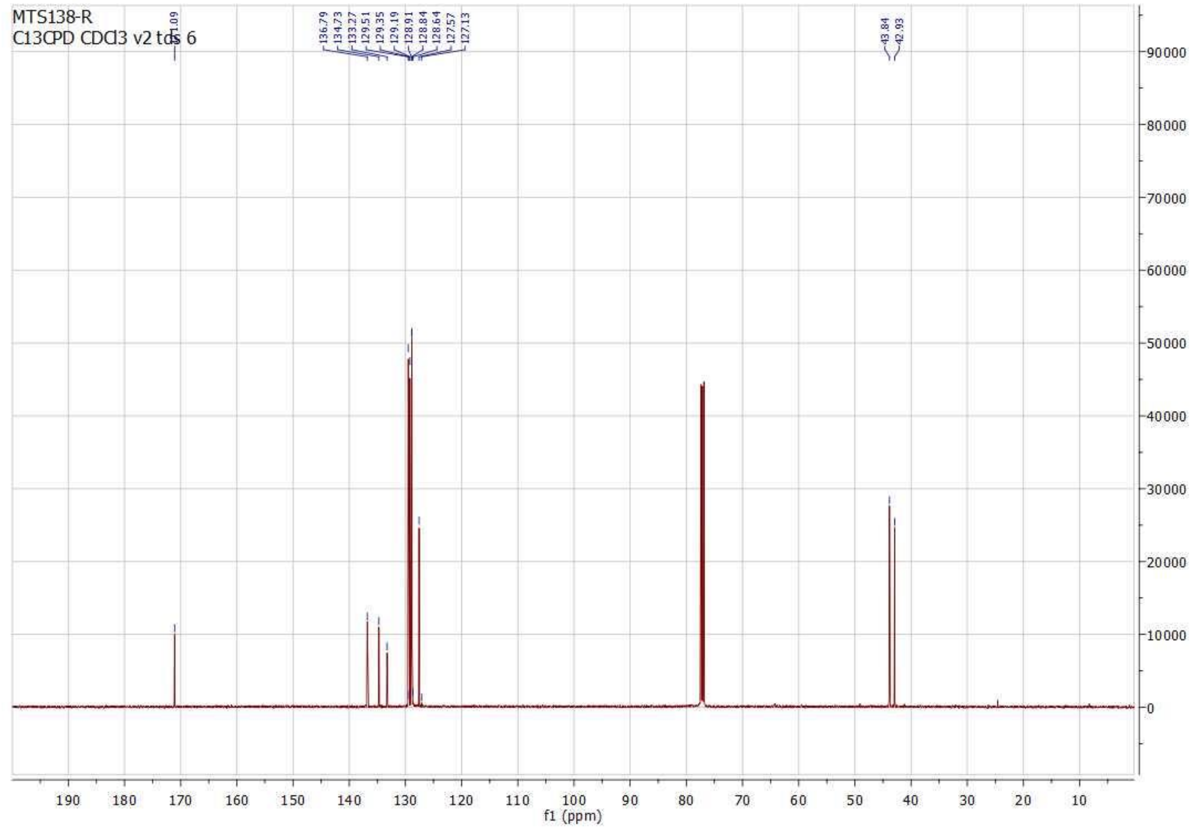

***N*-benzyl-2-phenylacetamide (10)**

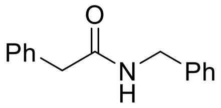

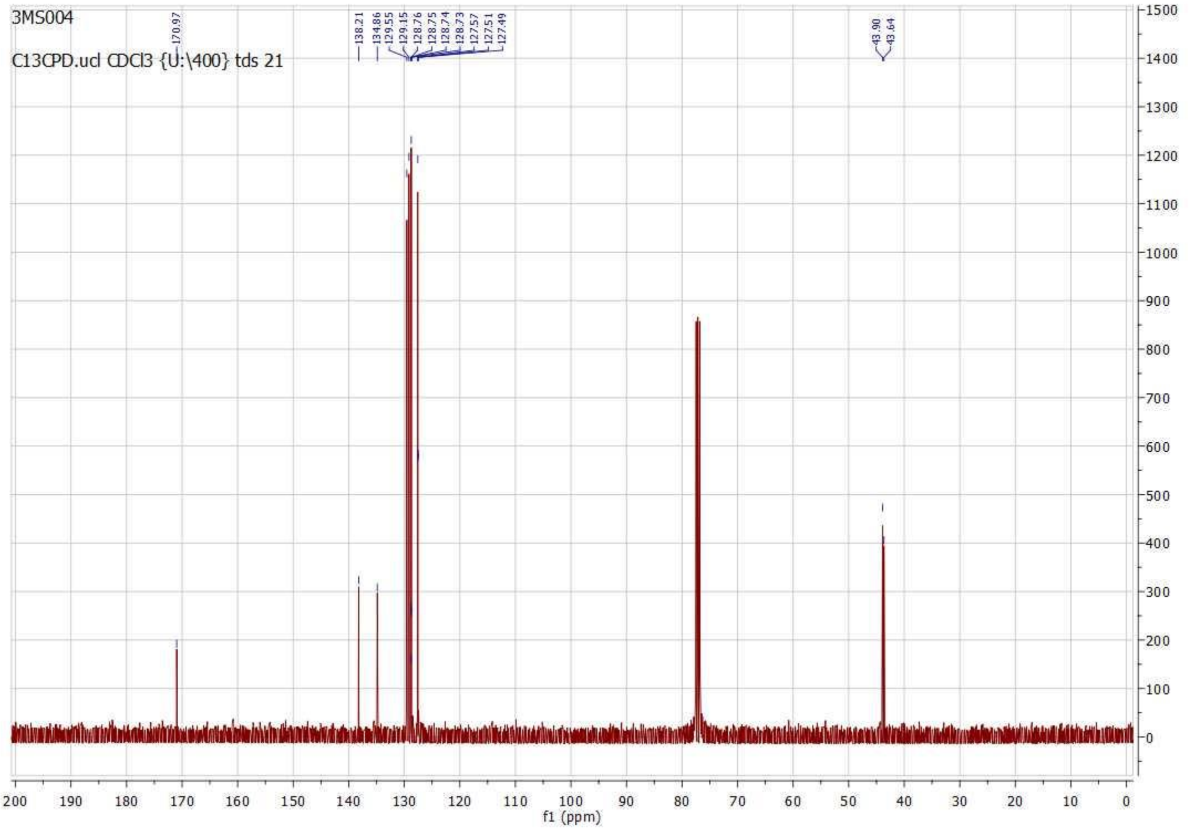

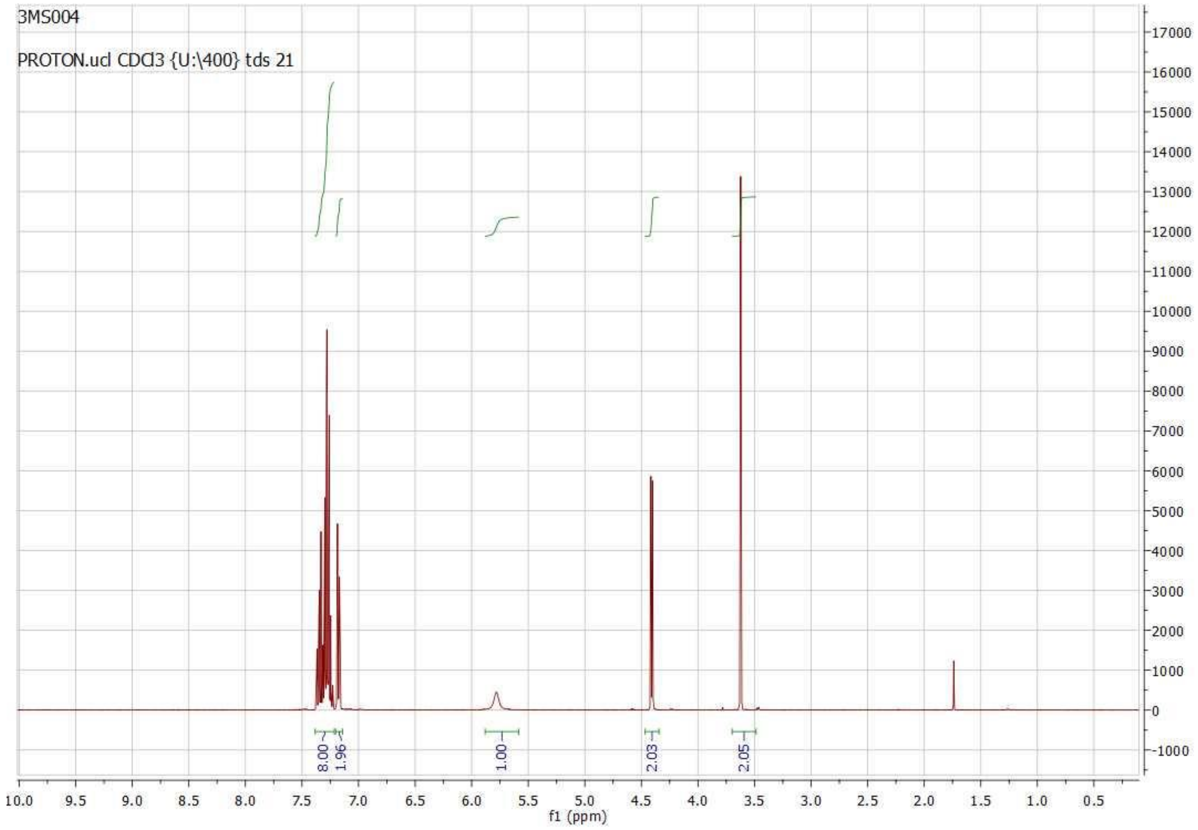

***N*-benzyl-2-(3-fluorophenyl)acetamide (11)**

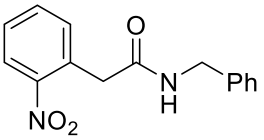

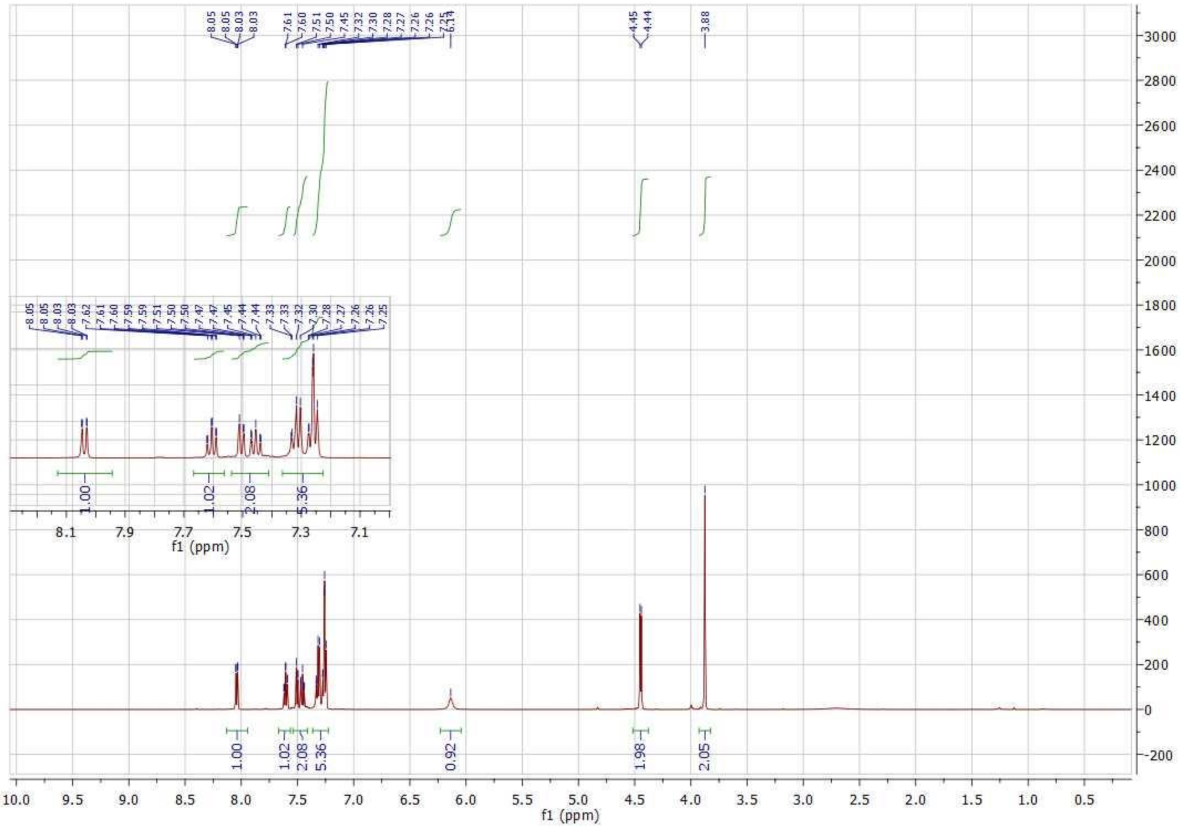

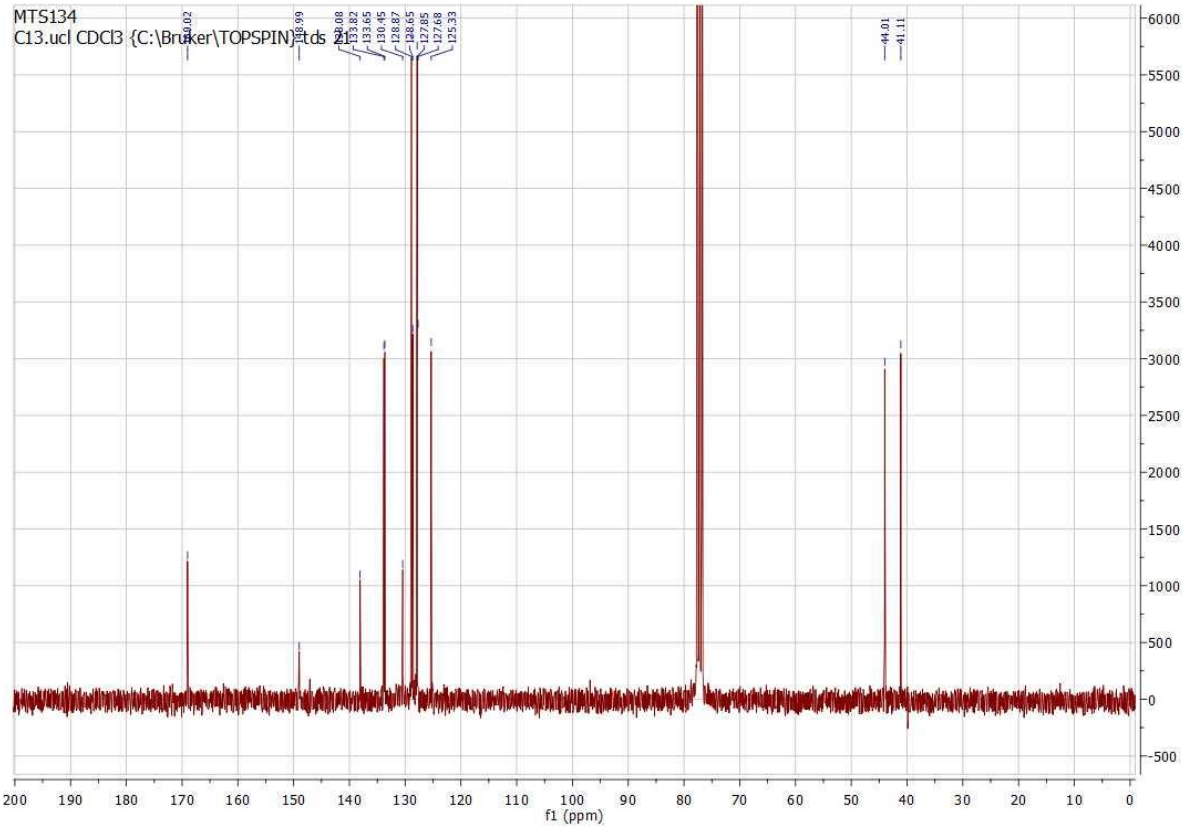

***N*-benzyl-2-(2-methoxyphenyl)acetamide (12)**

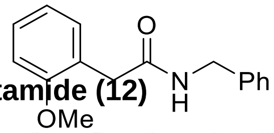

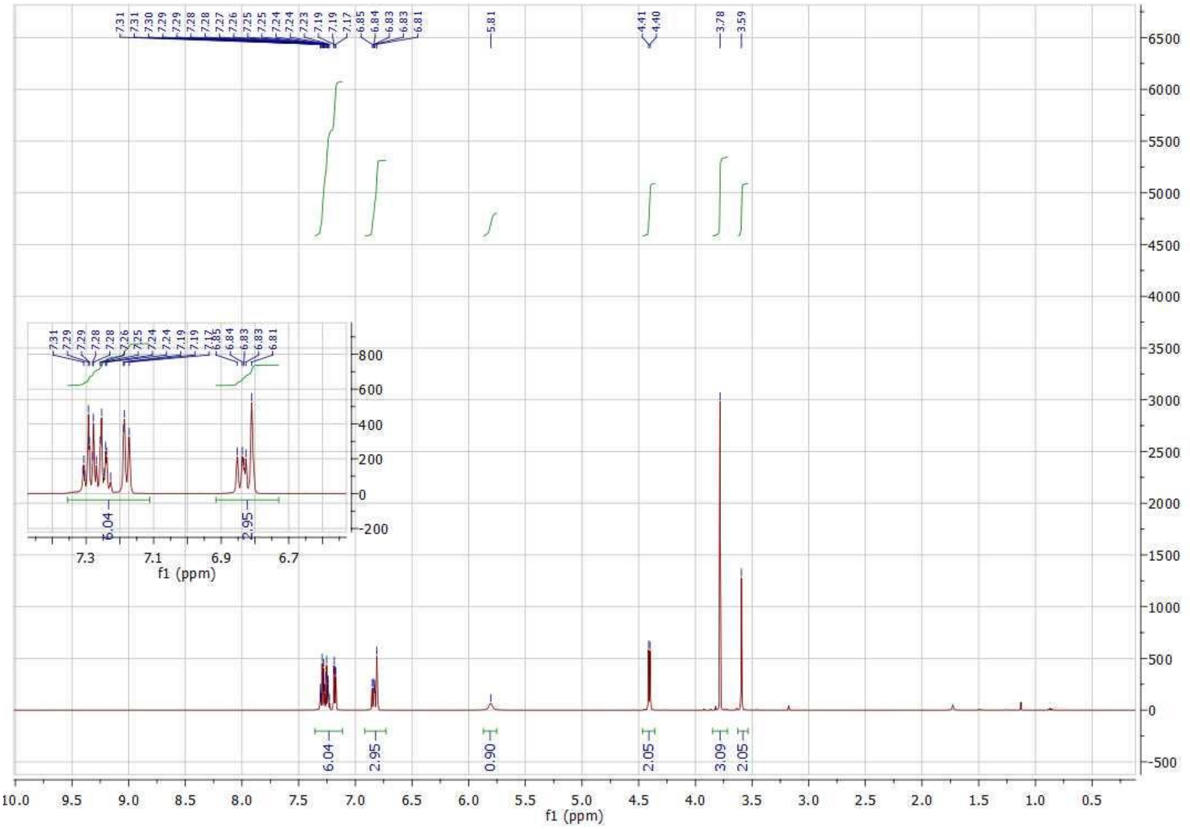

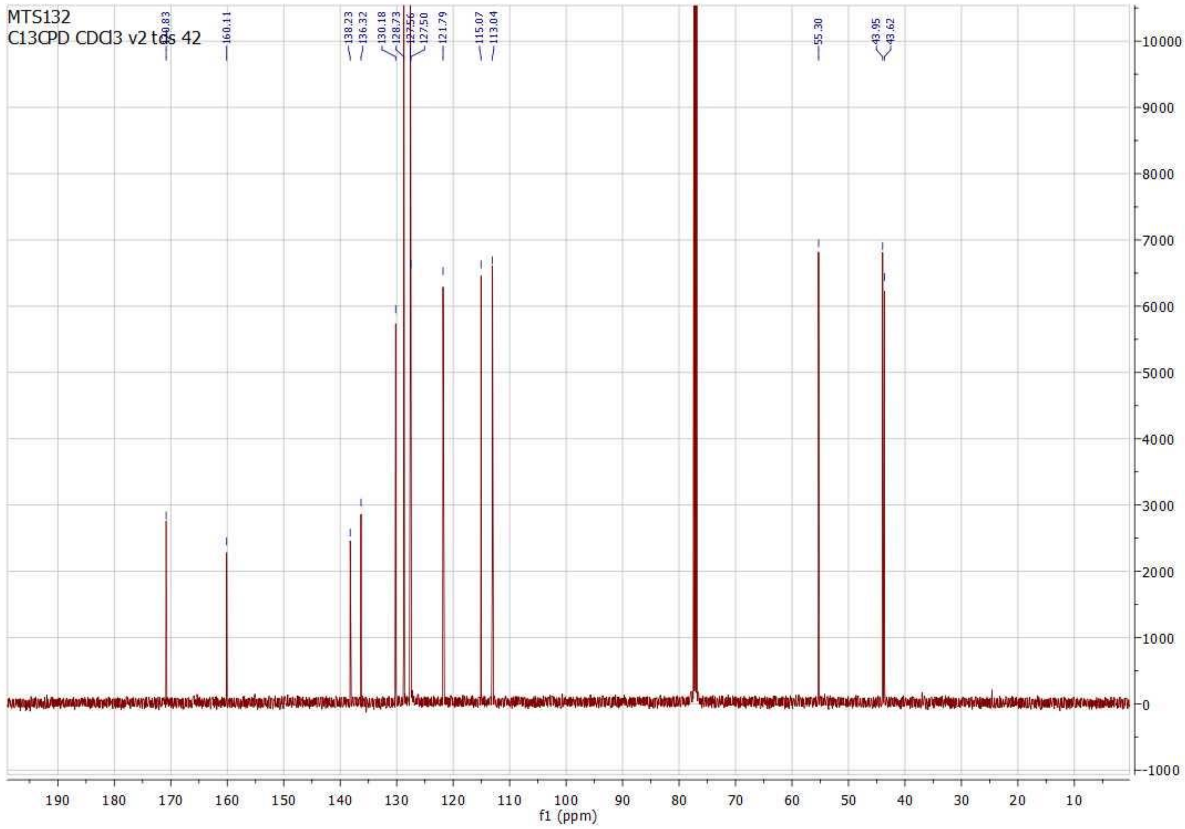

_N_-benzyl-2-(3-fluorophenyl)acetamide (13)

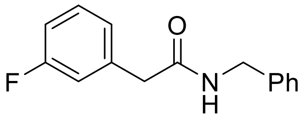

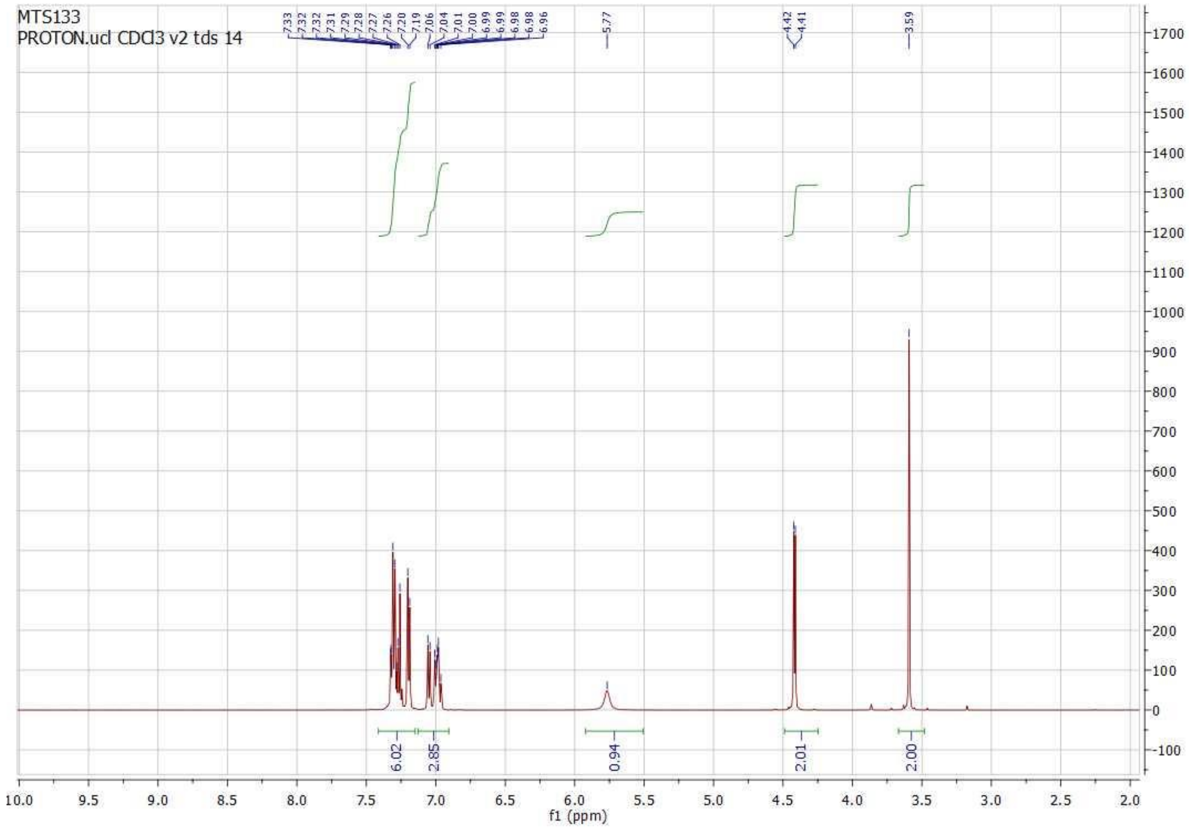

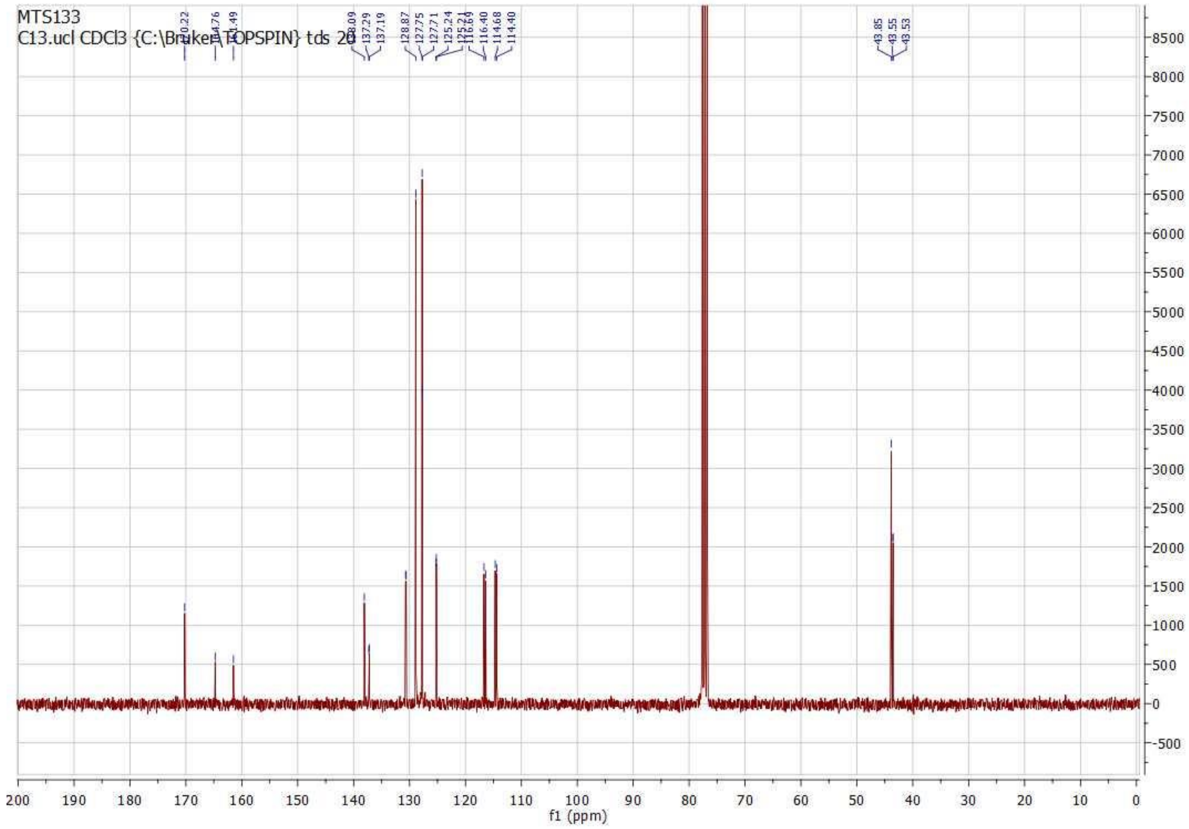

1-(3,4-dihydroisoquinolin-2(1H)-yl)-2-phenylethan-1-one (14)

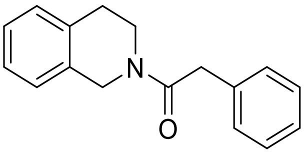

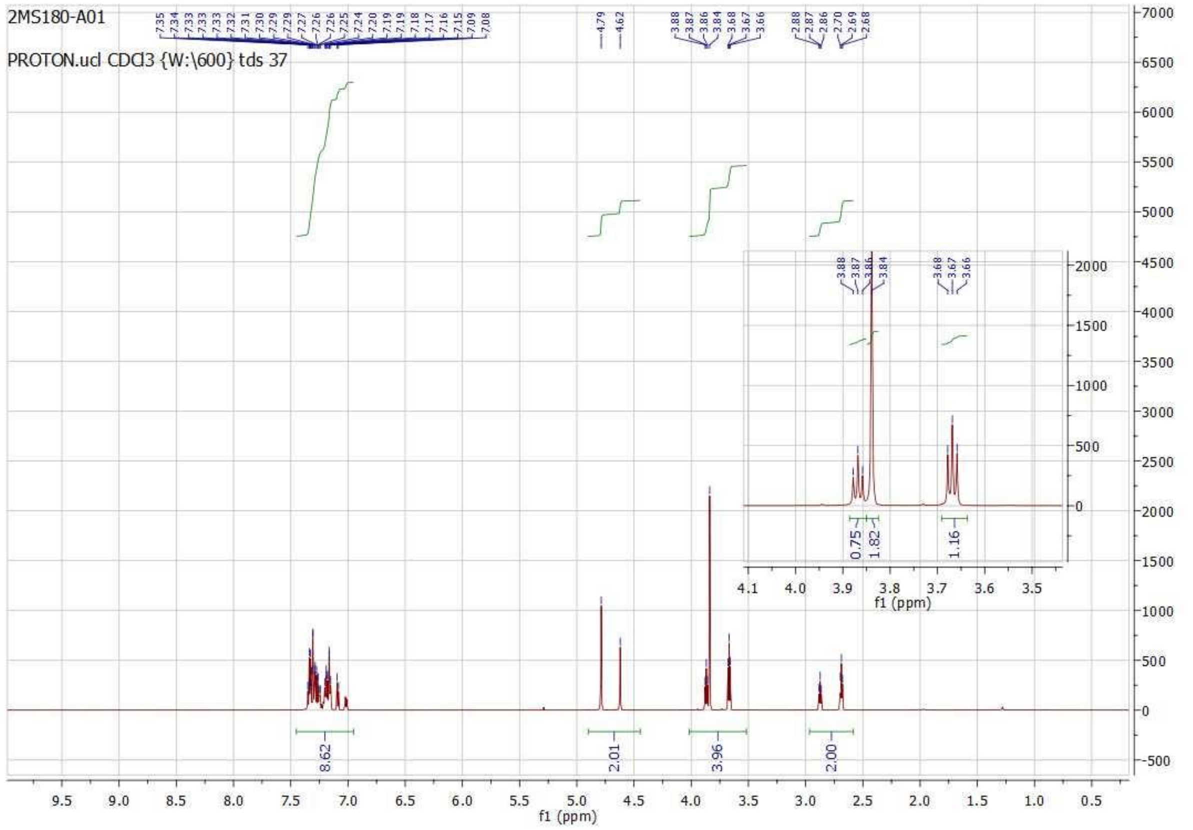

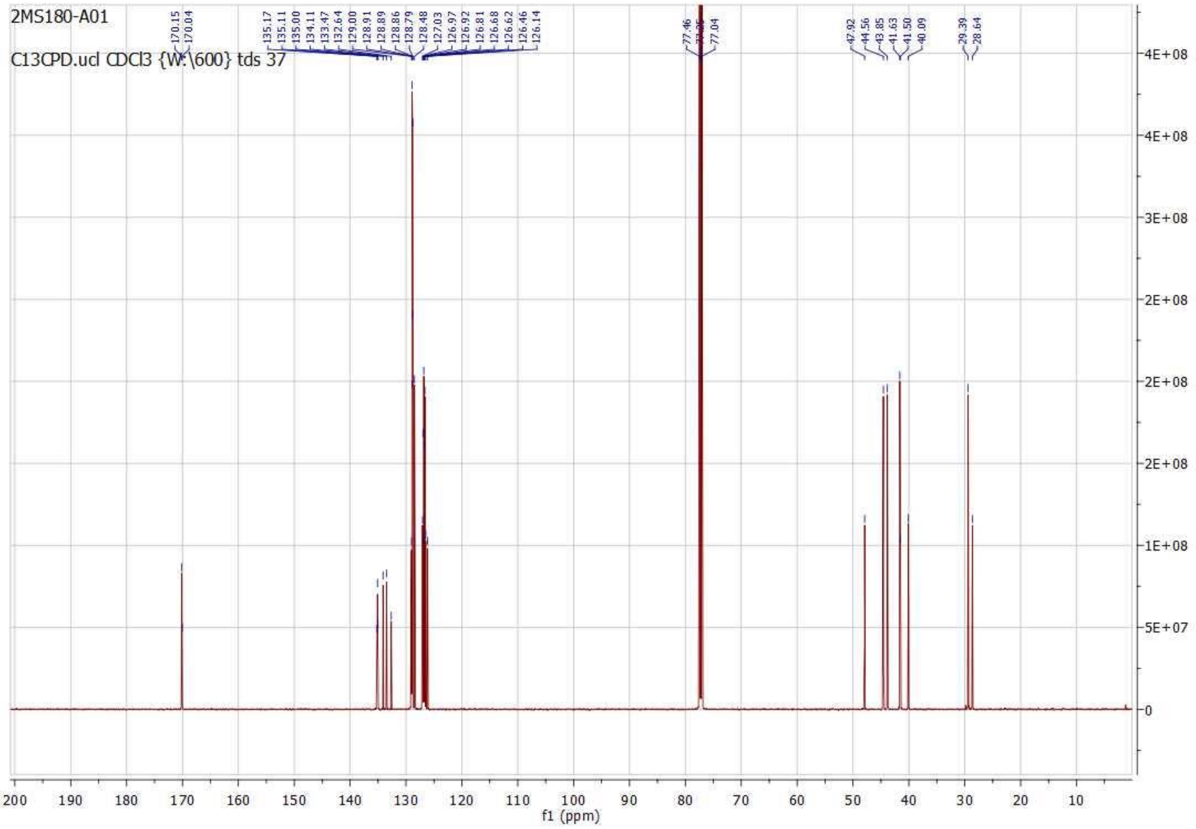

1-morpholino-2-phenylethan-1-one (15)

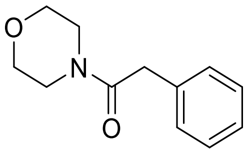

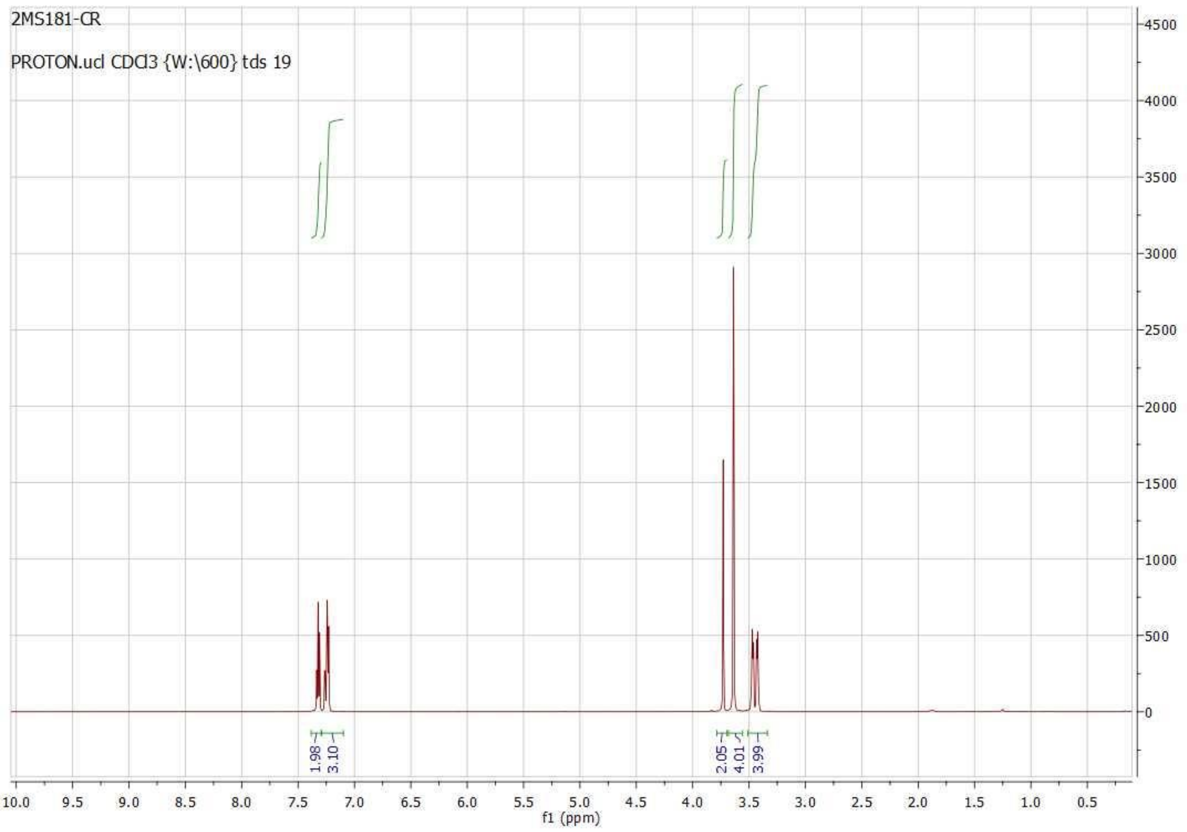

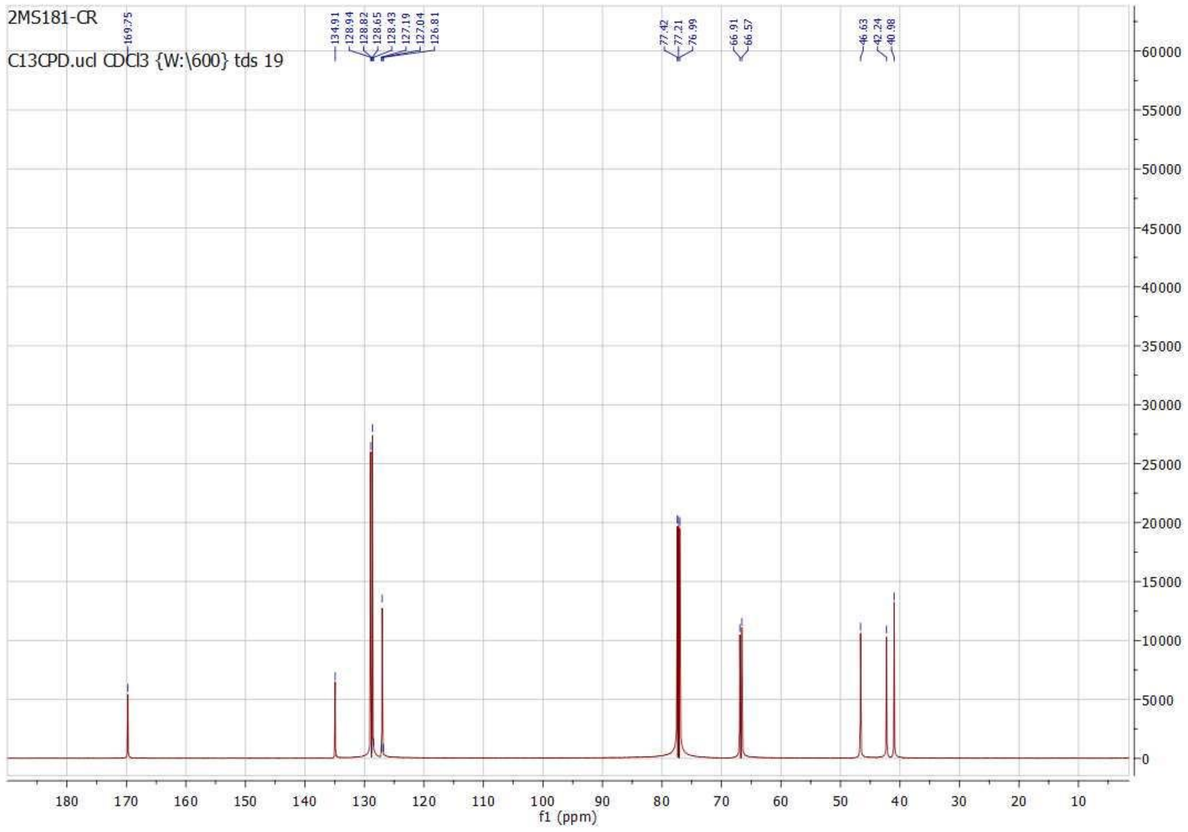

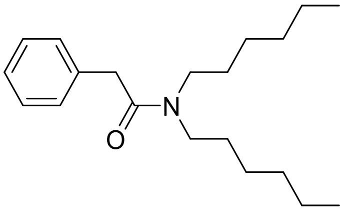

***N,N*-dihexyl-2-phenylacetamide (16)**

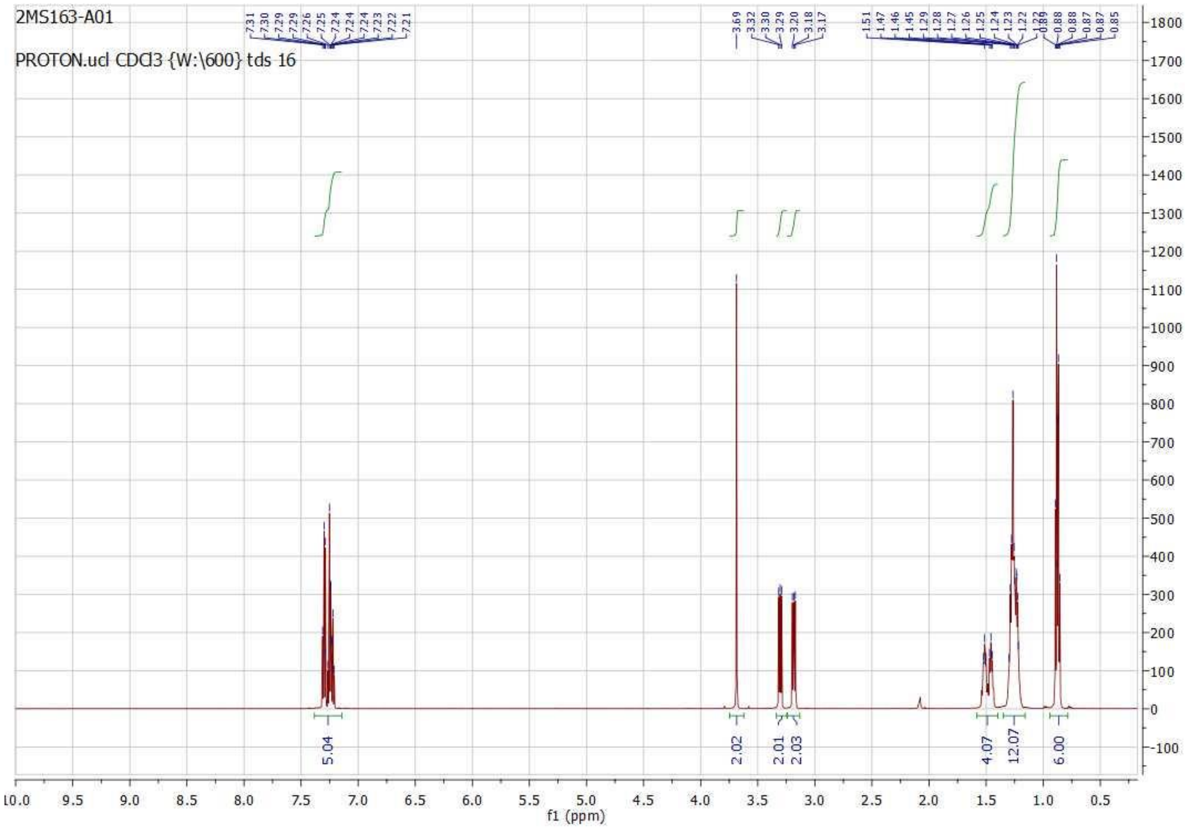

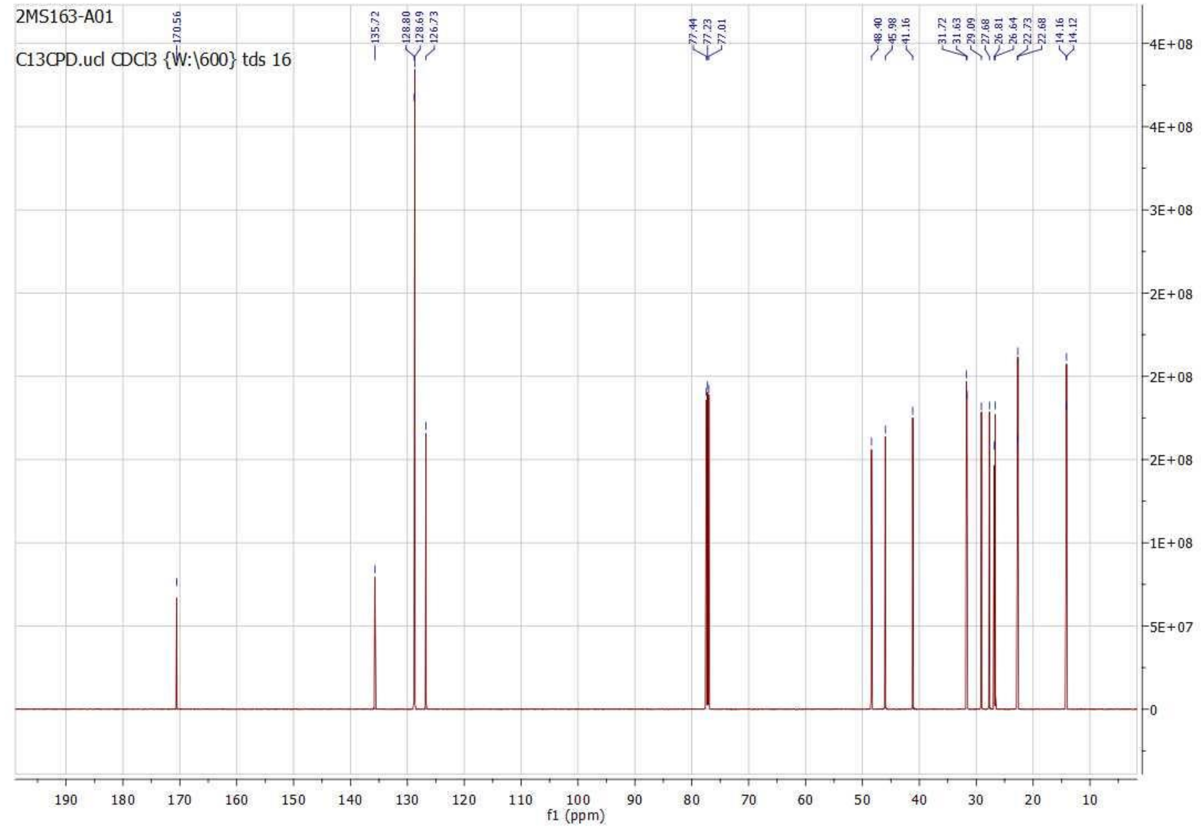

***N,N*-dioctyl-2-phenylacetamide (17)**

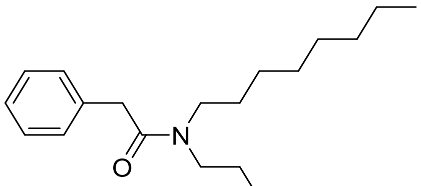

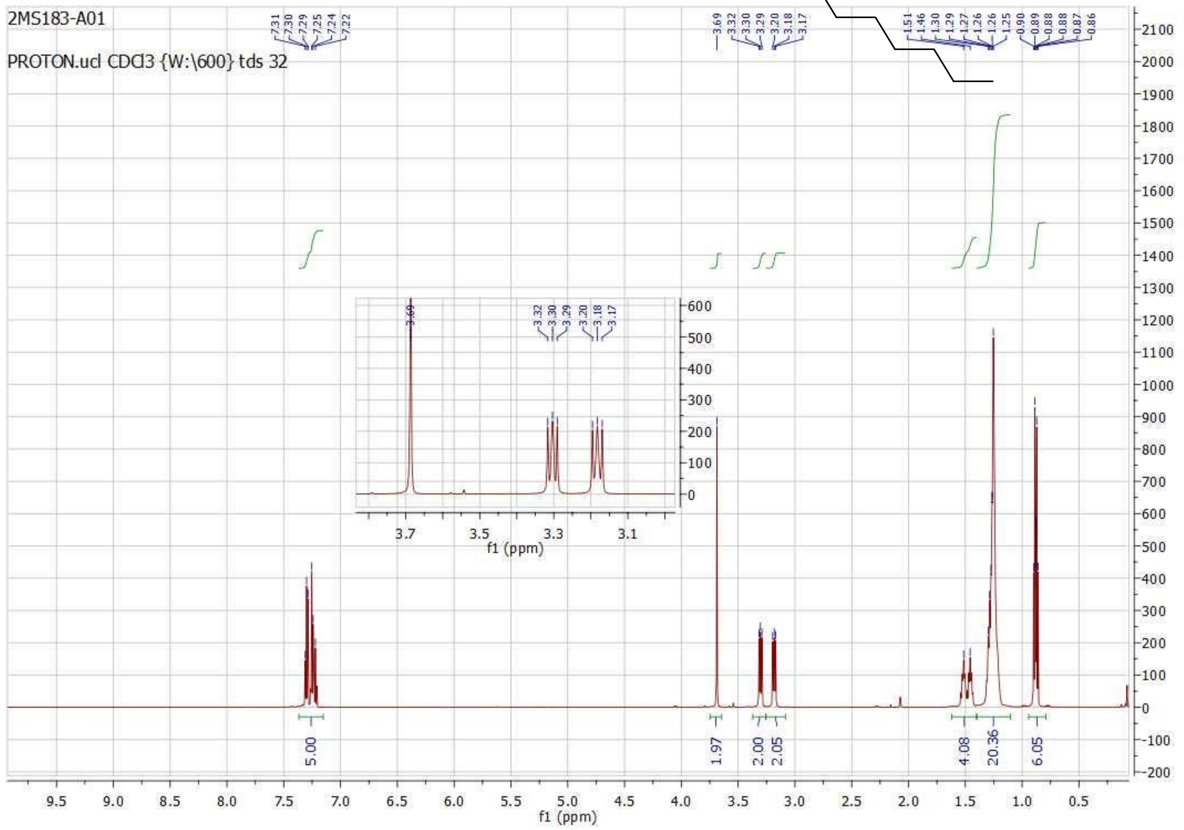

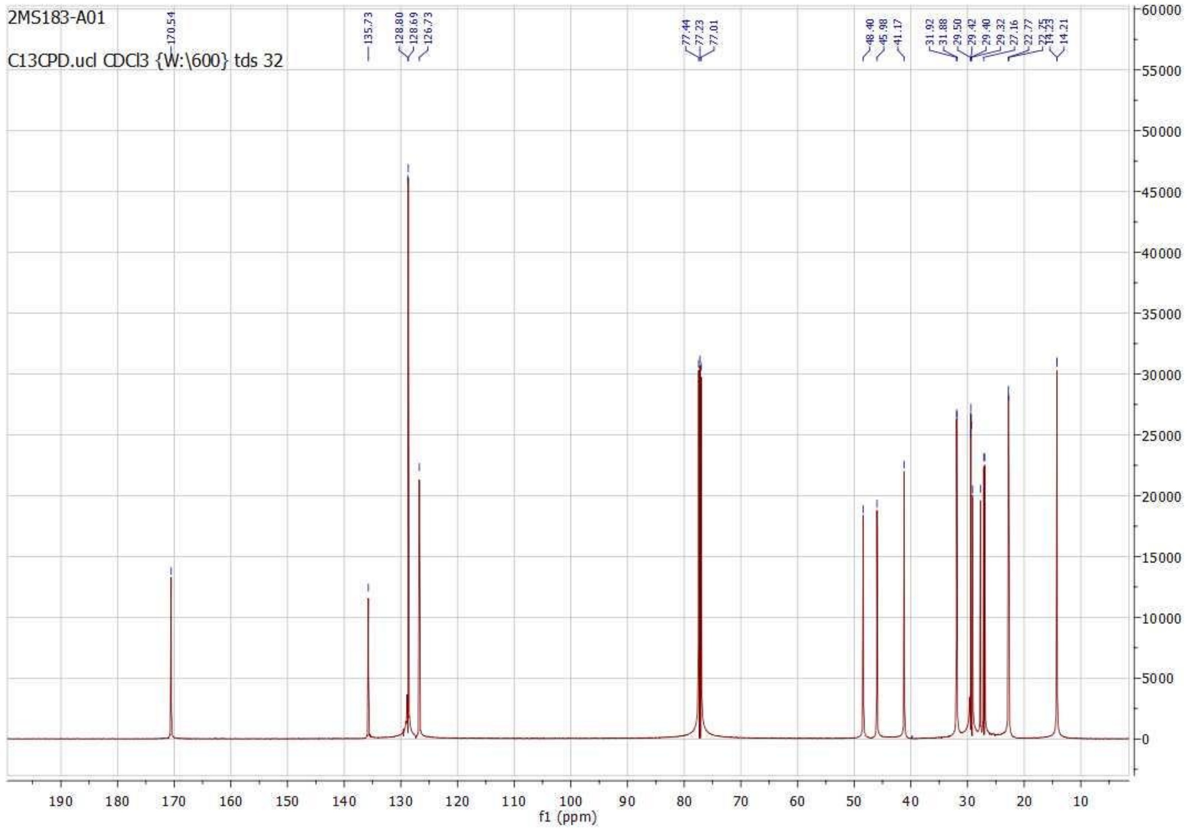

N-benzyl-N-methyl-2-phenylacetamide (18)

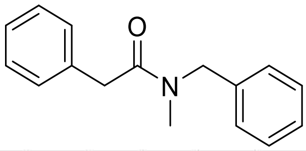

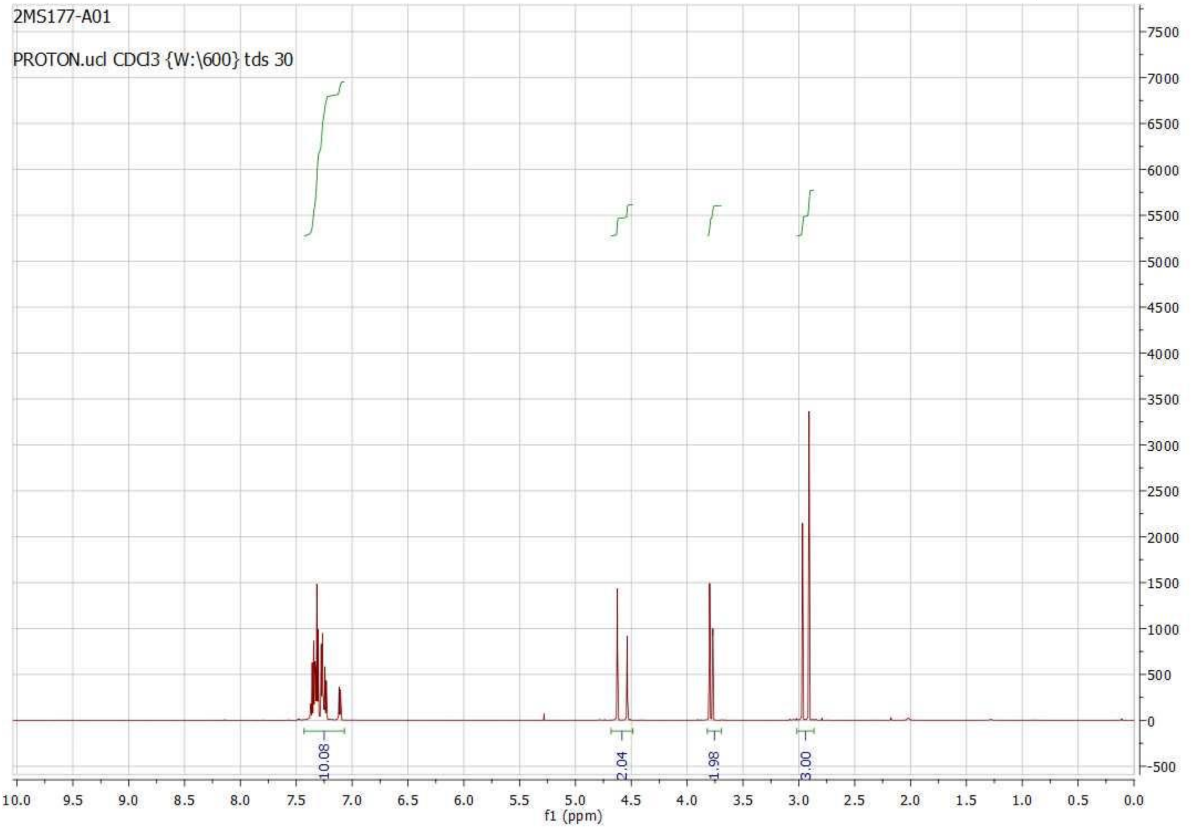

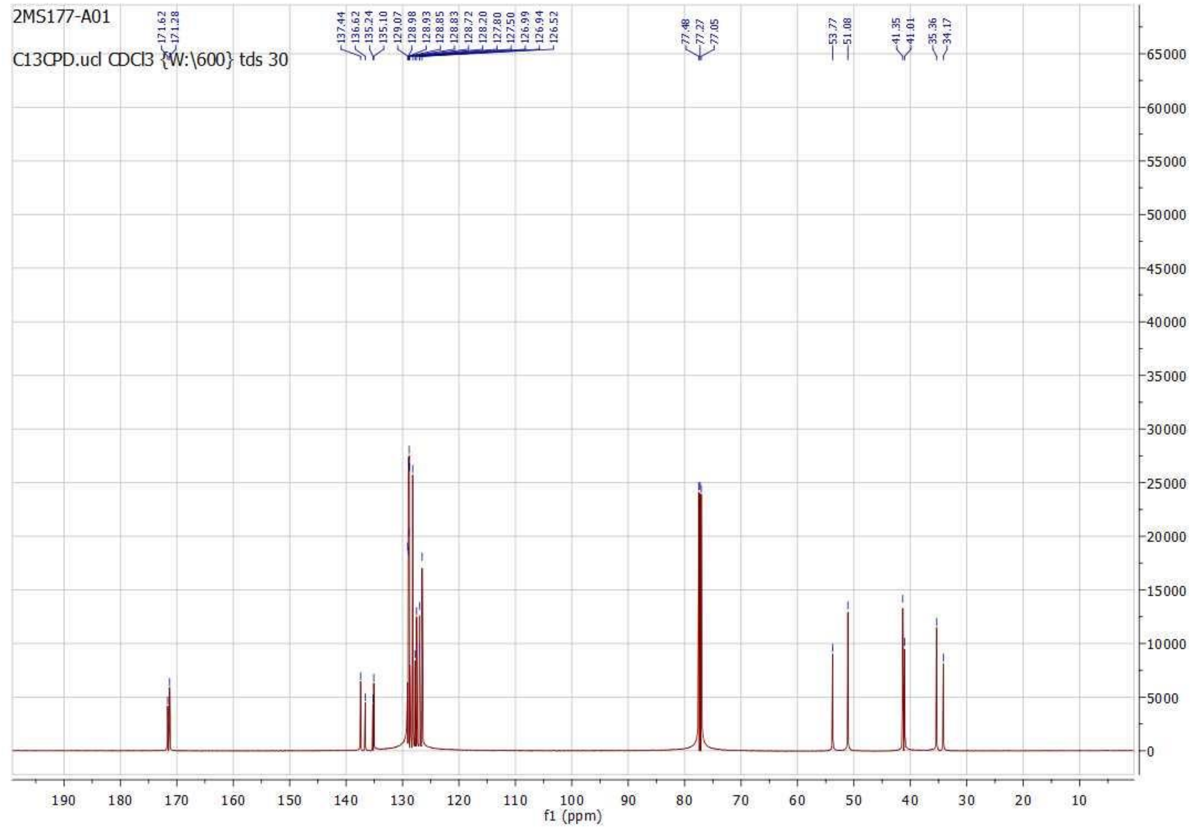

N-allyl-N,2-diphenylacetamide (19)

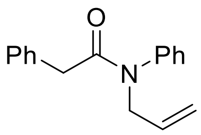

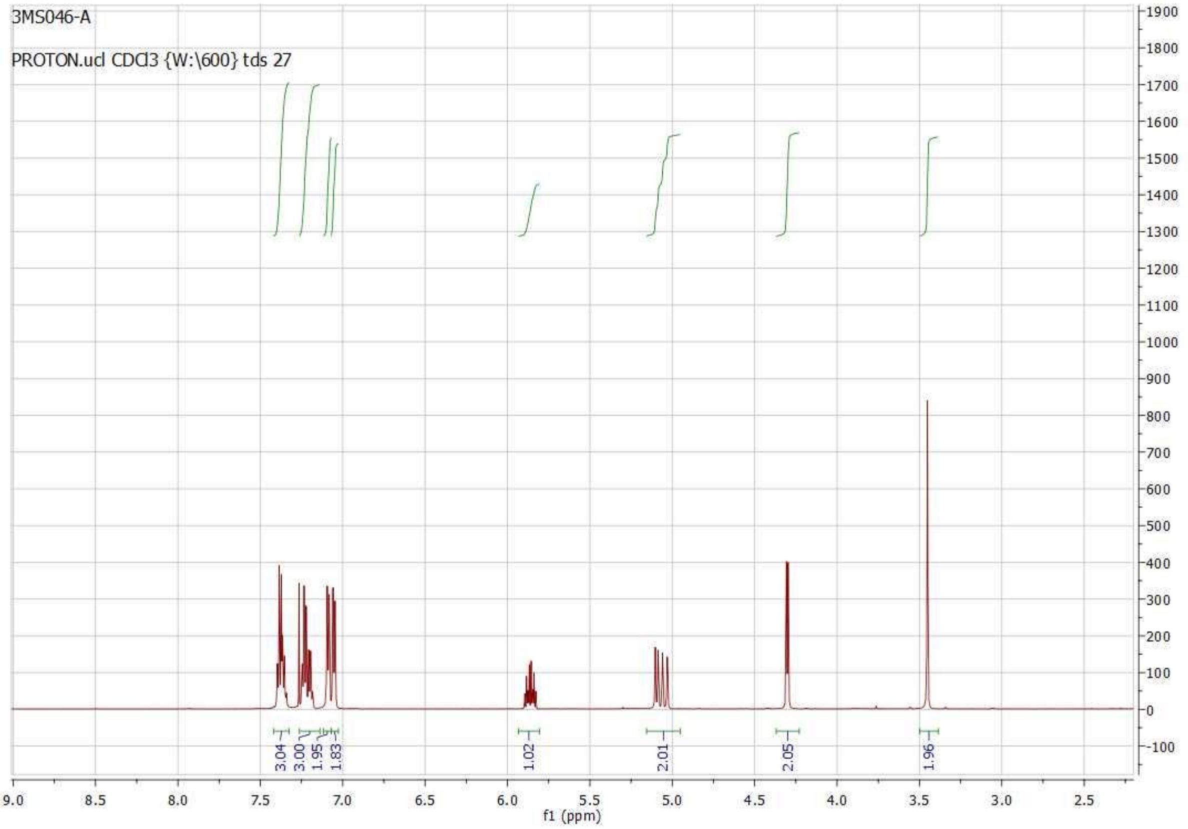

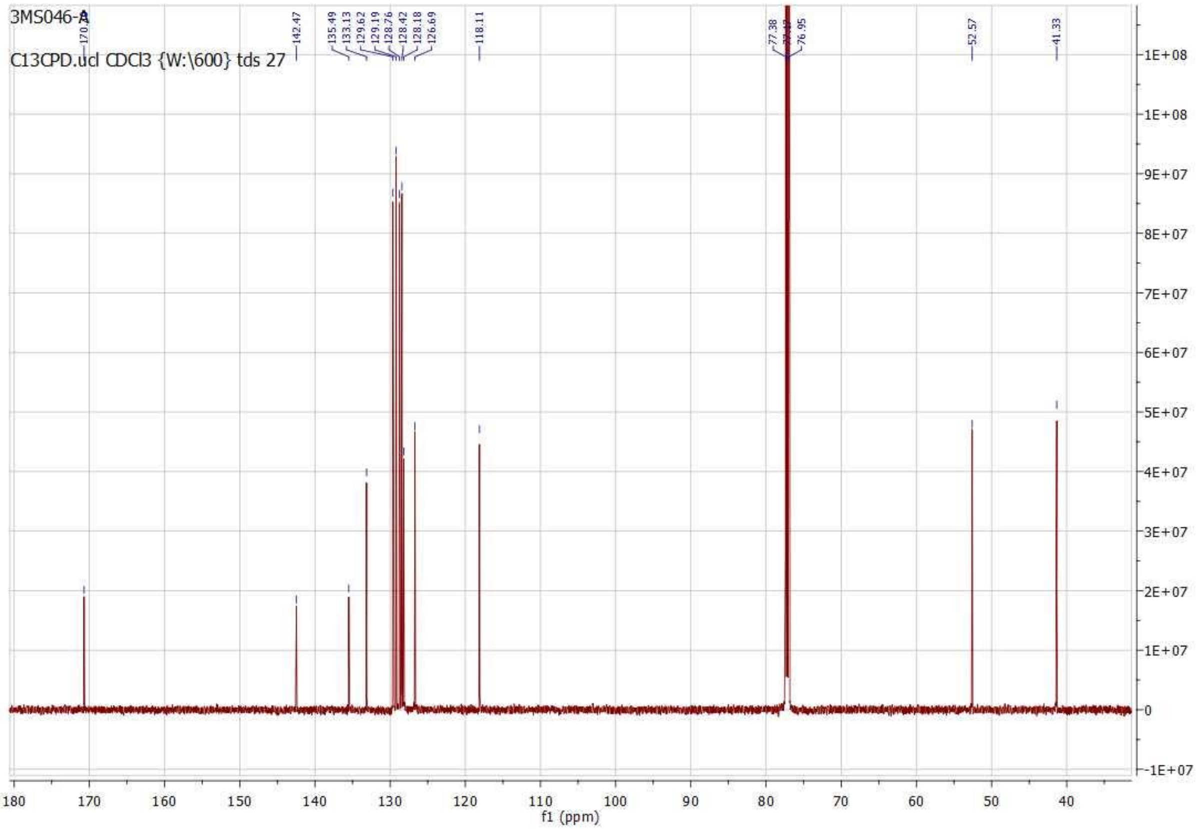

2-phenyl-1-(3-phenylpiperazin-1-yl)ethan-1-one (20)

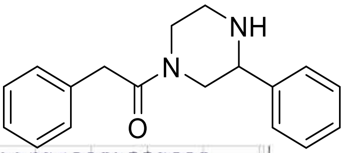

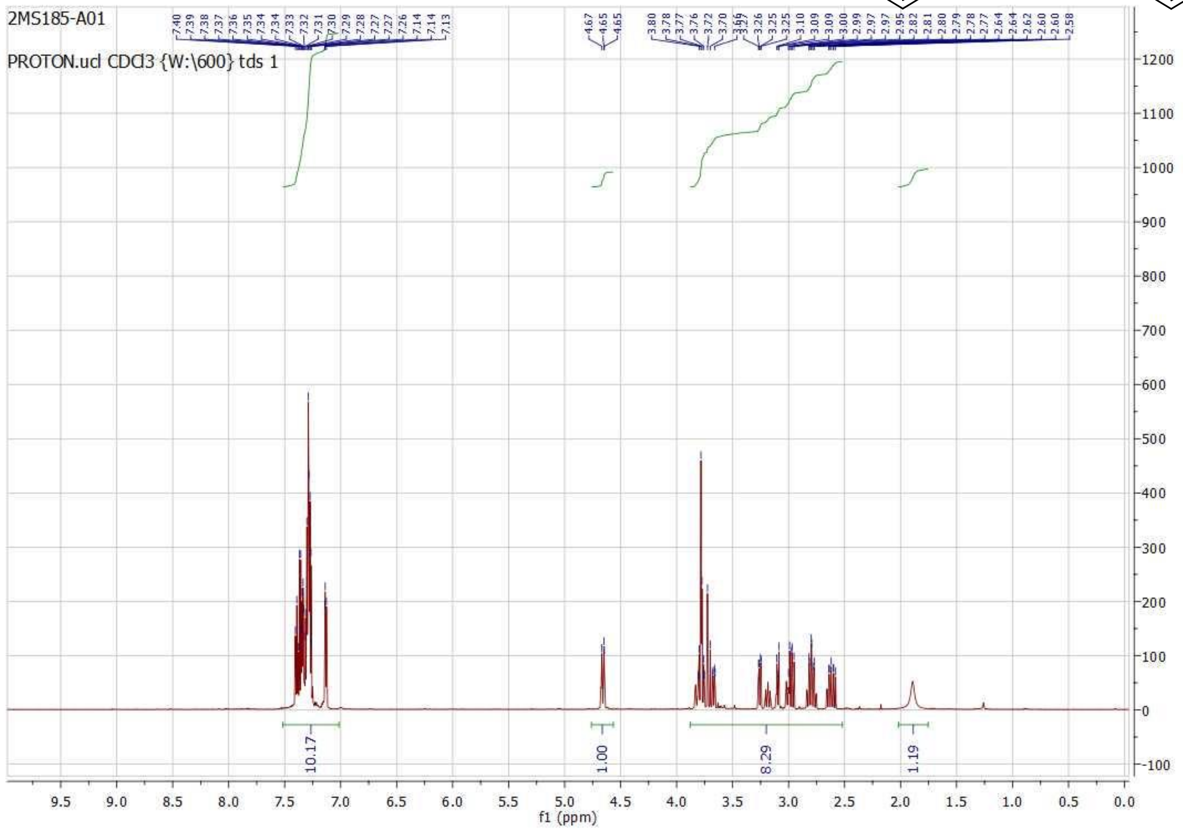

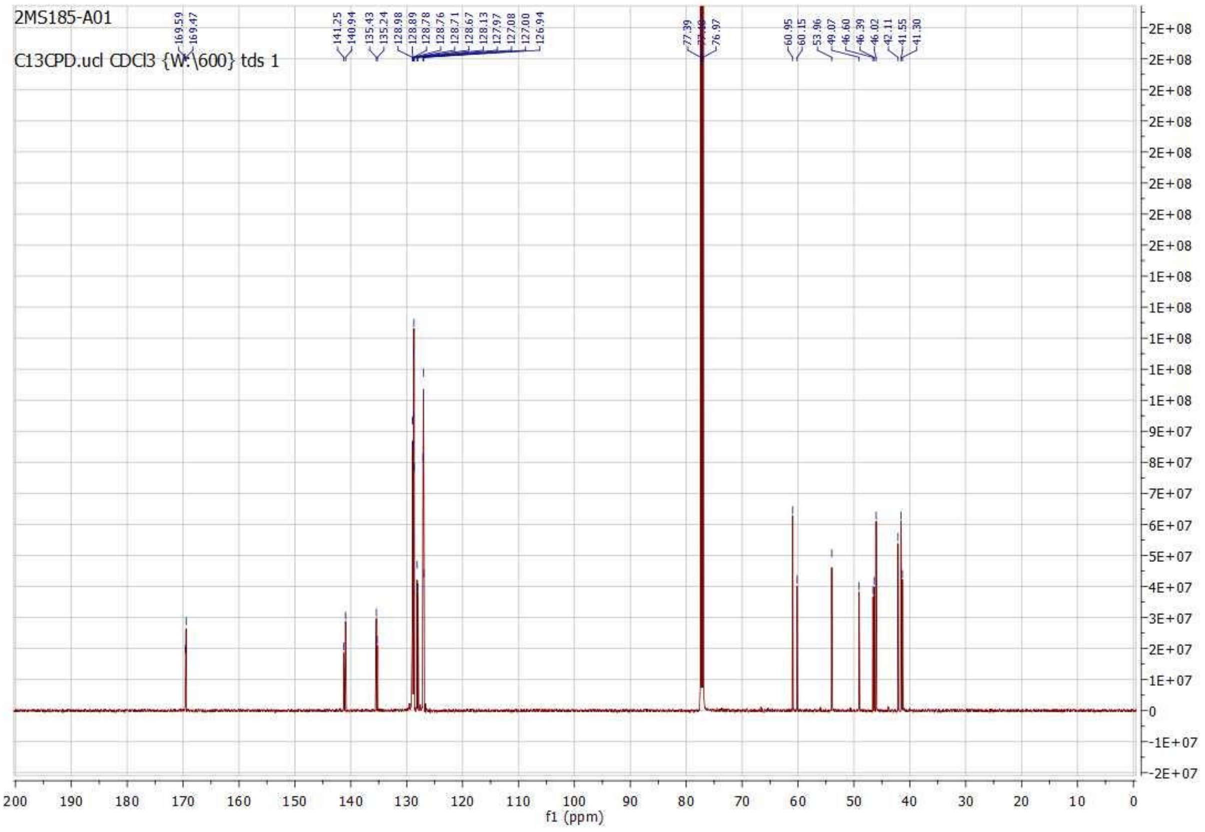

3-hydroxy-1-(3-(trifluoromethyl)-5,6-dihydro-[1,2,4]triazolo[4,3-a]pyrazin-7(8H)-yl)butan-1-one (21)

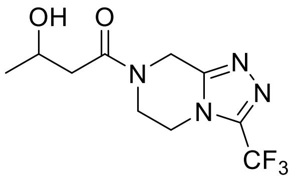

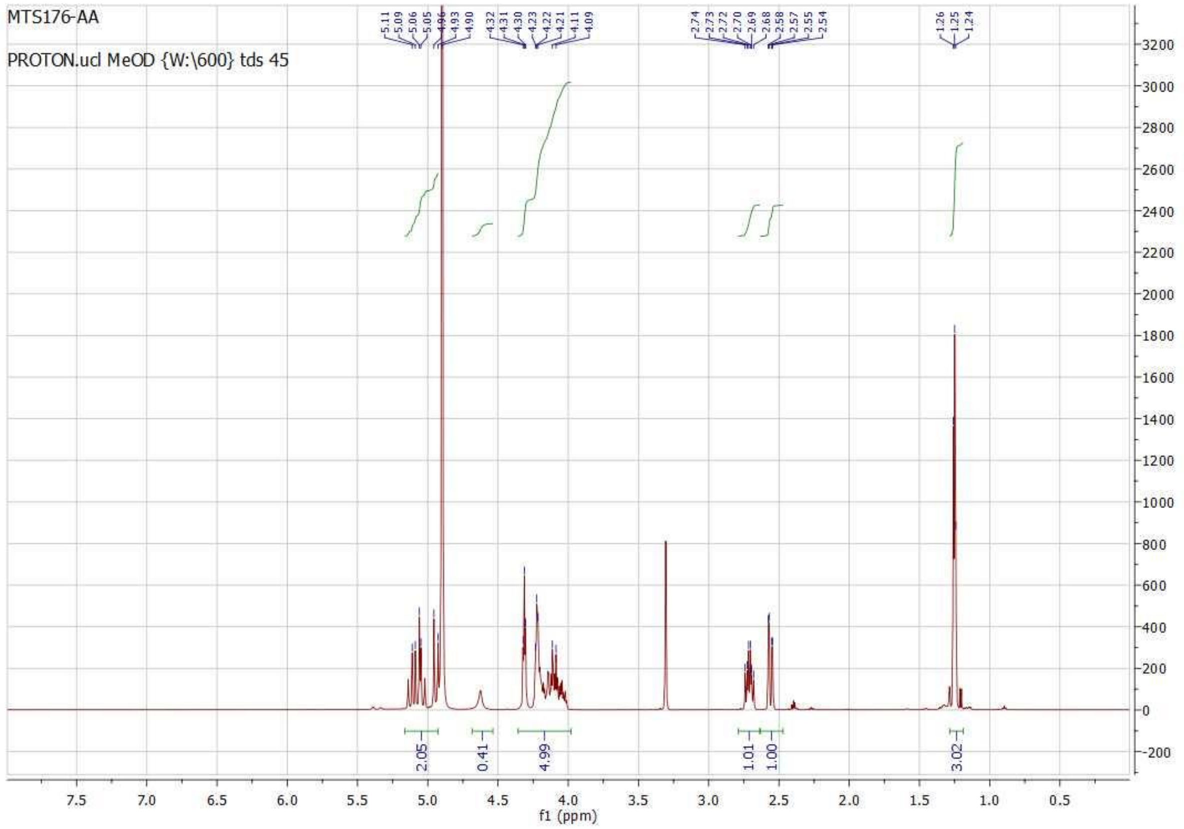

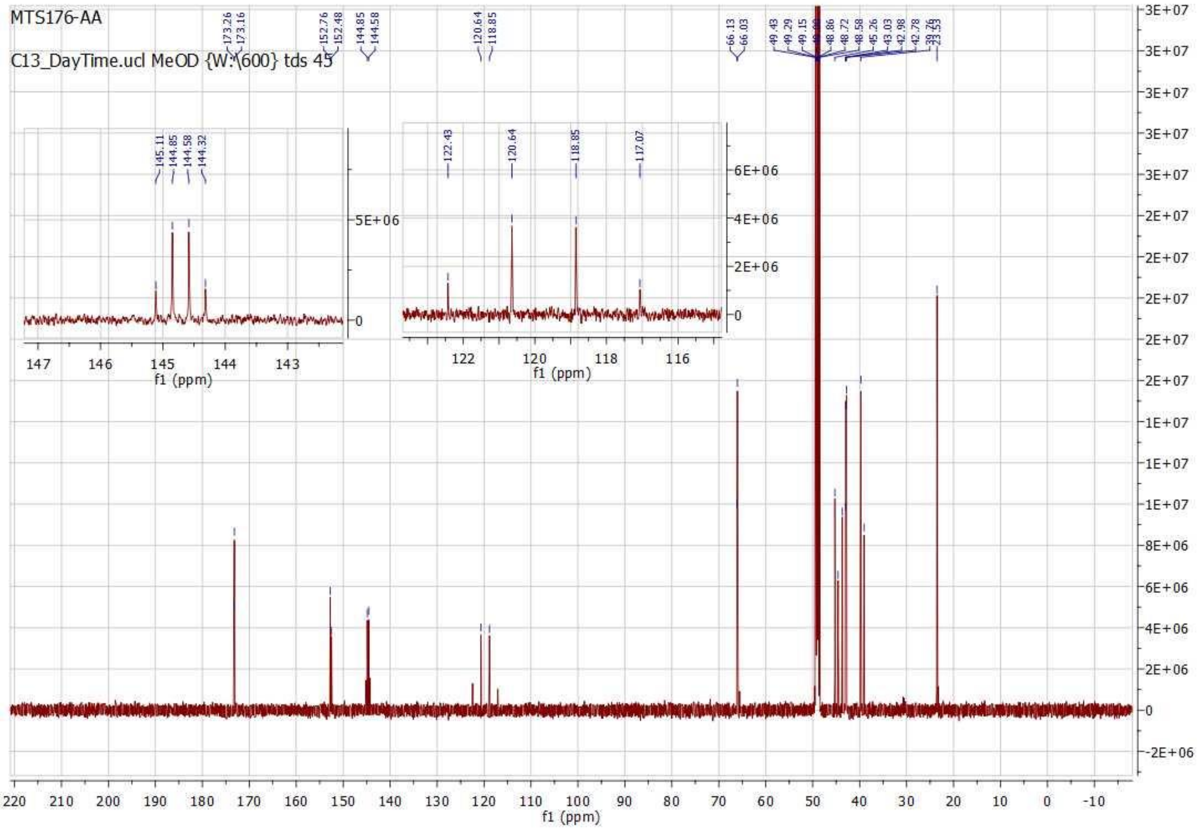

***N*-benzyl-2-(thiophen-2-yl)acetamide (22)**

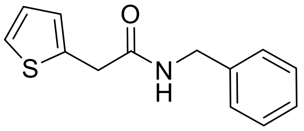

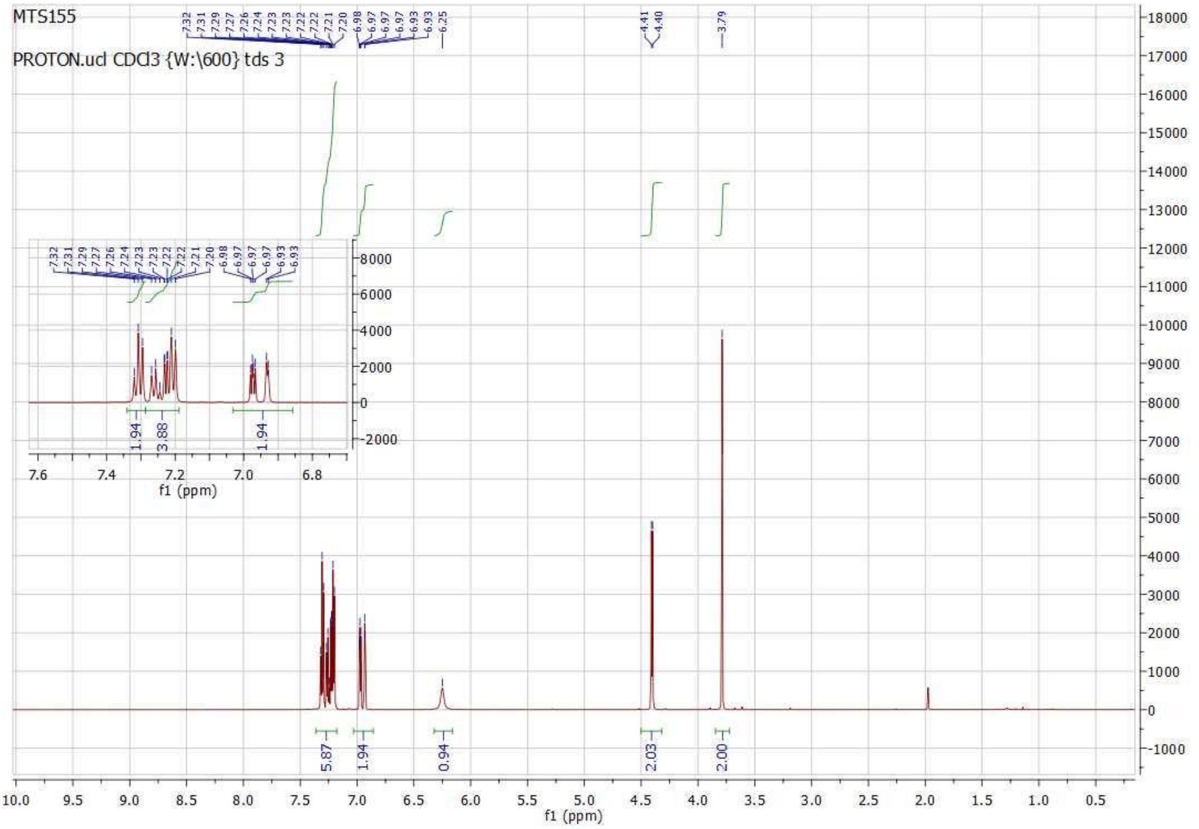

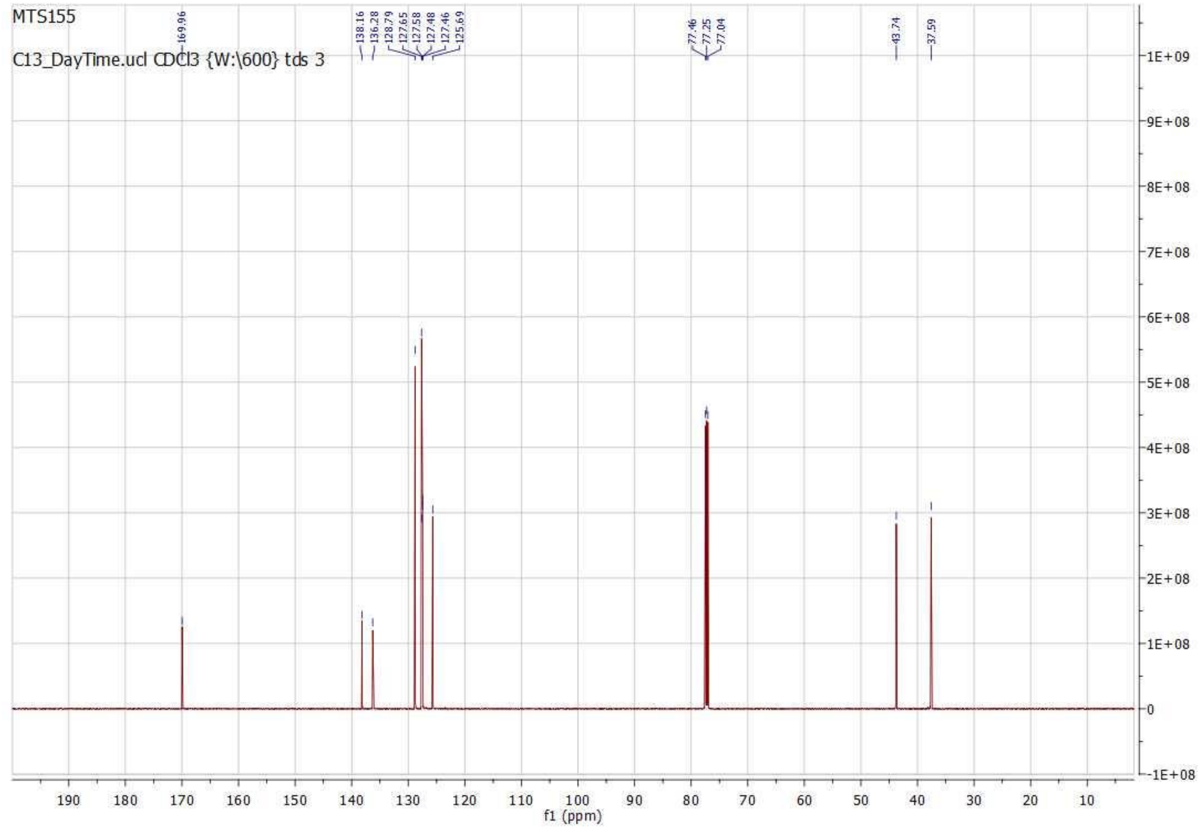

***N*-benzylpicolinamide (23)**

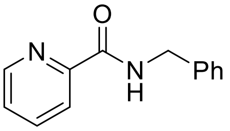

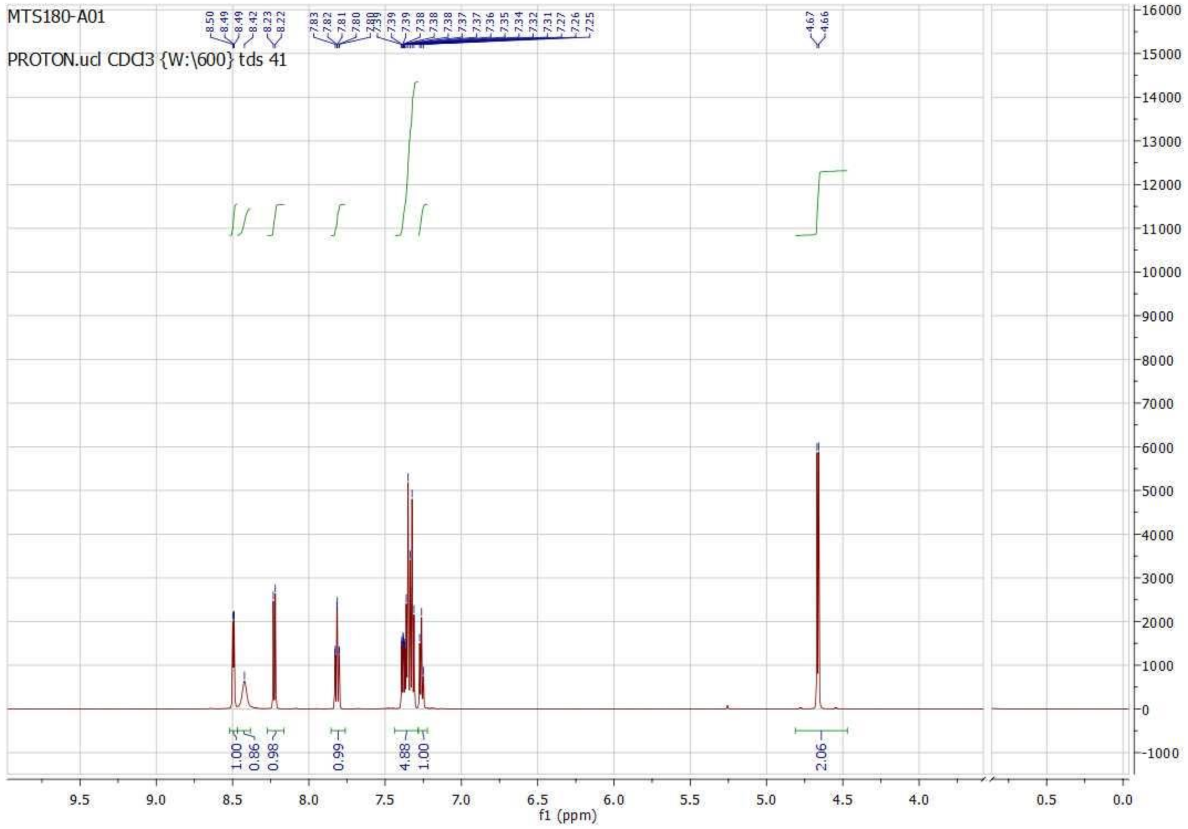

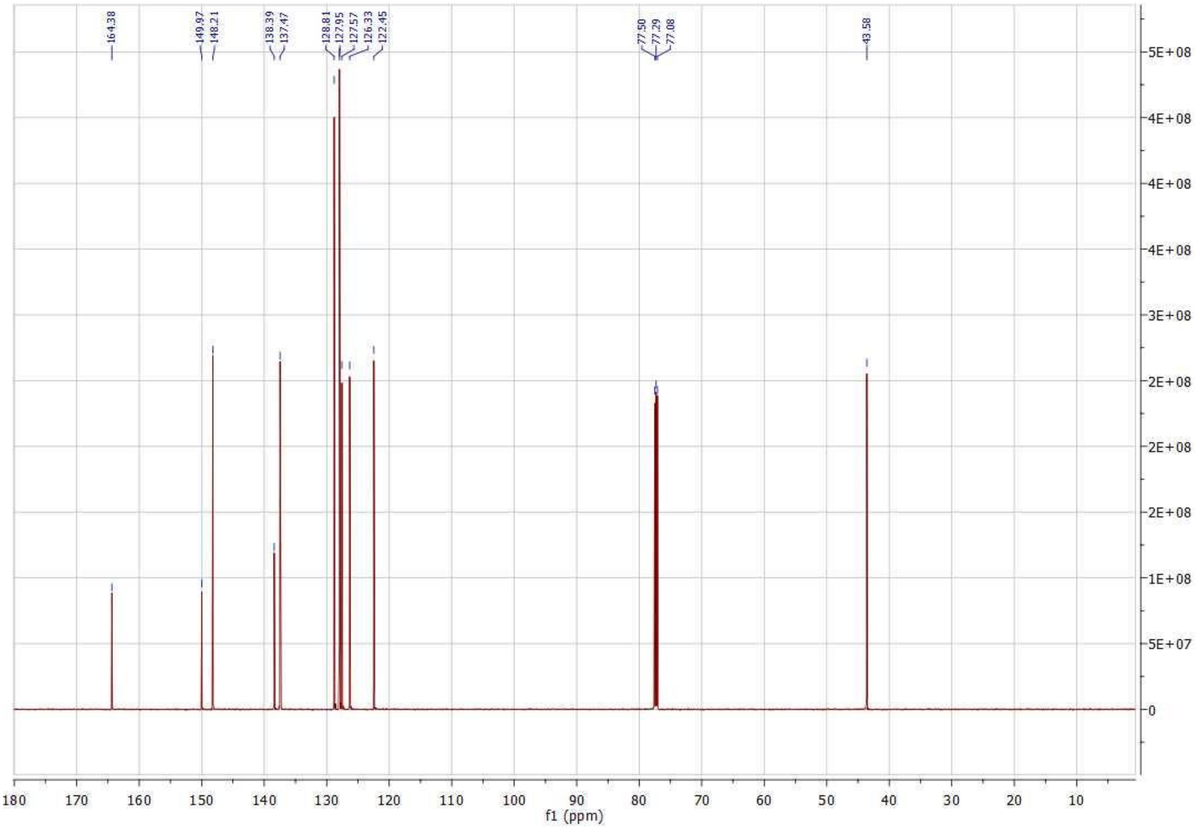

2-phenyl-N-(pyridin-2-yl)acetamide (24)

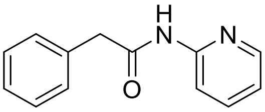

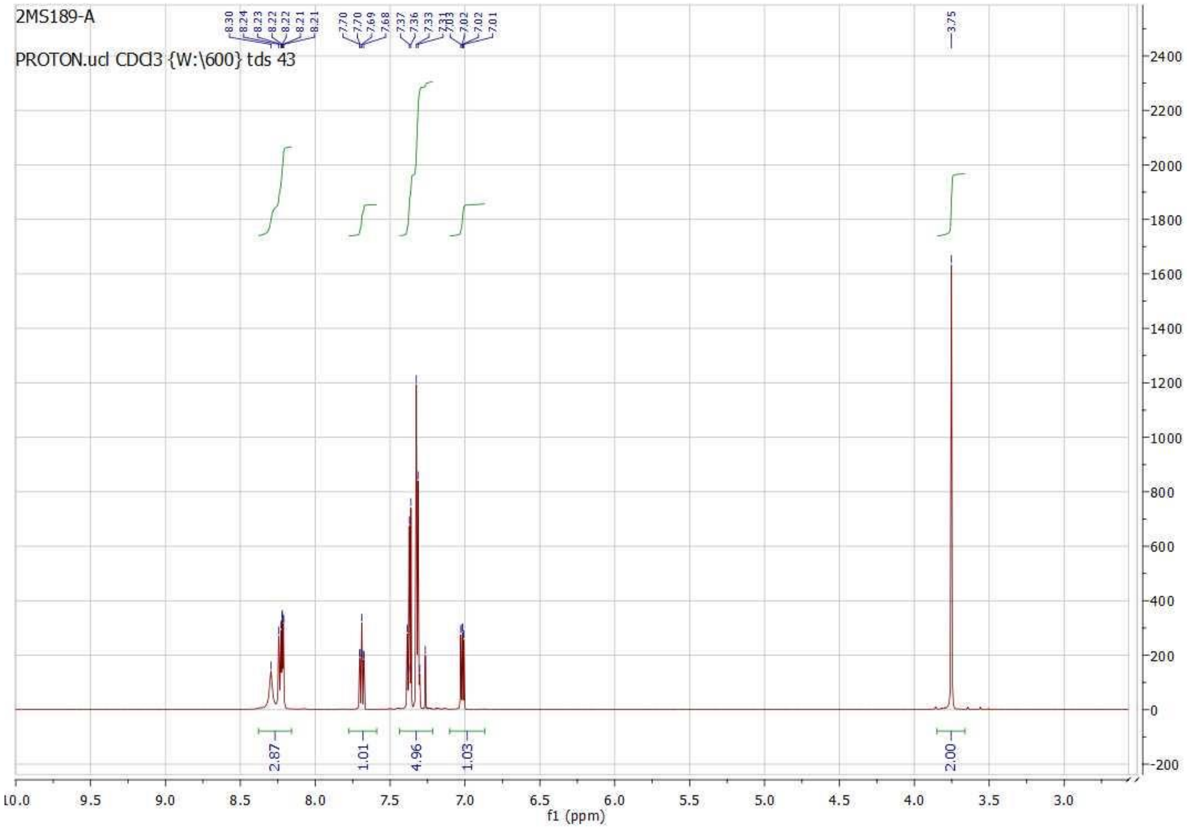

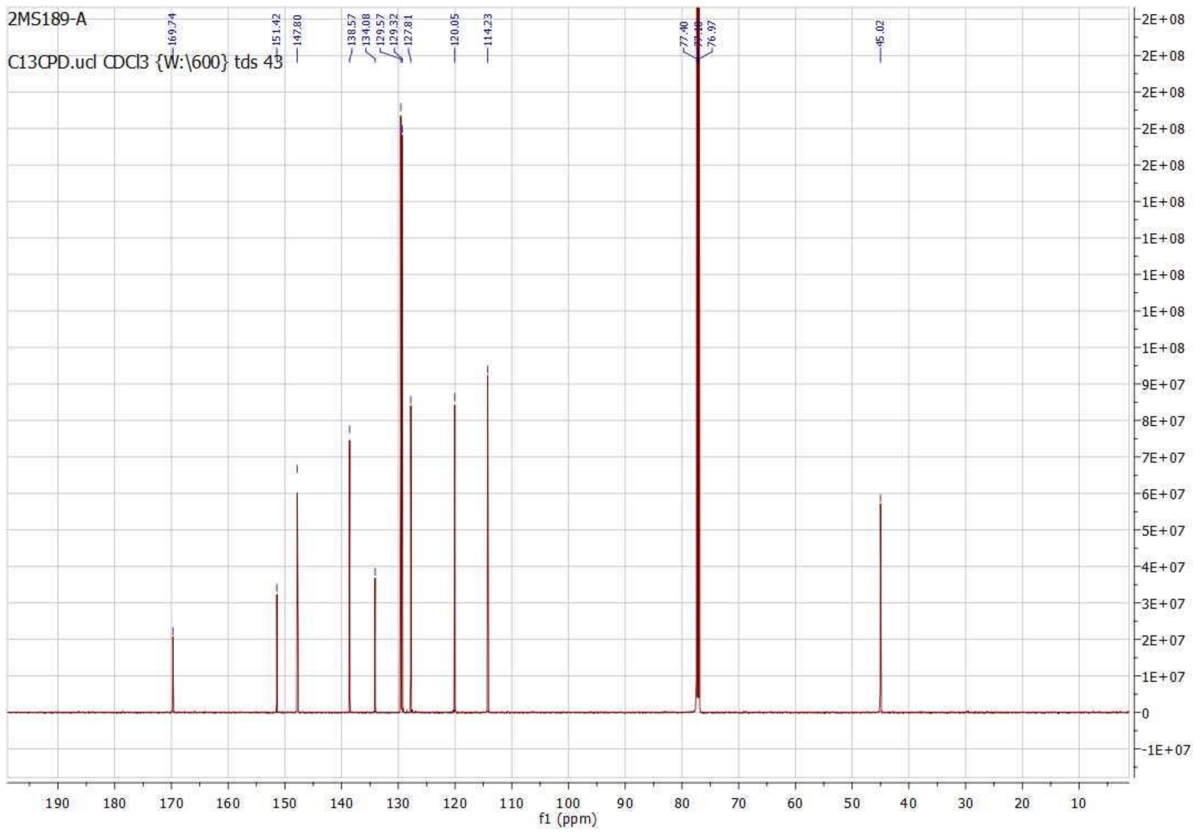

_N_-mesityl-2-phenylacetamide (25)

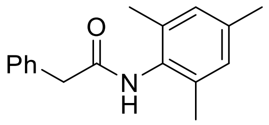

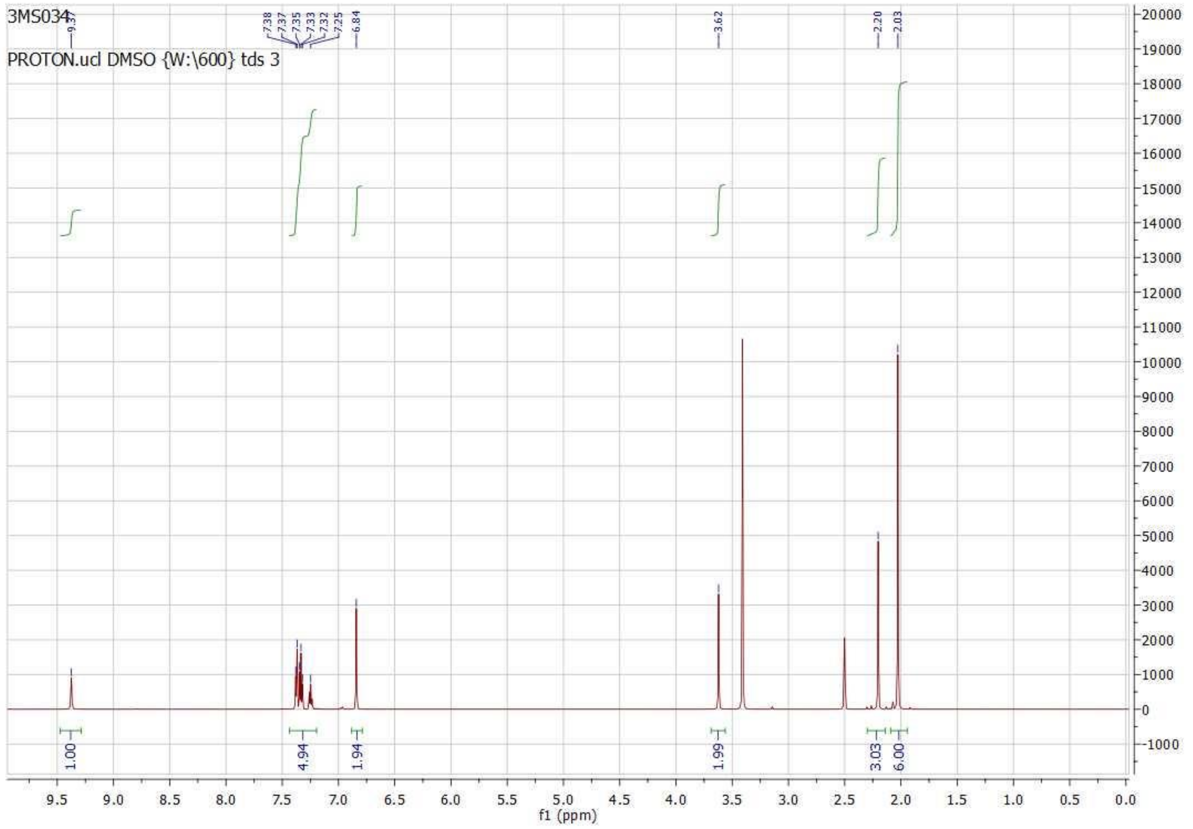

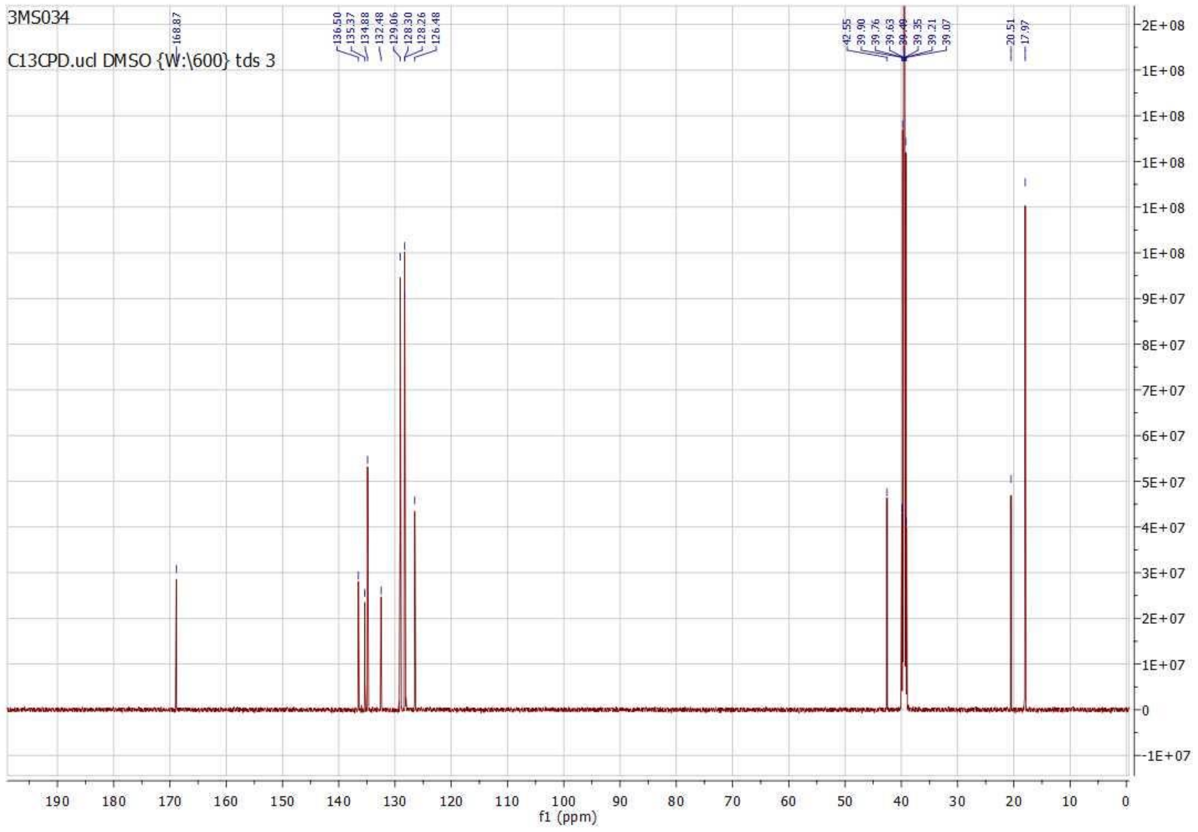

N-benzylpivalamide (26)

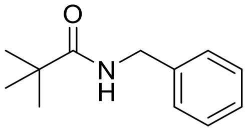

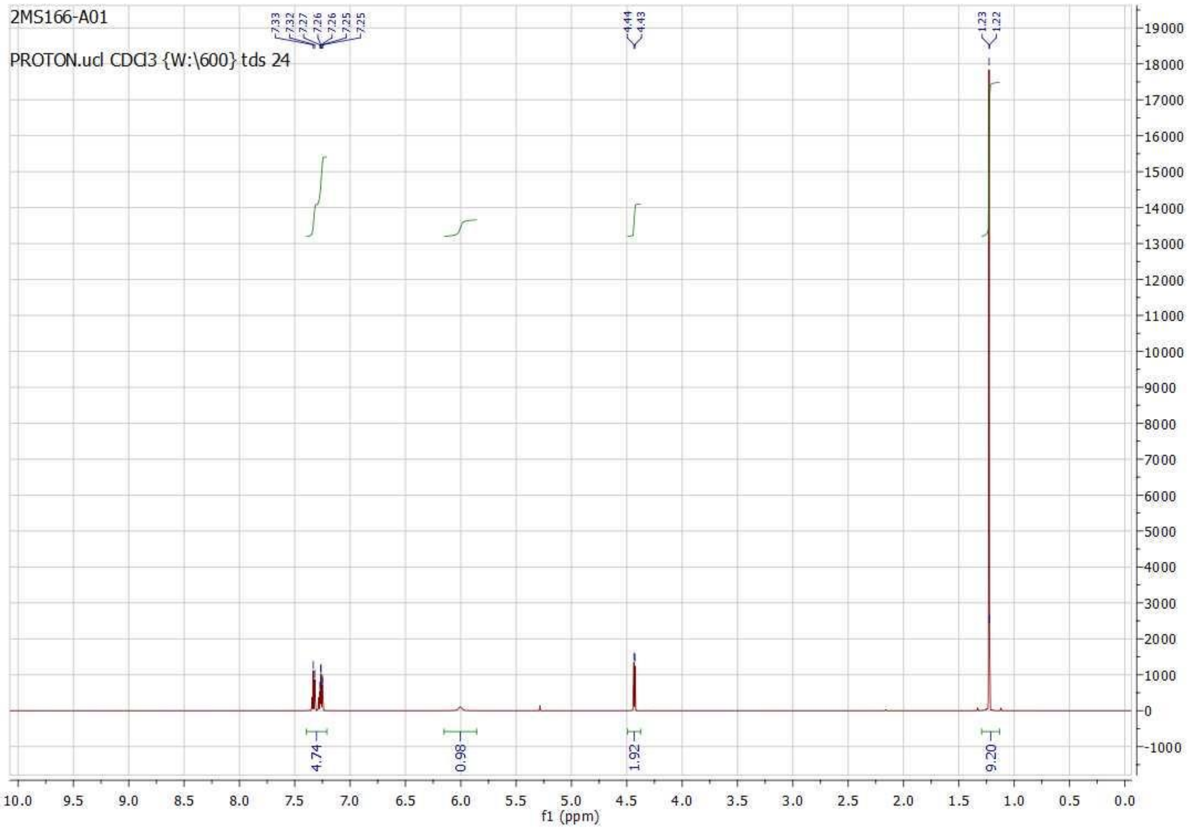

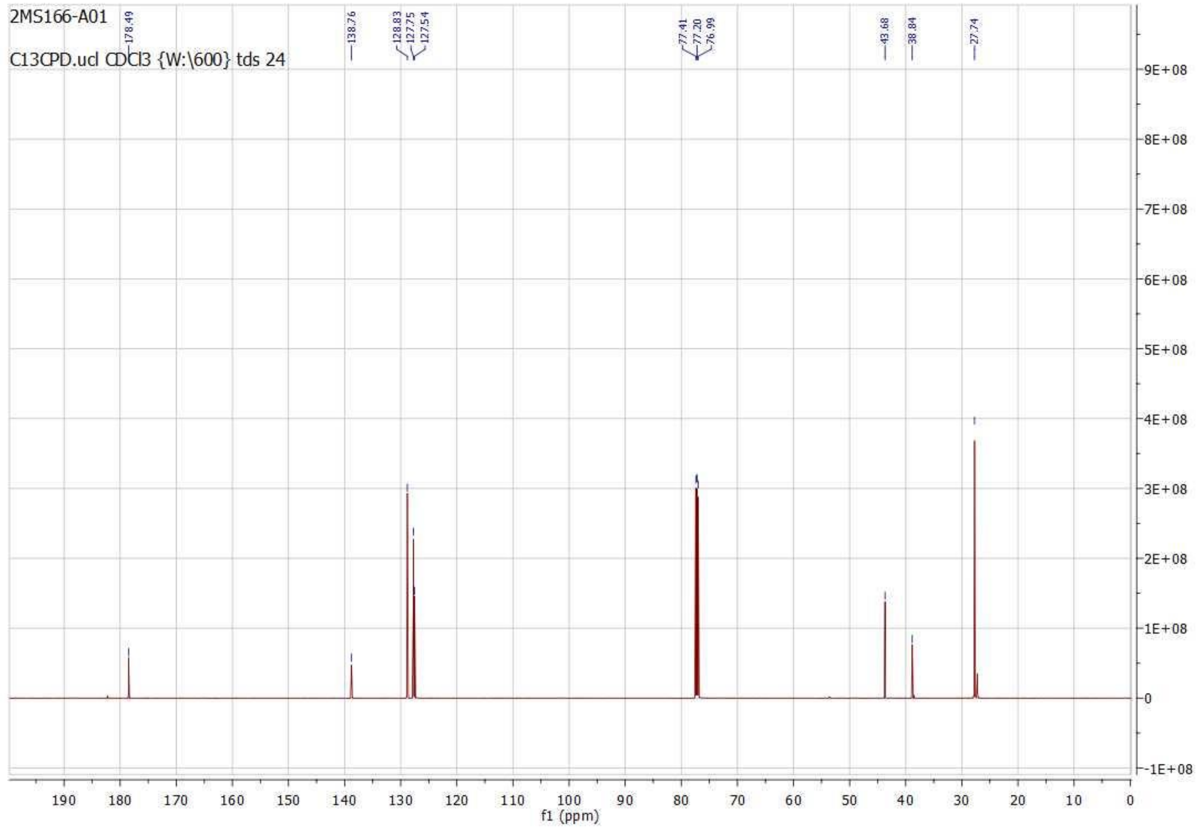

***N*-benzylbut-2-ynamide (27)**

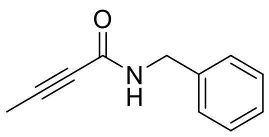

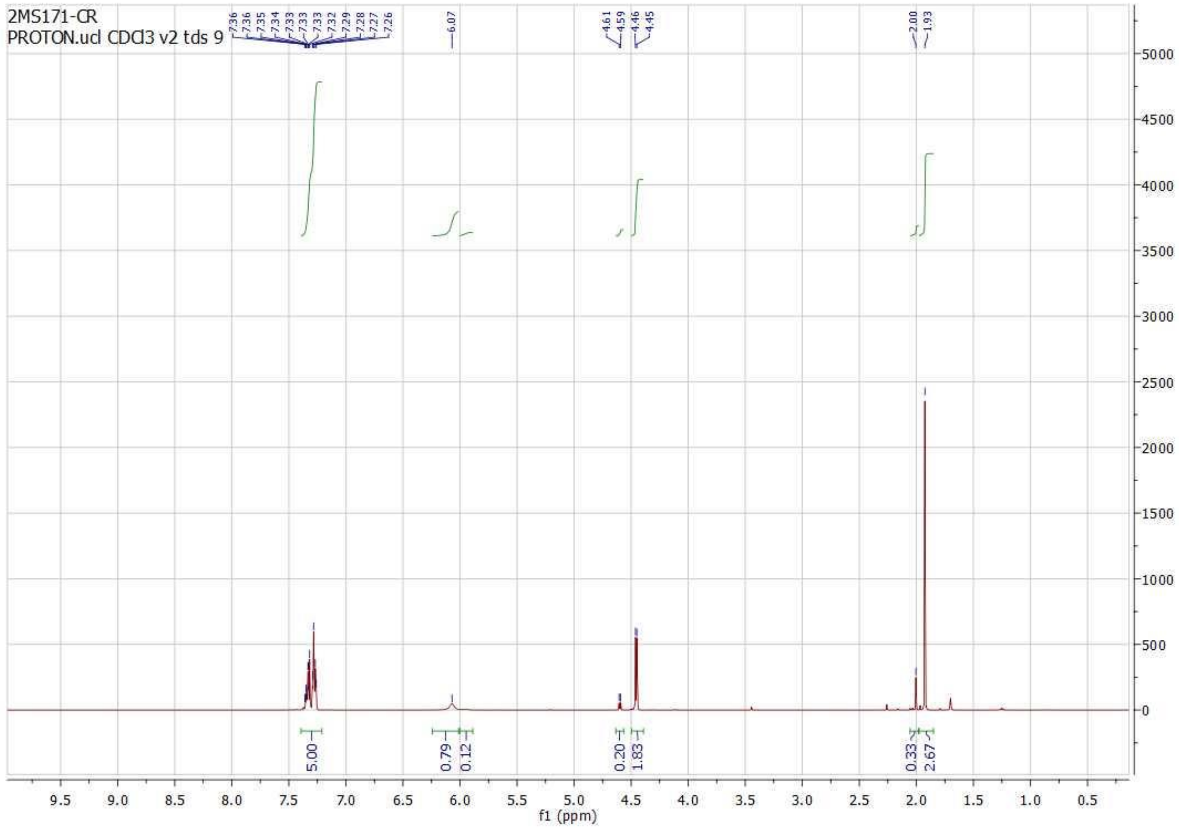

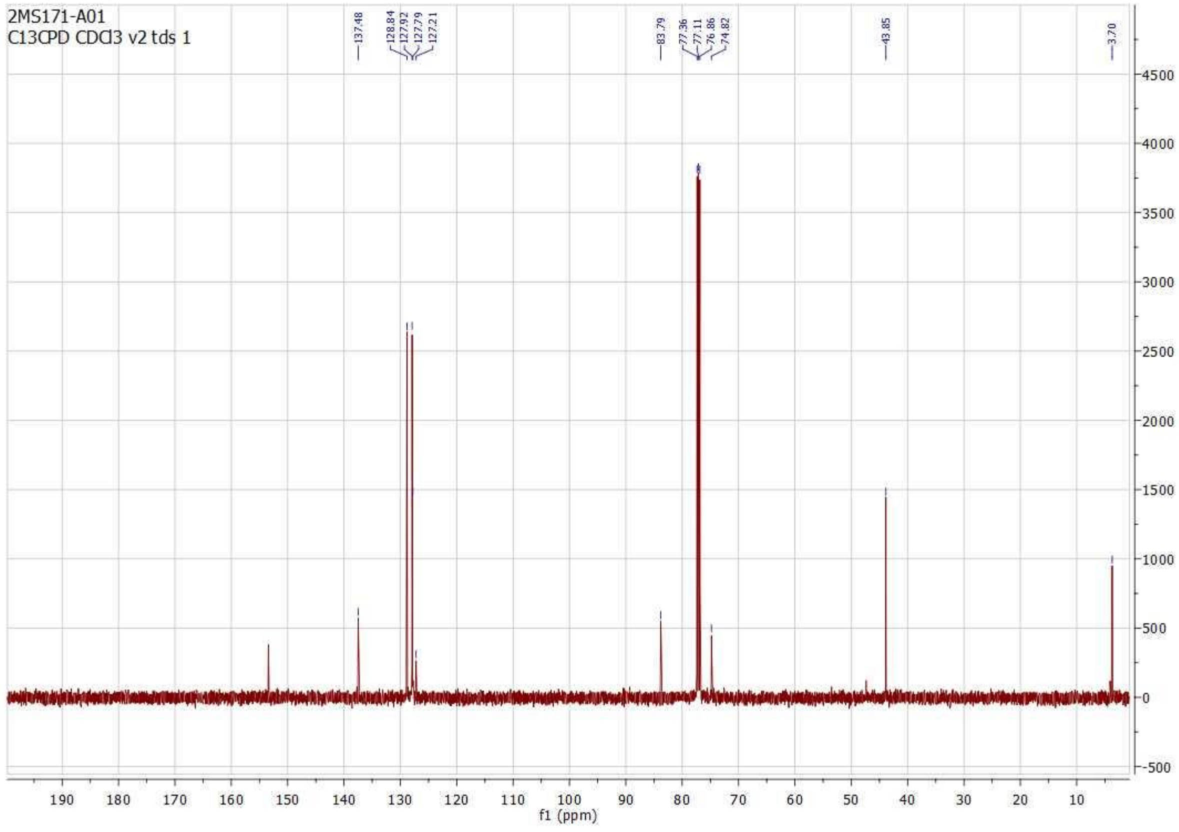

***N*-(2-(1H-indol-3-yl)ethyl)-2-phenylacetamide (28)**

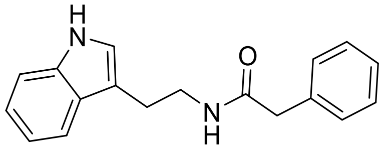

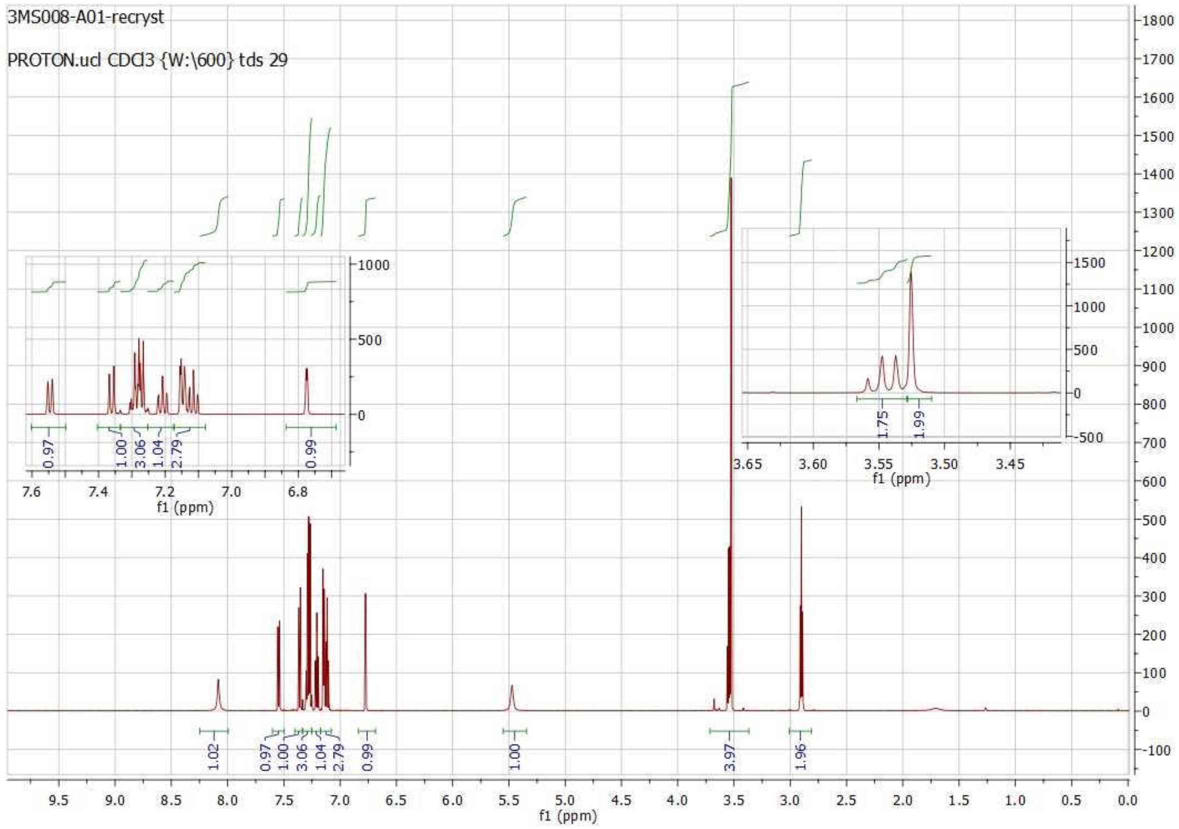

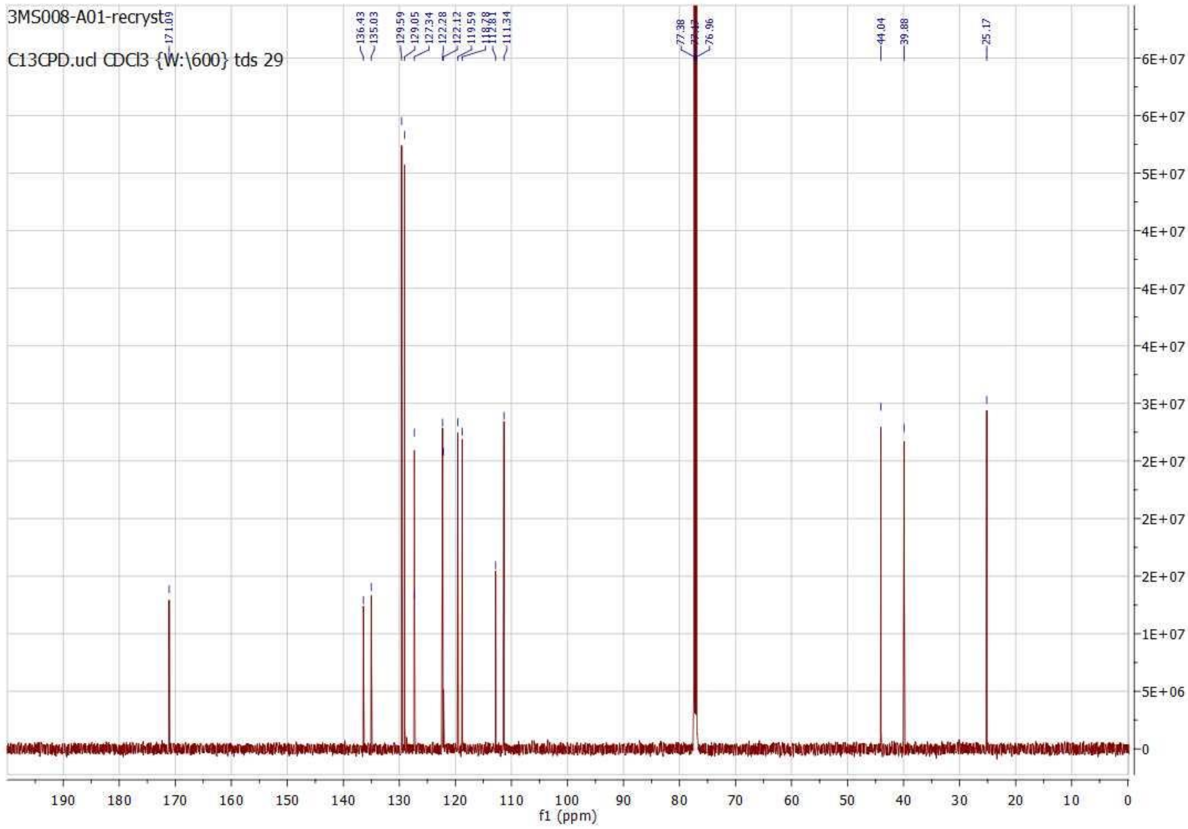

***N*-benzyl-2-(1H-indol-3-yl)acetamide (29)**

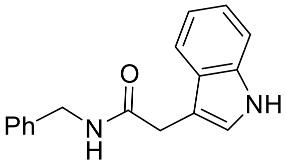

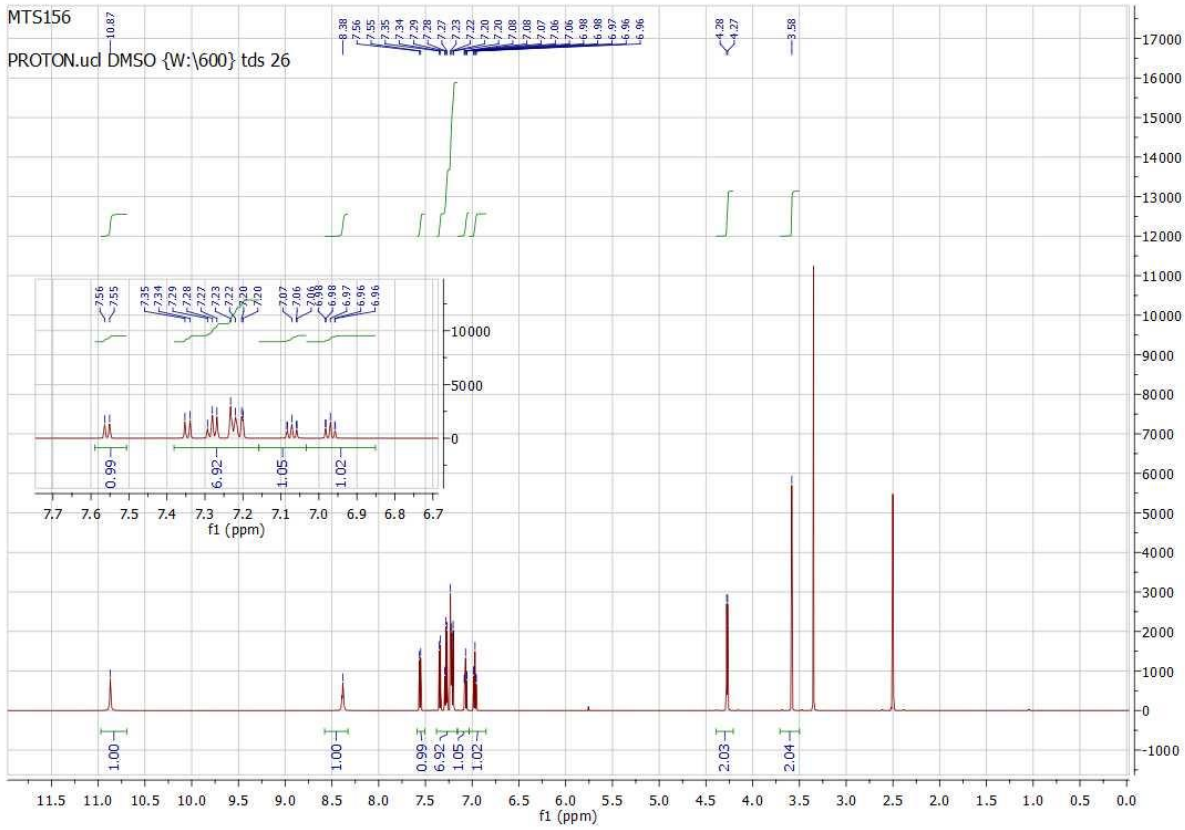

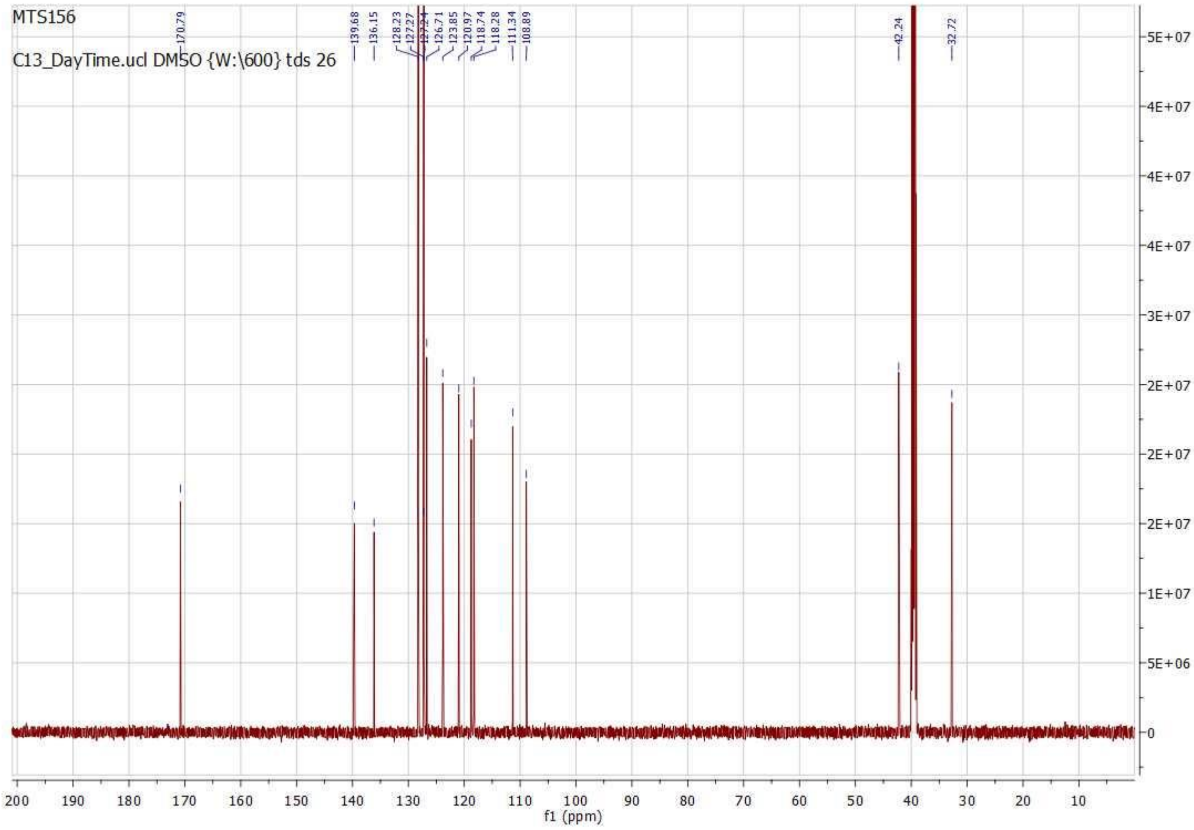

2-phenyl-N-tosylacetamide (30)

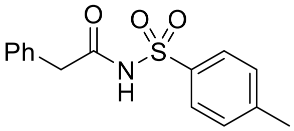

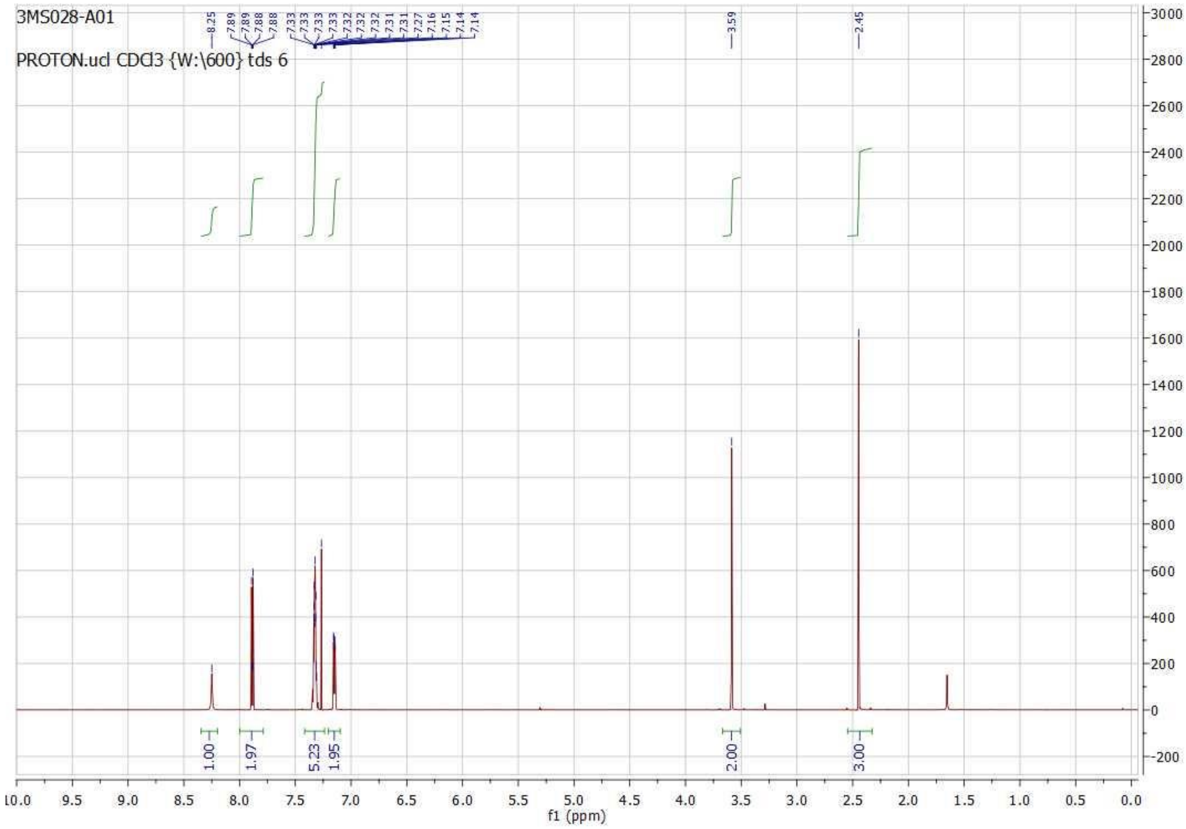

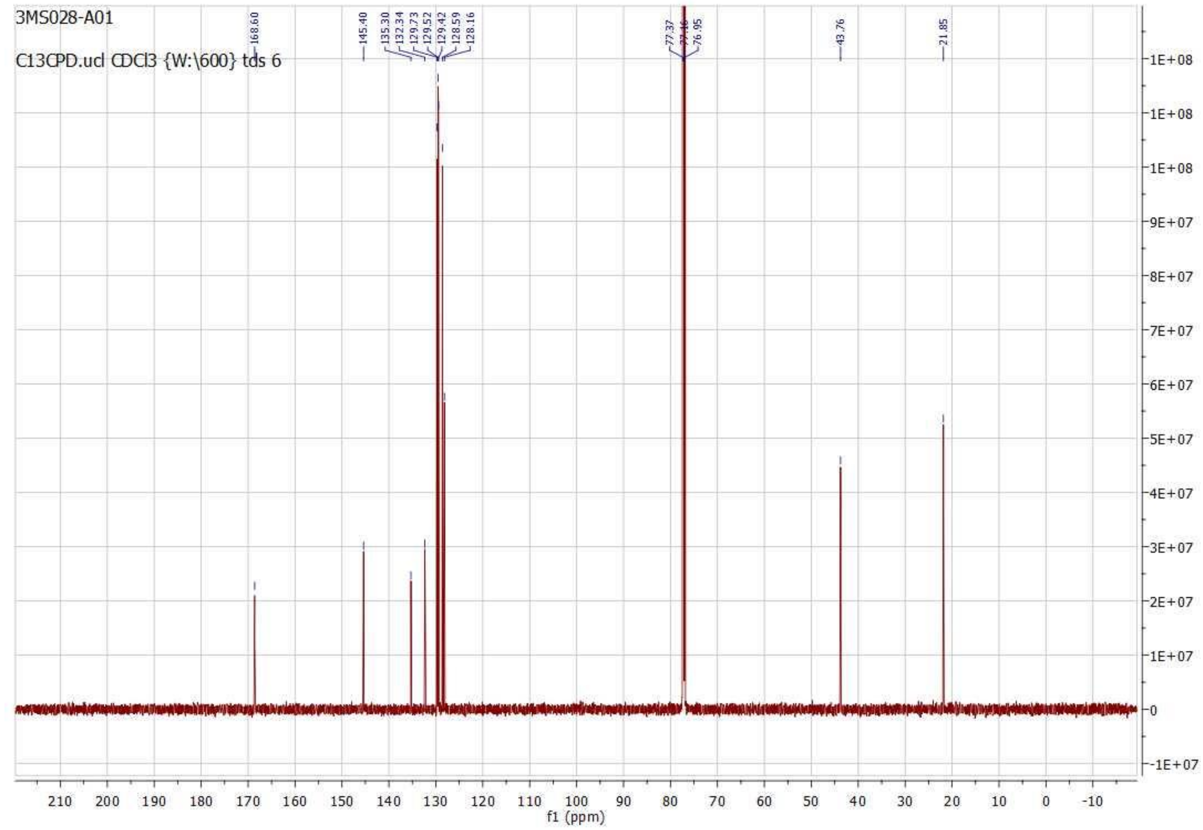

(*S*)-N-Benzyl-2-(4-isobutylphenyl)propanamide (31)

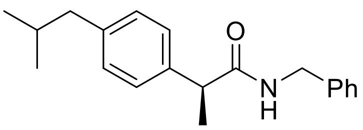

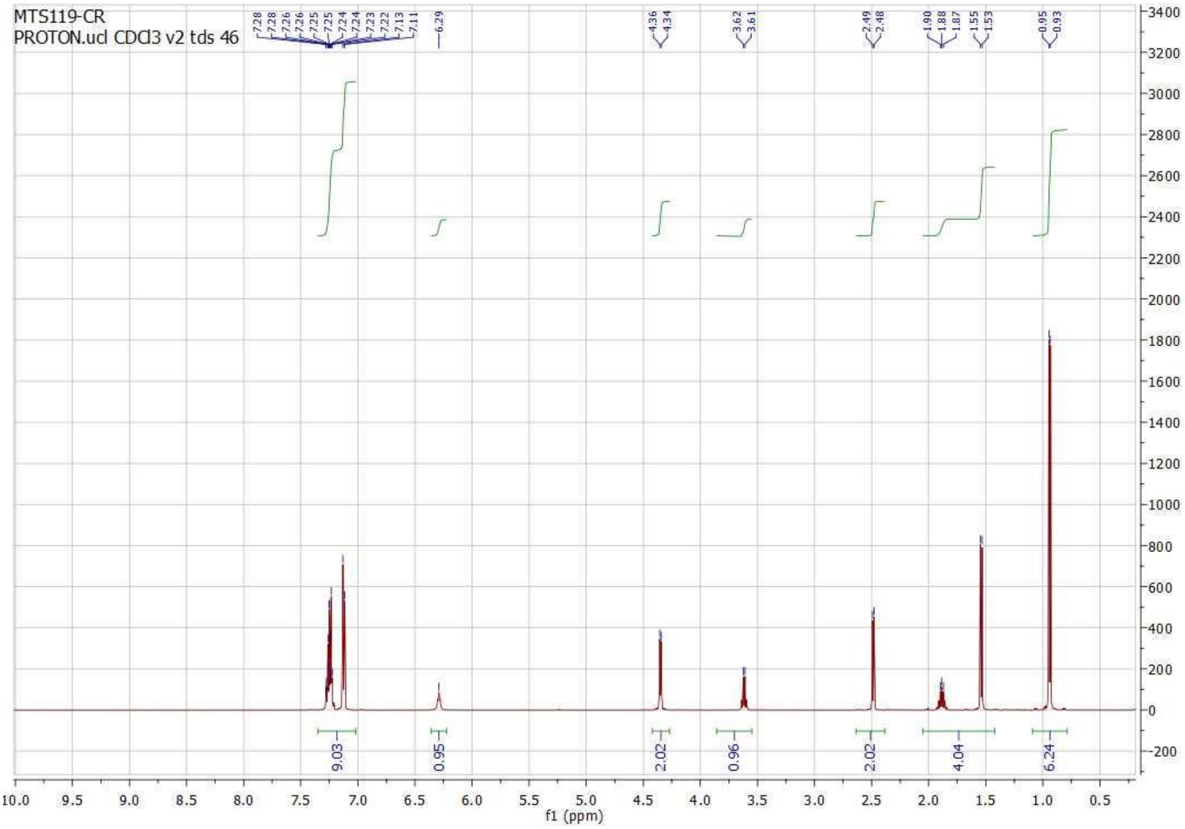

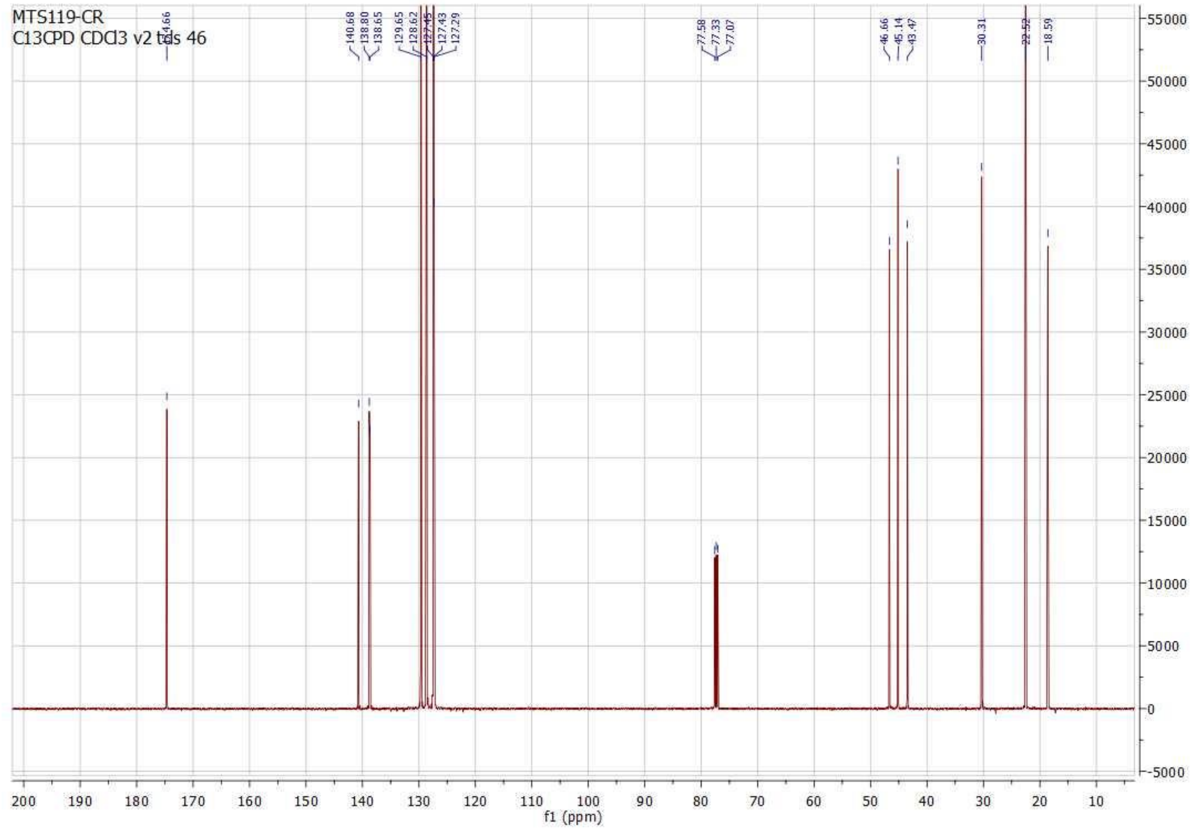

N-benzyl-5-((3aS,4S,6aR)-2-oxohexahydro-1H-thieno[3,4-d]imidazol-4-yl)pentanamide (32)

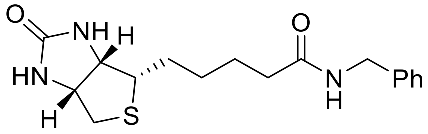

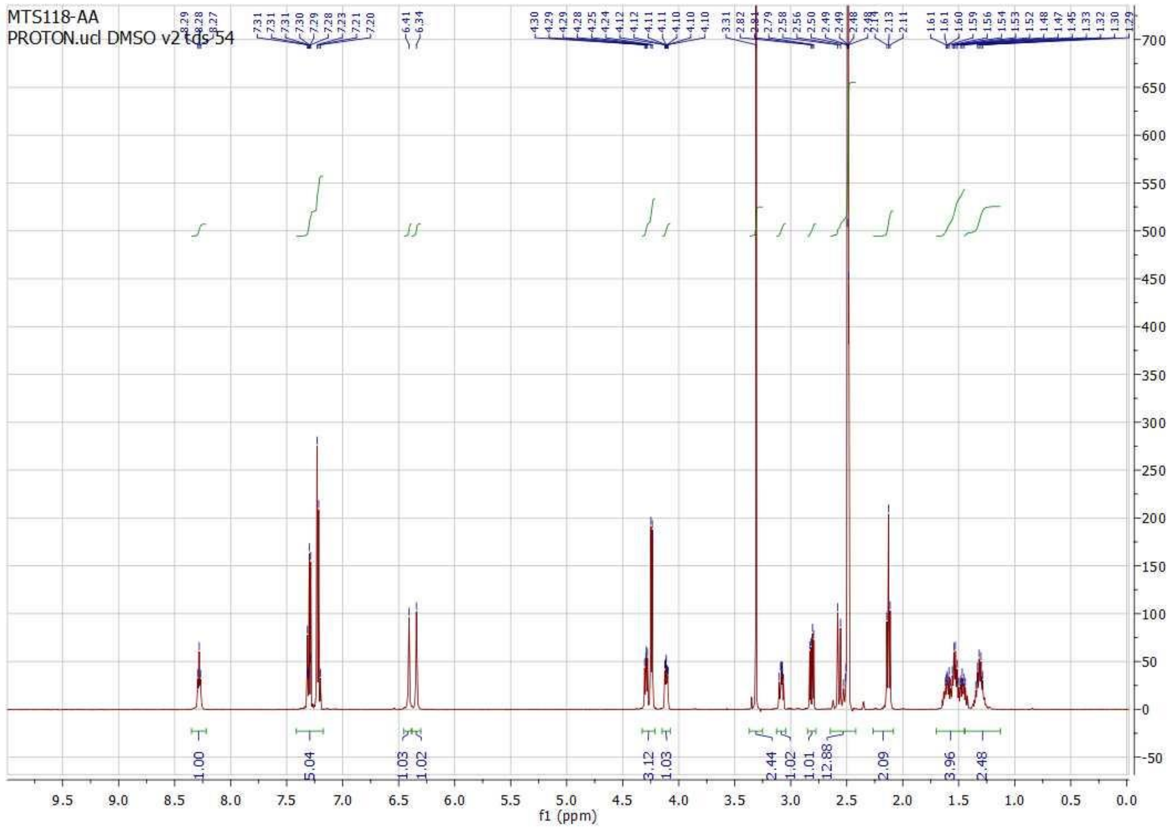

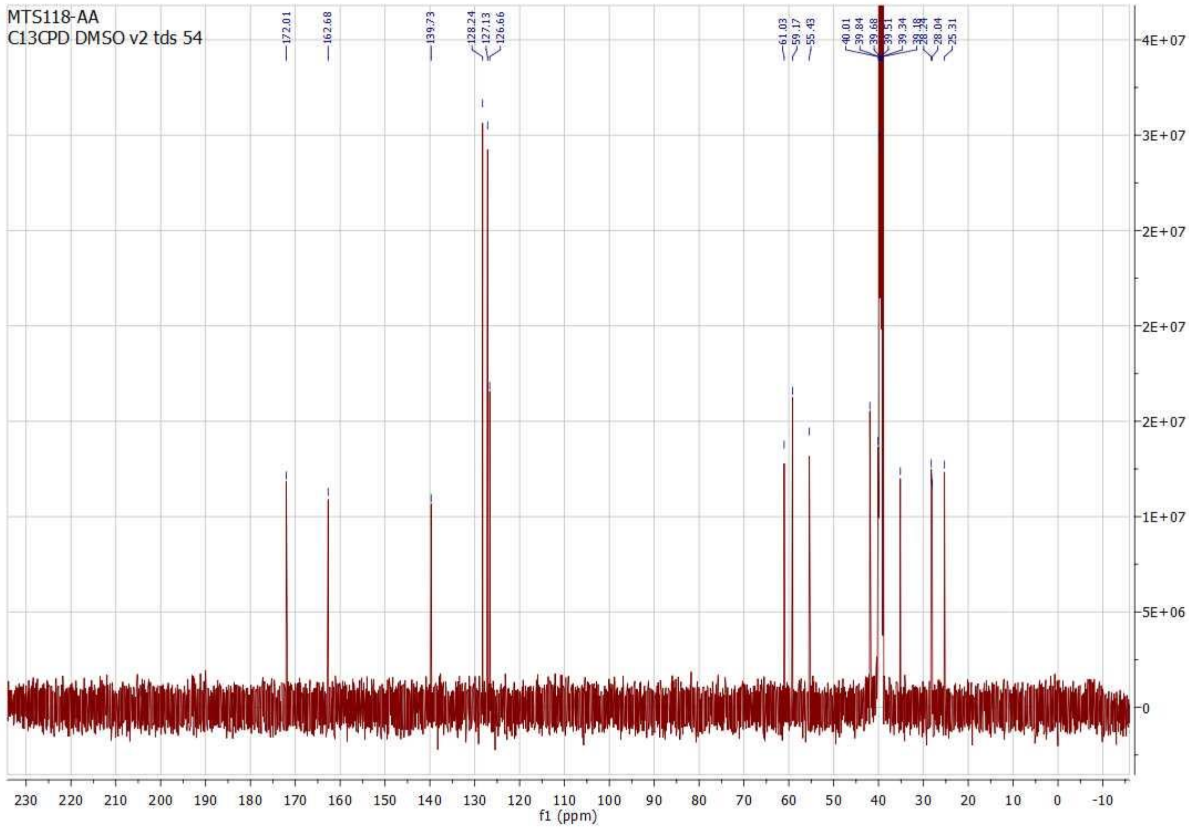

_(4R)-N_-benzyl-4-_((3R,8R,9S,10S,13R,14S,17R)_-3-hydroxy-10,13-dimethylhexadecahydro-1H-cyclopenta[a]phenanthren-17-yl)pentanamide (33)

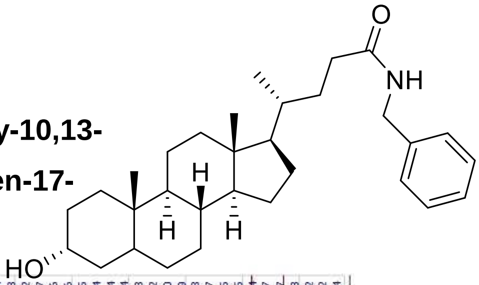

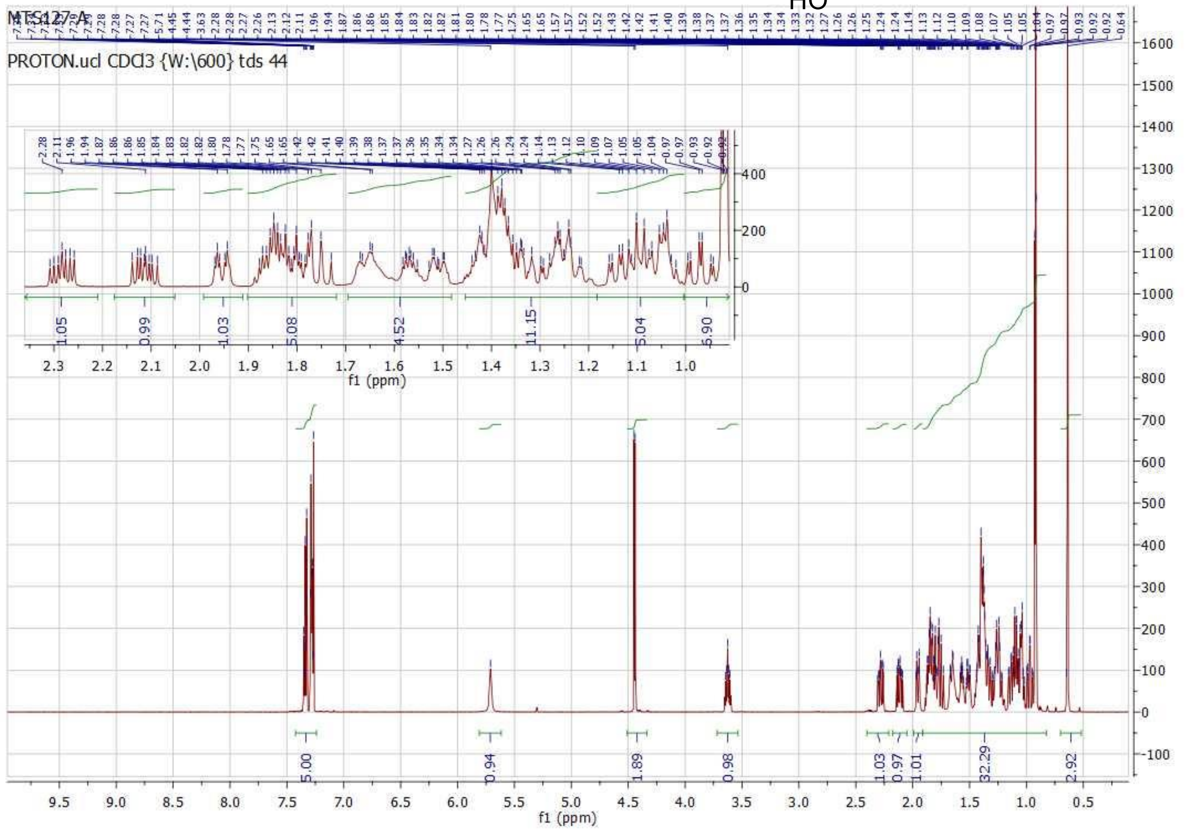

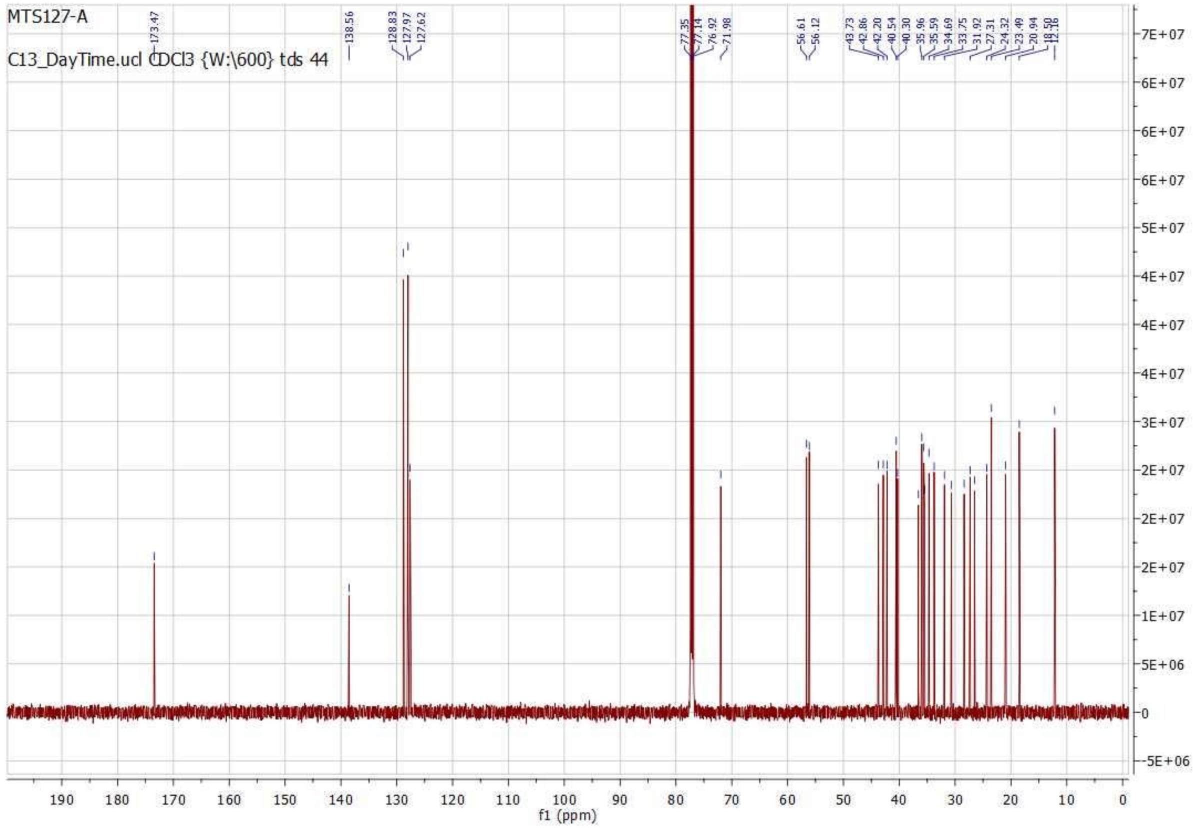

***tert*-butyl (2-oxo-2-((2-oxoazepan-3-yl)amino)ethyl)carbamate (34)**

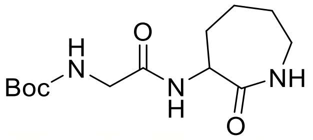

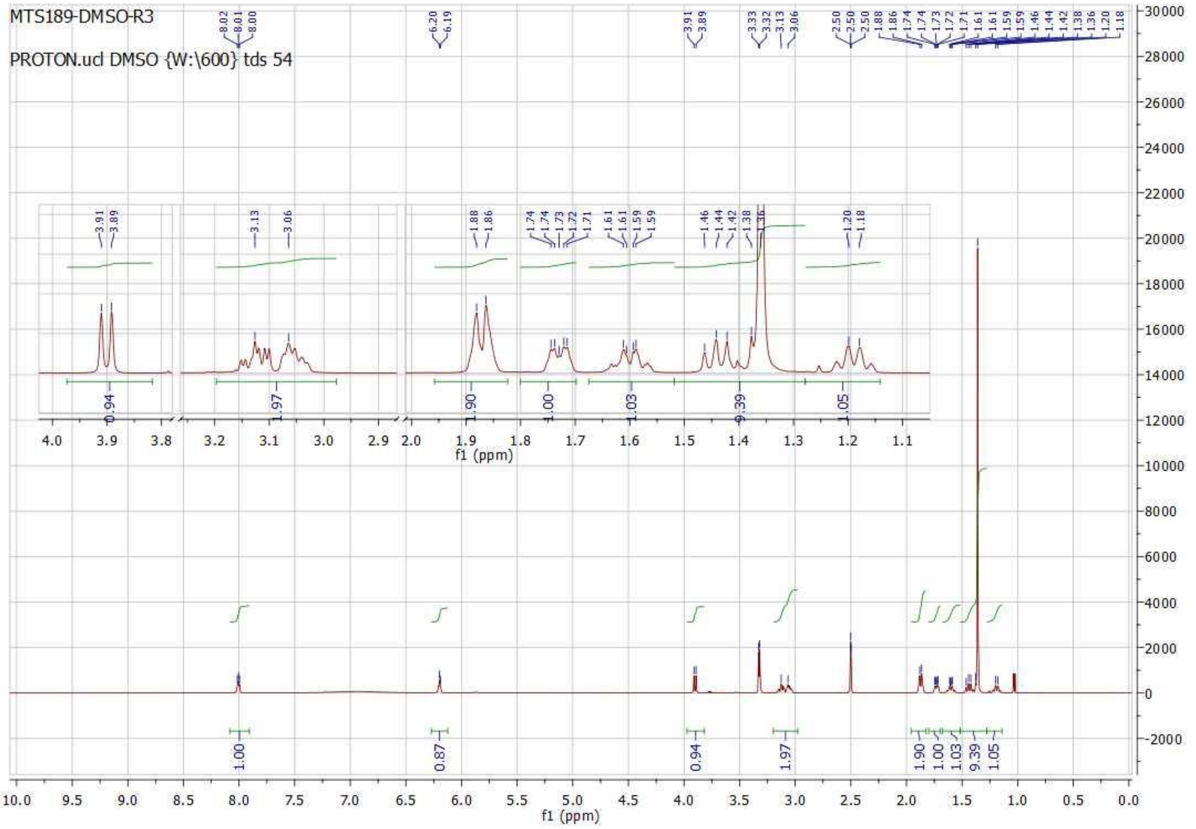

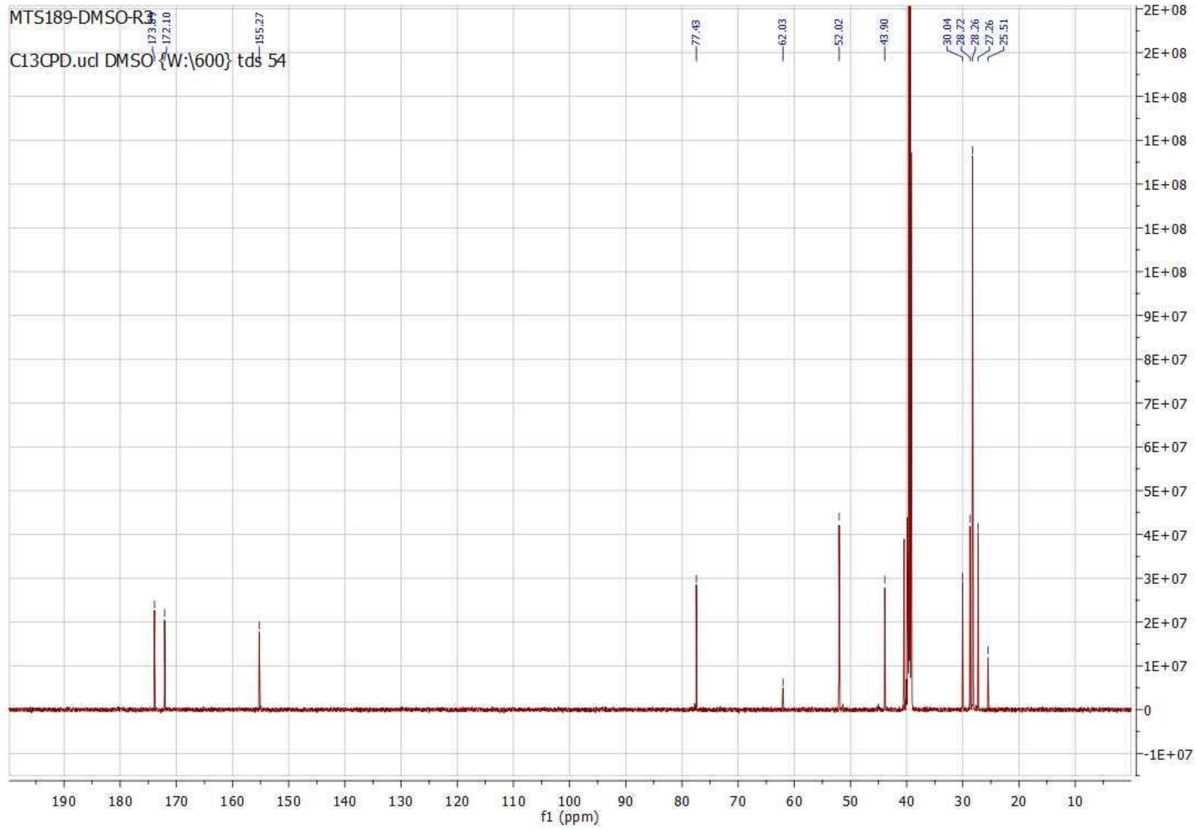

***tert*-butyl ((*2S,3R*)-1-((1H-pyrazol-3-yl)amino)-3-hydroxy-1-oxobutan-2-yl)carbamate (35)**

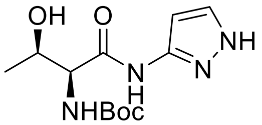

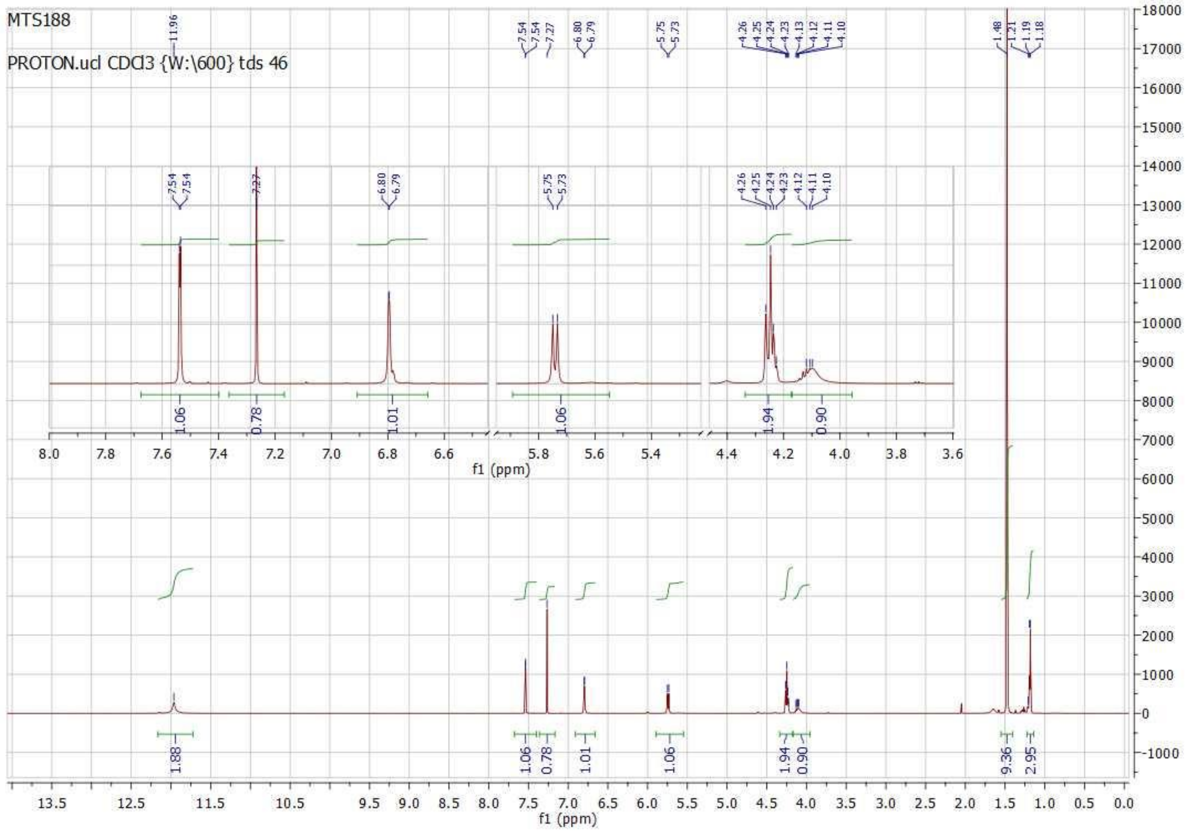

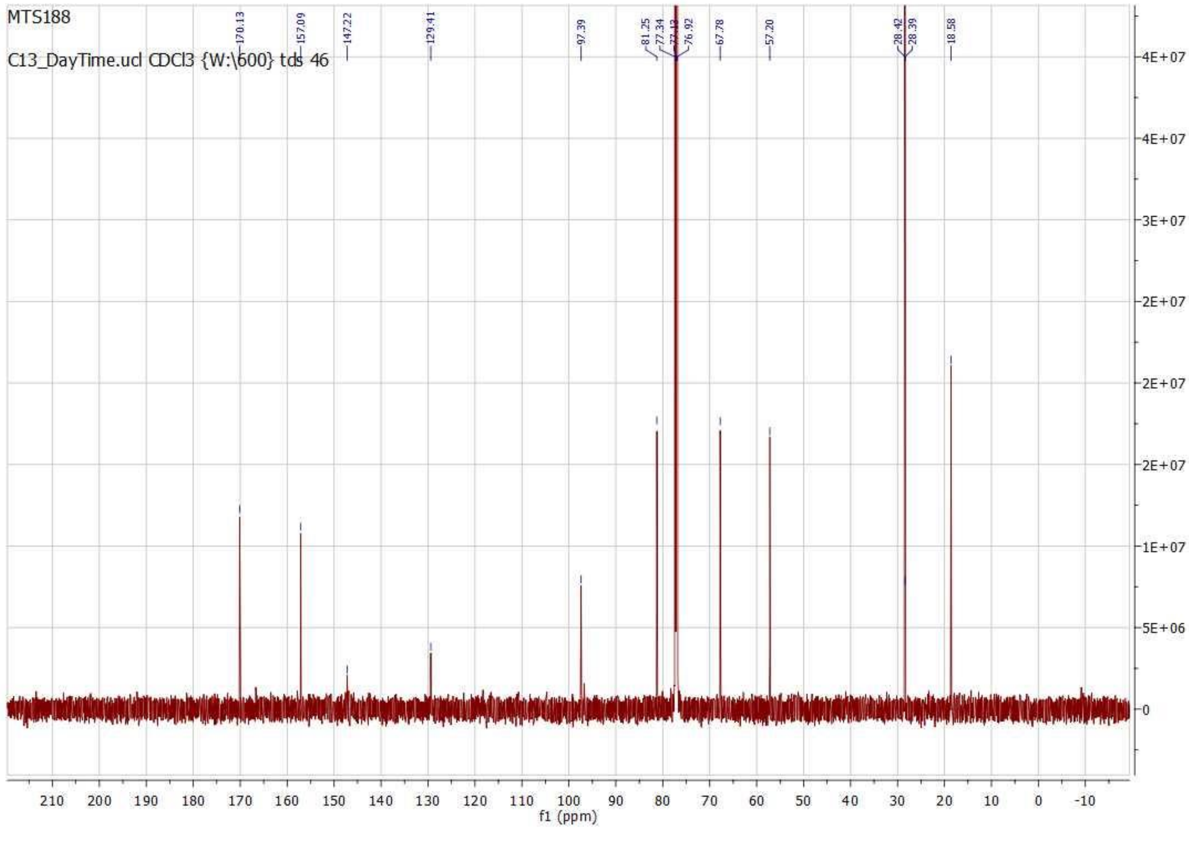

***Tert*-butyl (2-(benzylamino)-2-oxoethyl)carbamate (36)**

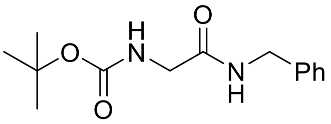

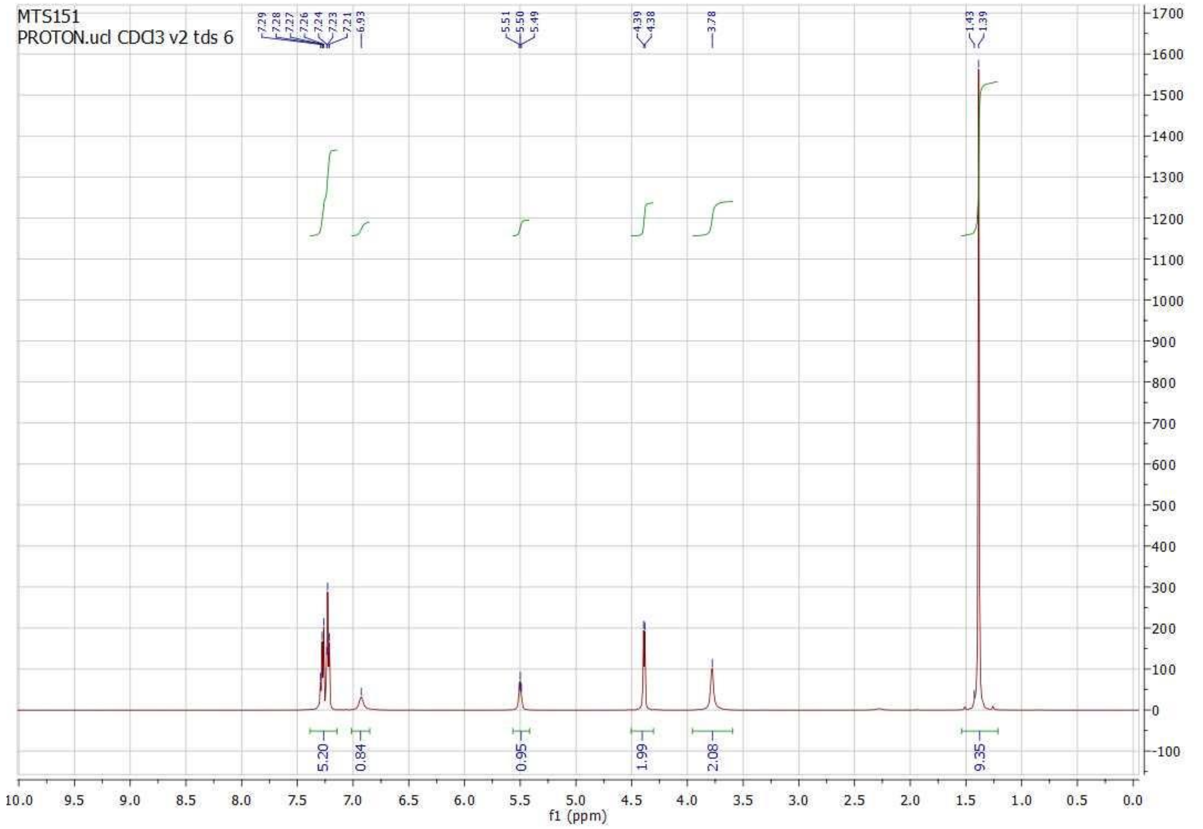

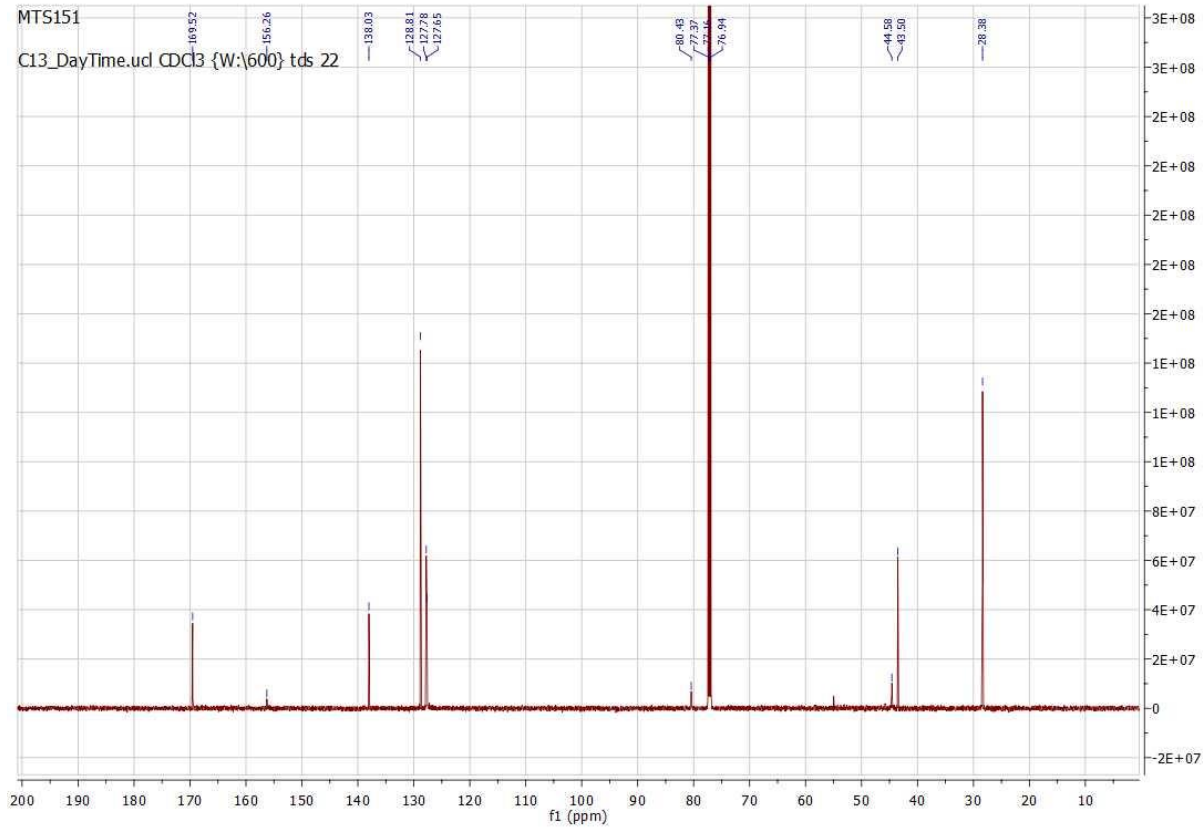

***tert*-butyl (*S*)-(1-(benzylamino)-1-oxopropan-2-yl)carbamate (37)**

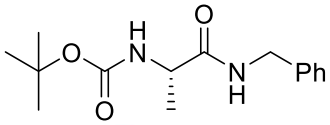

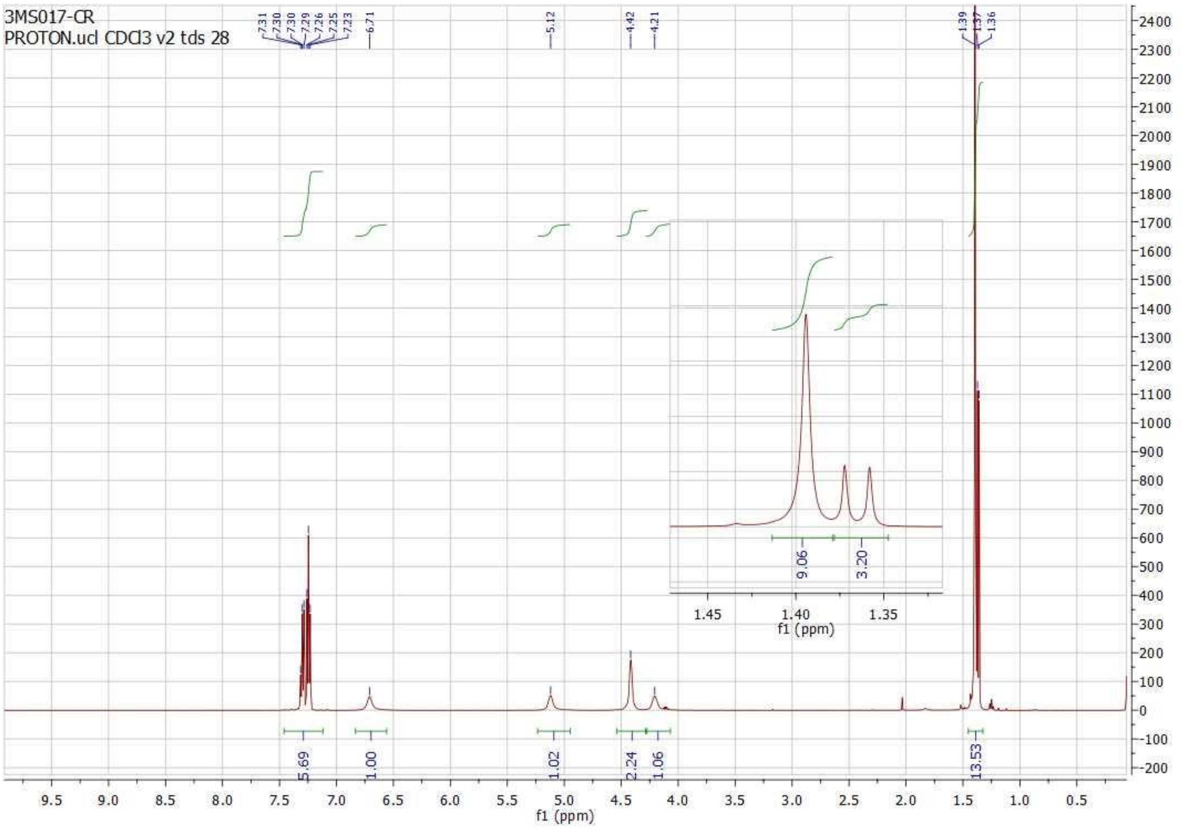

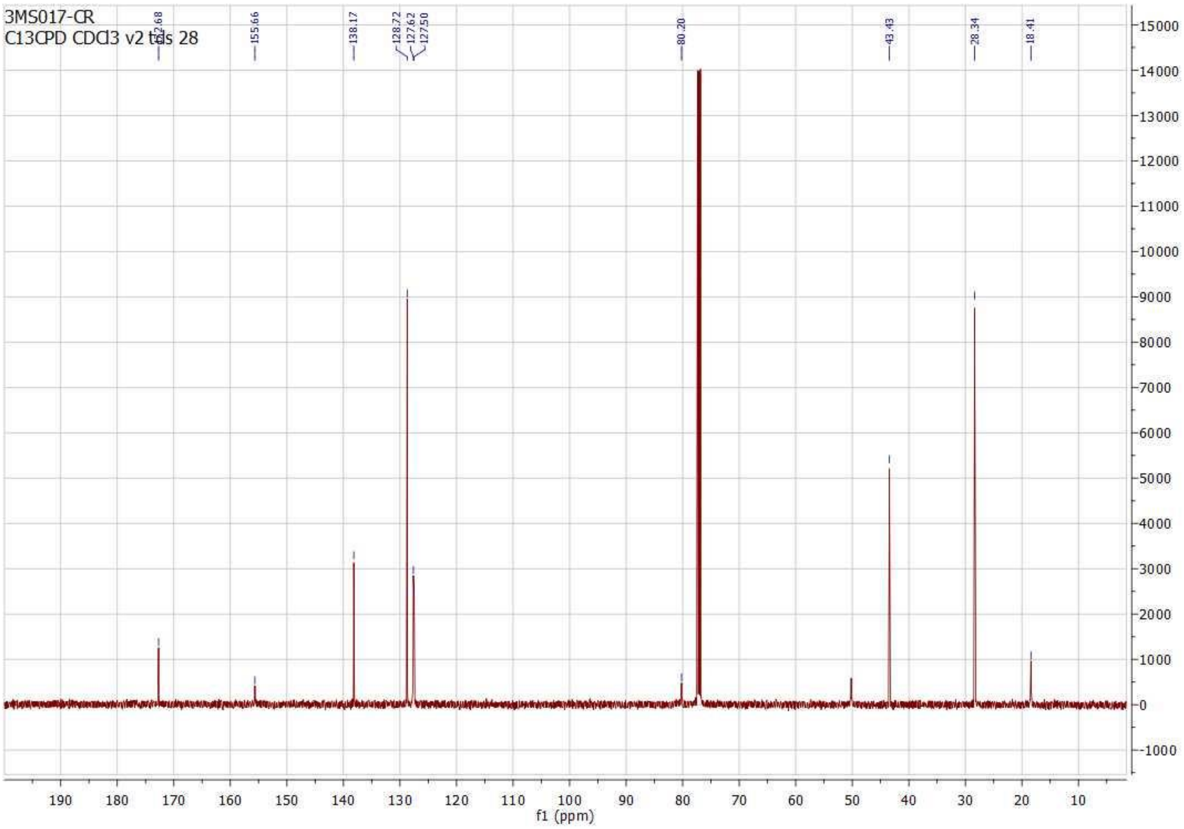

***tert*-butyl ((*2S,3R*)-1-(benzylamino)-3-hydroxy-1-oxobutan-2-yl)carbamate (38)**

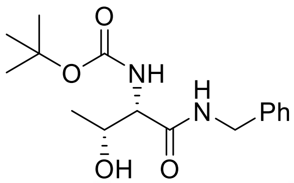

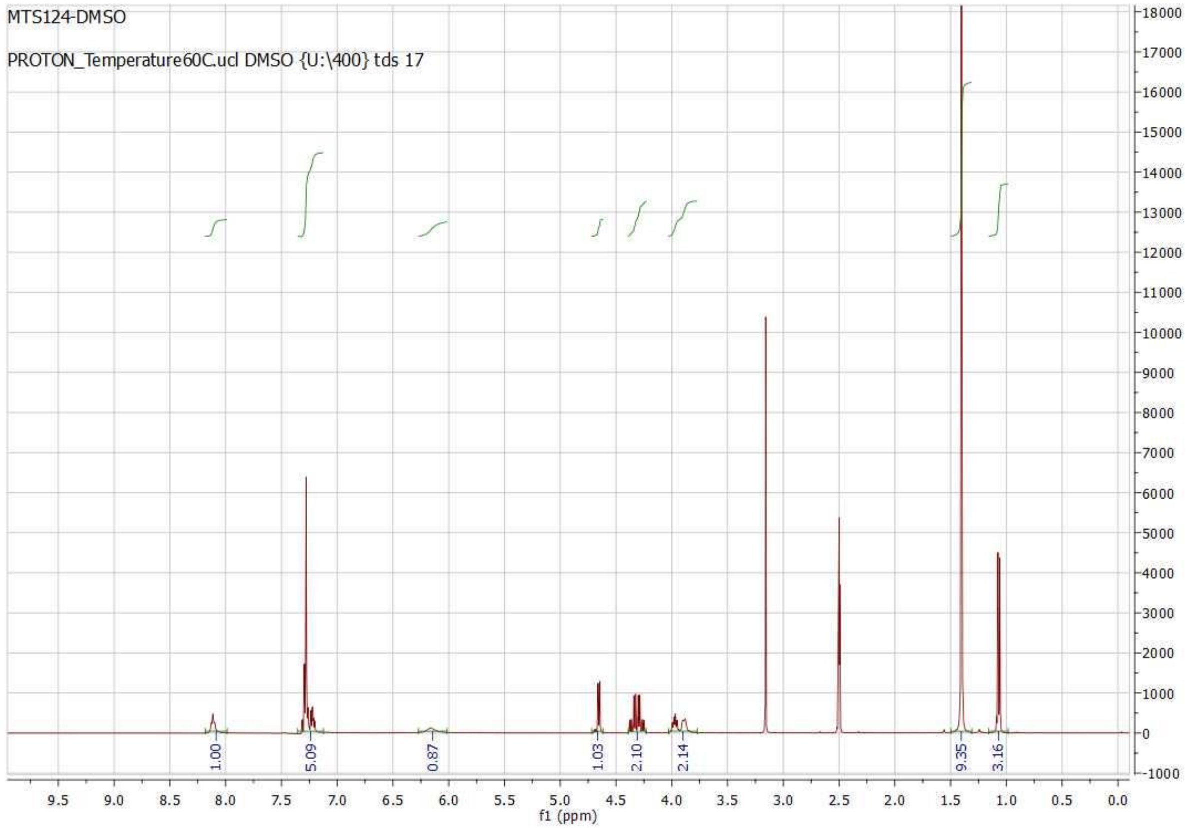

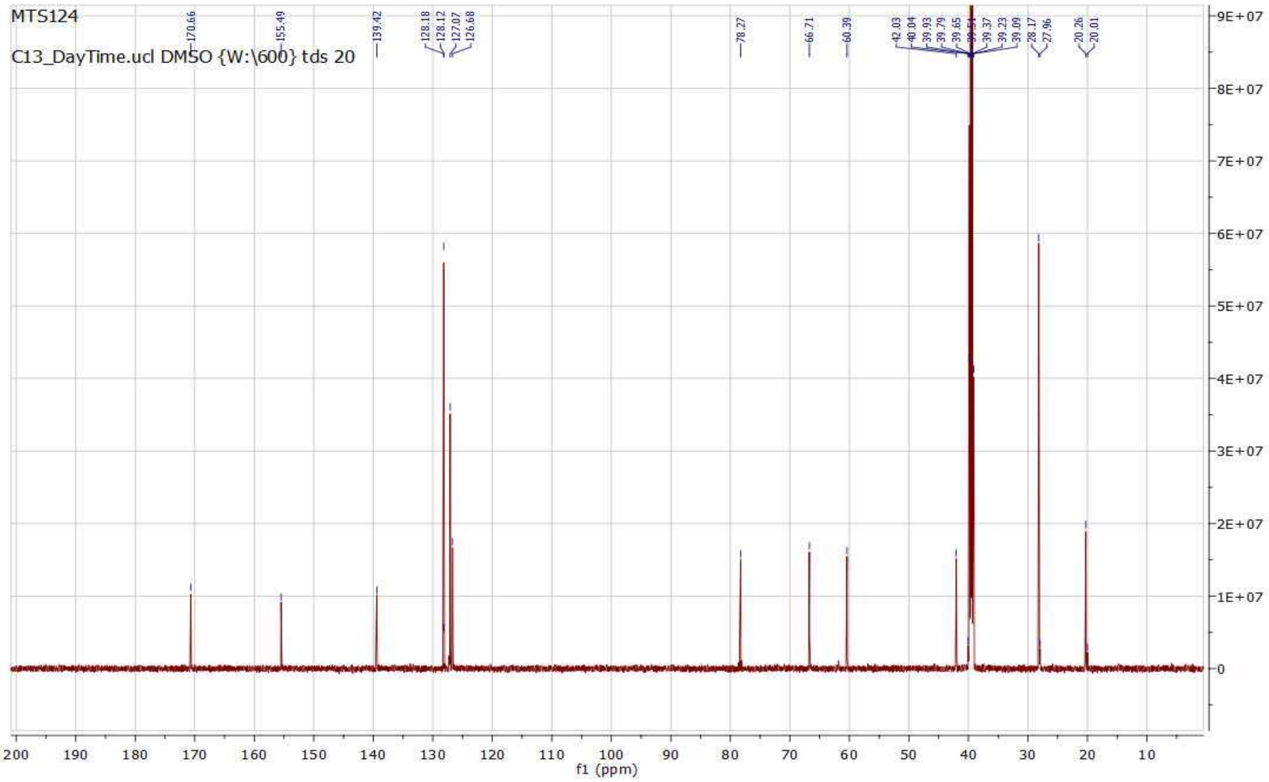

***tert*-butyl (*S*)-2-(benzylcarbamoyl)pyrrolidine-1-carboxylate (*39*)**

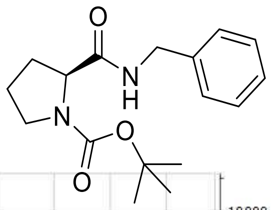

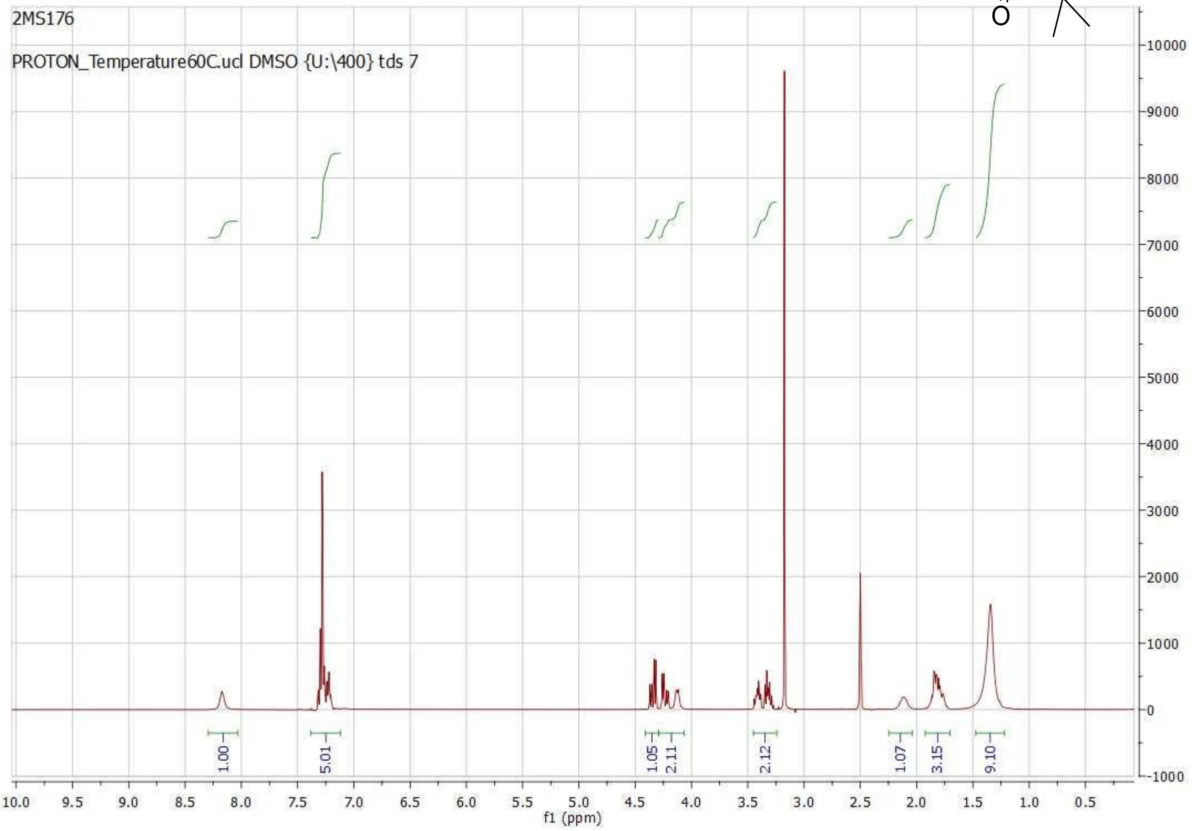

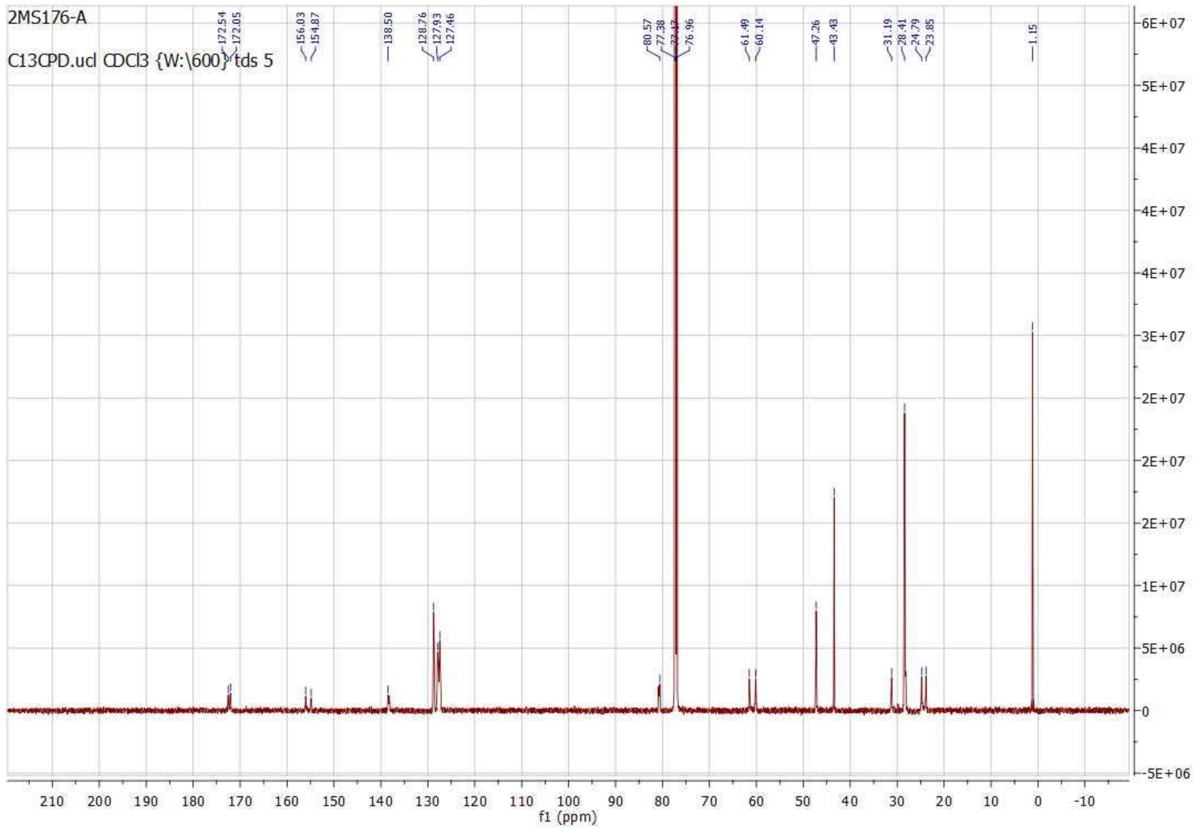

benzyl (*tert*-butoxycarbonyl)-L-phenylalanyl-L-valinate *(40)*

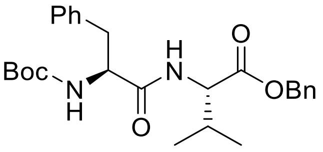

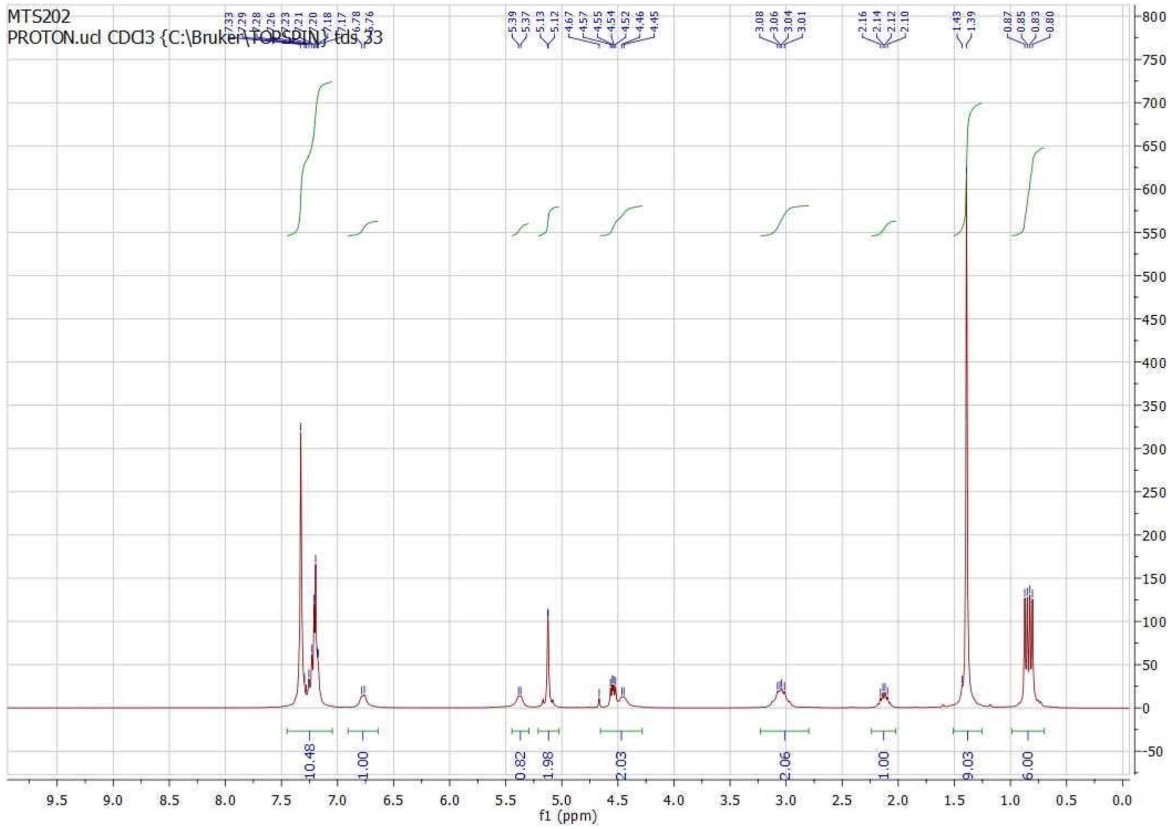

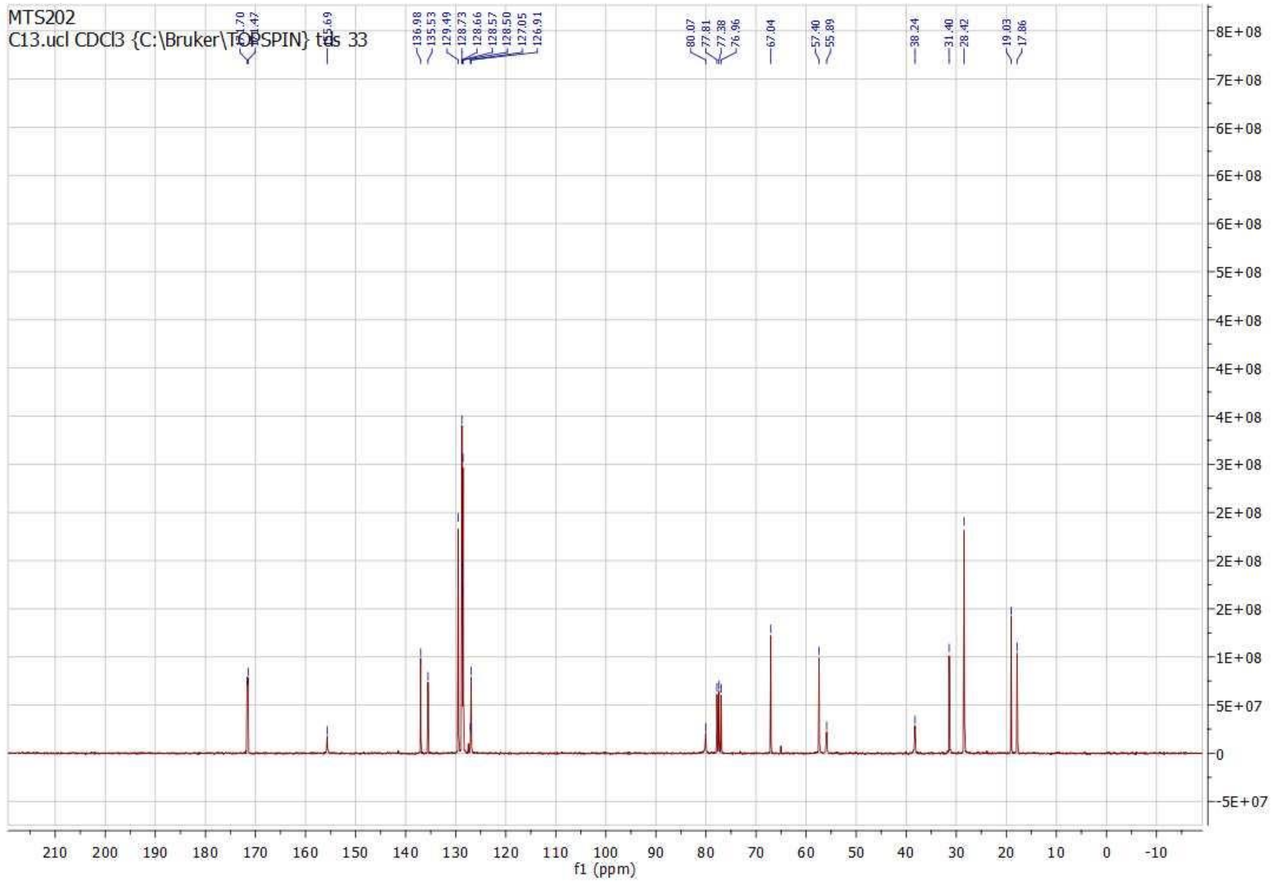

***tert*-butyl (*tert*-butoxycarbonyl)-D-alanyl-L-valinate (41)**

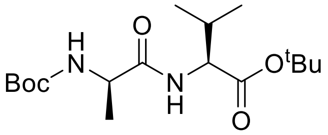

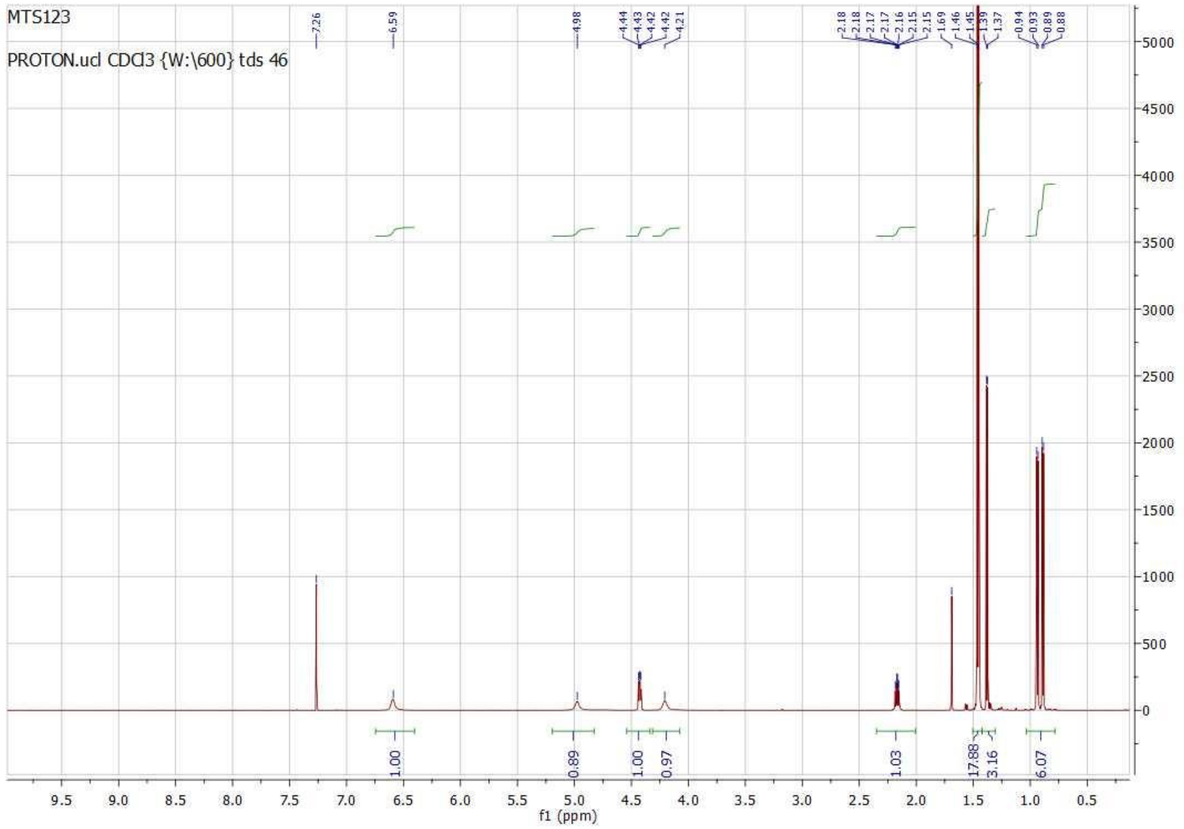

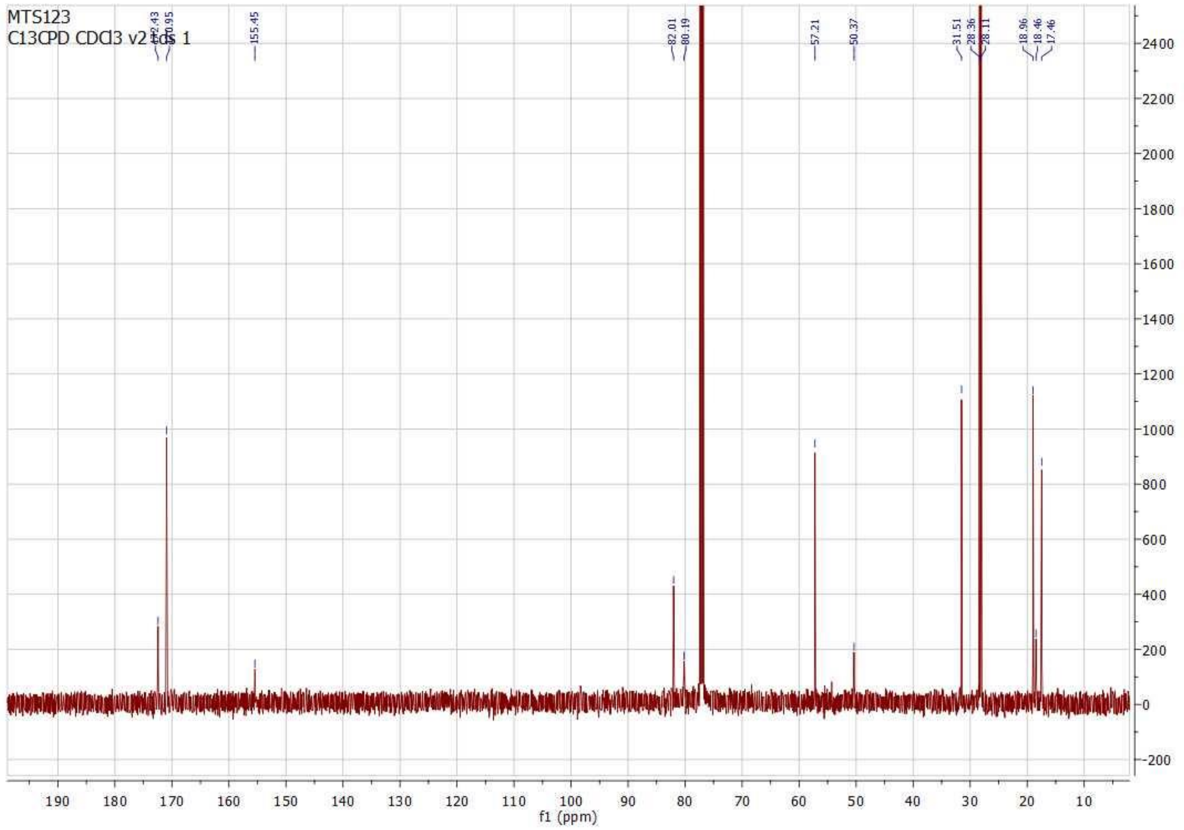

tert-butyl (_tert_-butoxycarbonyl)-D-alanyl-L-phenylalaninate (42)

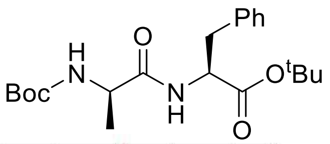

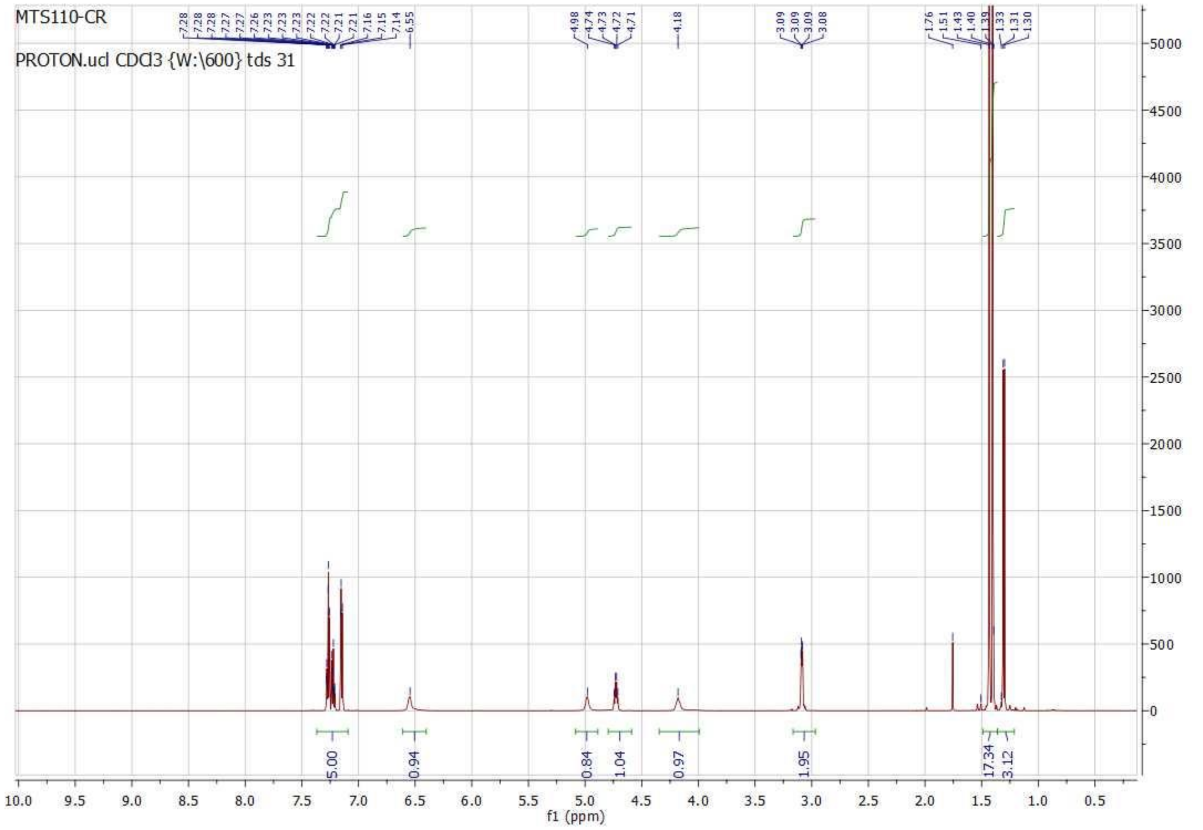

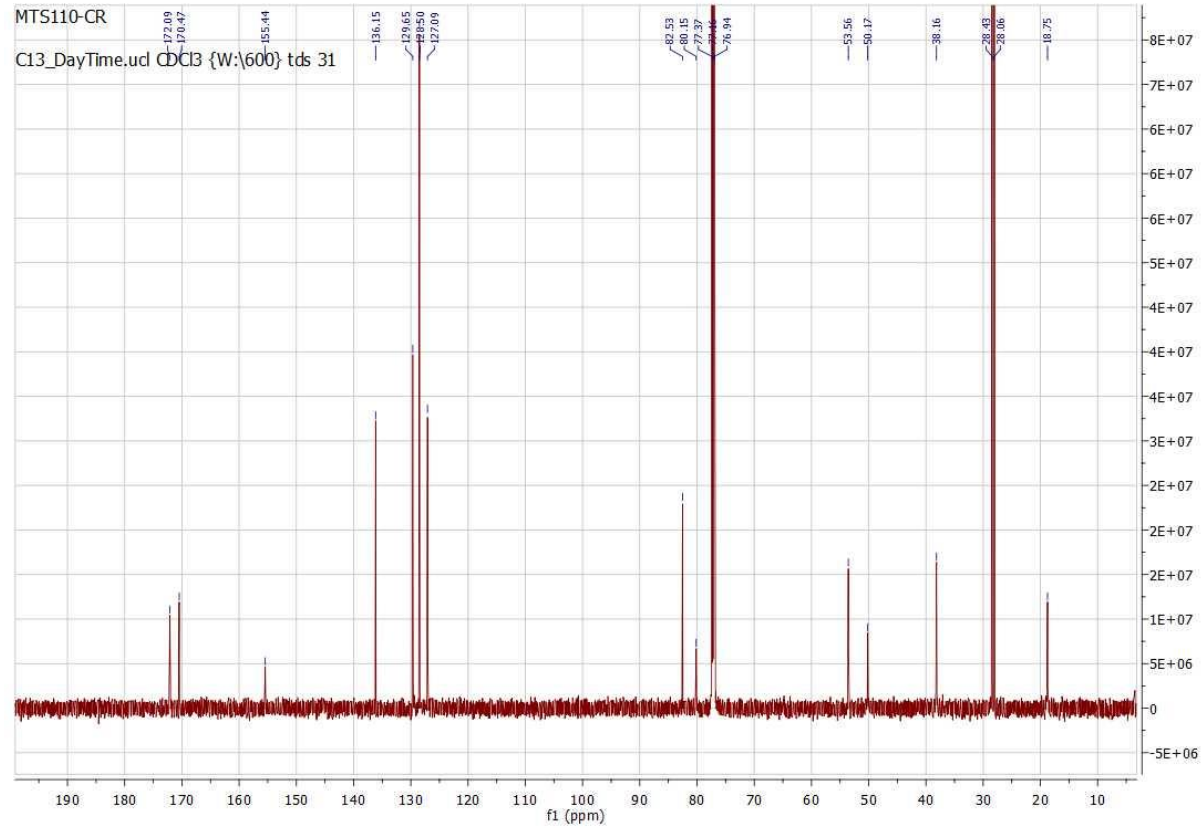

***tert*-butyl (*tert*-butoxycarbonyl)-L-threonyl-L-valinate (43)**

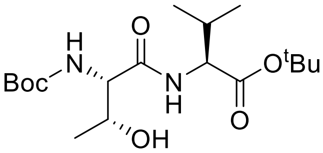

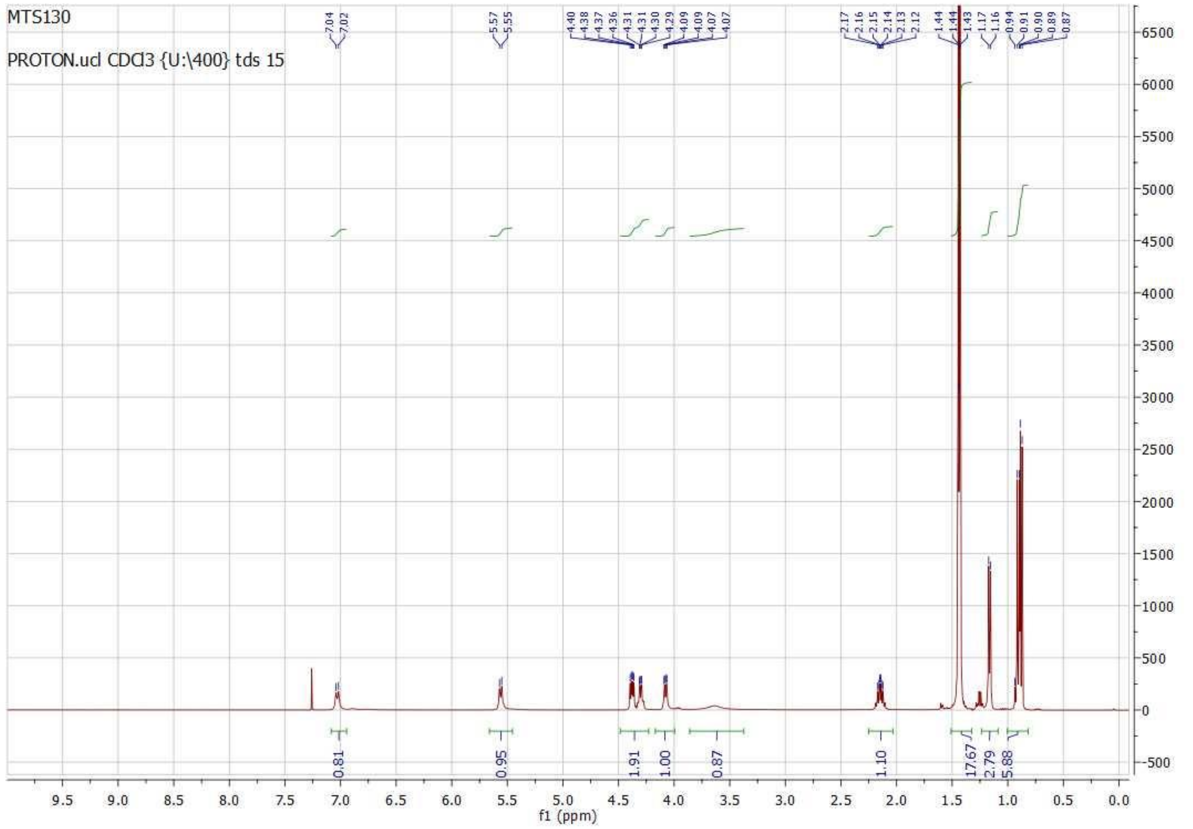

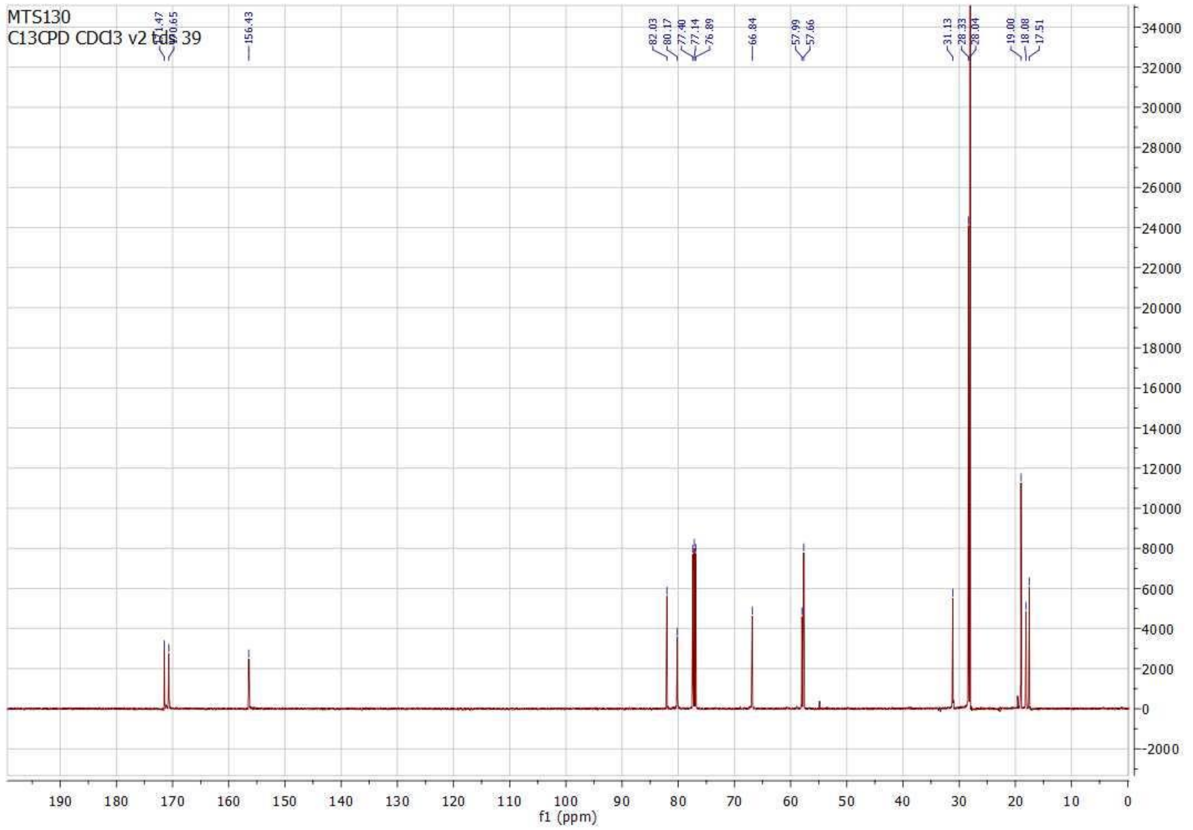

benzyl (*tert*-butoxycarbonyl)-L-threonyl-L-valinate (44)

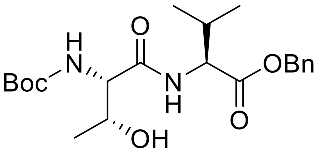

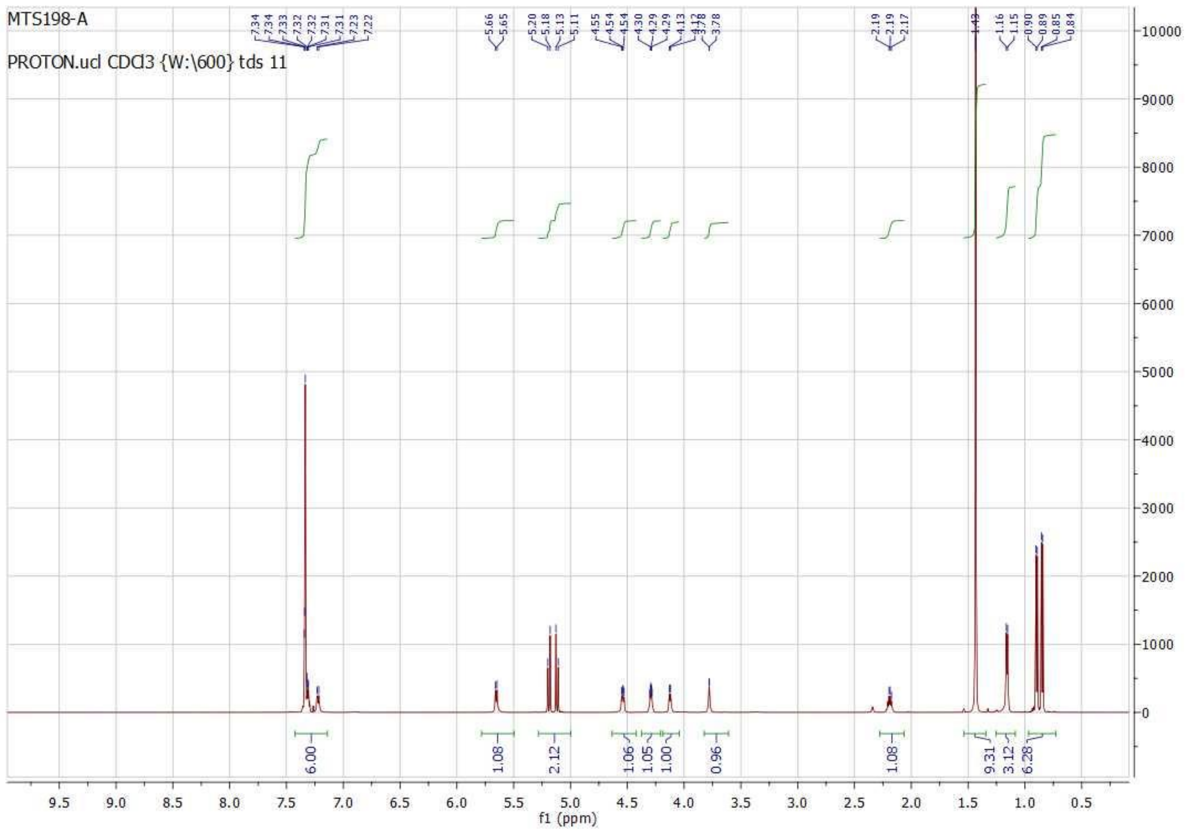

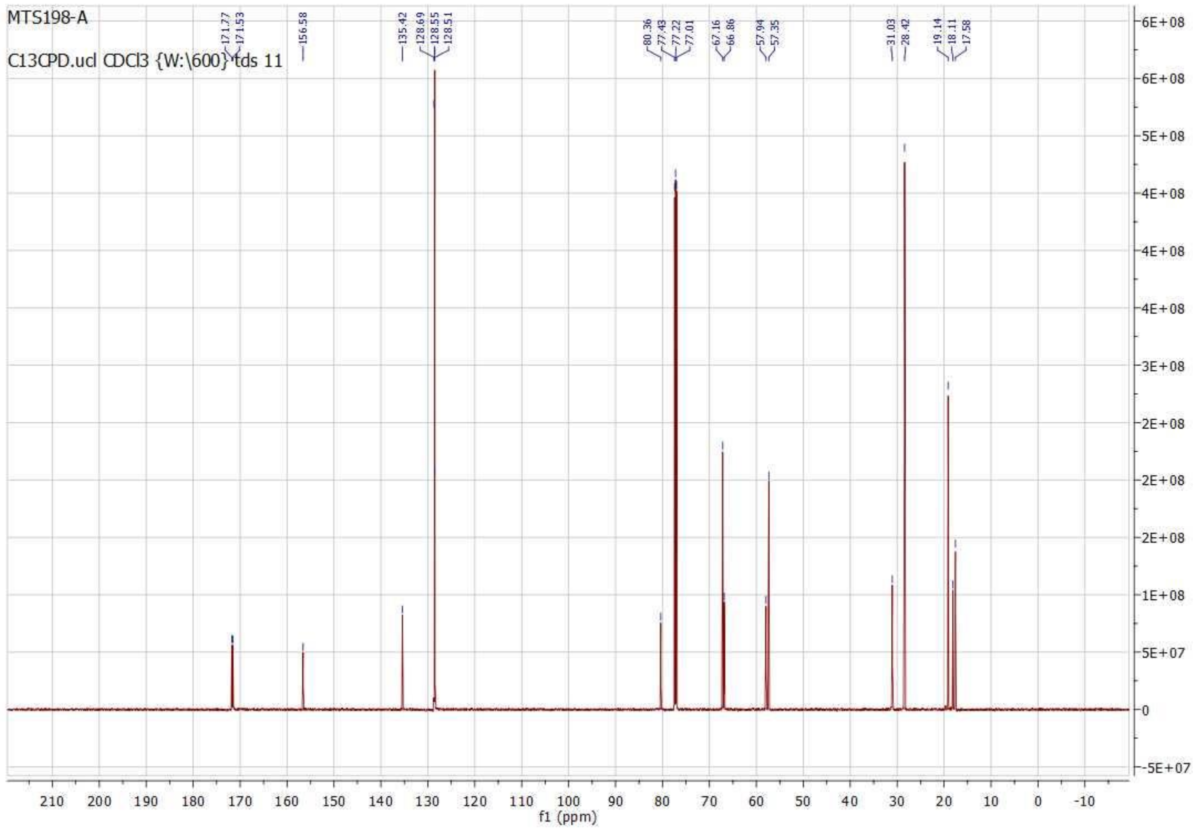

tert-butyl (tert-butoxycarbonyl)glycylglycylglycinate (45)

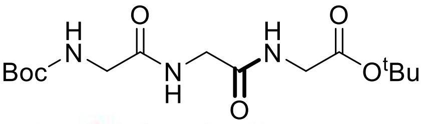

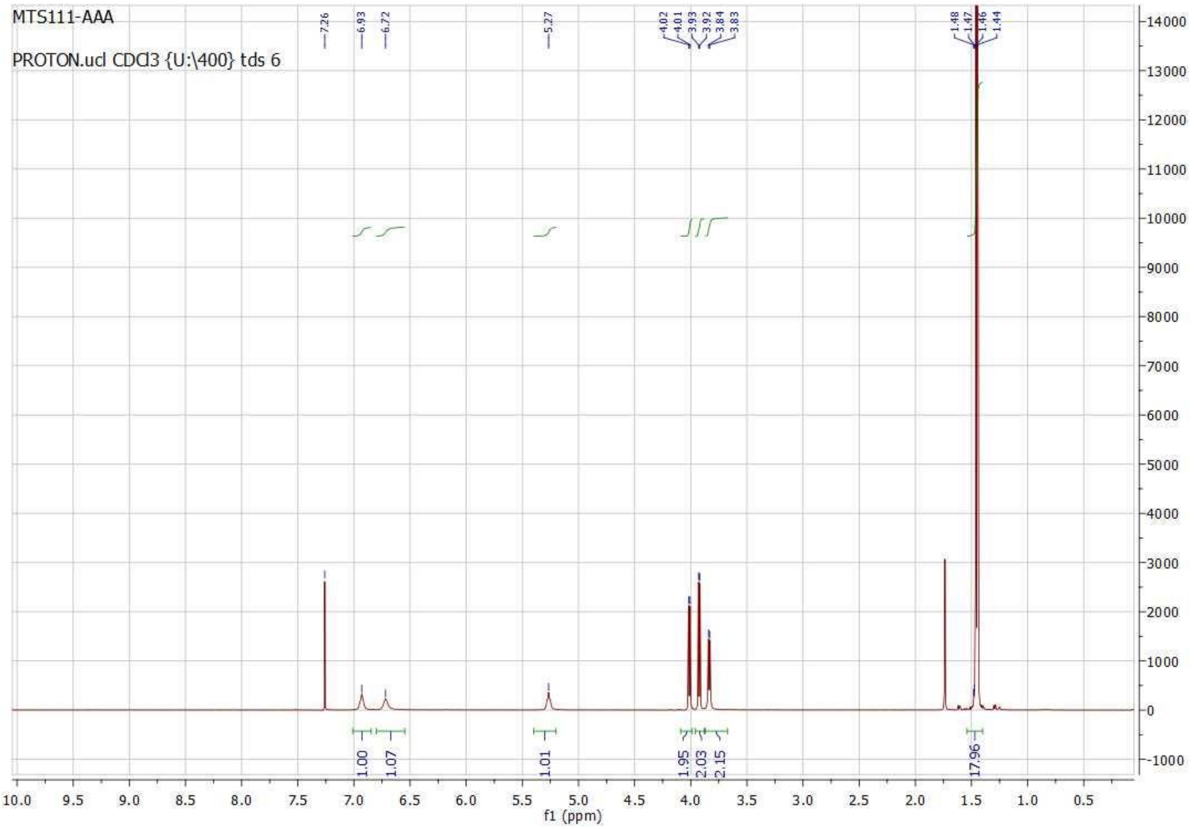

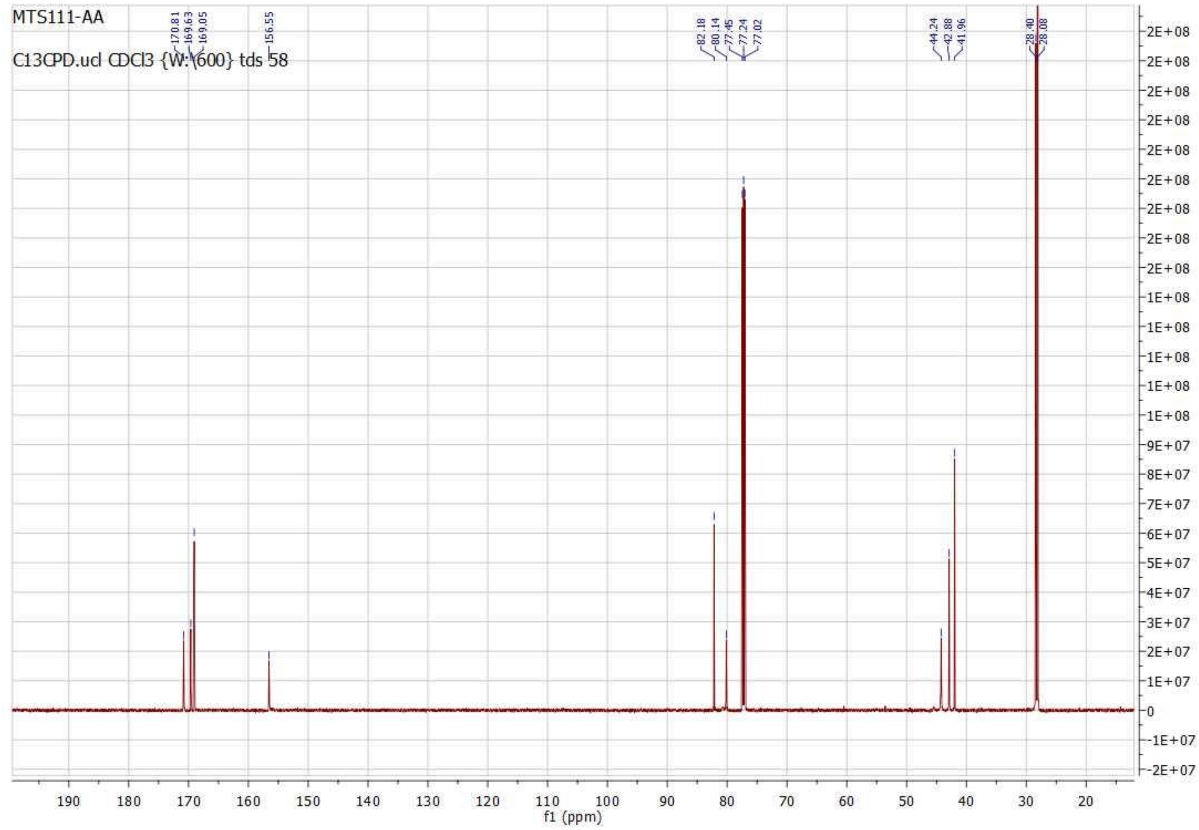

***(S)*-2-amino-N-benzyl-3-(1H-indol-3-yl)propanamide (46)**

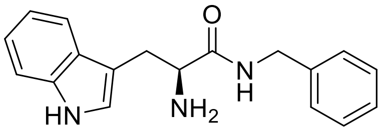

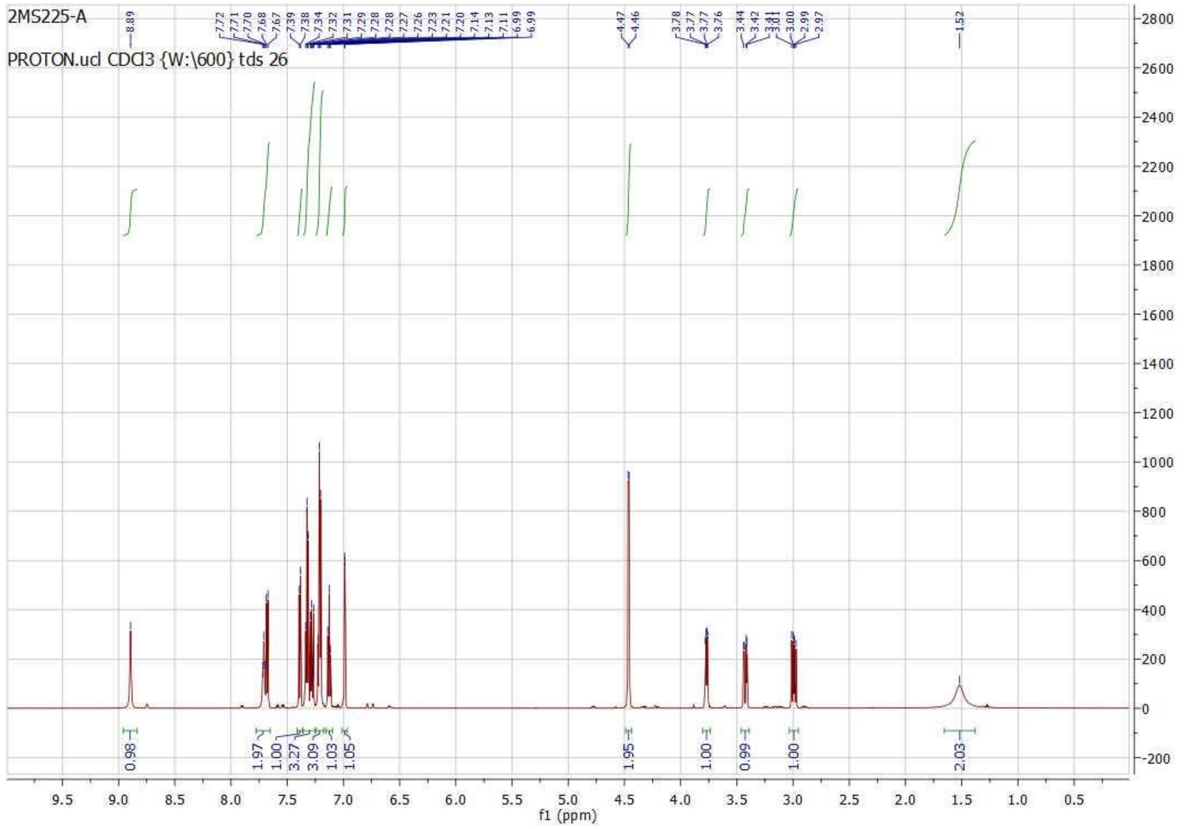

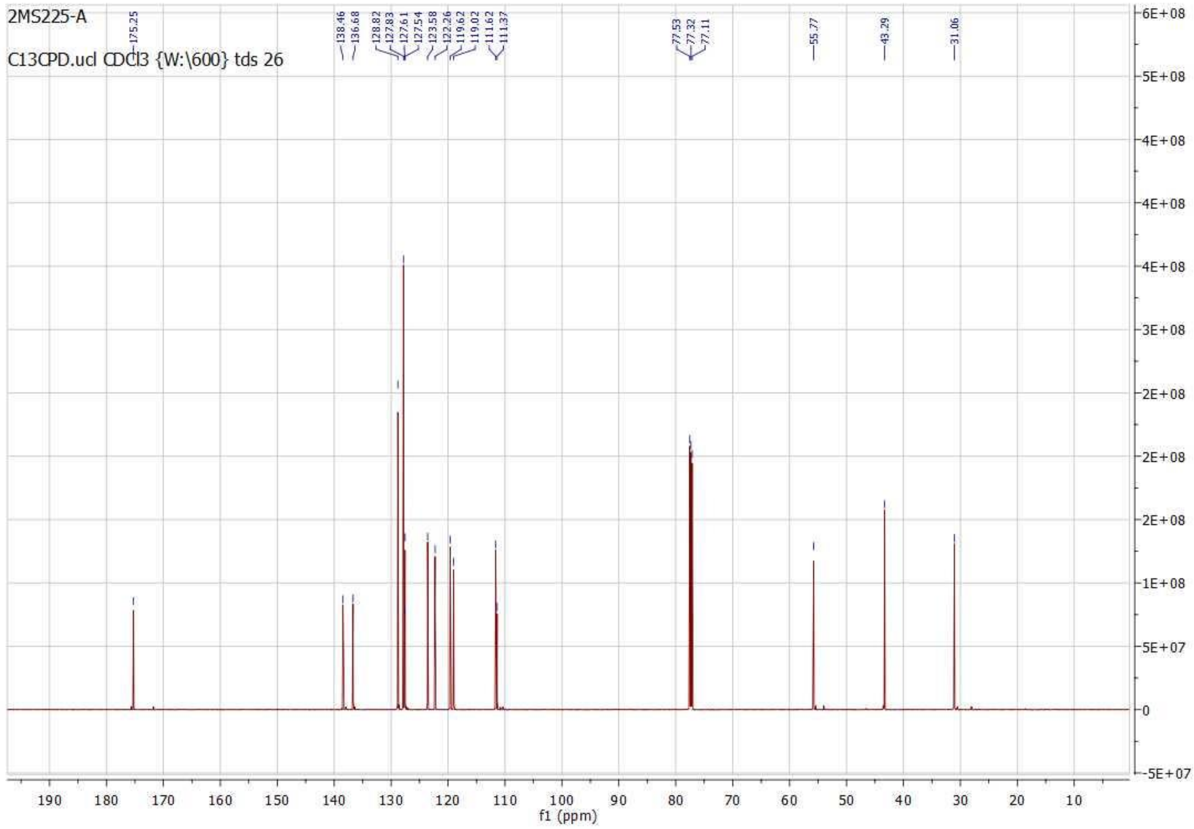

(S)-2-Amino-*N*-benzyl-3-methylbutanamide (47)

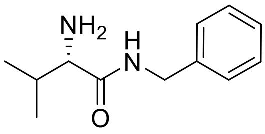

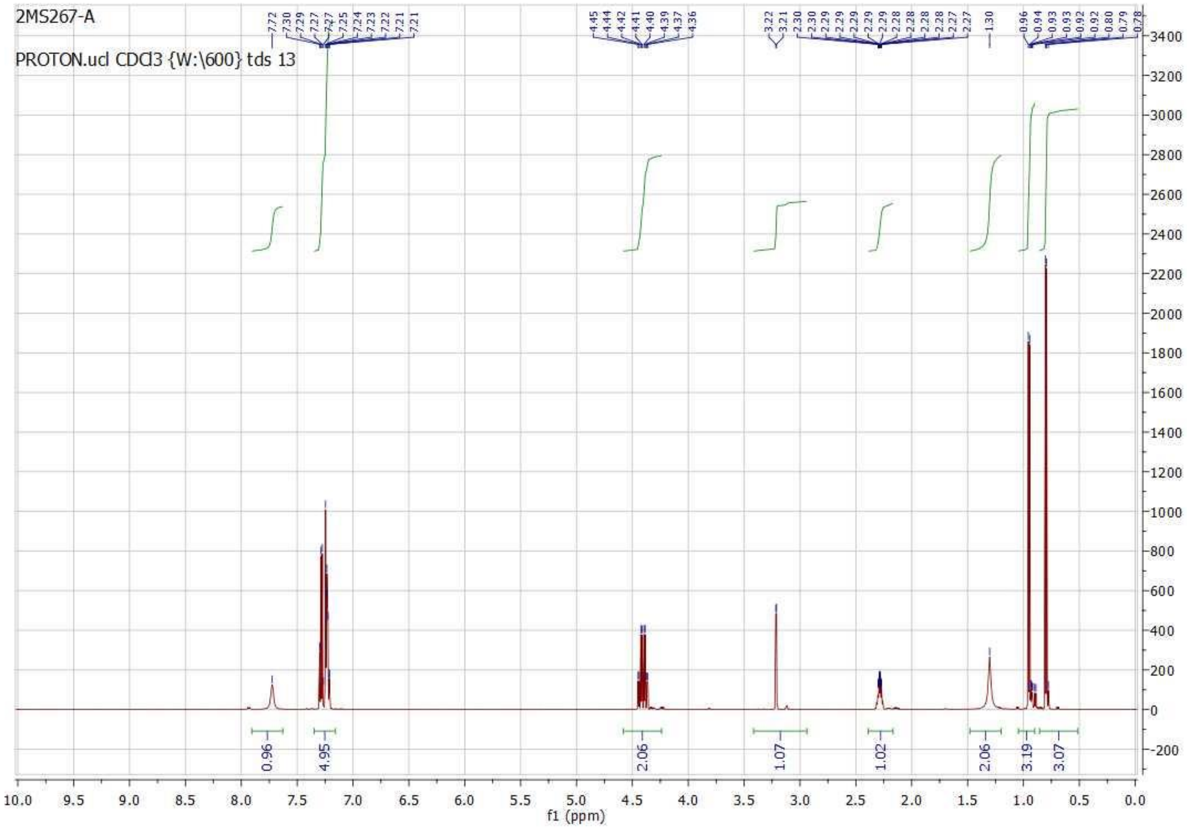

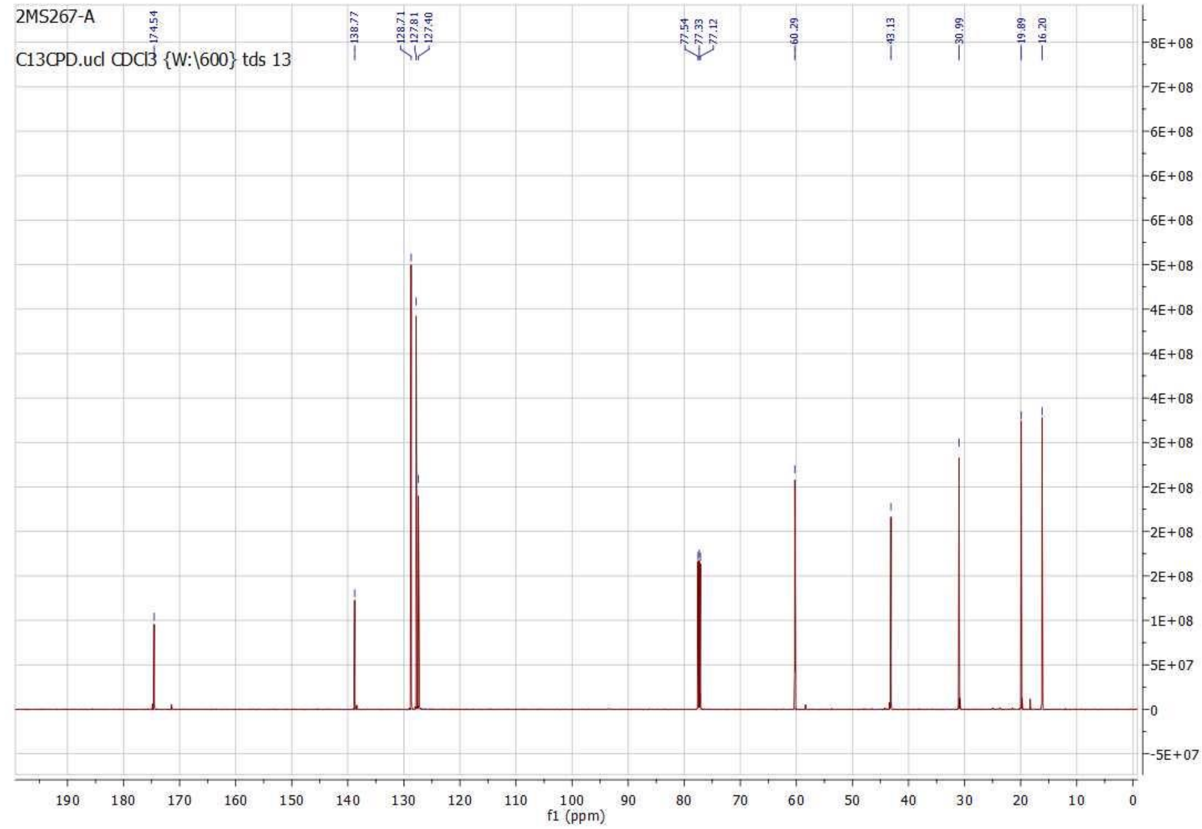

(*2S,3S*)-2-amino-N-benzyl-3-methylpentanamide (48)

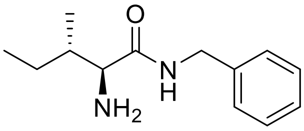

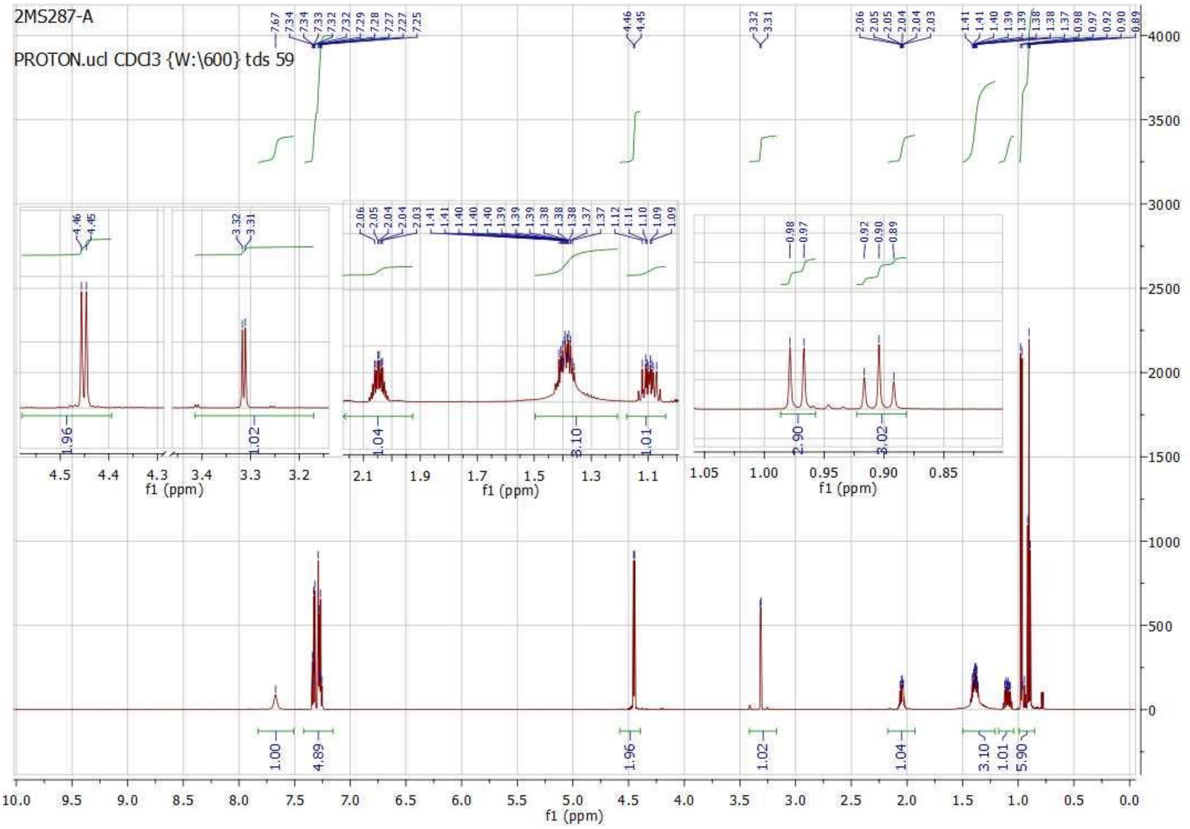

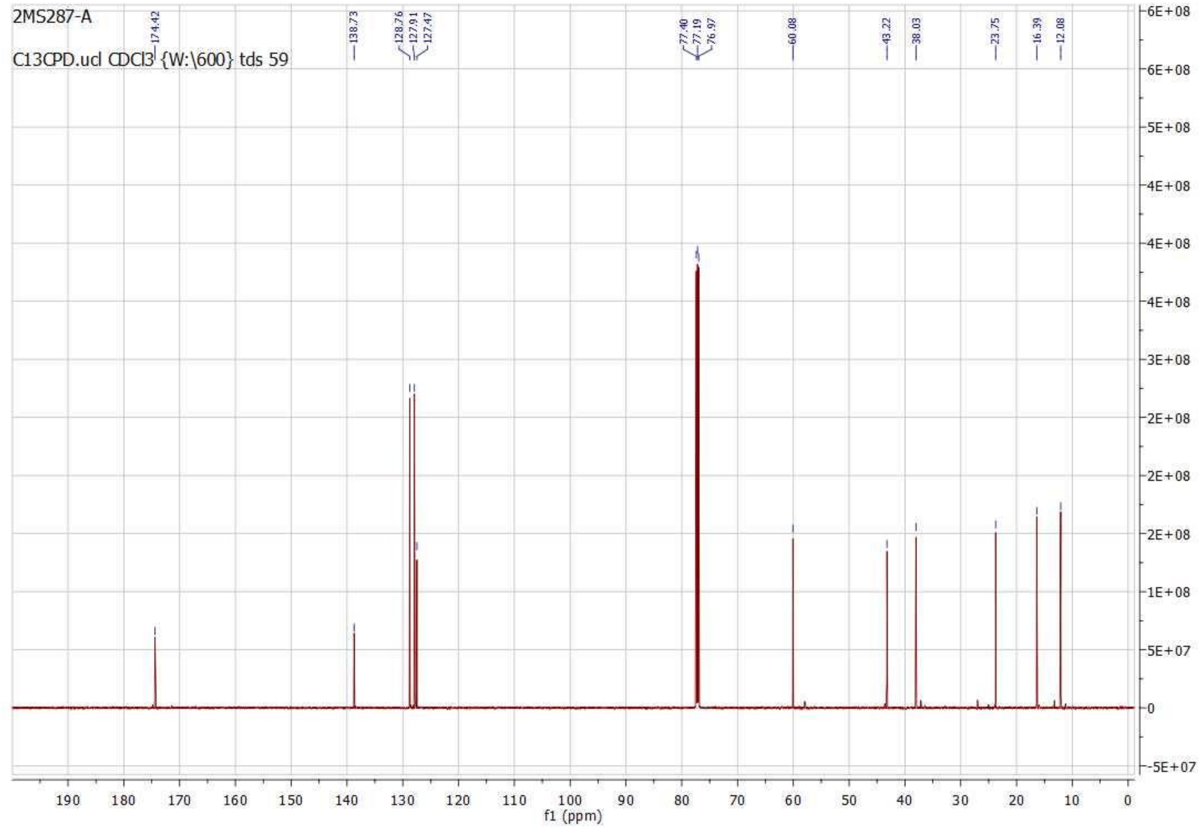

(*S*)-2-amino-N-benzyl-4-(methylthio)butanamide (49)

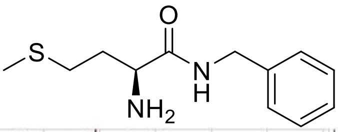

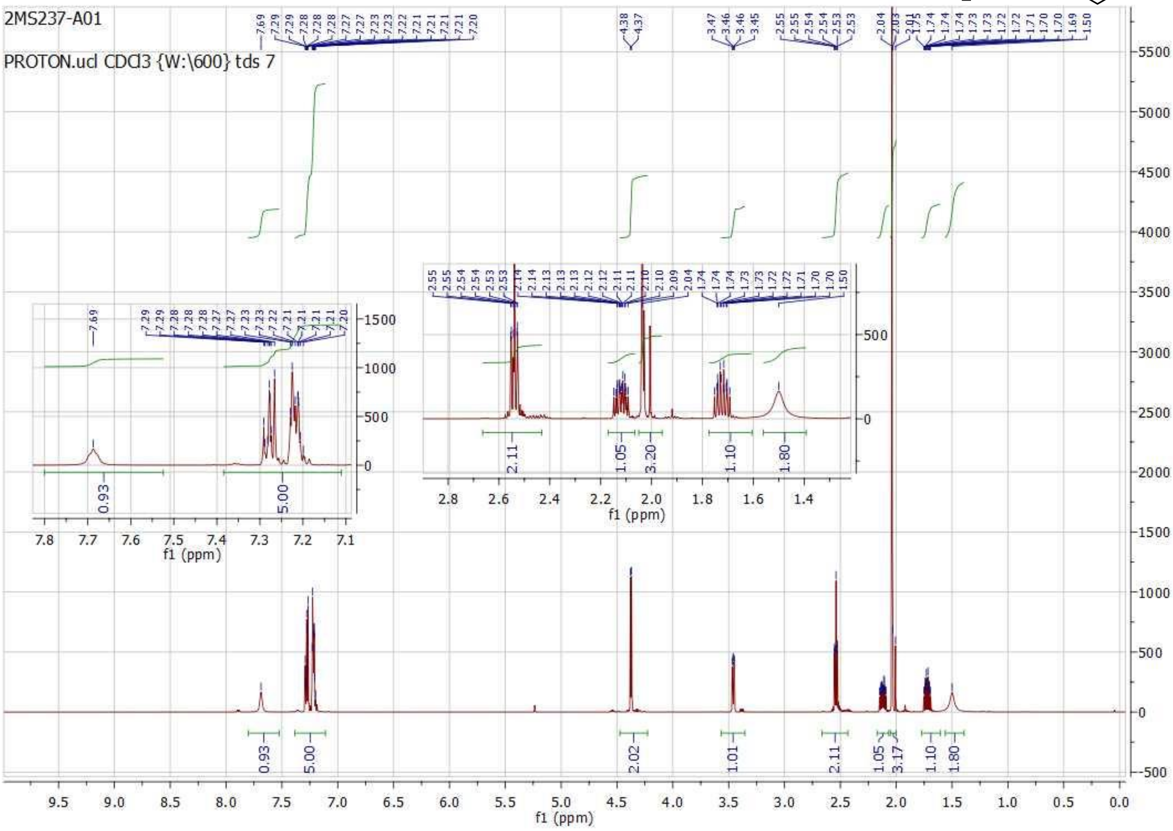

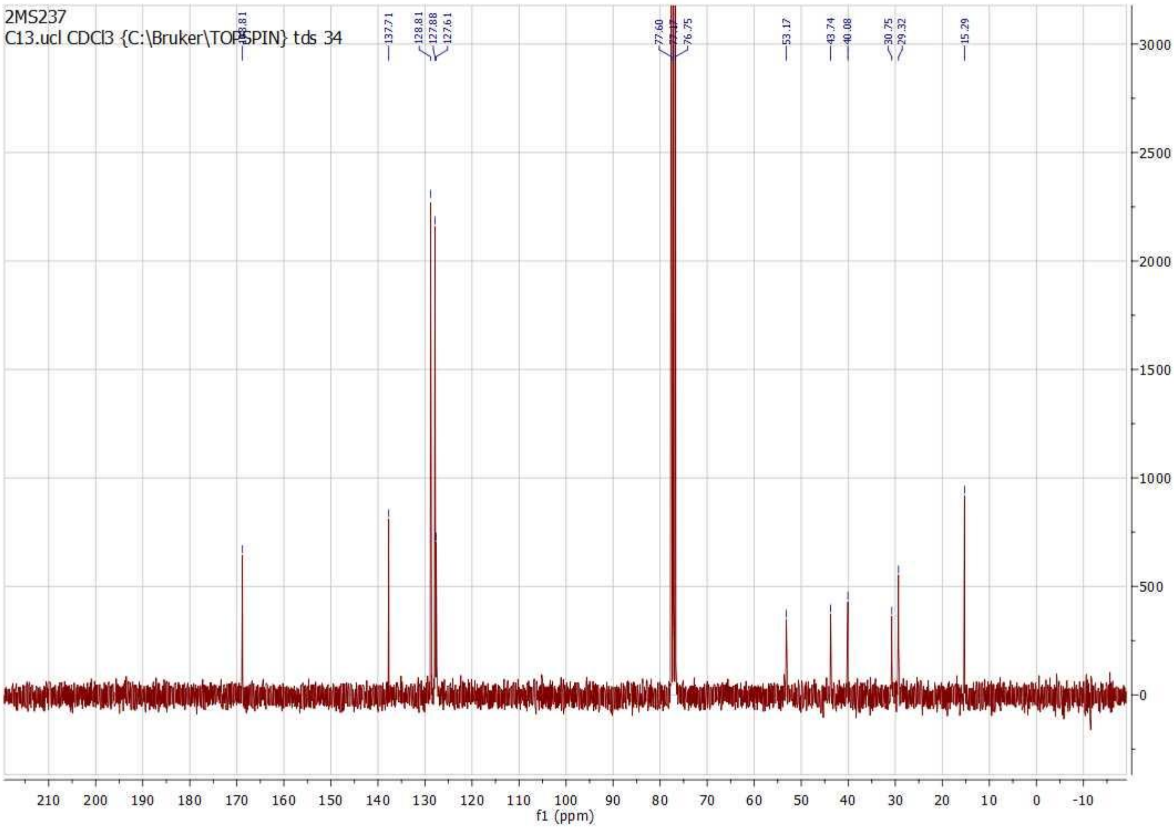

(*S*)-2-amino-N-benzyl-4-methylpentanamide (50)

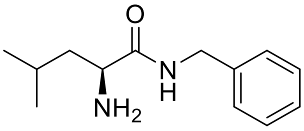

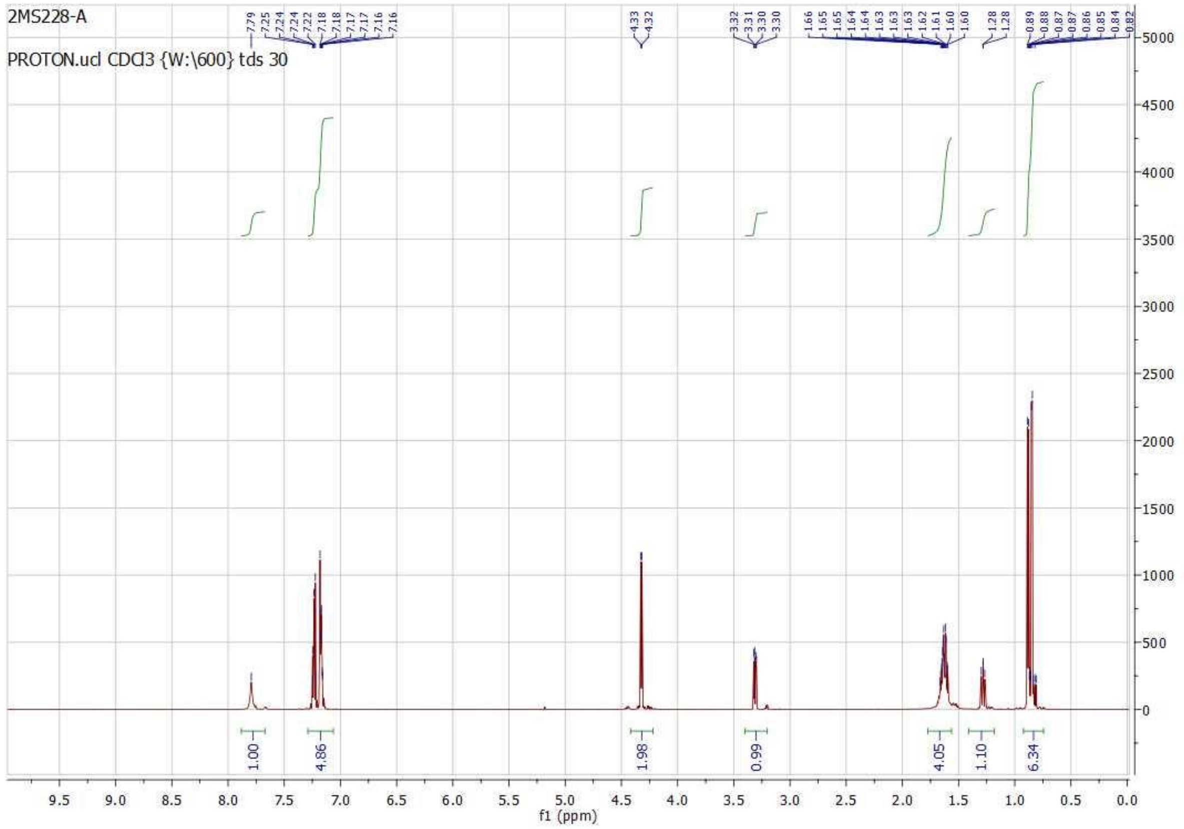

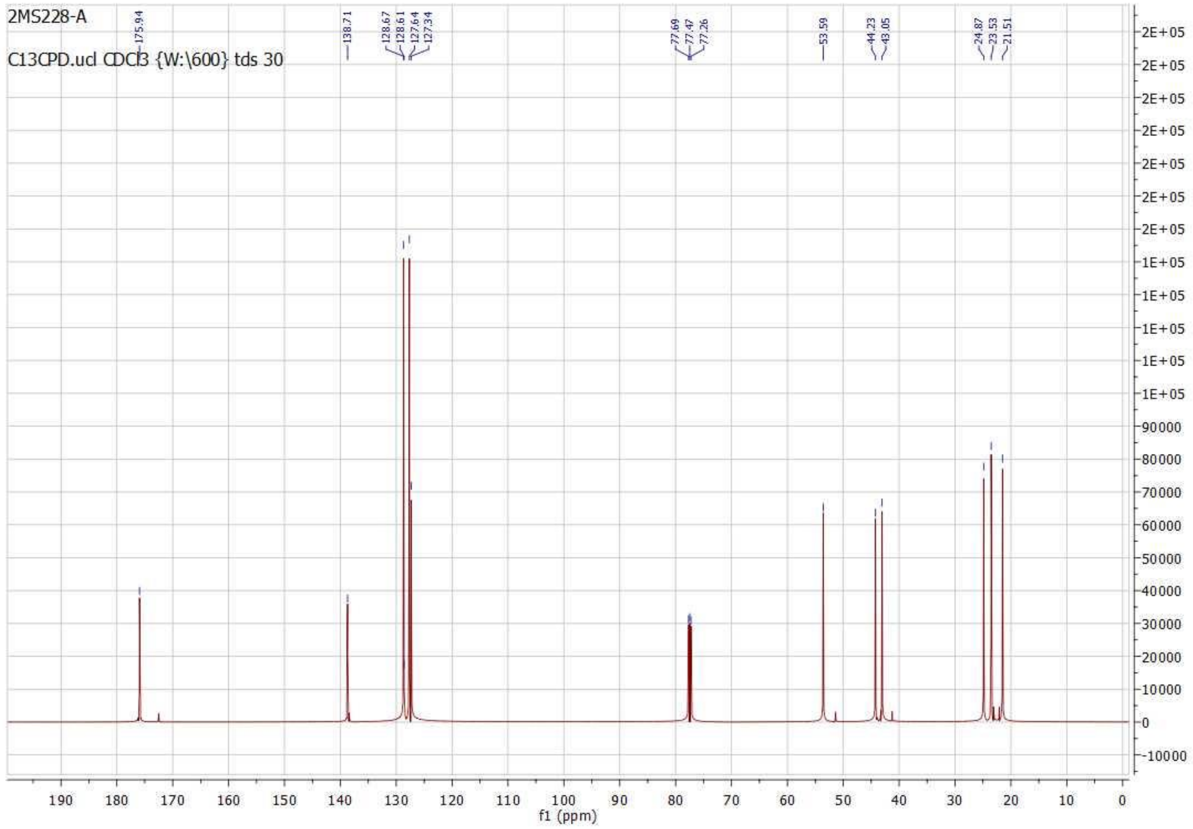

(S)-2-amino-*N*-benzyl-3-phenylpropanamide (51)

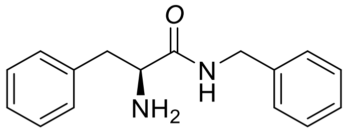

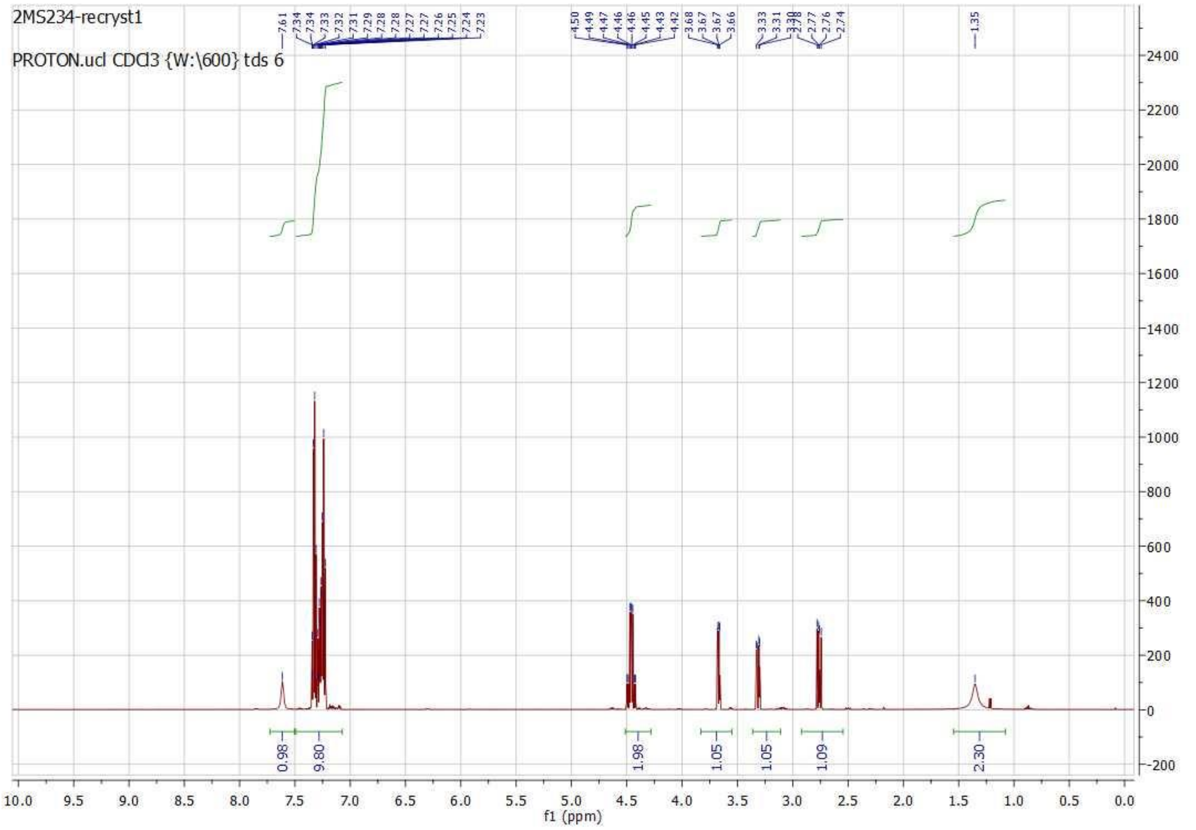

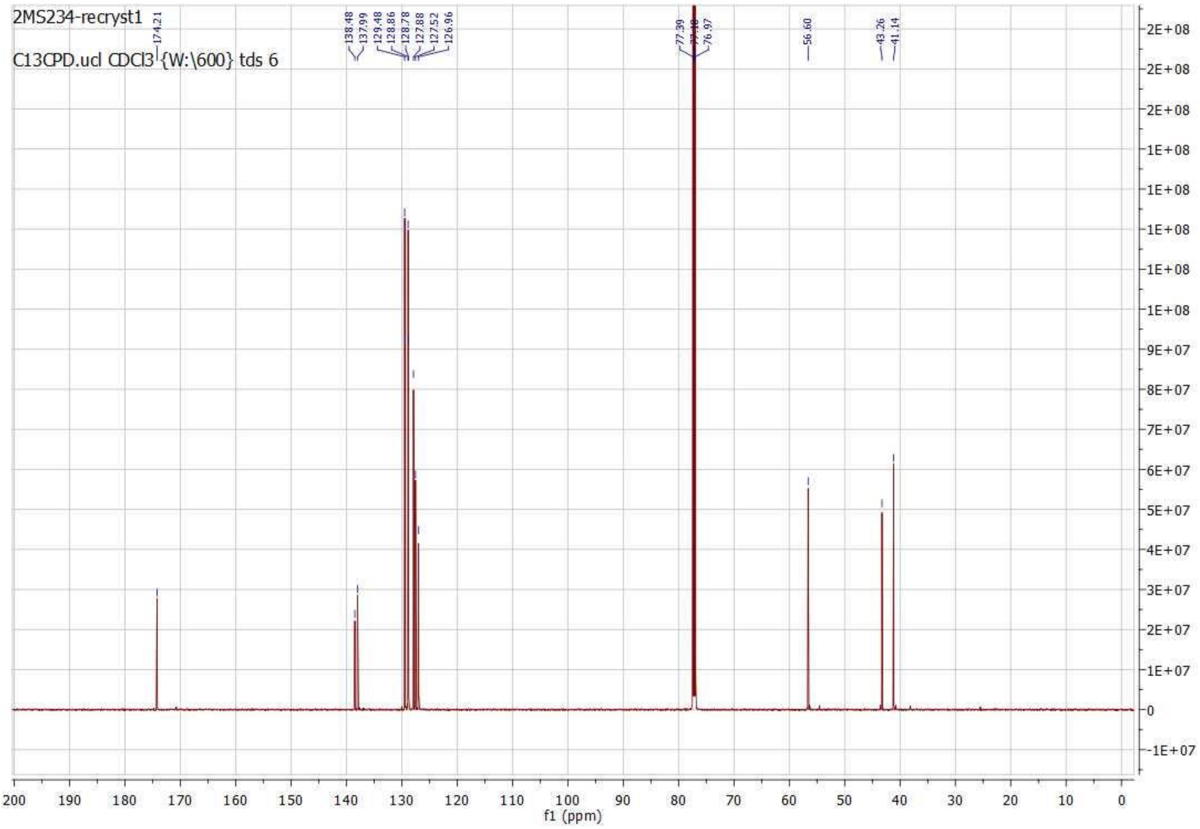

(*2S,3R*)-2-amino-N-benzyl-3-hydroxybutanamide (52)

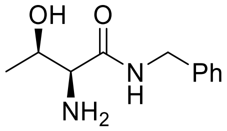

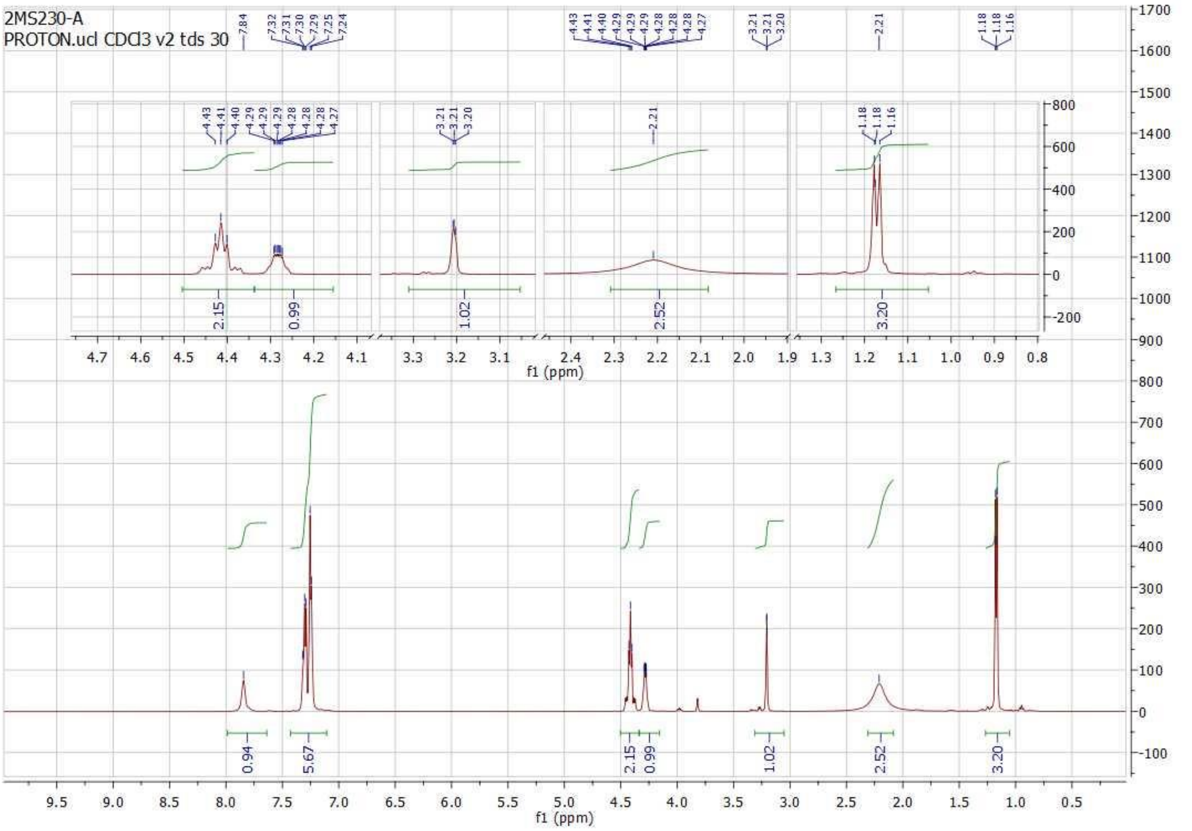

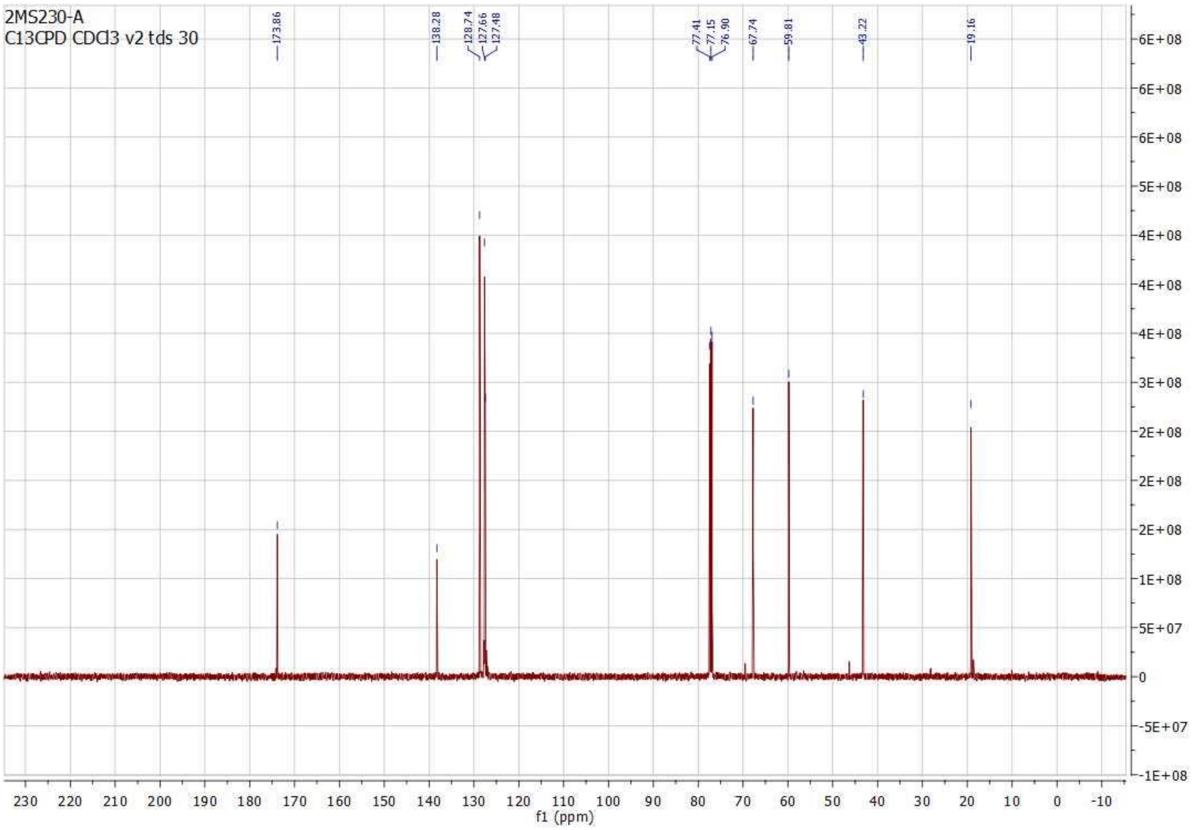

***(S)*-2-amino-N-benzylbutanamide (53)**

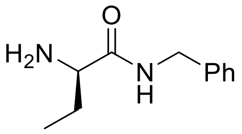

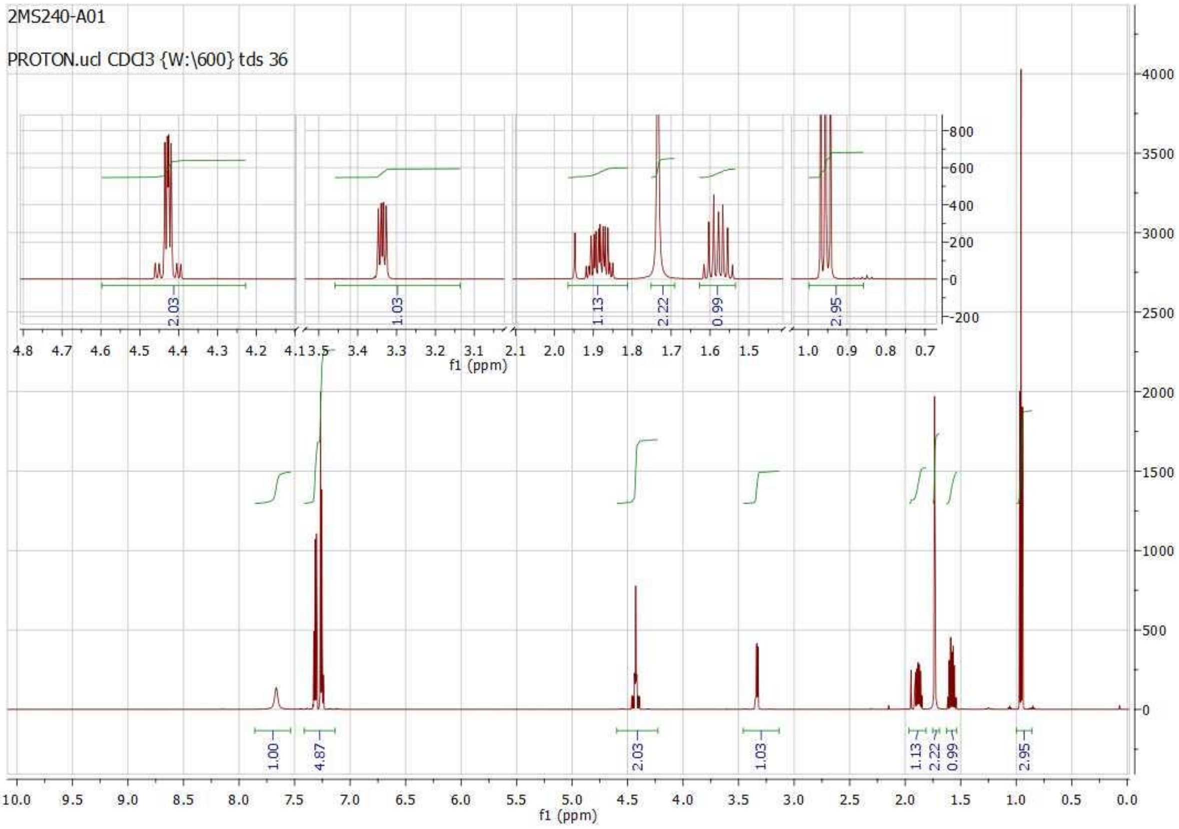

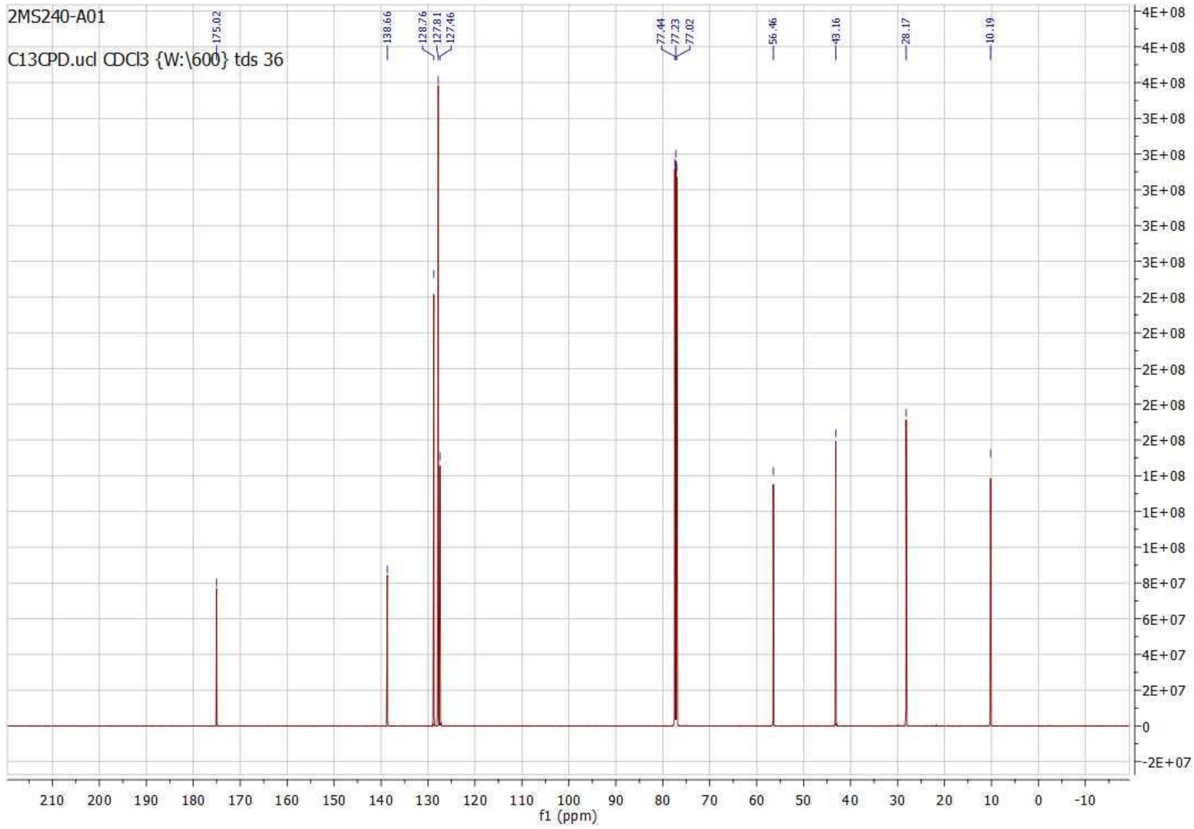

(*S*)-N-benzylpyrrolidine-2-carboxamide (54)

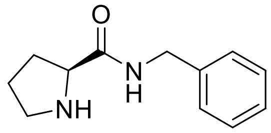

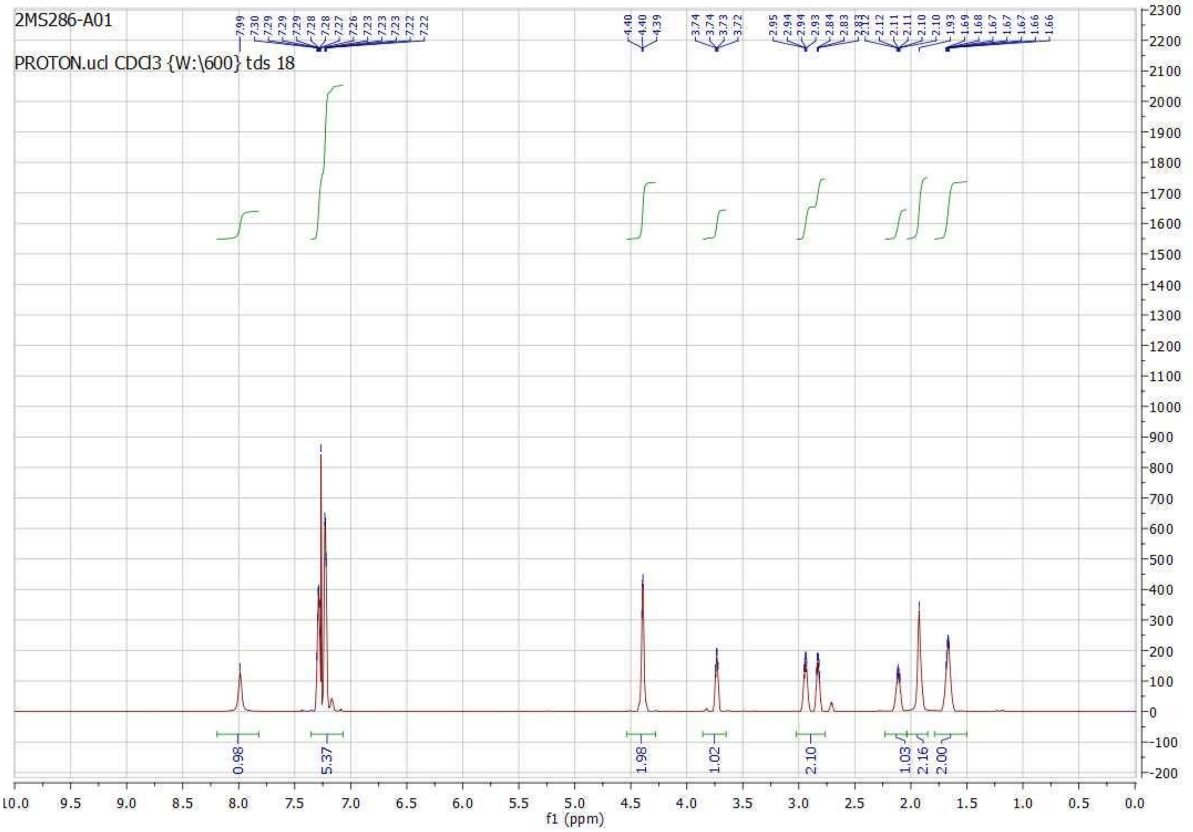

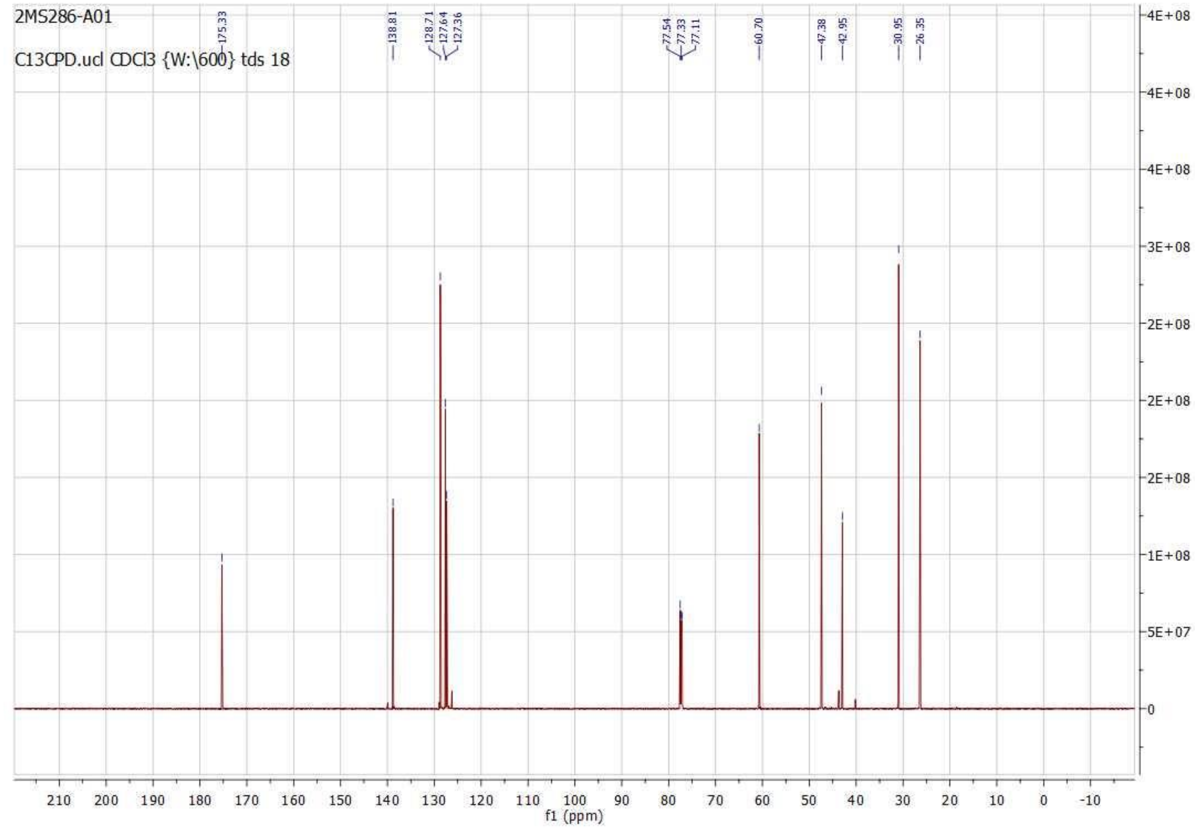

3-amino-N-benzylbutanamide (55)

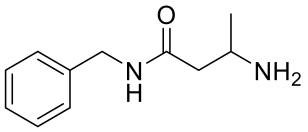

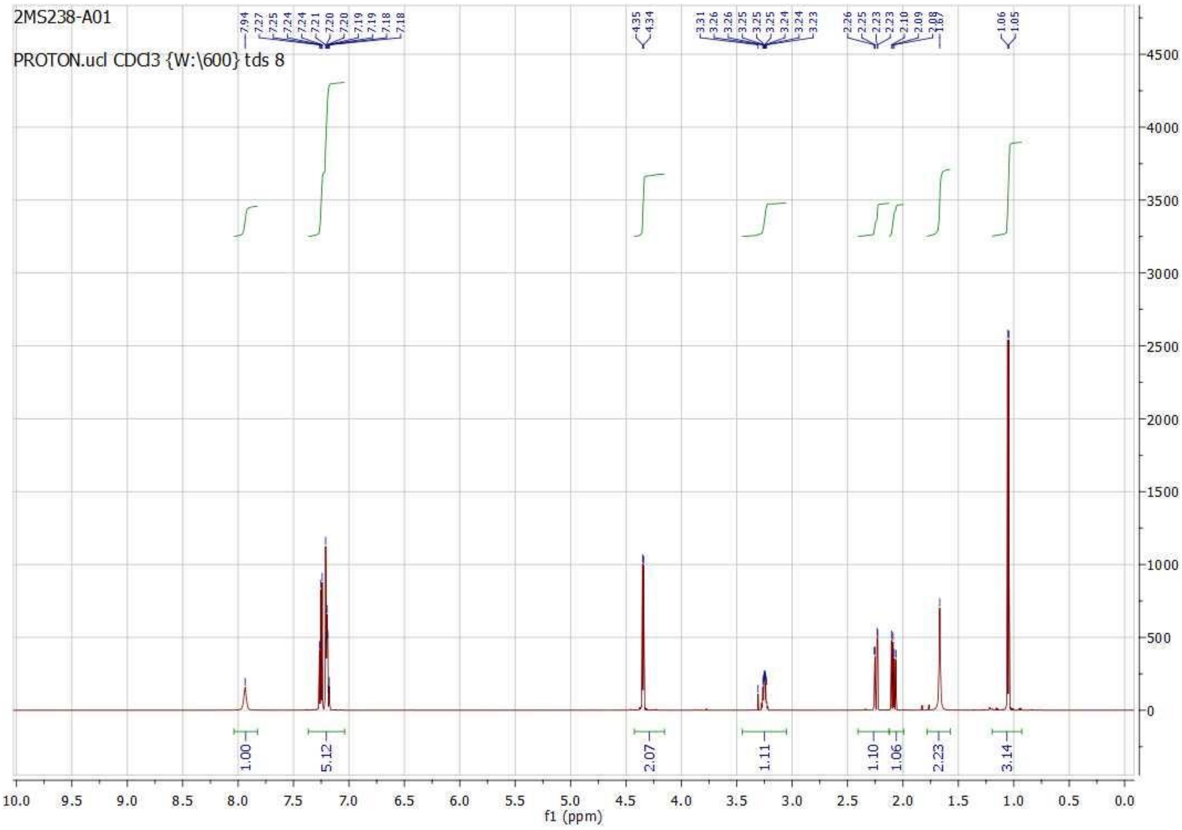

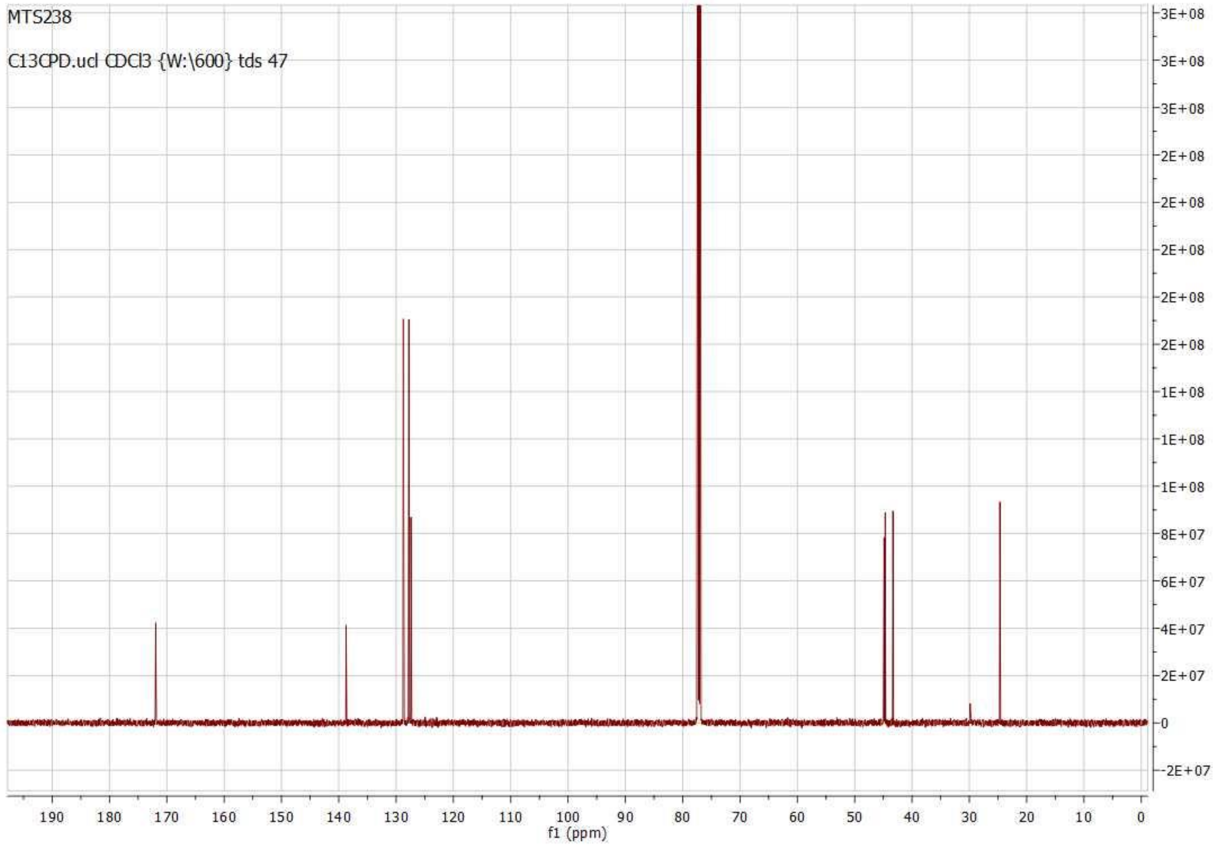

***(S)*-2-amino-N-benzylbutanamide (56)**

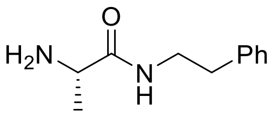

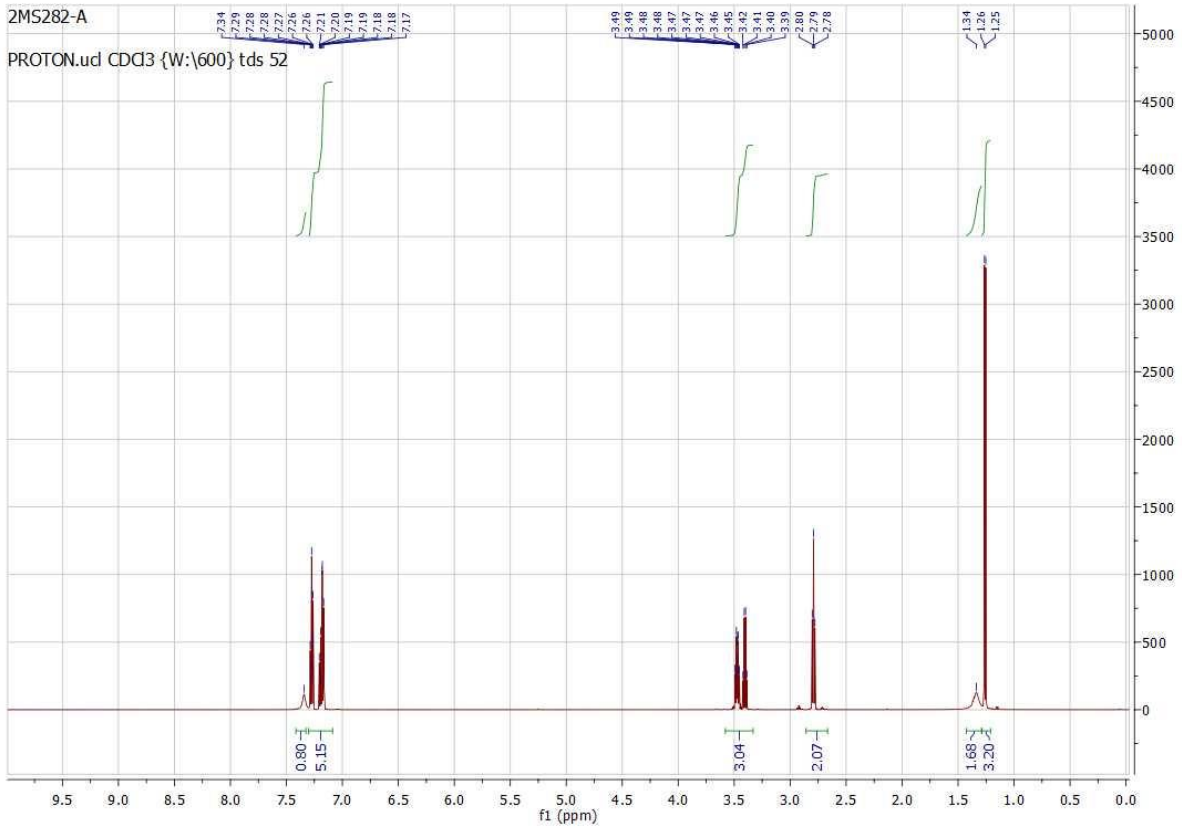

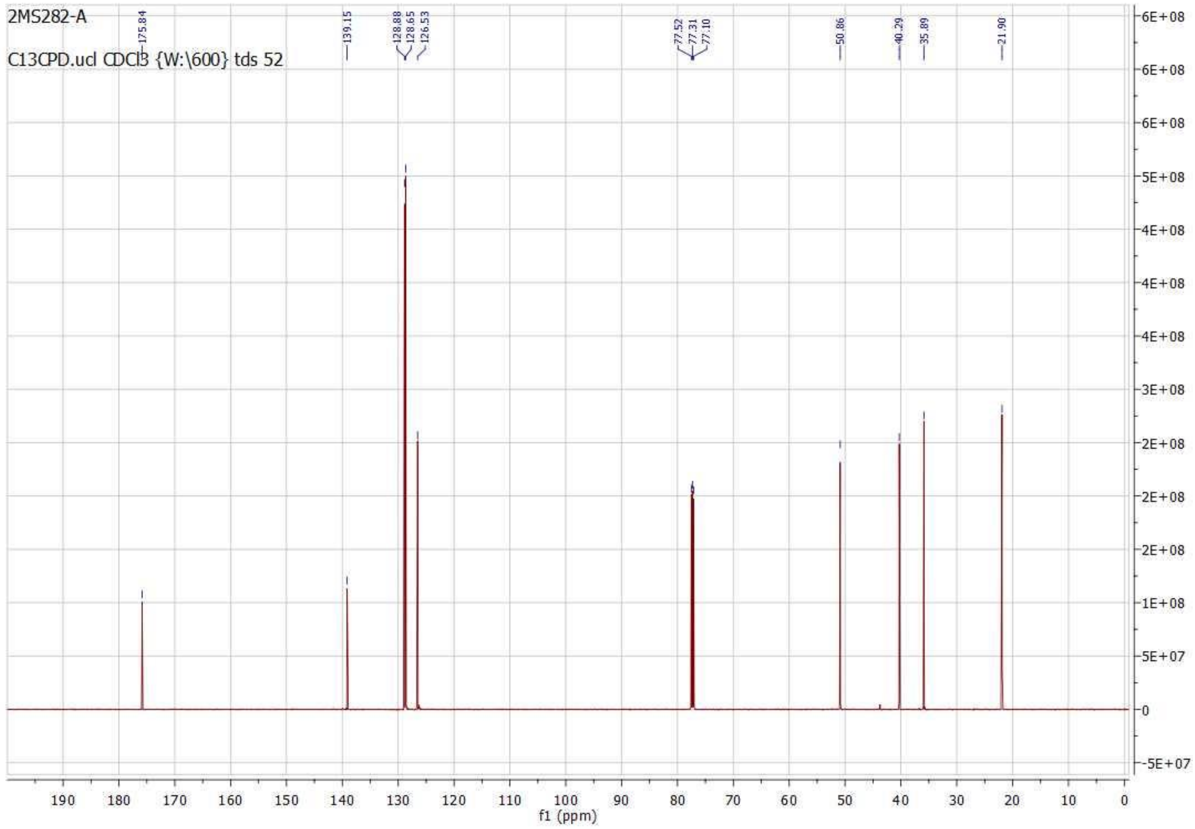

***N*-(cyclohexylmethyl)-2-(methylamino)acetamide (57)**

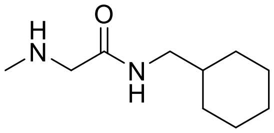

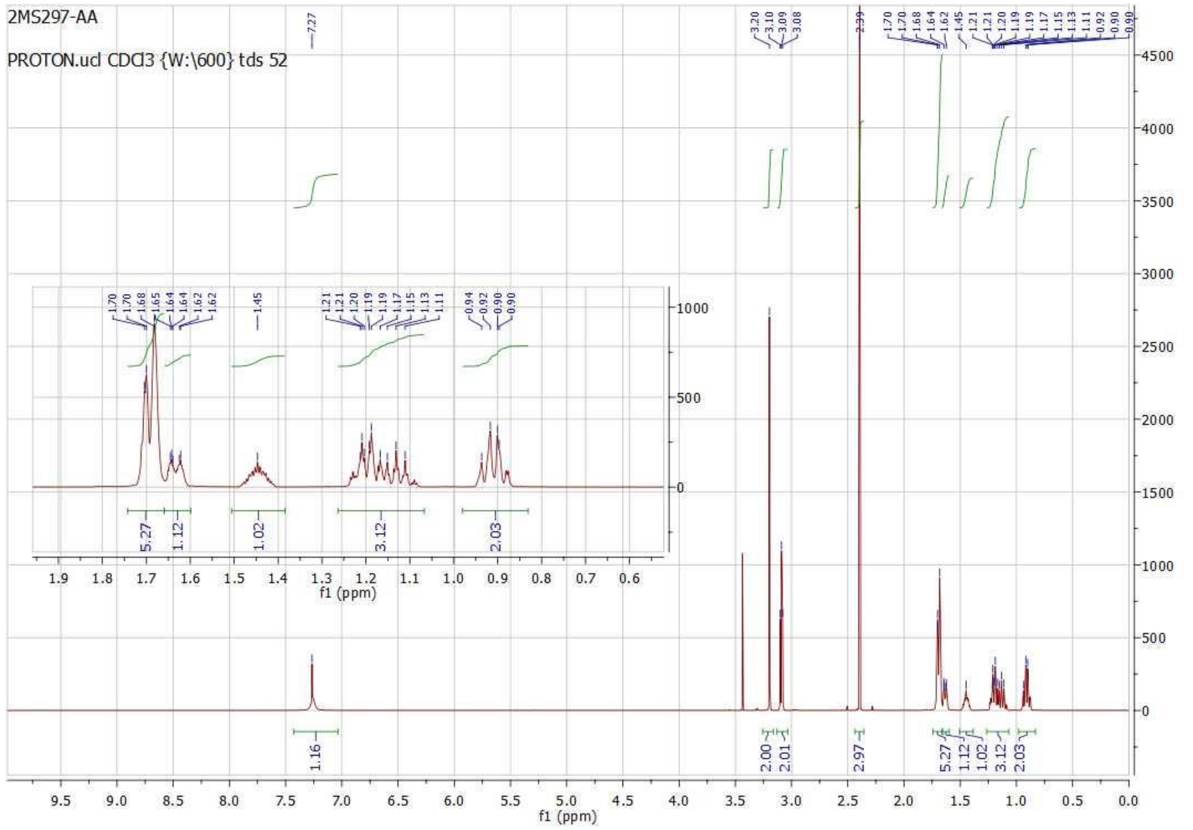

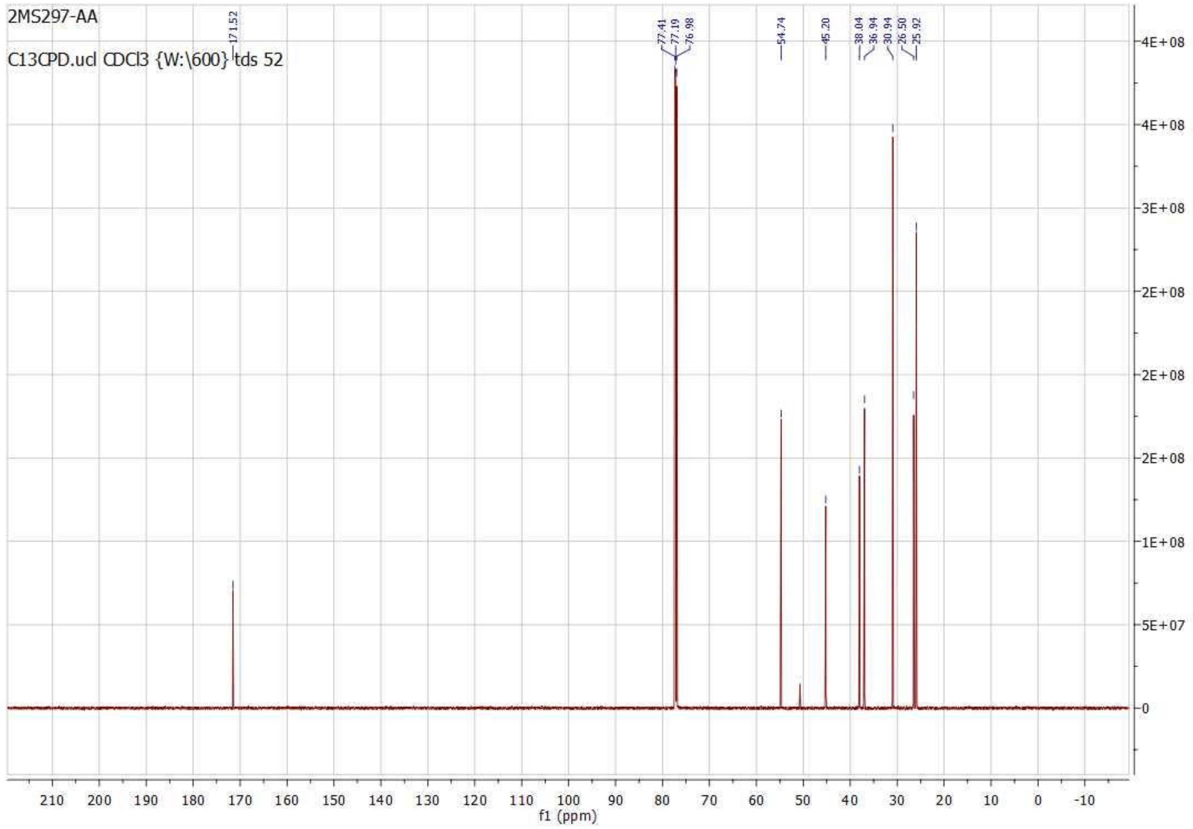

(S)-2-amino-N-hexyl-3-phenylpropanamide (58)

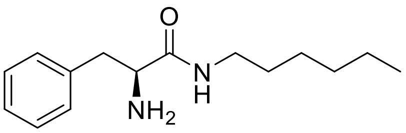

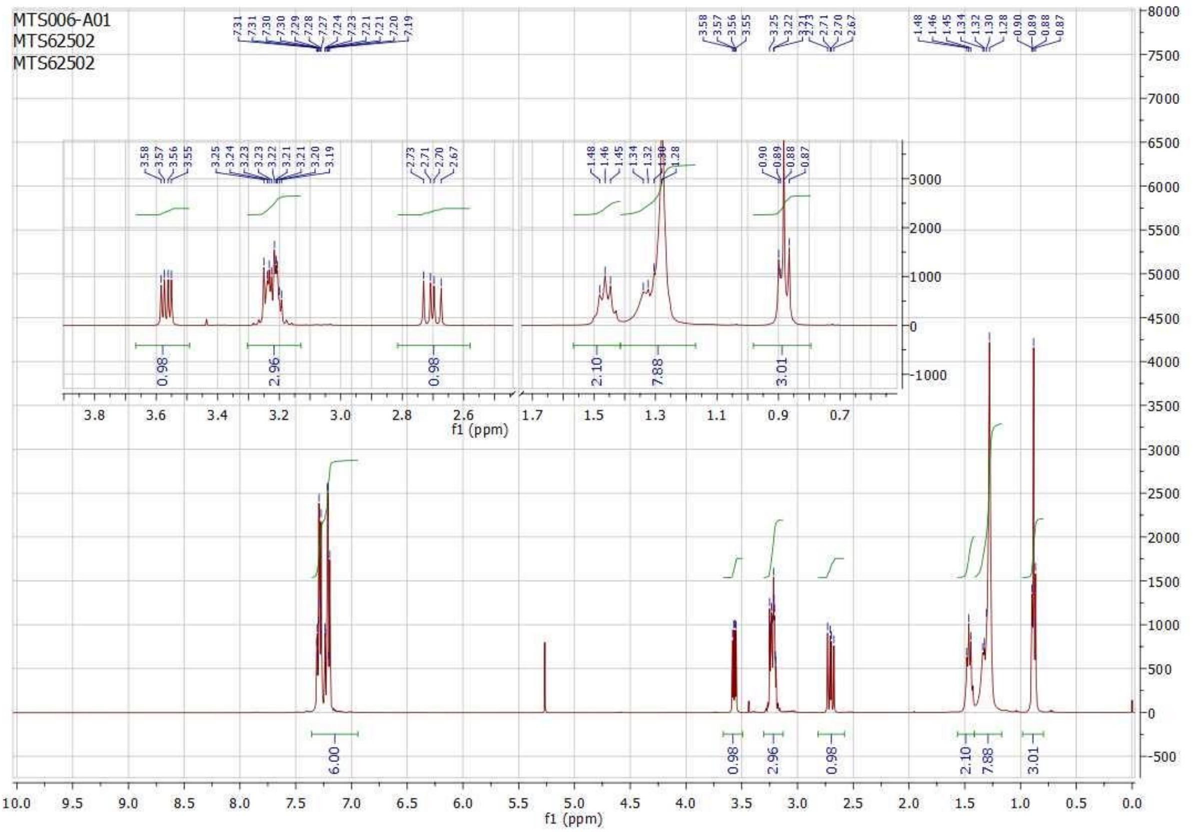

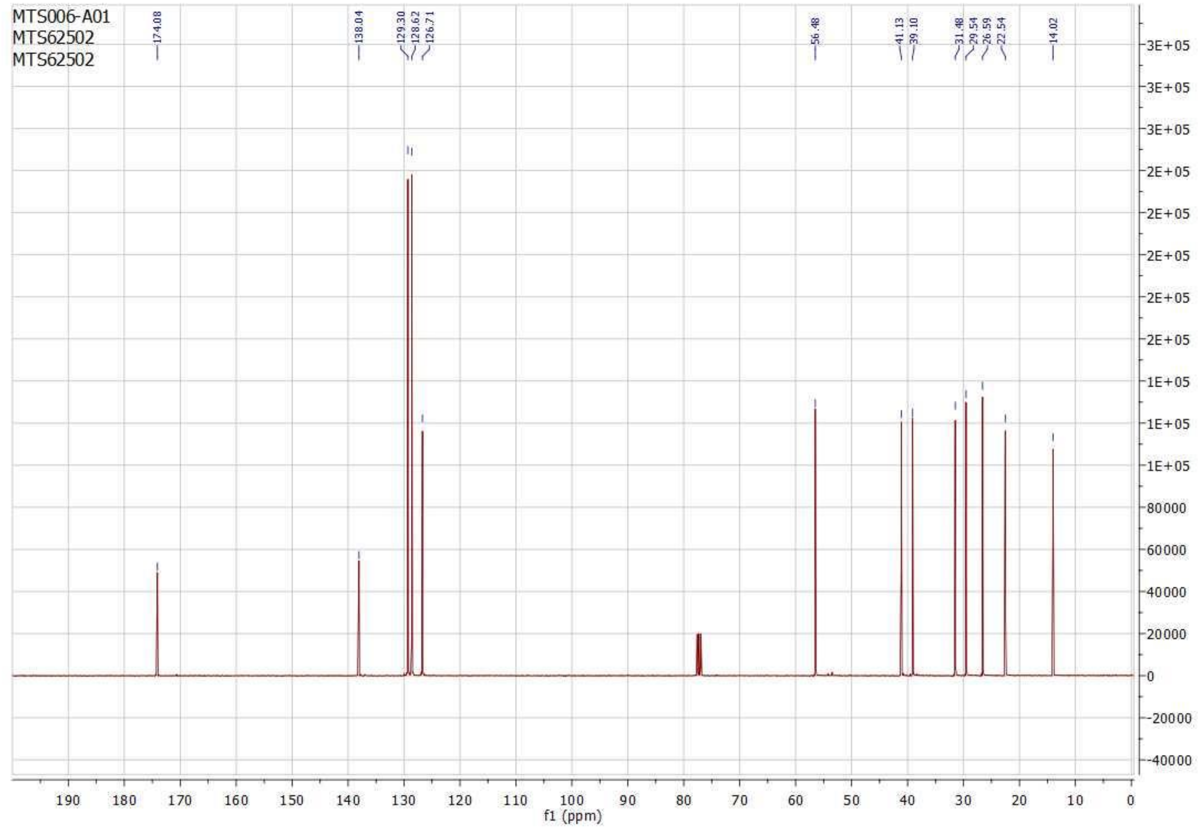

***tert*-Butyl (*S*)-prolylglycinate (59)**

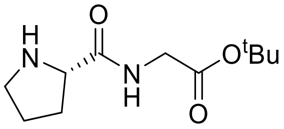

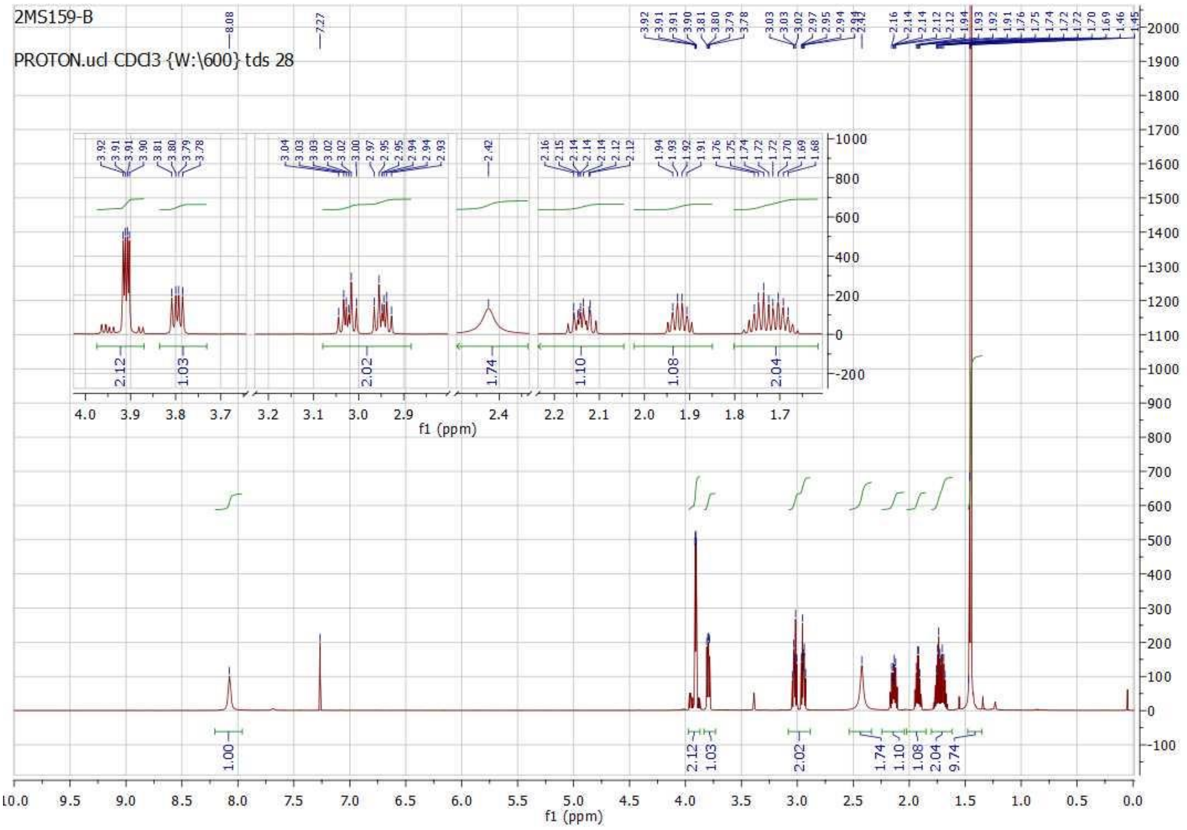

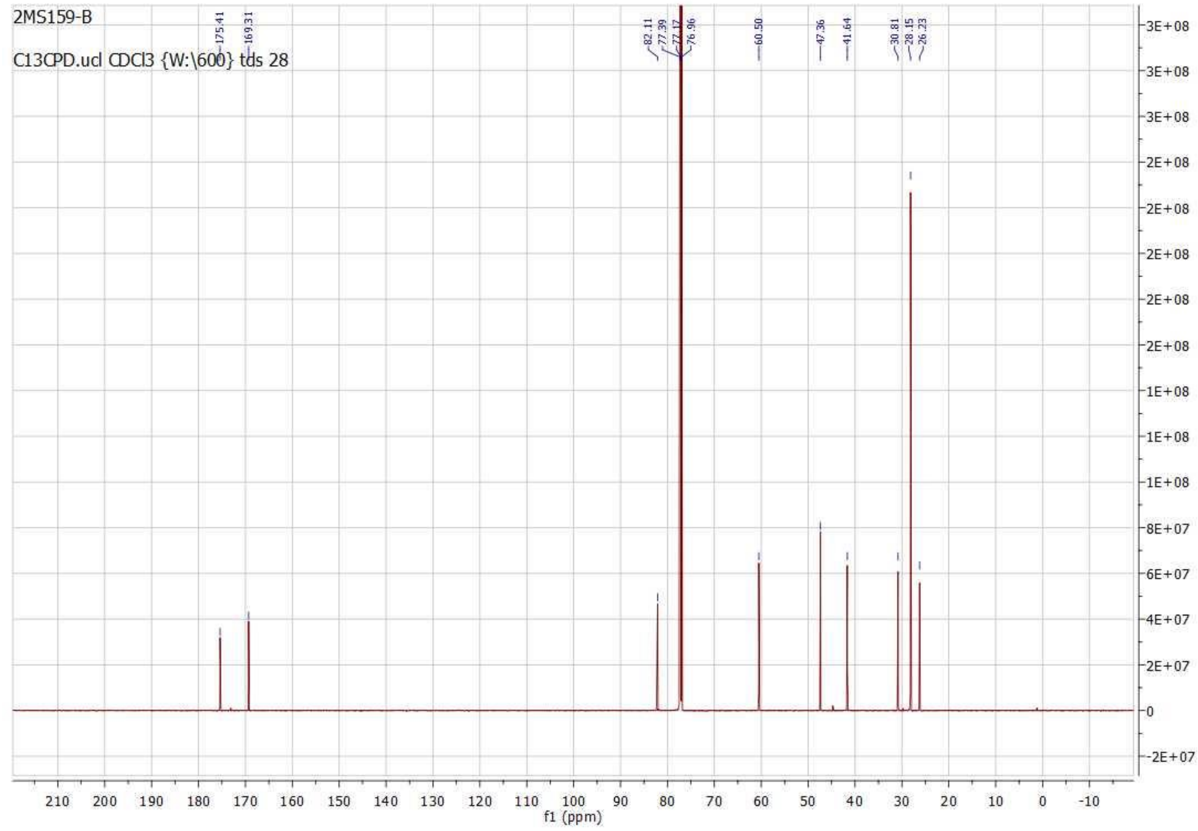

(R)-5-(4-Methylpiperazine-1-carbonyl)pyrrolidin-2-one (60)

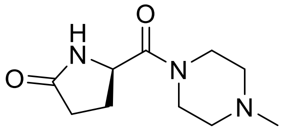

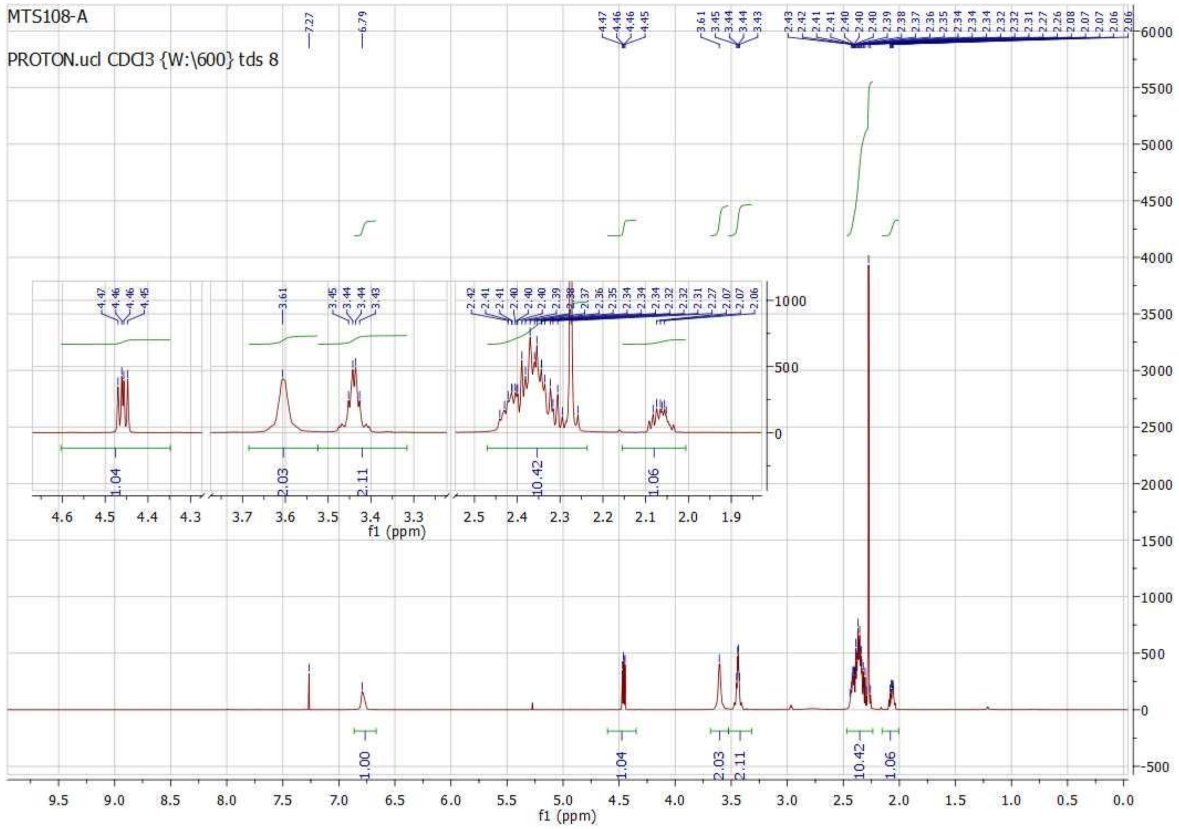

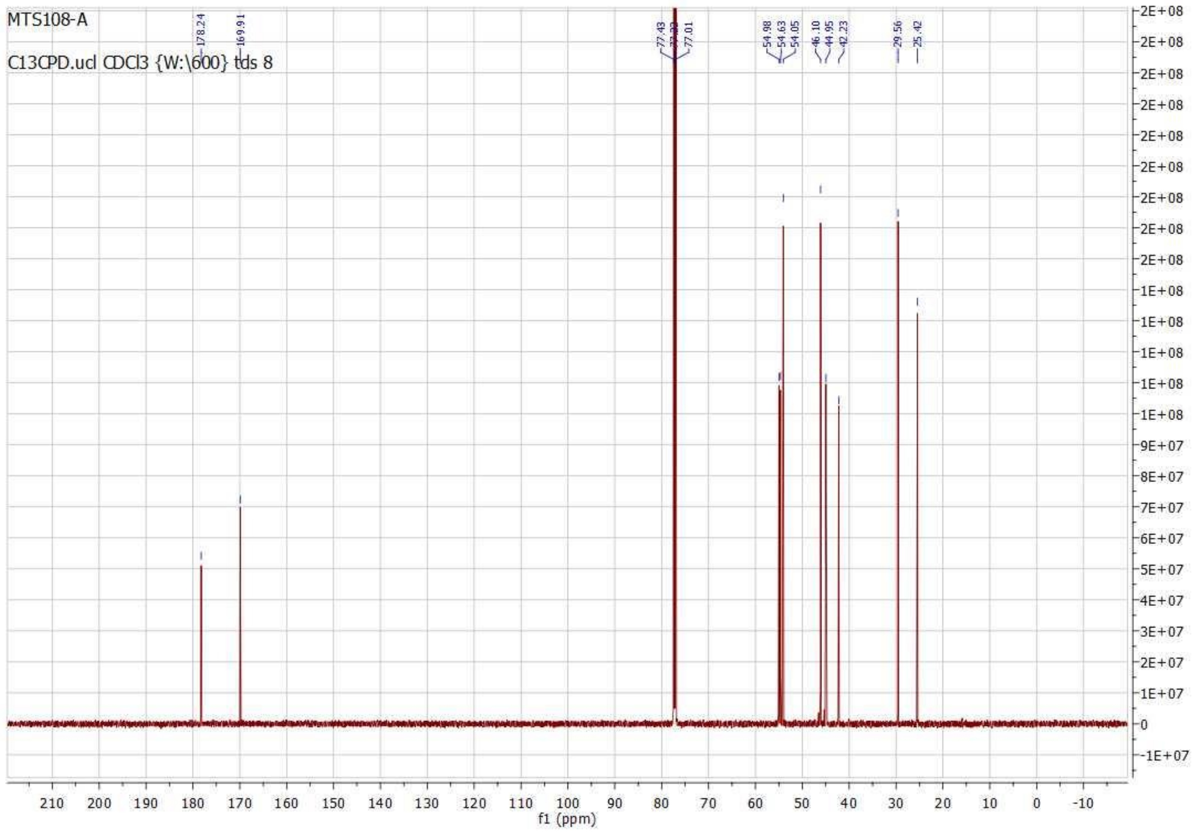

(*S*)-3,5-dibenzyl-2,2-dimethylimidazolidin-4-one (61)

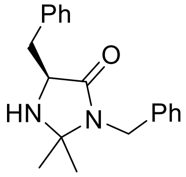

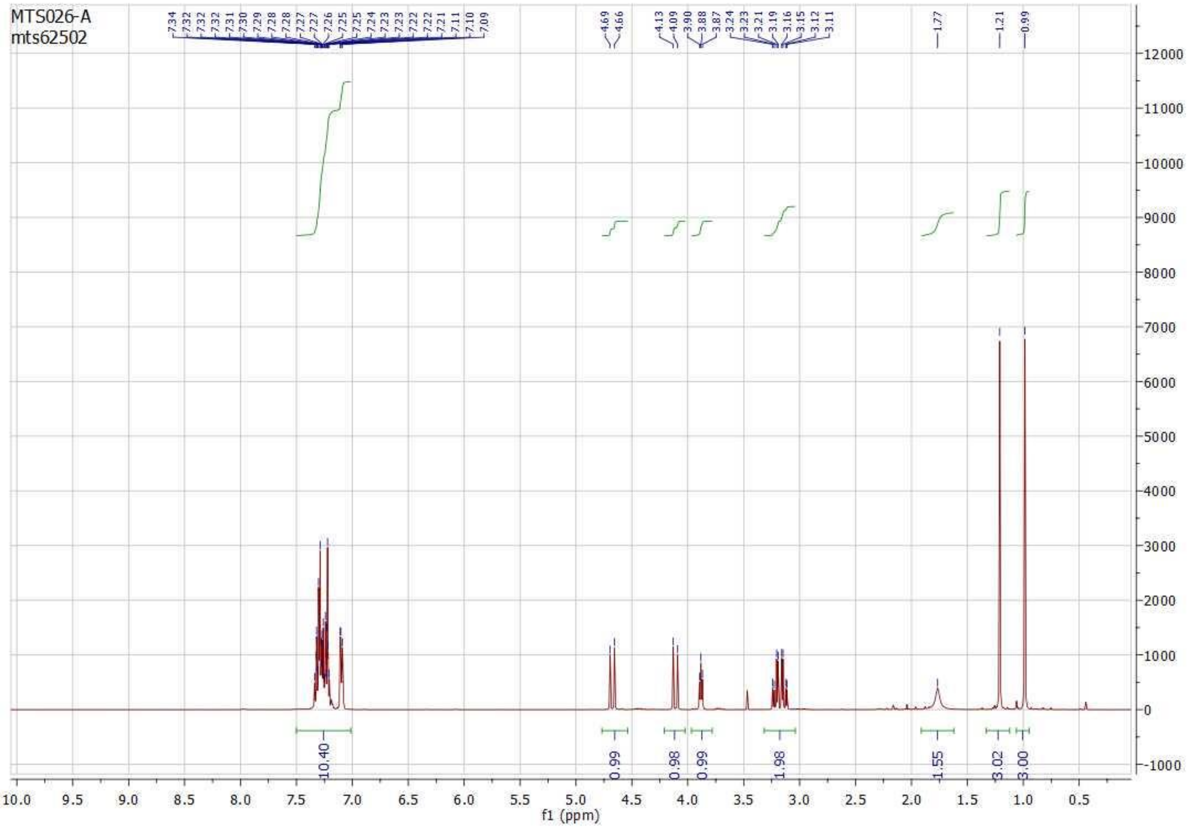

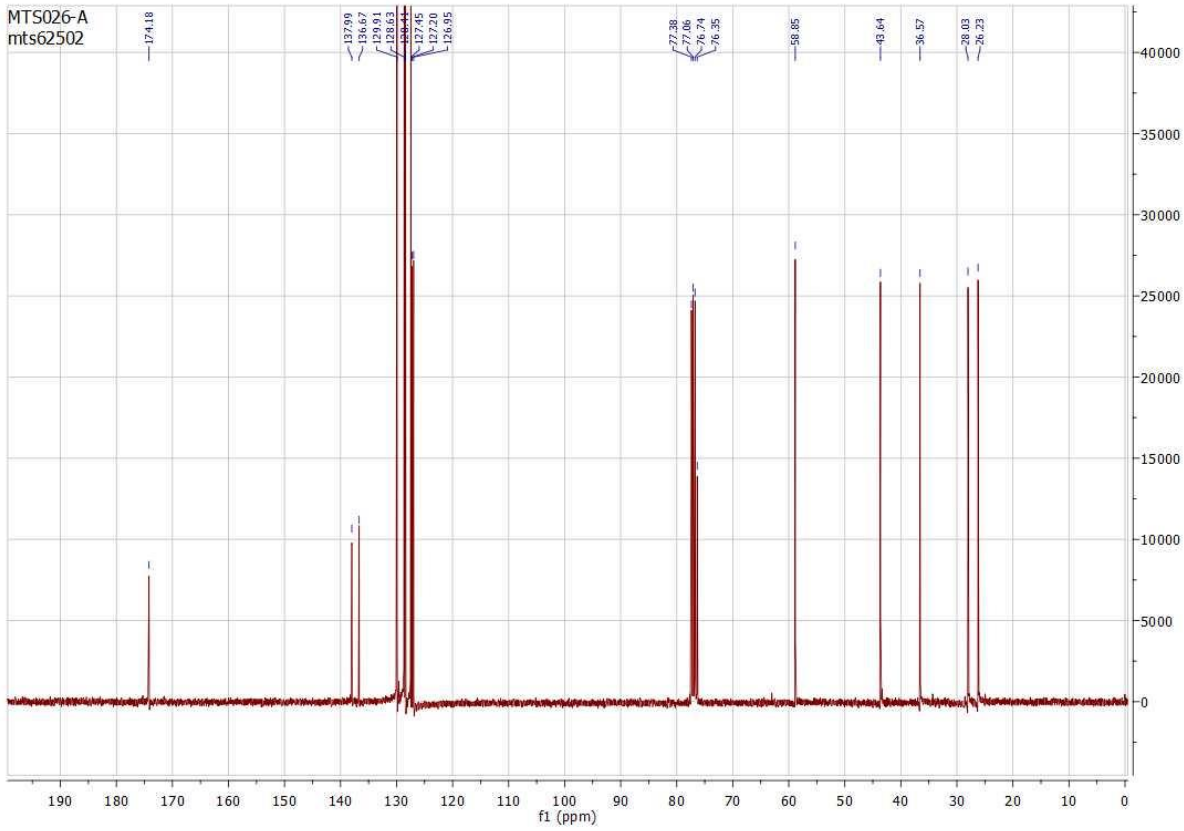

***(2R,5S)*-3,5-dibenzyl-2-(tert-butyl)imidazolidin-4-one (62 A)**

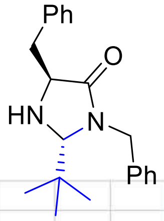

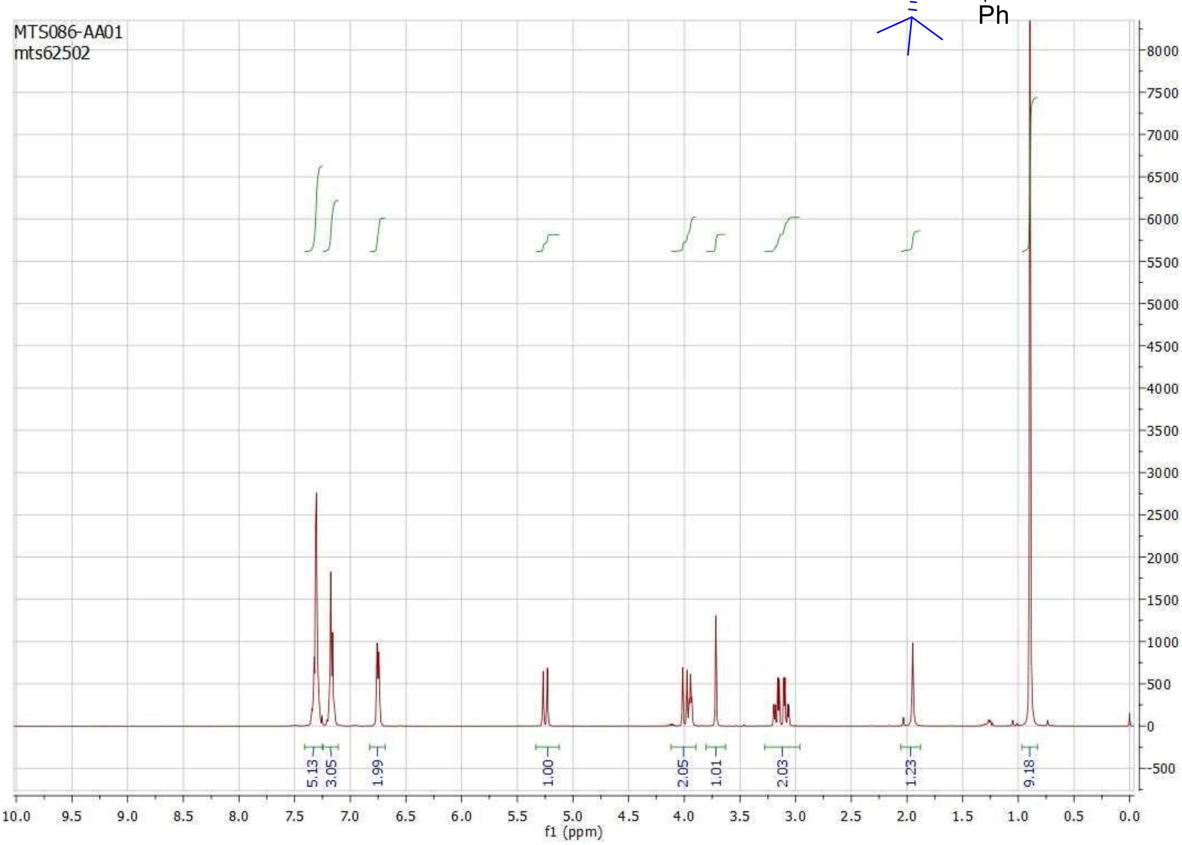

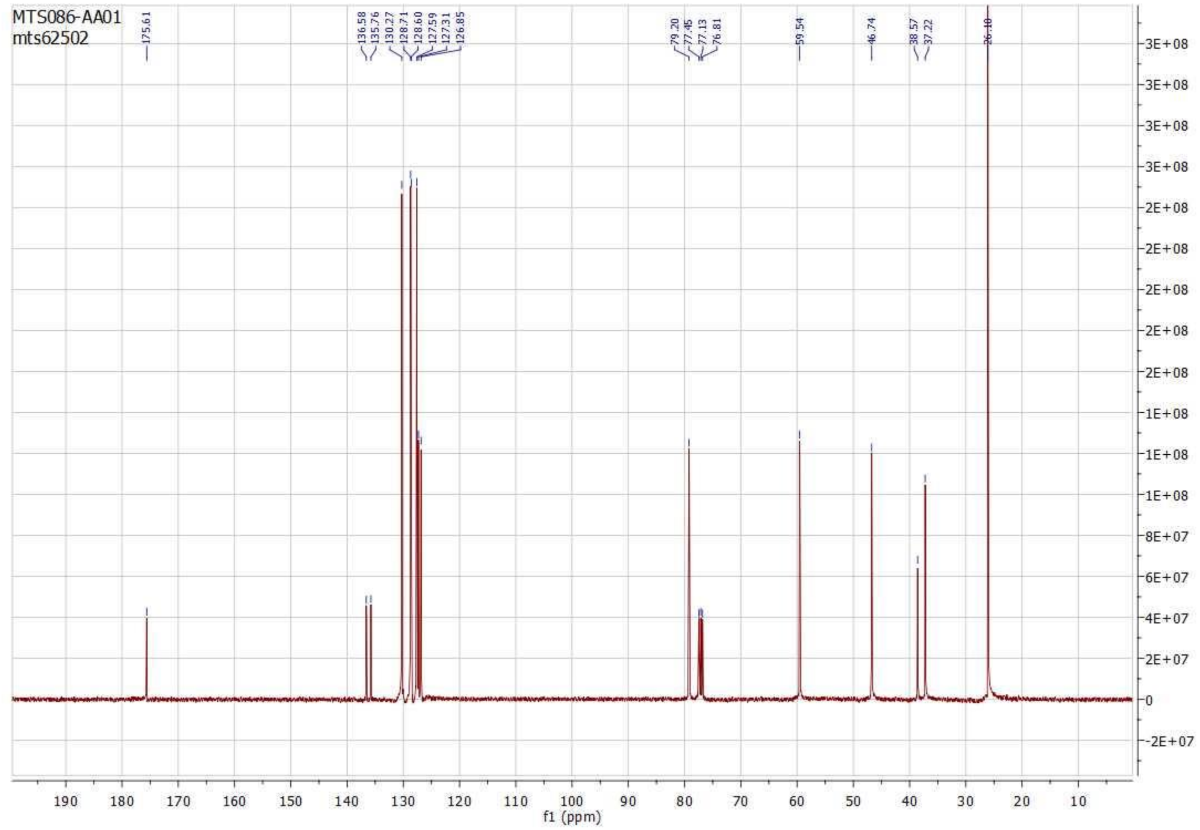

(2S,5S)-3,5-dibenzyl-2-(tert-butyl)imidazolidin-4-one (62 B)

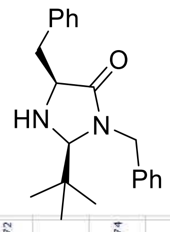

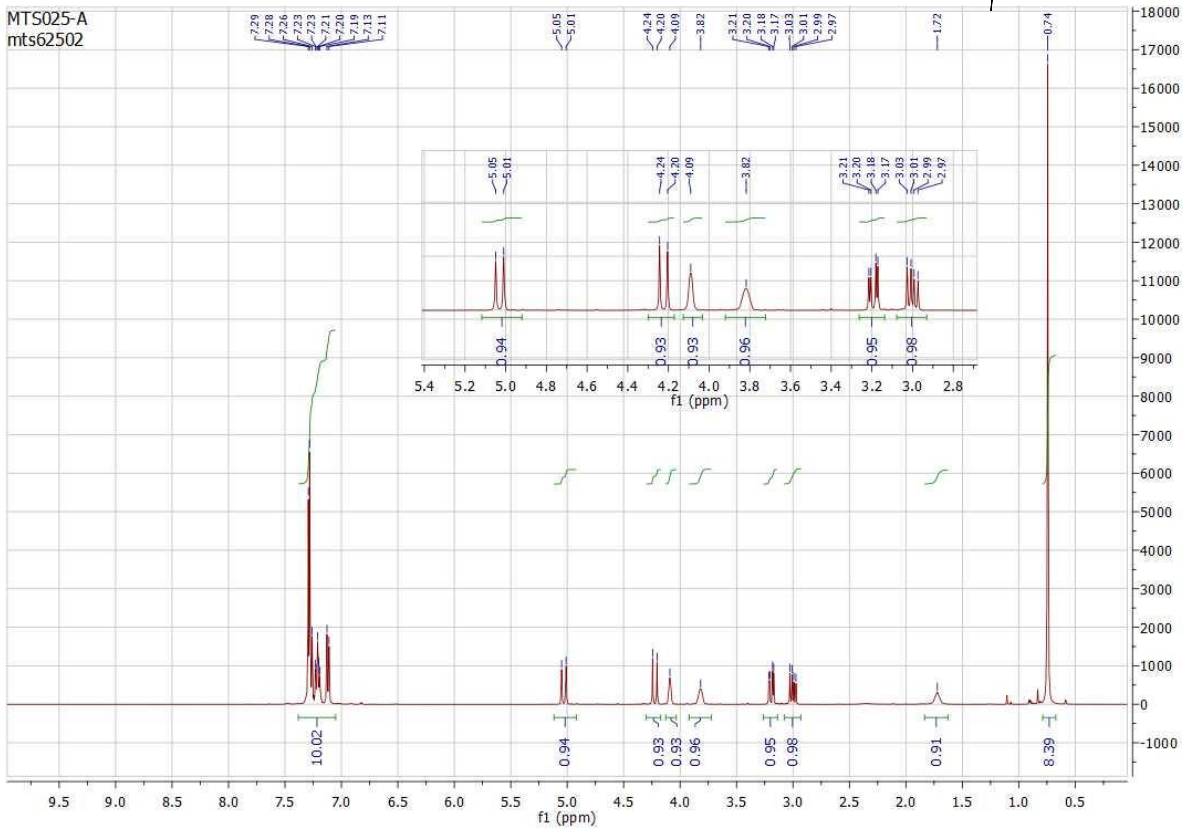

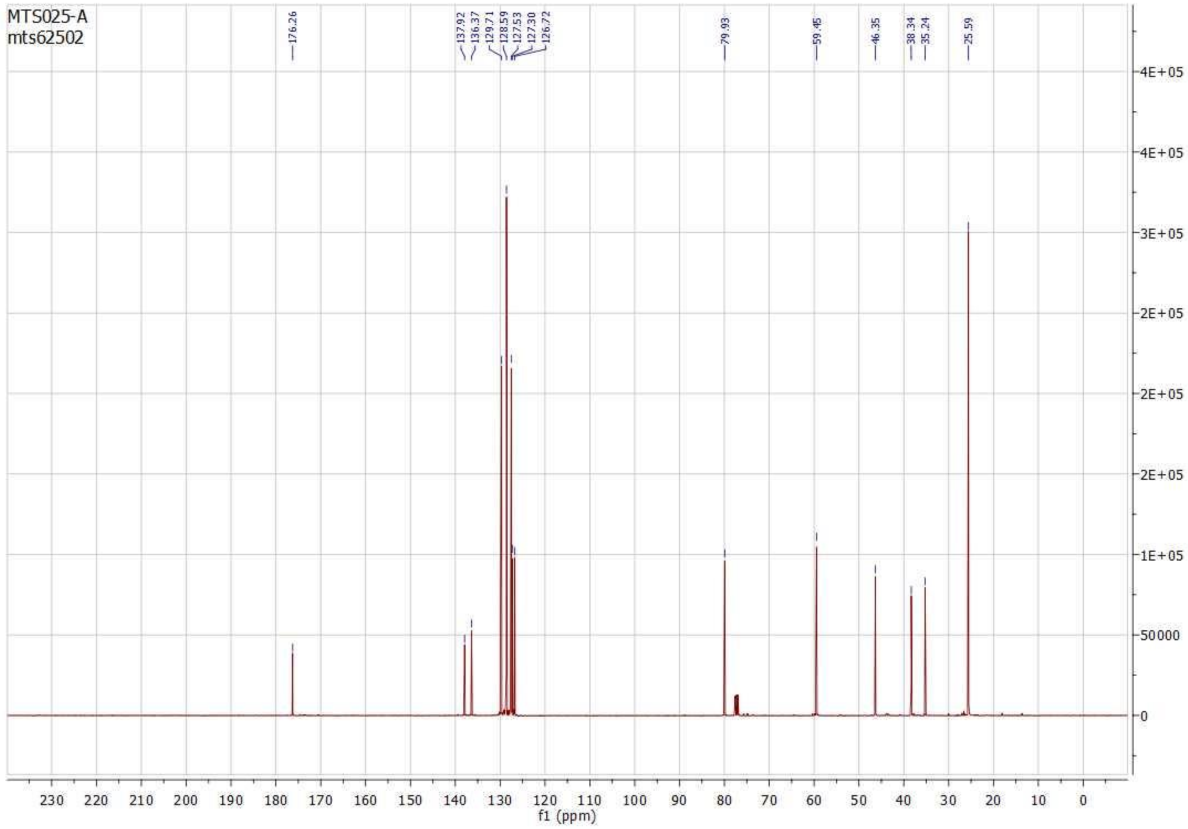

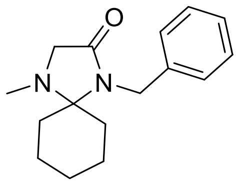

1-benzyl-4-methyl-1,4-diazaspiro[4.5]decan-2-one, (63)

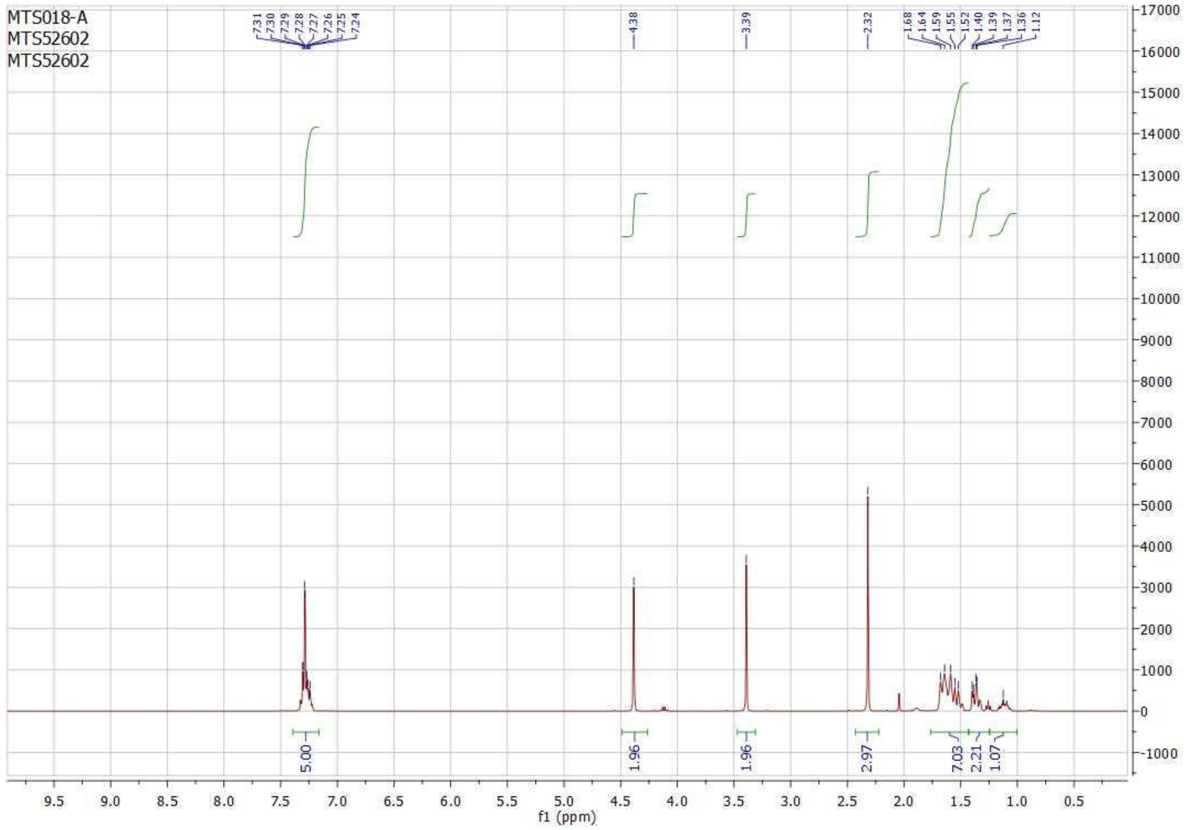

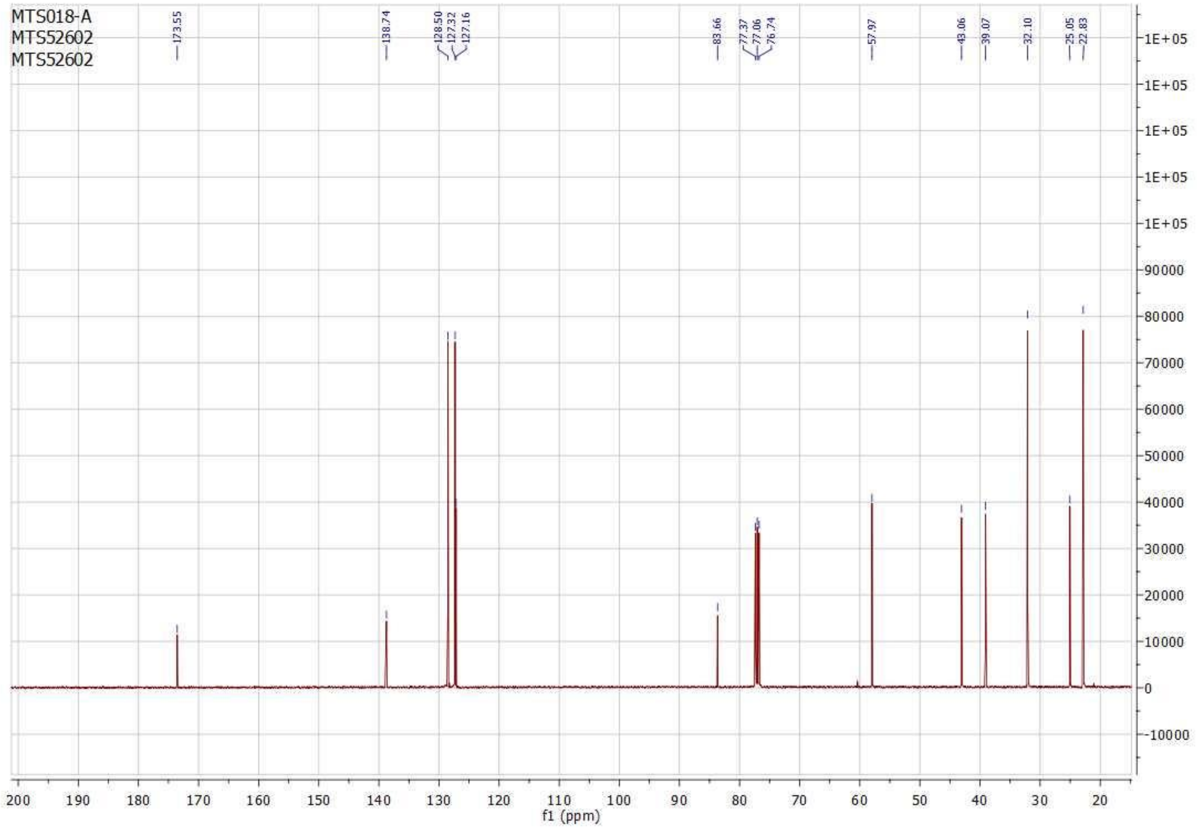

(5S)-2-ethyl-2,5-dimethyl-3-phenethylimidazolidin-4-one (64)

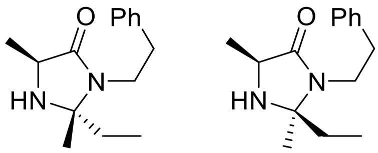

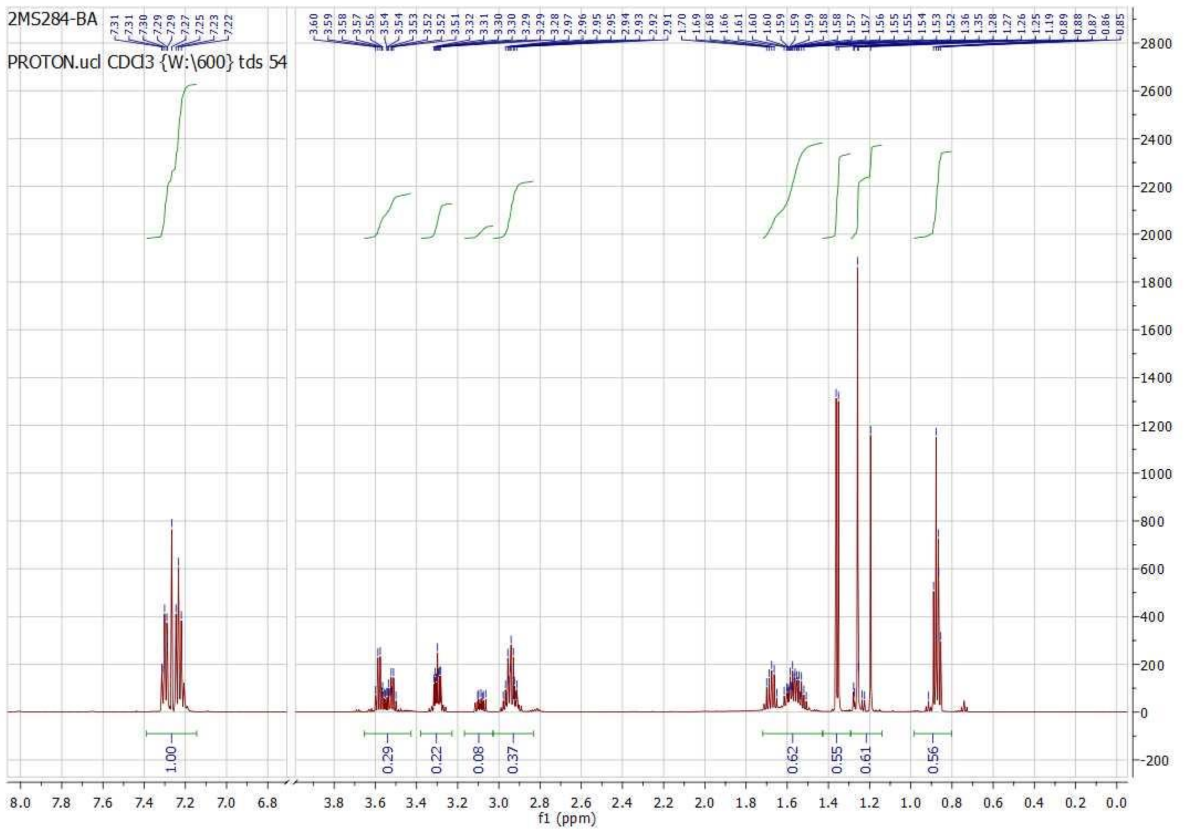

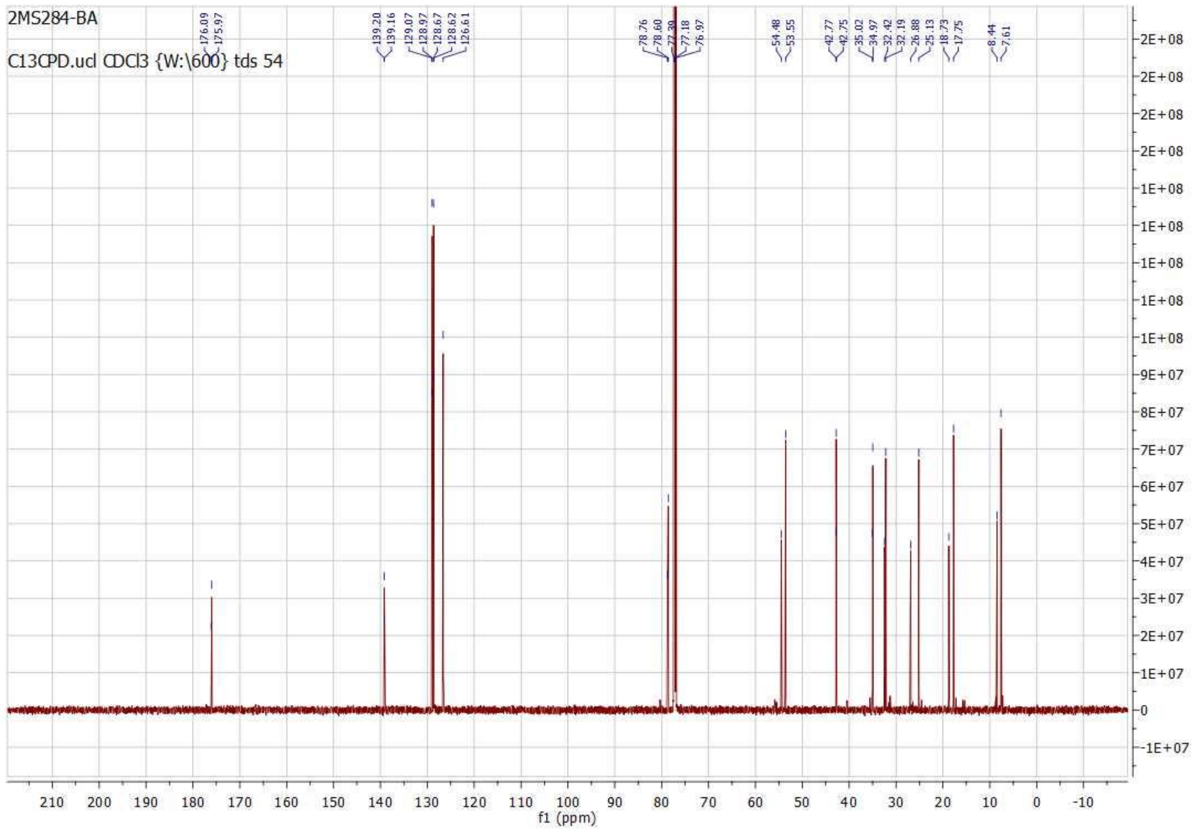

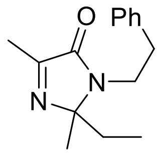

2-ethyl-2,5-dimethyl-3-phenethyl-2,3-dihydro-4H-imidazol-4-one (65)

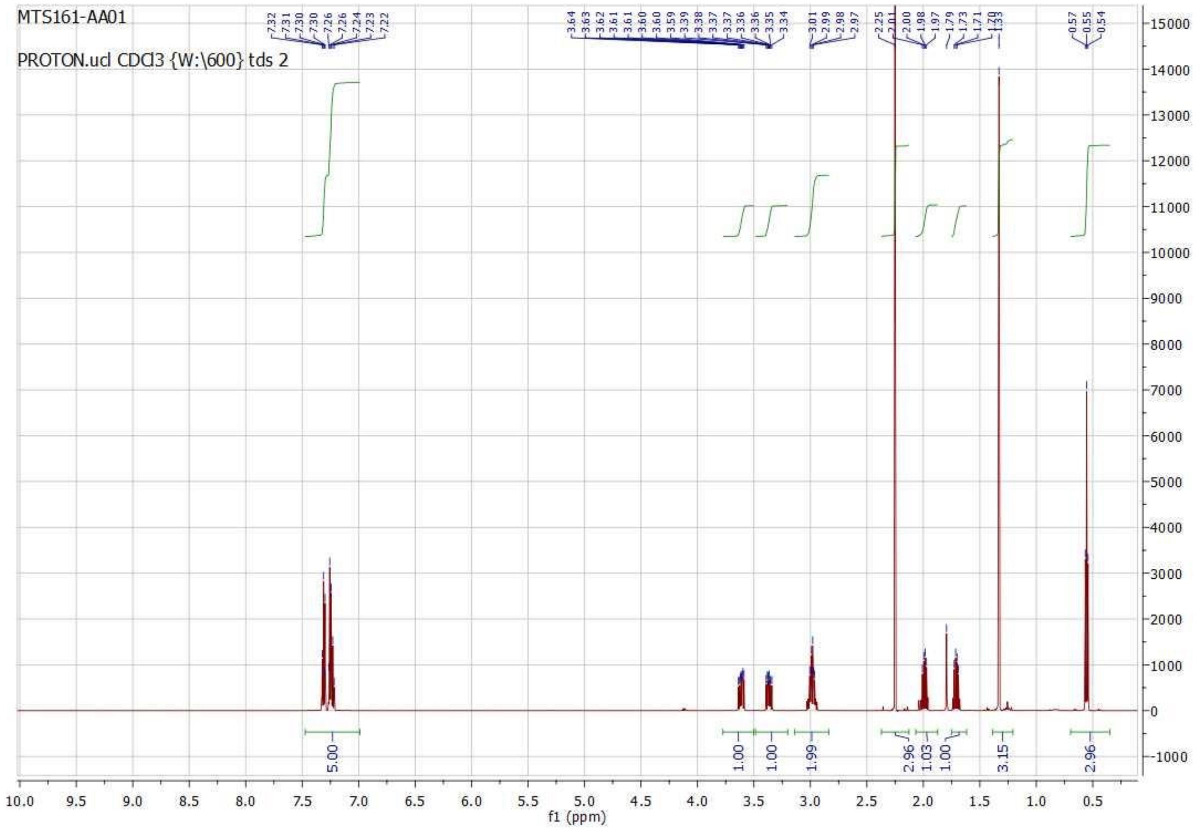

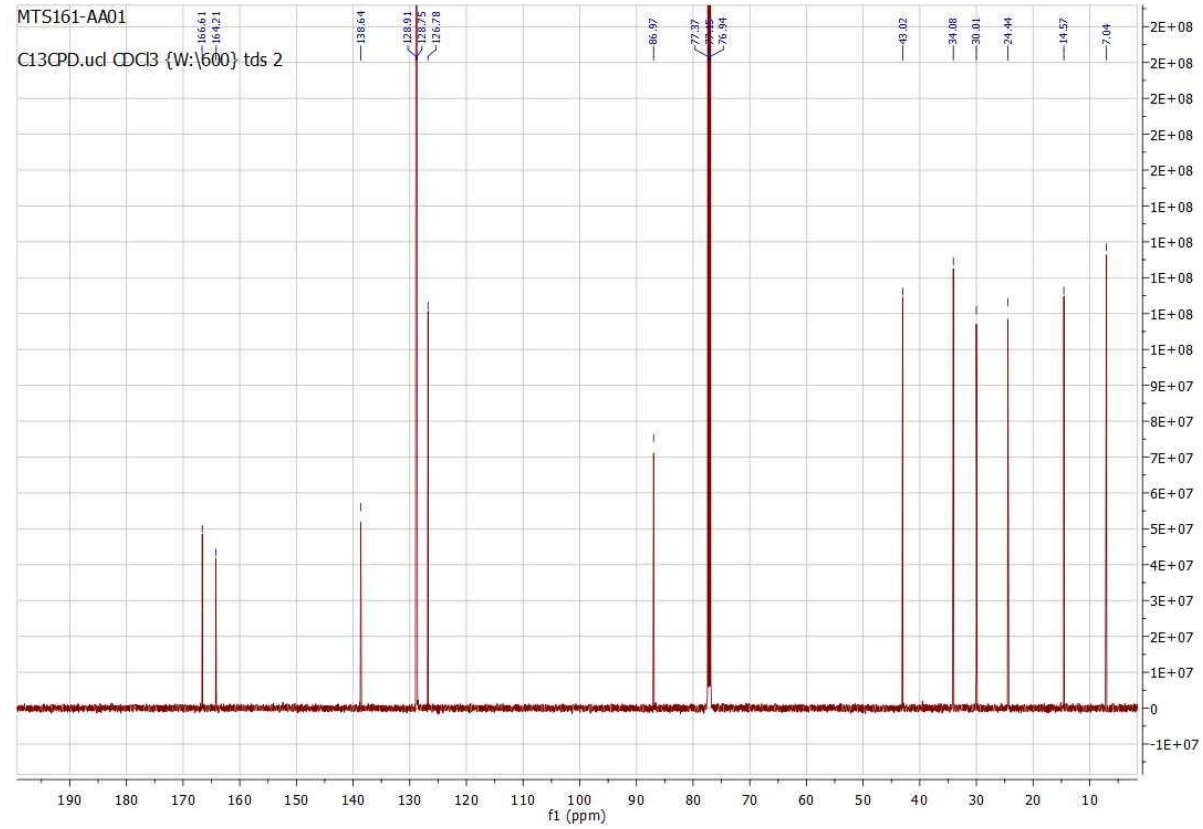

***tert*-butyl pentanoyl-L-valinate (66)**

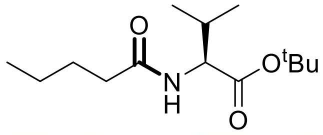

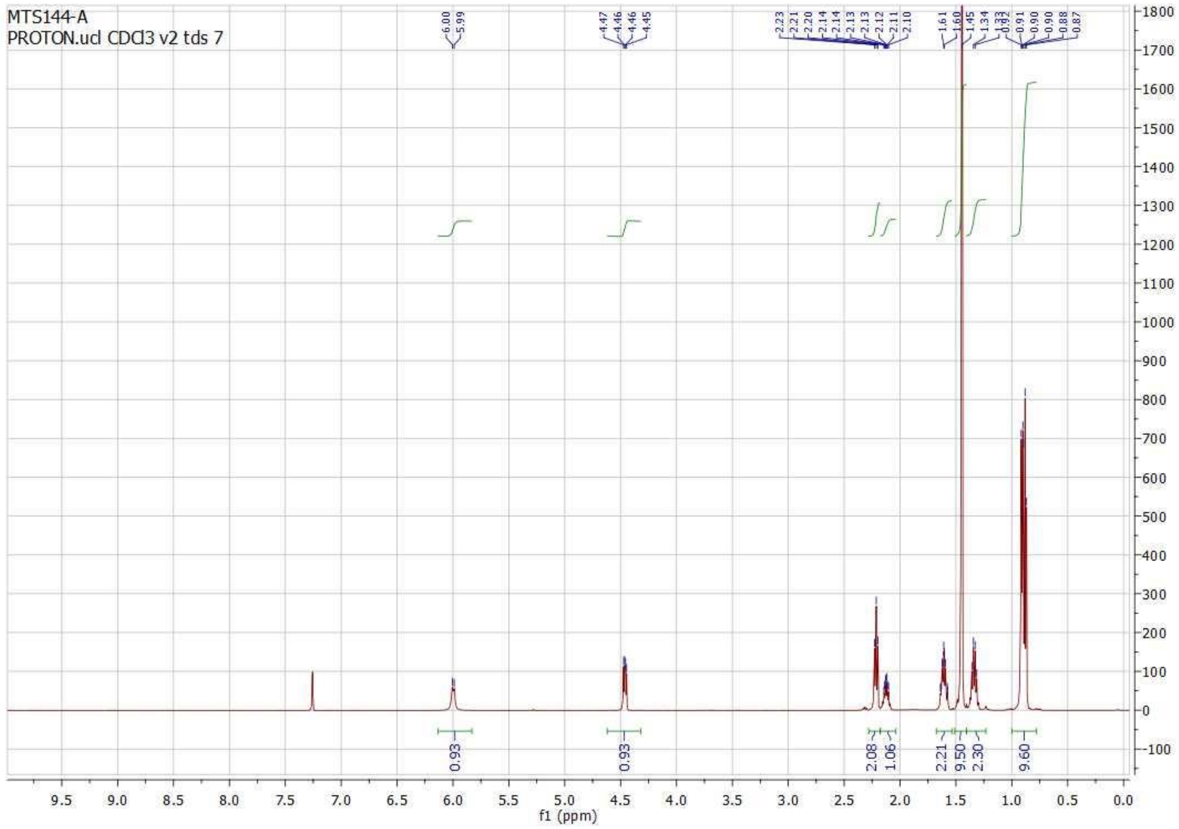

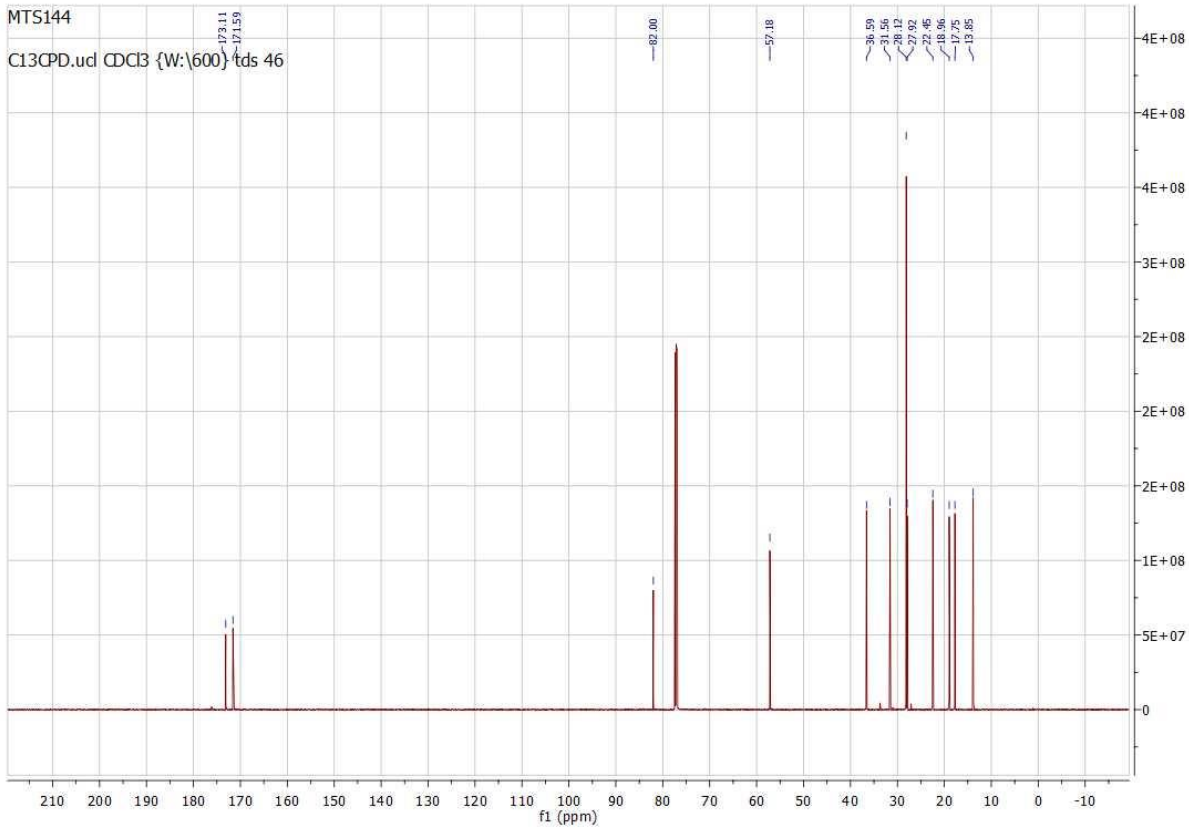

1-(1,4-diazepan-1-yl)butan-1-one (67)

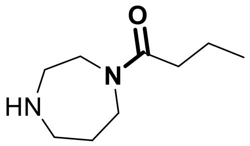

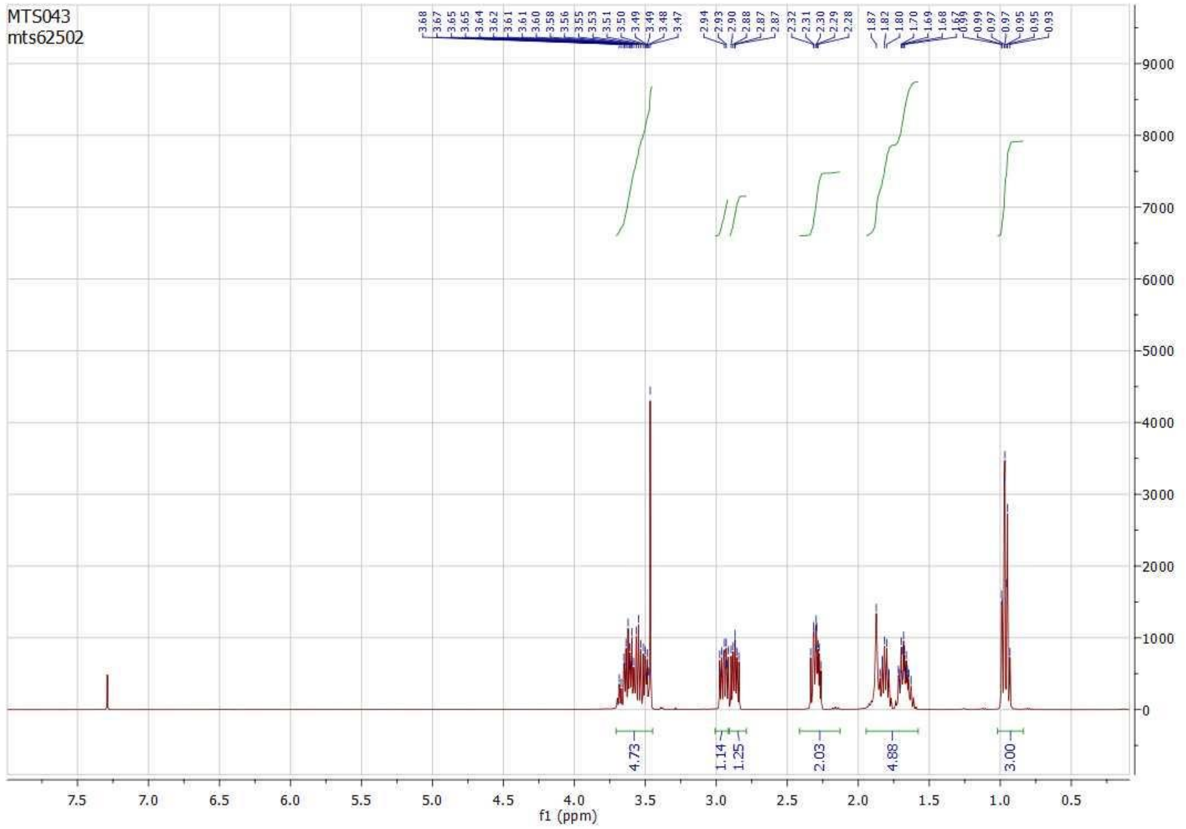

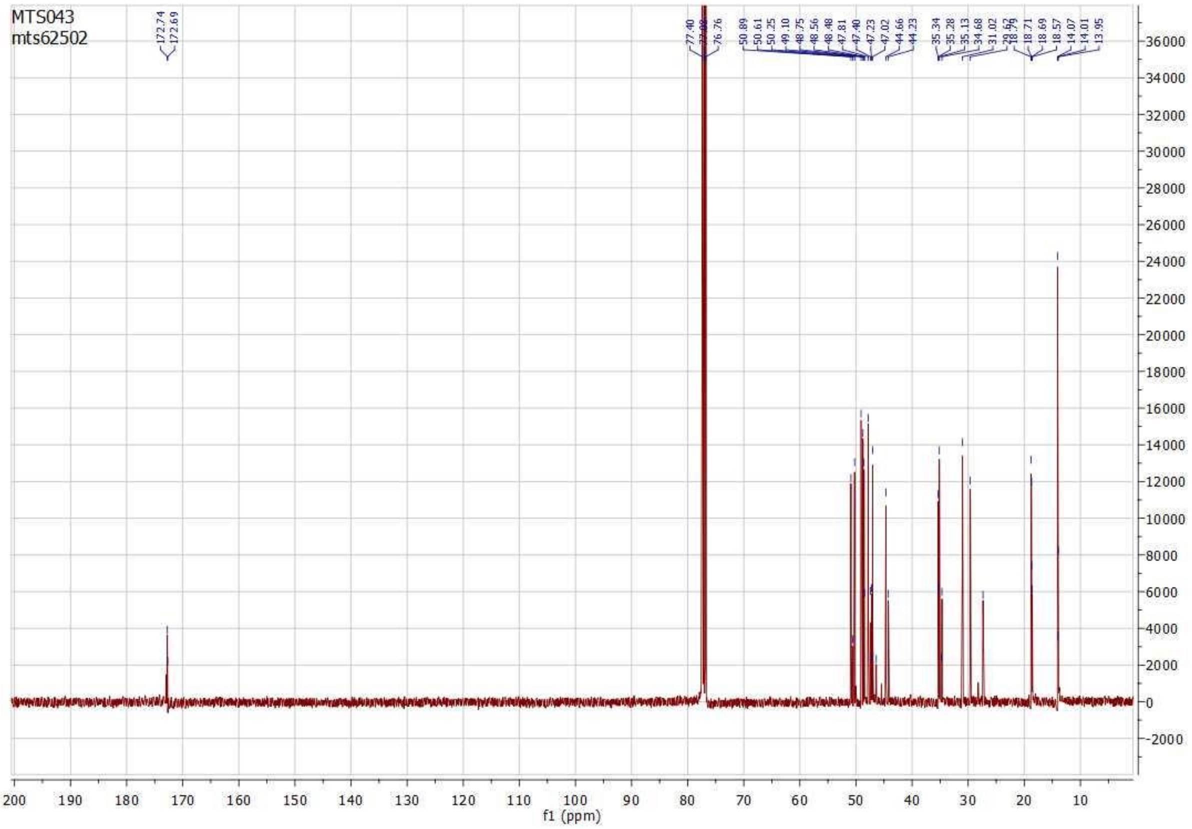

tert-butyl (2-phenylacetyl)-L-prolylglycinate (68)

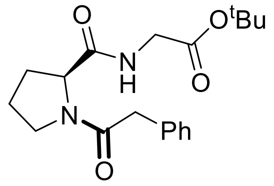

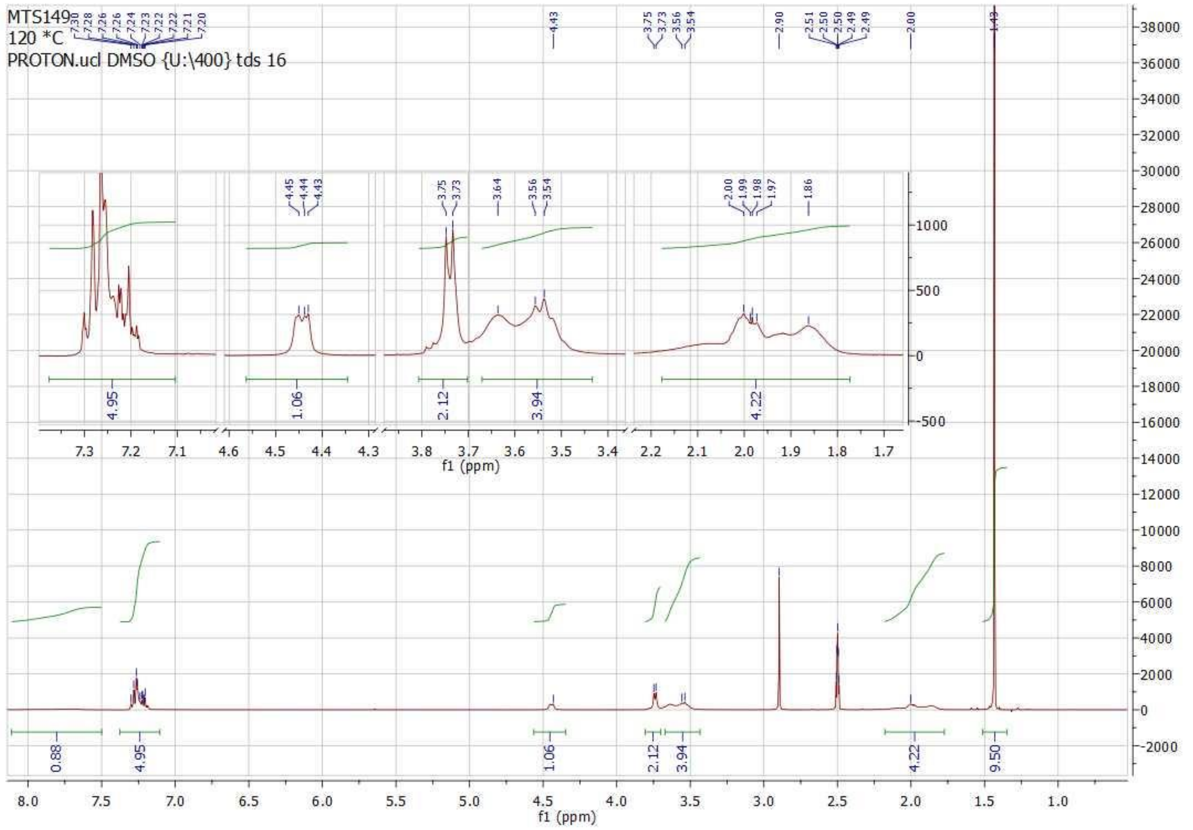

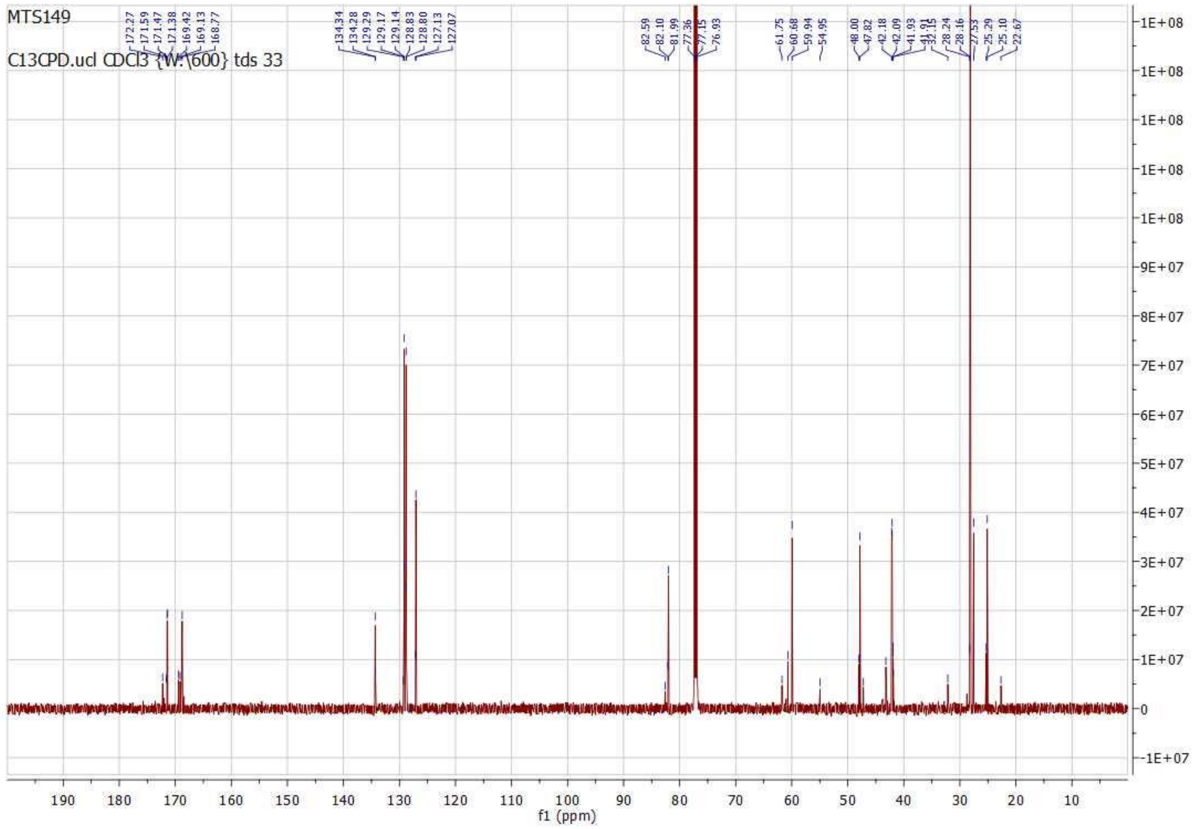

_N_,3-diphenylpropiolamide (69)

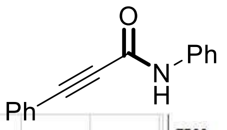

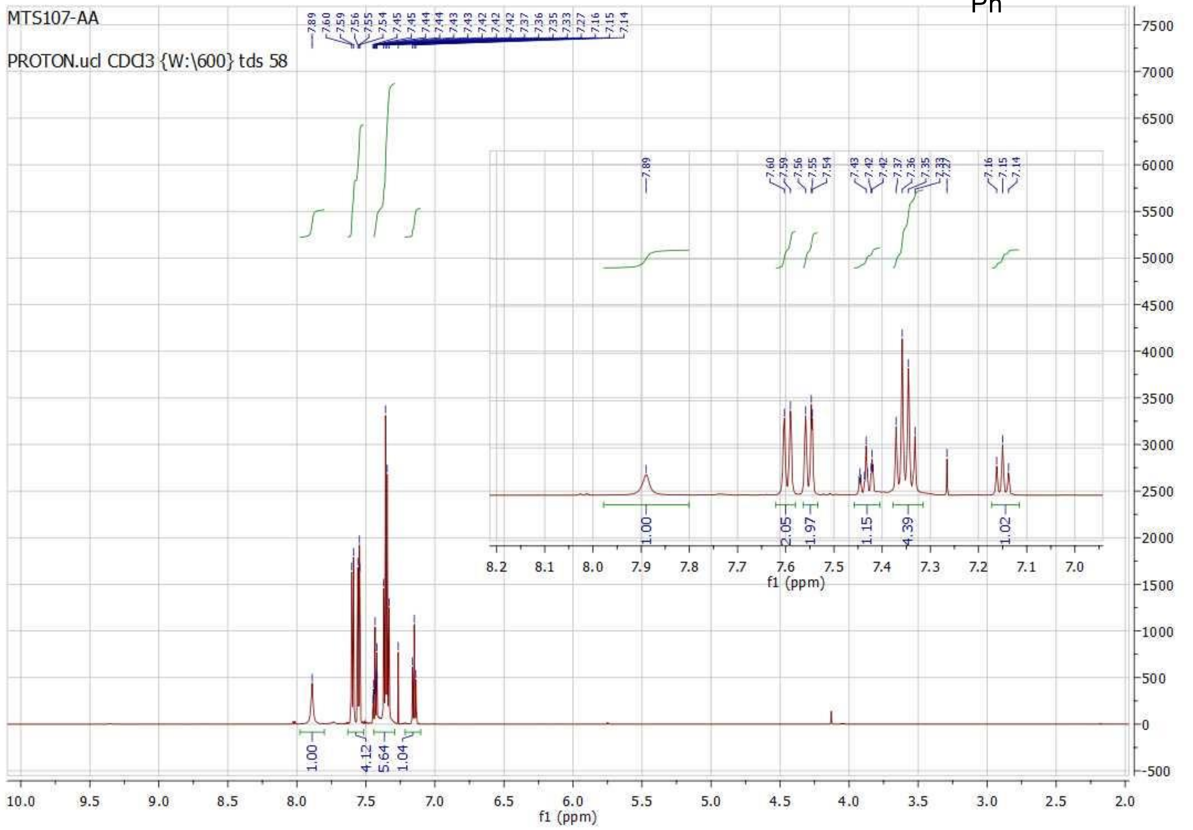

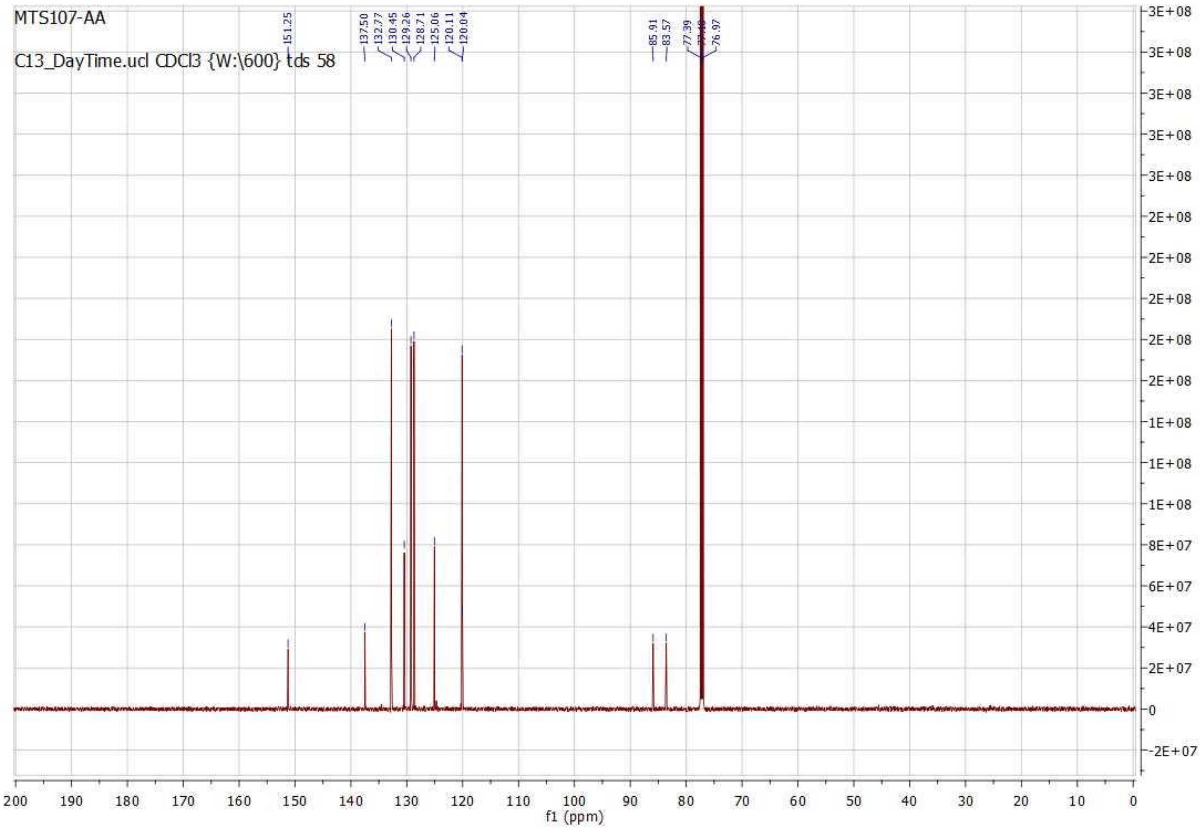

1-methyl-N-9-methyl-9-azabicyclo[3.3.1]nonan-3-yl)-1H-indazole-3-carboxamide (Granisetron, 70)

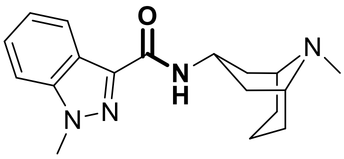

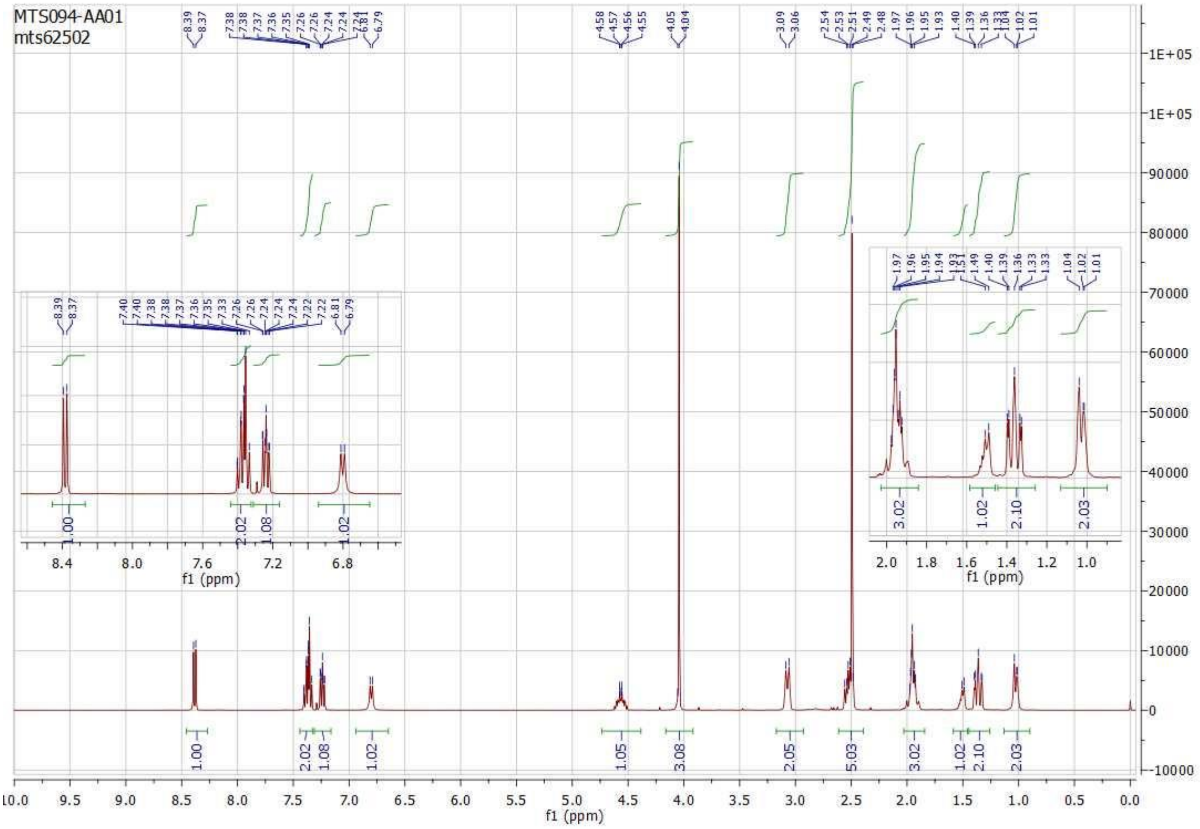

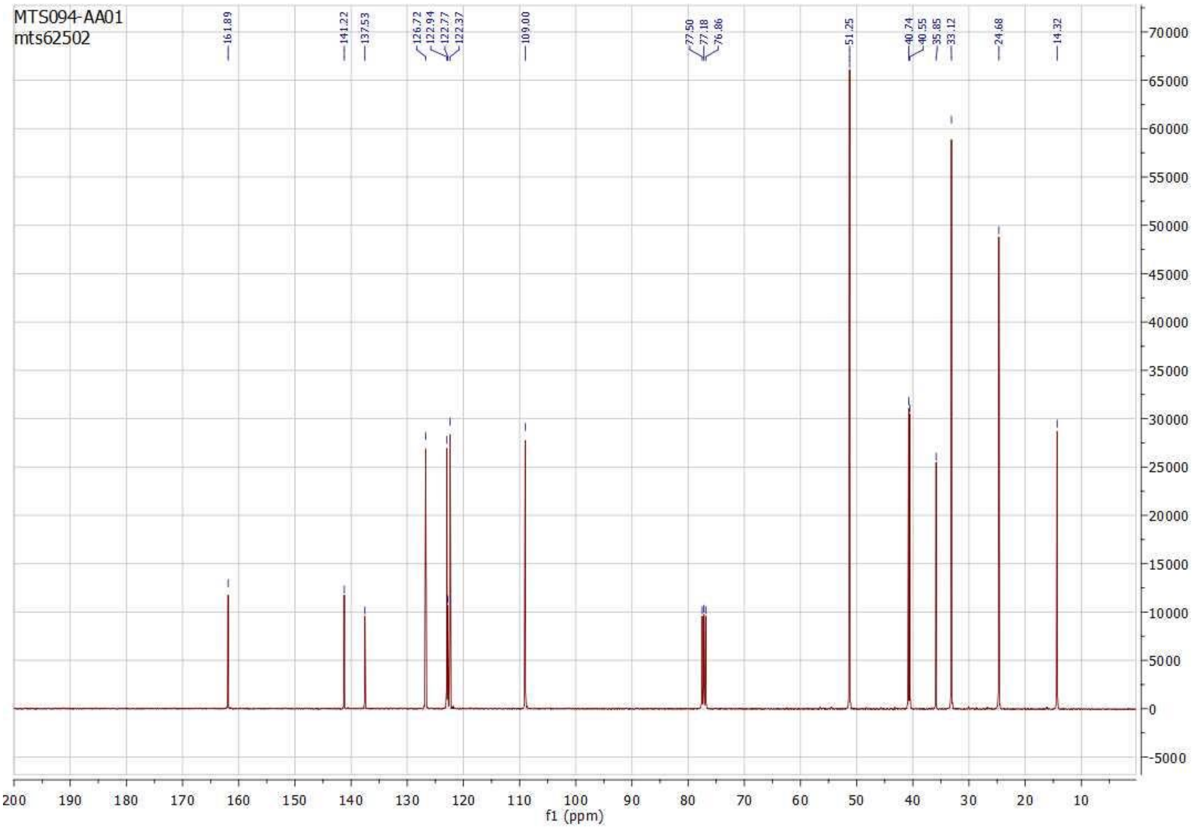

tert-butyl (R)-(4-oxo-4-(3-(trifluoromethyl)-5,6-dihydro-[1,2,4]triazolo[4,3-a]pyrazin-7(8H)-yl)-1-(2,4,5-trifluorophenyl)butan-2-yl)carbamate (71)

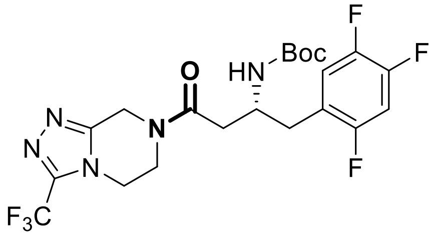

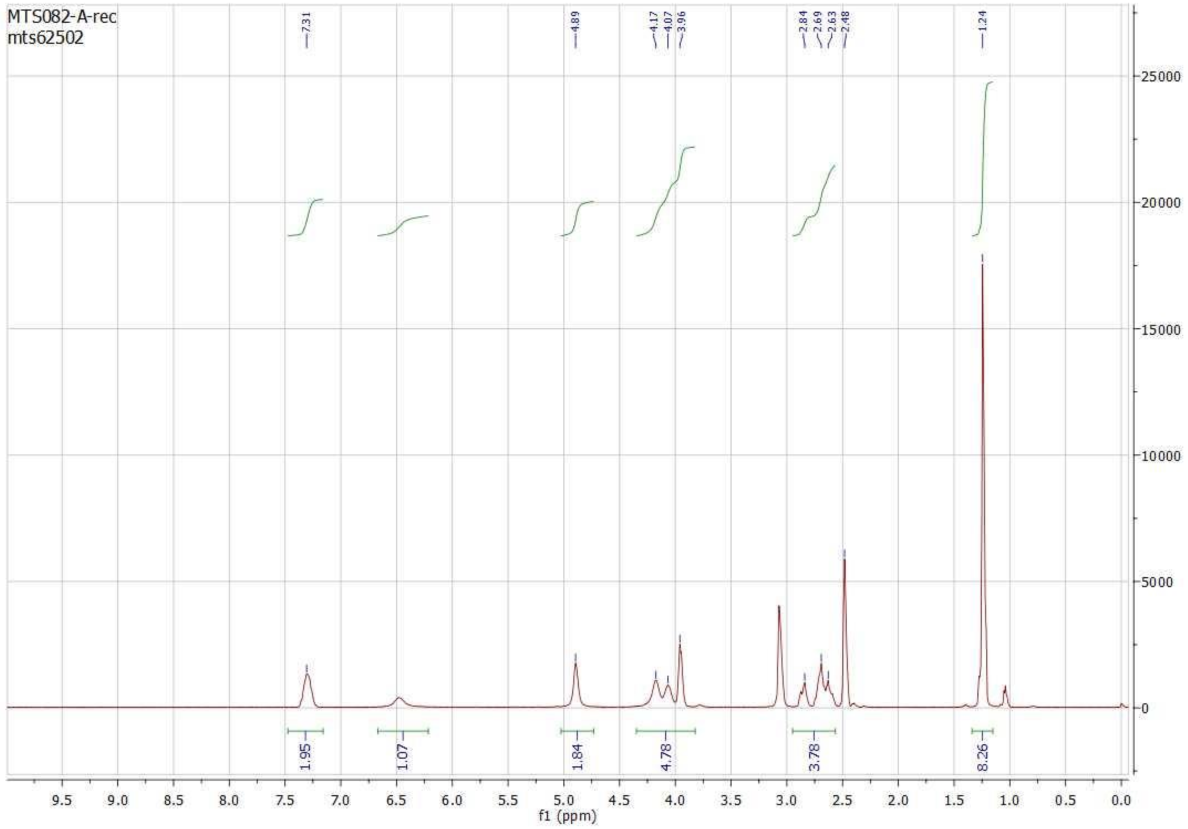

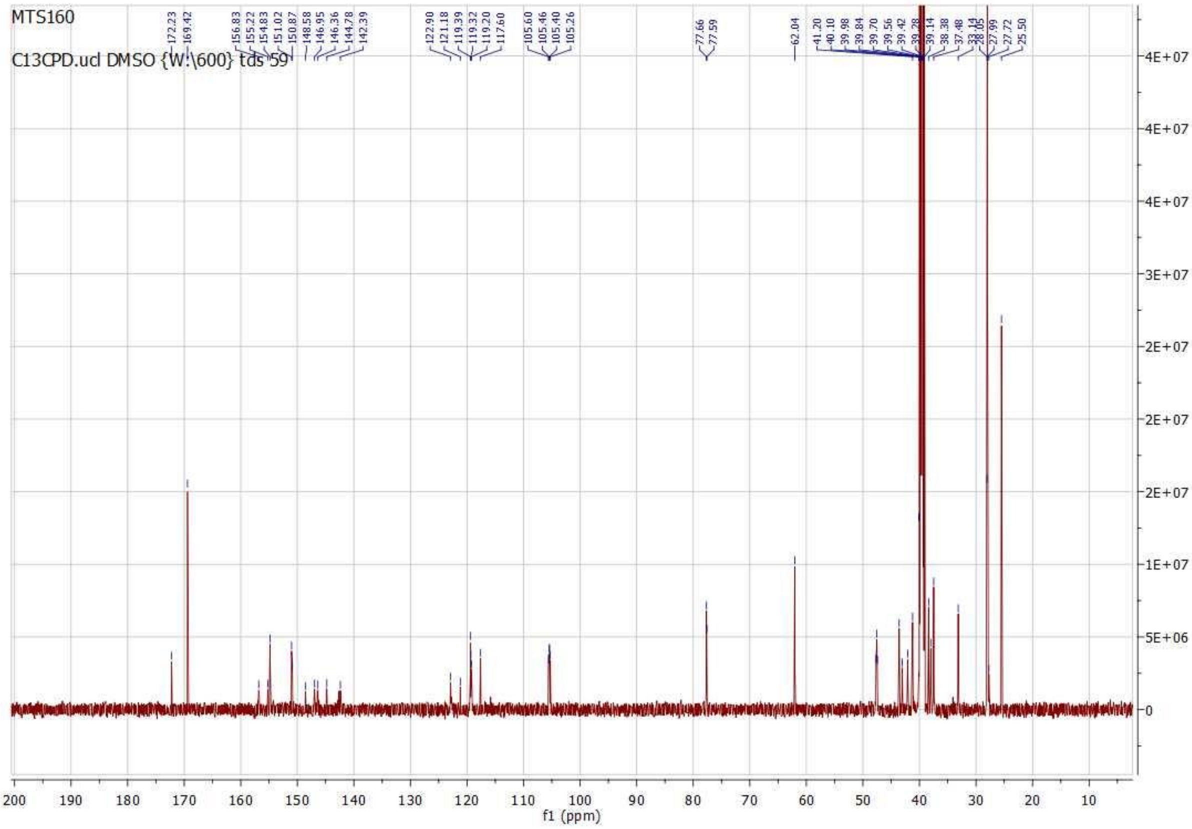

(*R*)-3-amino-1-(3-(trifluoromethyl)-5,6-dihydro-[1,2,4]triazolo[4,3-a]pyrazin-7(8H)-yl)-4-(2,4,5-trifluorophenyl)butan-1-one (Sitagliptin, 72)

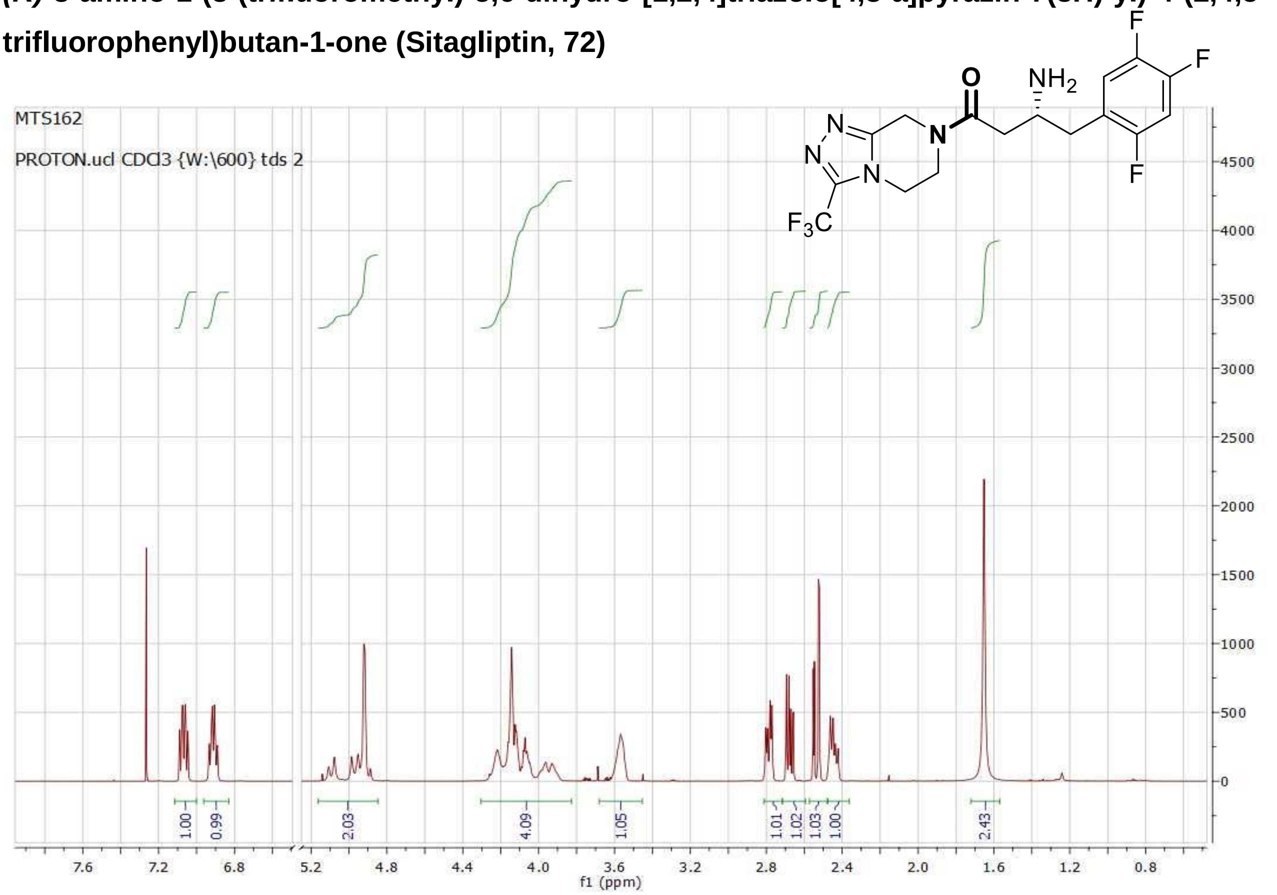

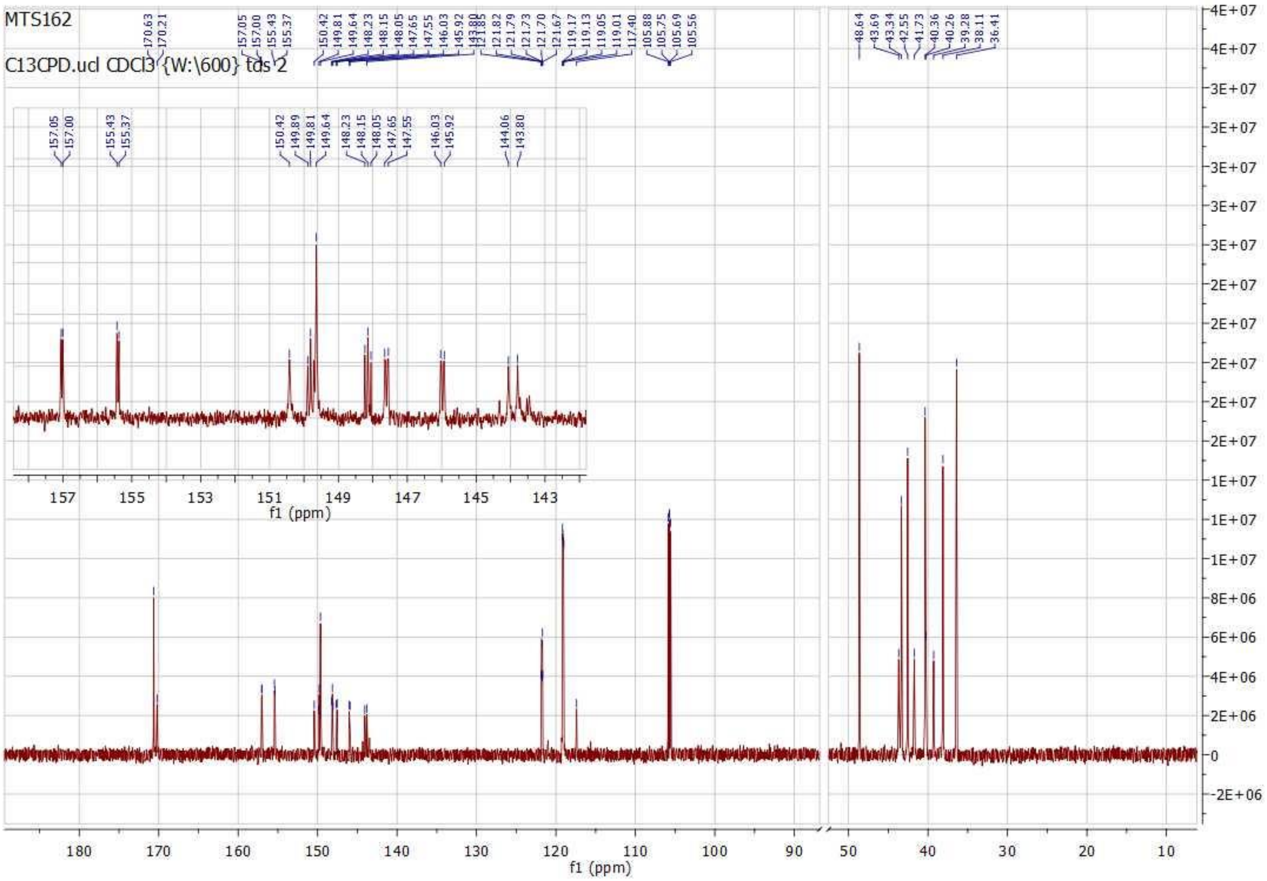

***(R)*-5-(piperidine-1-carbonyl)pyrrolidin-2-one (73)**

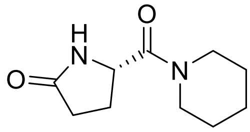

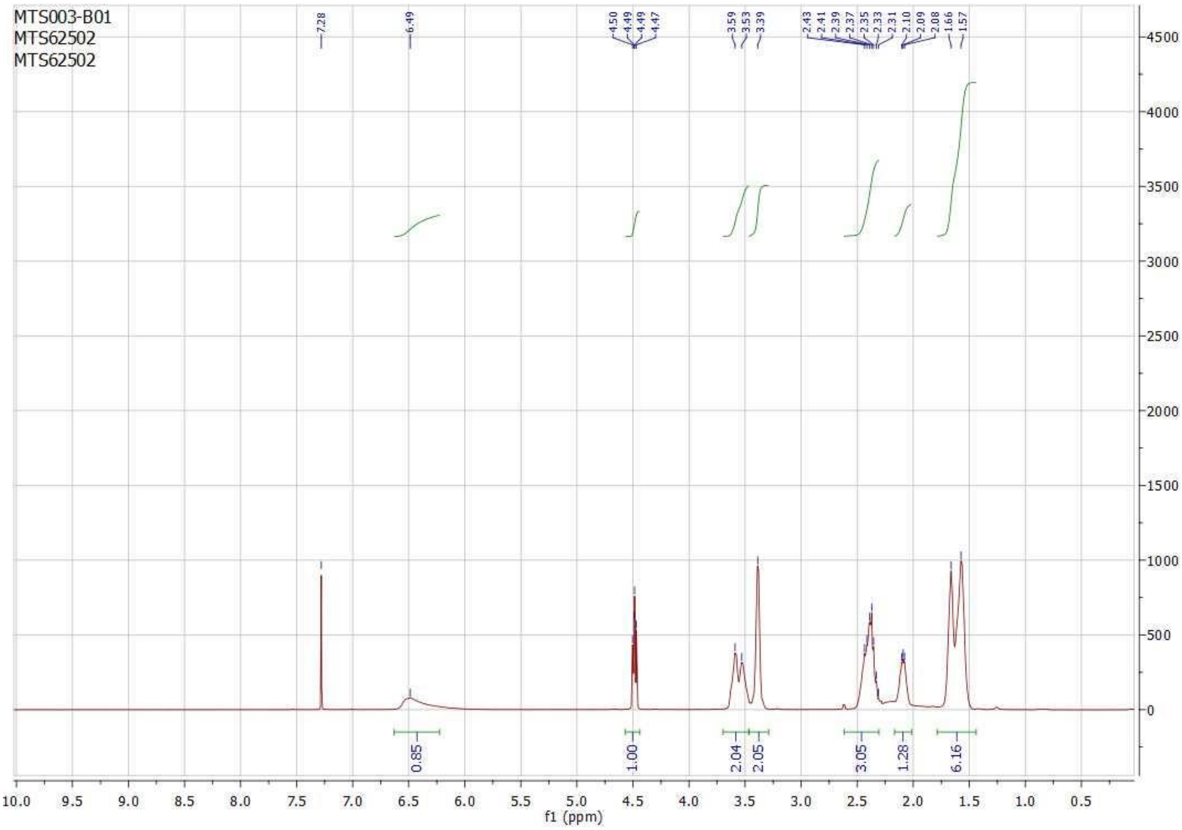

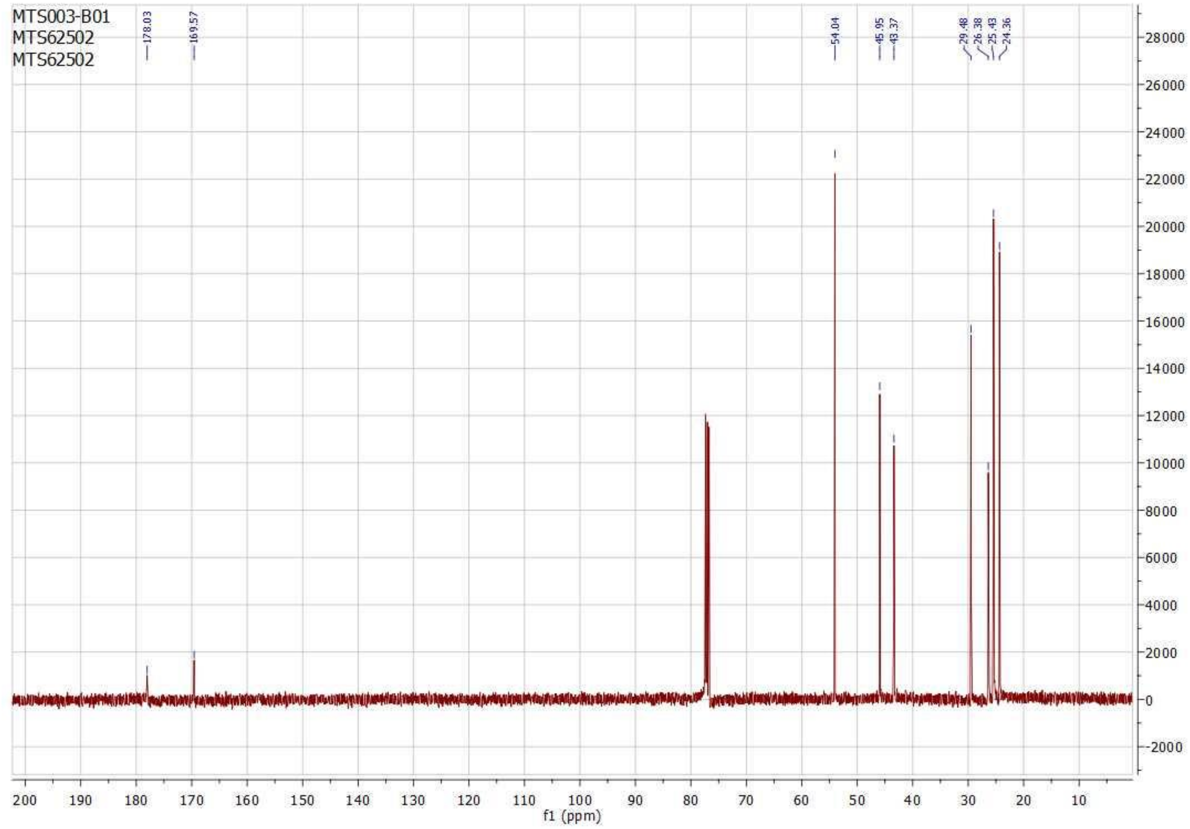

***N*-(4-methoxybenzyl)-2-(thiophen-2-yl)acetamide (74)**

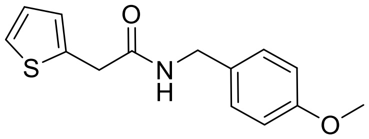

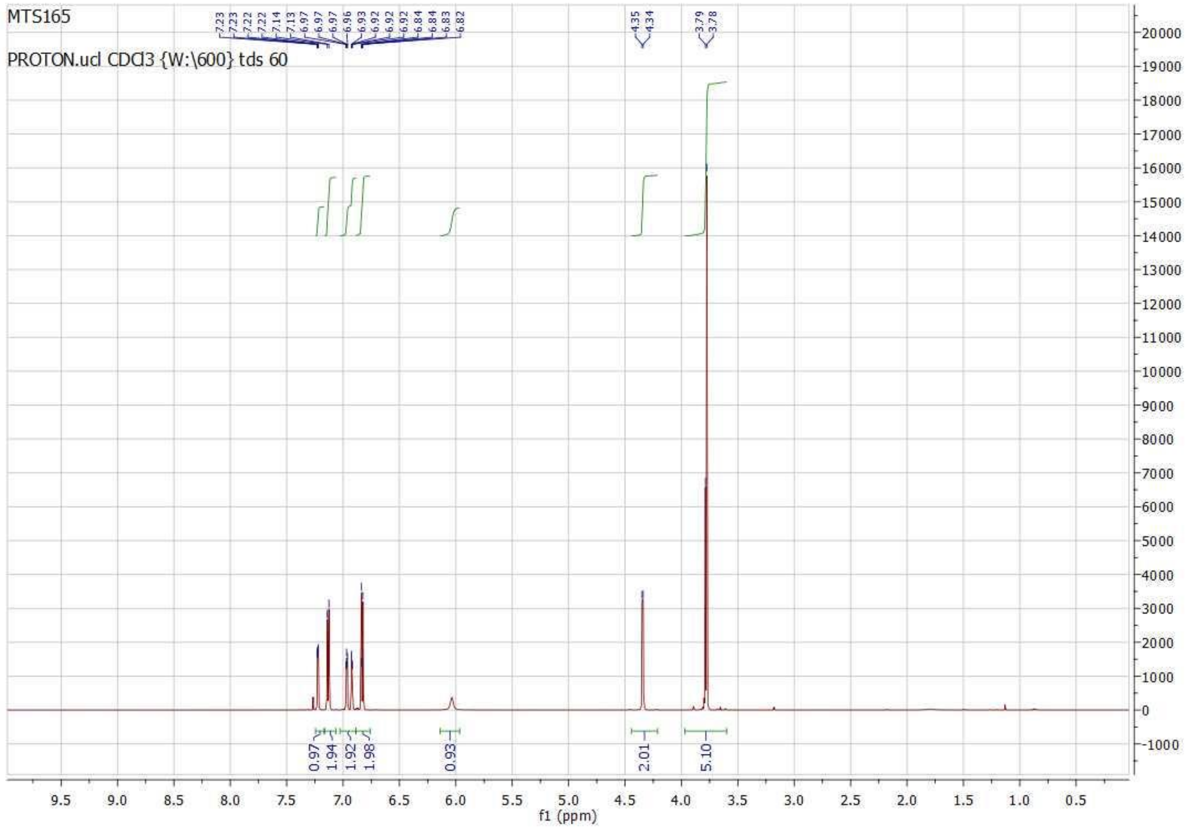

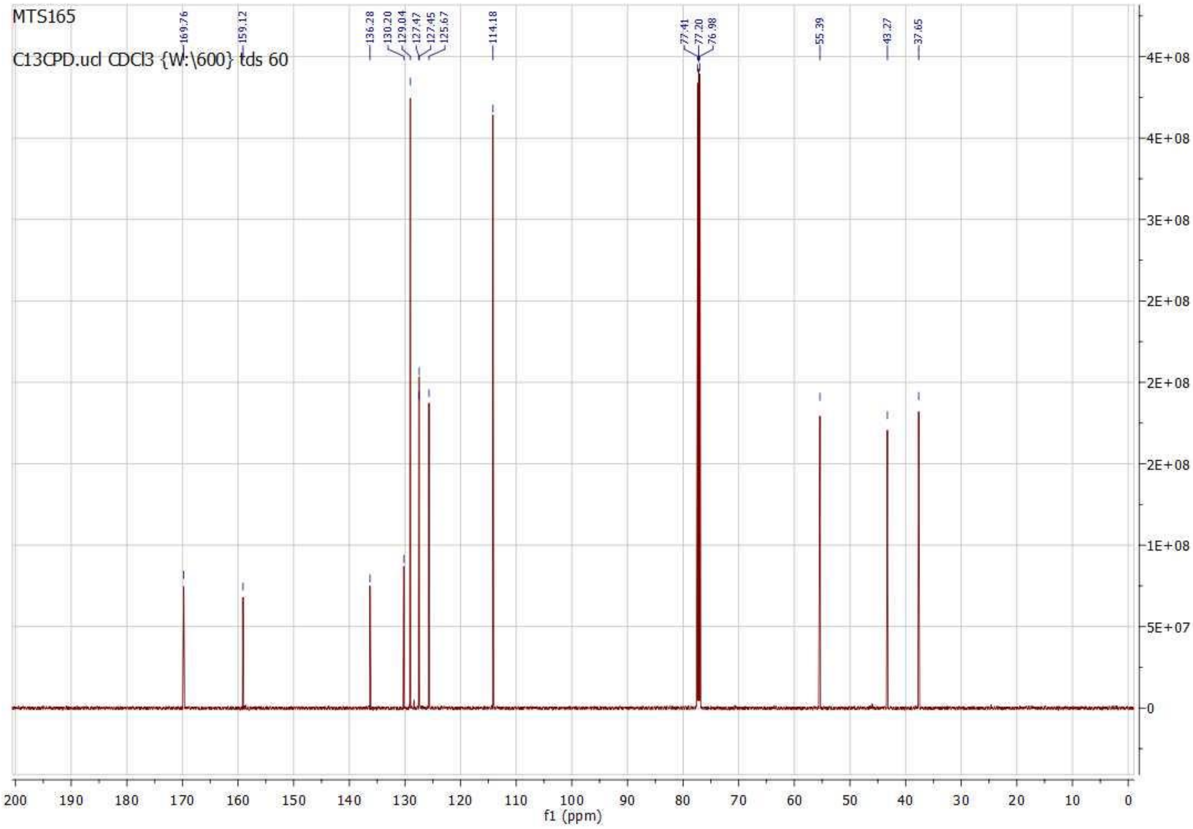

2,2,2-trifluoroethyl benzoate (76)

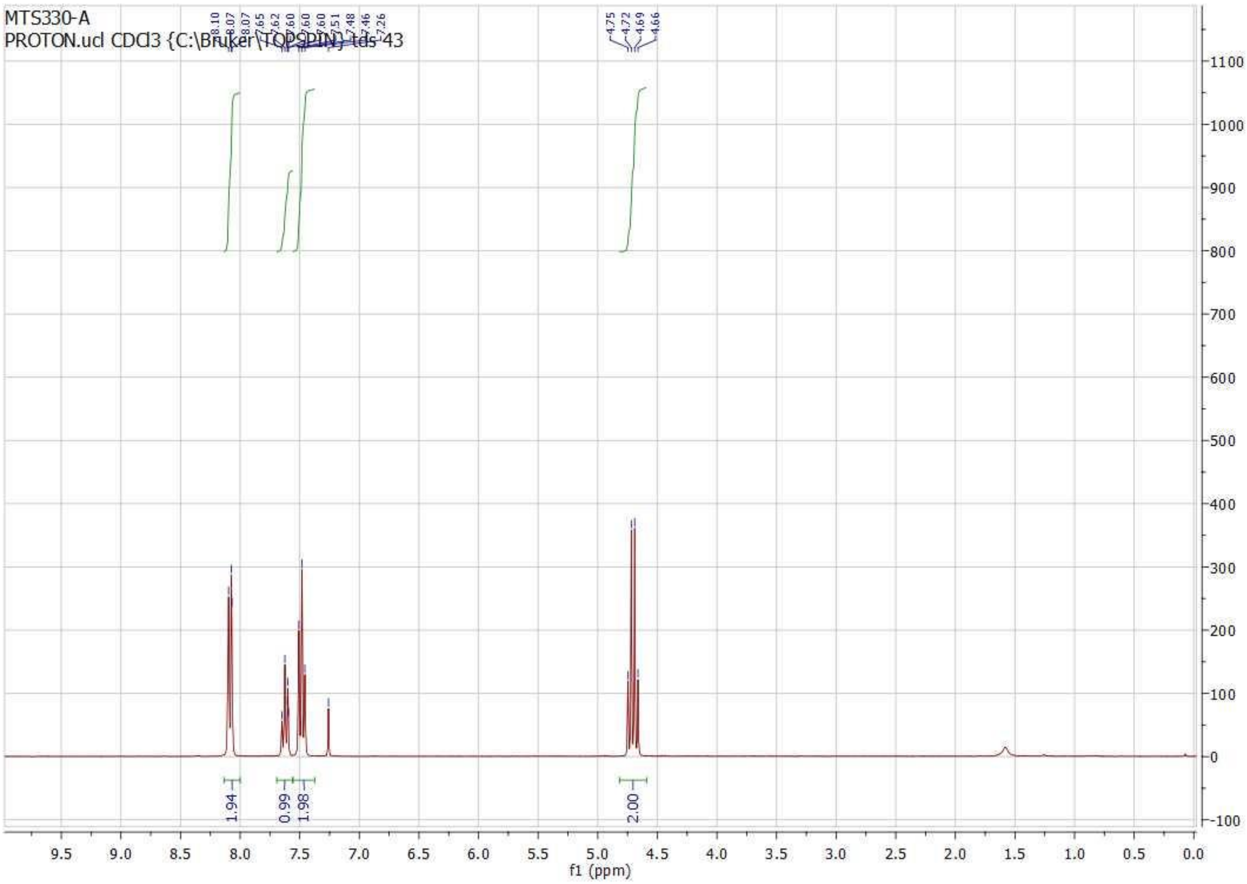

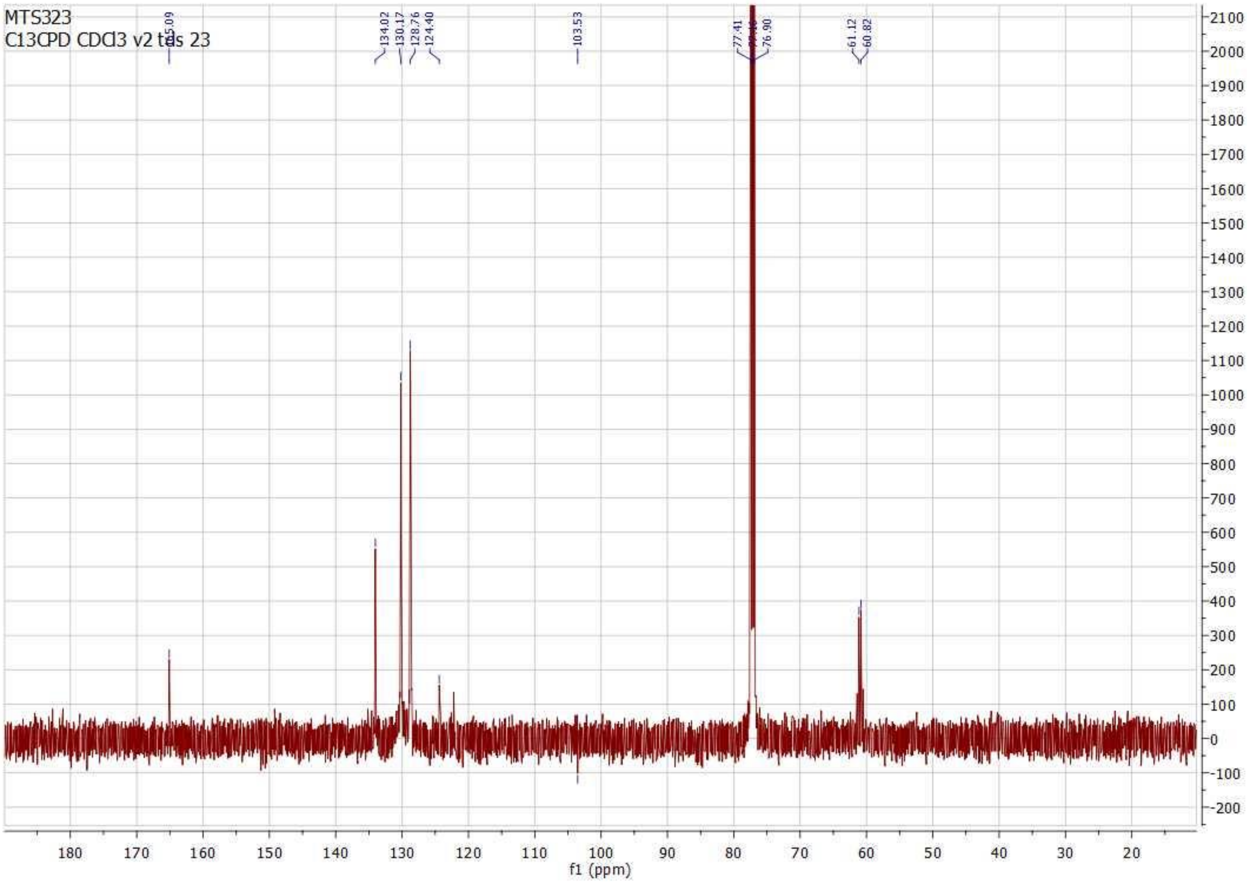

Chiral HPLC traces for enantiopurity measurements

(*R*)-2-phenyl-N-(1-phenylethyl)acetamide (6)

Conditions: OD1, 90:10 hexane/propanol, 0.8 ml/min, 218 nm

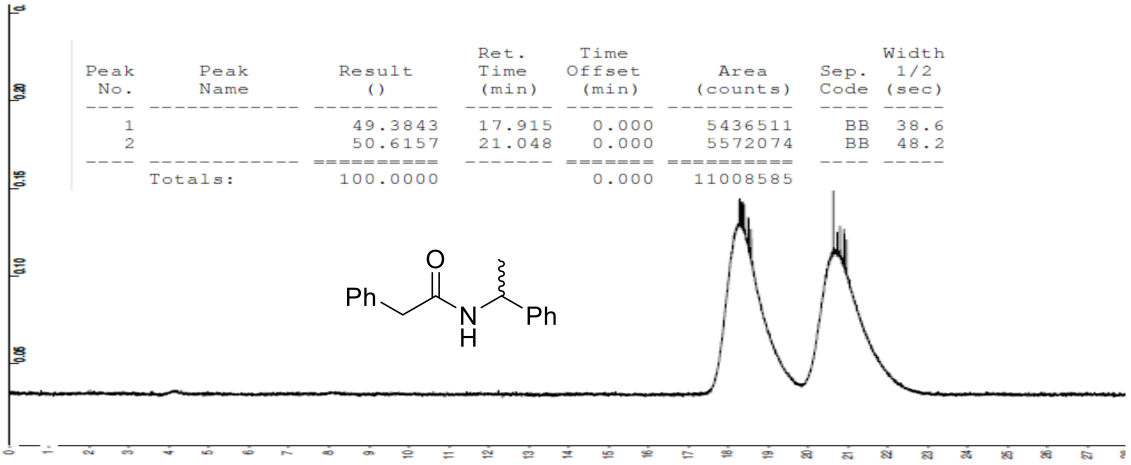

Peak No.	Peak Name	Result ()	Ret. Time (min)	Time Offset (min)	Area (counts)	Sep. Code	Width 1/2 (sec)
1		49.3843	17.915	0.000	5436511	BB	38.6
2		50.6157	21.048	0.000	5572074	BB	48.2
	Totals:	100.0000		0.000	11008585		

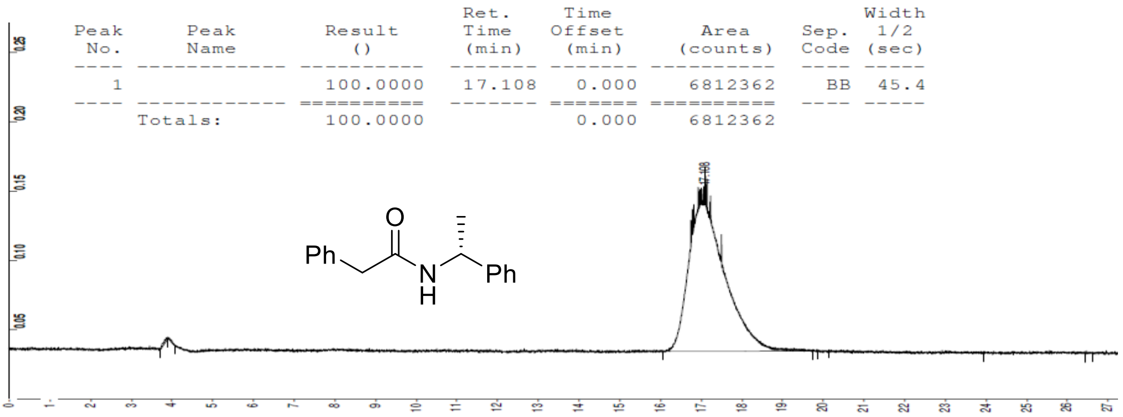

Peak No.	Peak Name	Result ()	Ret. Time (min)	Time Offset (min)	Area (counts)	Sep. Code	Width 1/2 (sec)
1		100.0000	17.108	0.000	6812362	BB	45.4
	Totals:	100.0000		0.000	6812362		

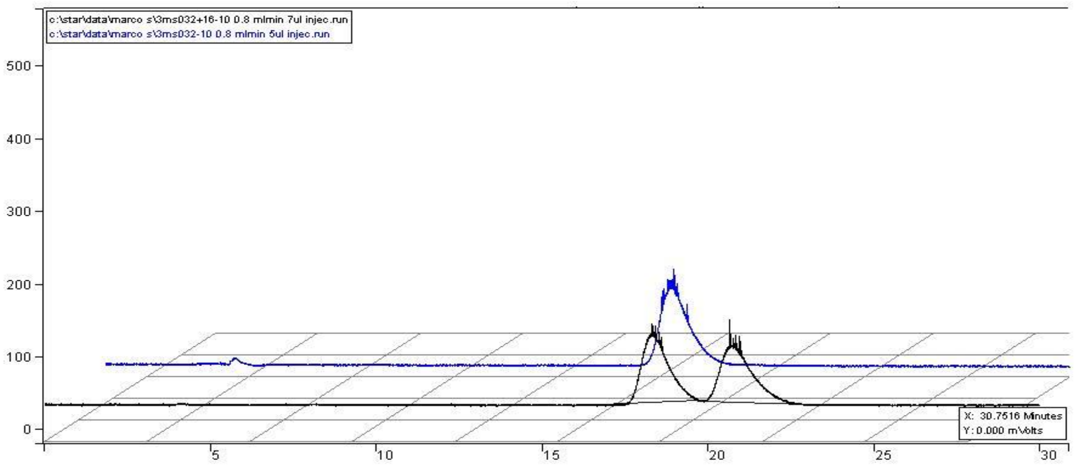

(*S*)-N-Benzyl-2-(4-isobutylphenyl)propanamide ((*S*)-Ibuprofen amide (31))

commercial starting material [98.5% ee] on next page

Conditions: ADH, 90:10, 0.8 ml/min , 220 nm

Peak No.	Peak Name	Result ()	Ret. Time (min)	Time Offset (min)	Area (counts)	Sep. Code	Width 1/2 (sec)
1		49.4	12.215	0.000	7178376	BB	35.3
2		50.6	21.012	0.000	7366219	BB	38.4
	Totals:	100.0		0.000	14544595		

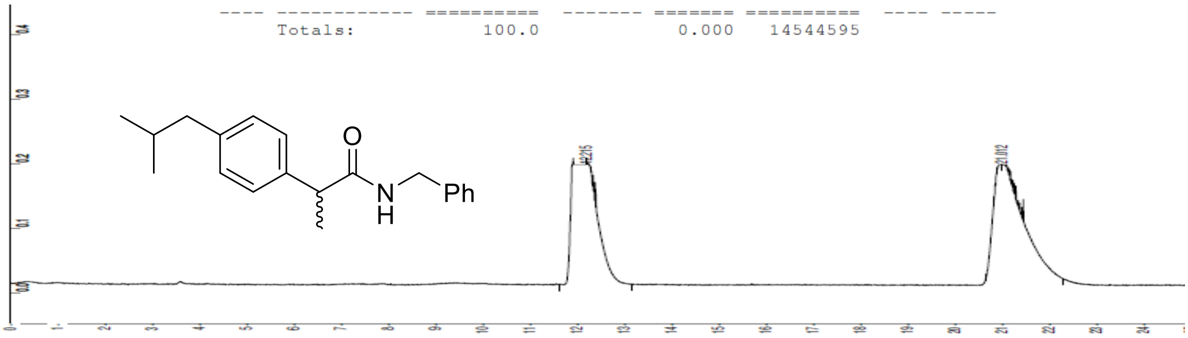

Peak No.	Peak Name	Result ()	Time (min)	Offset (min)	Area (counts)	Sep. Code	1/2 (sec)	Status Codes
1		98.5335	12.395	0.000	10929819	BB	31.9	
2		1.4665	23.265	0.000	162676	BB	31.1	
	Totals:	100.0000		0.000	11092495			

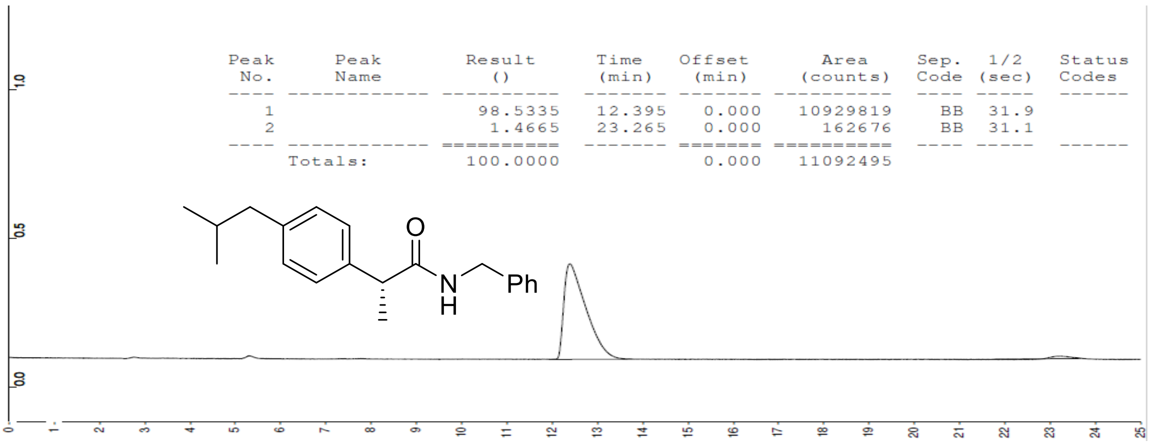

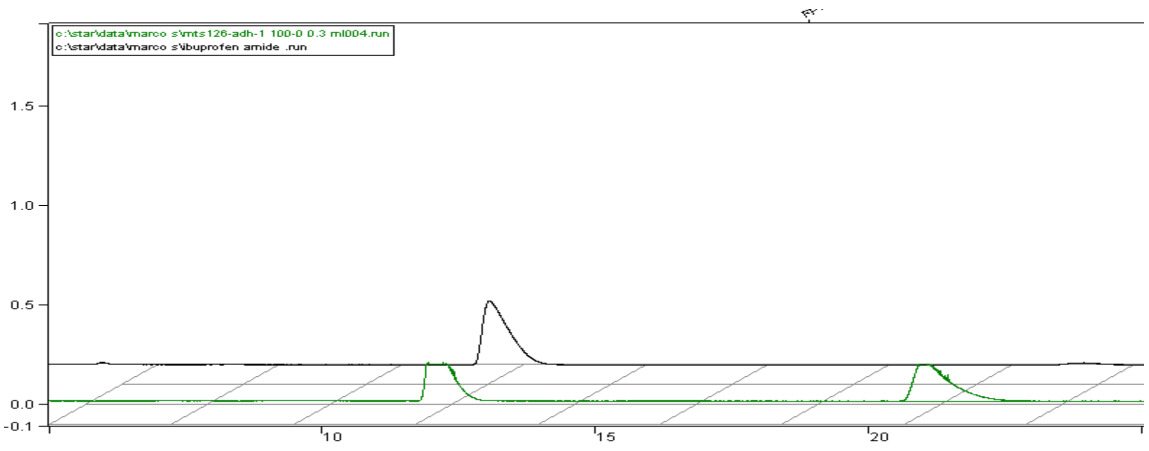

(*S*)-Ibuprofen starting material (for the use in the synthesis of ibuprofen amide **31**)

Conditions: ADH, 97:3 hexane/propanol, 0.8 ml/min , 220 nm

Peak No.	Peak Name	Result ()	Time (min)	Offset (min)	Area (counts)	Sep. Code	1/2 (sec)	Status Codes
1		0.0079	0.456	0.000	1396	BB	0.0	
2		0.0218	29.443	0.000	3841	BB	0.0	
3		0.0085	30.722	0.000	1506	BB	0.9	
4		50.2459	32.668	0.000	8857040	BB	36.6	
5		49.6569	34.635	0.000	8753224	BB	39.3	
	Totals:	100.0000		0.000	17627401			

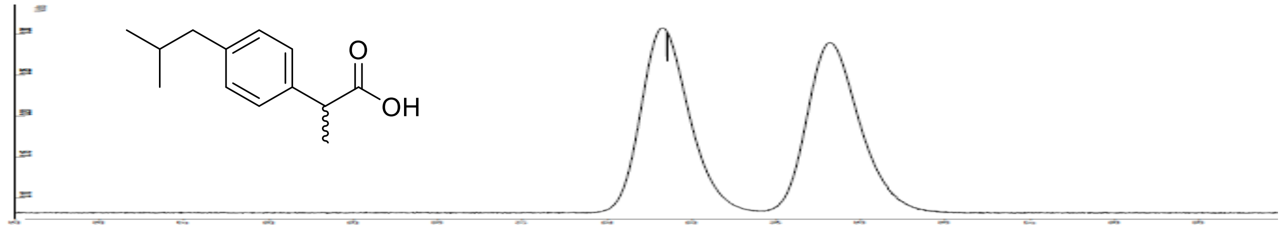

Peak No.	Peak Name	Result ()	Ret. Time (min)	Time Offset (min)	Area (counts)	Sep. Code	Width 1/2 (sec)	Status Codes
1		99.2337	32.645	0.000	21870848	BB	35.0	
2		0.7663	34.398	0.000	168900	BB	16.1	
	Totals:	100.0000		0.000	22039748			

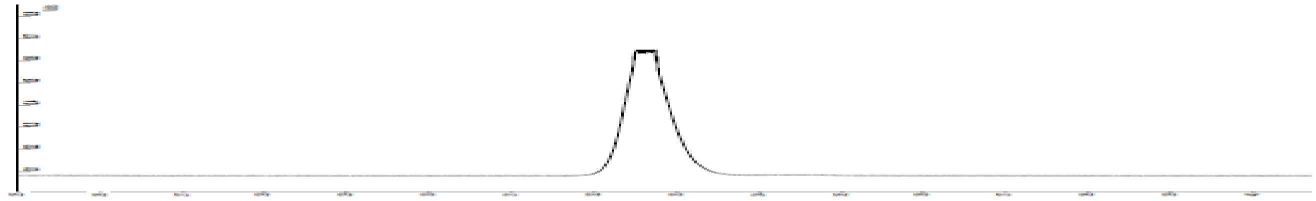

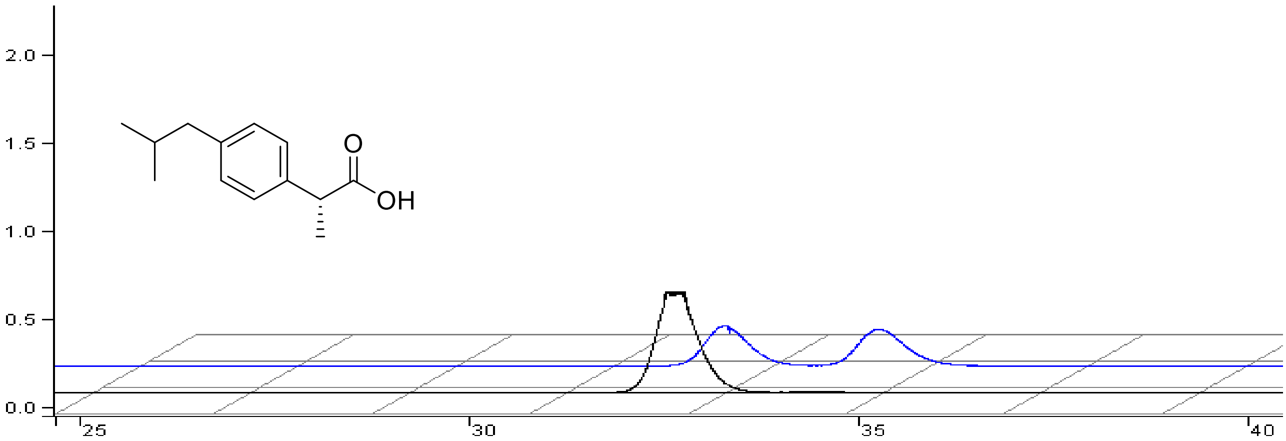

***Tert*-butyl (*S*)-(1-(benzylamino)-1-oxopropan-2-yl)carbamate (37)**

Condition: OD1, 90:10 hexane/propanol, 0.8 ml/min, 254 nm

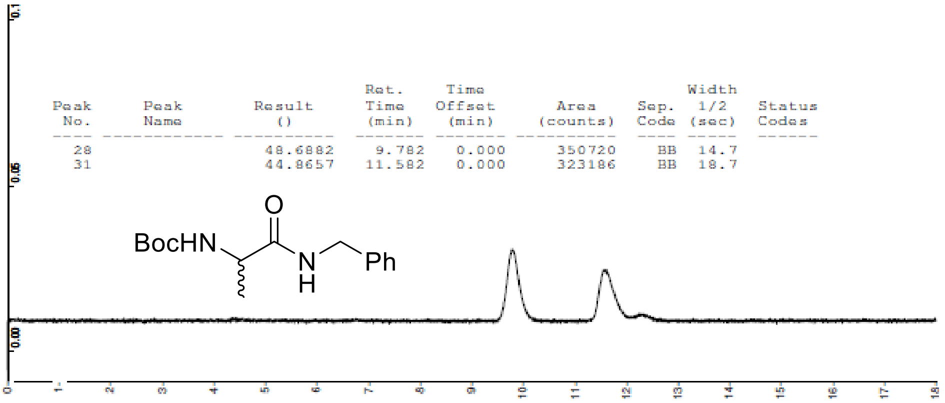

Peak No.	Peak Name	Result ()	Ret. Time (min)	Time Offset (min)	Area (counts)	Sep. Code	Width 1/2 (sec)	Status Codes
28		48.6882	9.782	0.000	350720	BB	14.7	
31		44.8657	11.582	0.000	323186	BB	18.7	

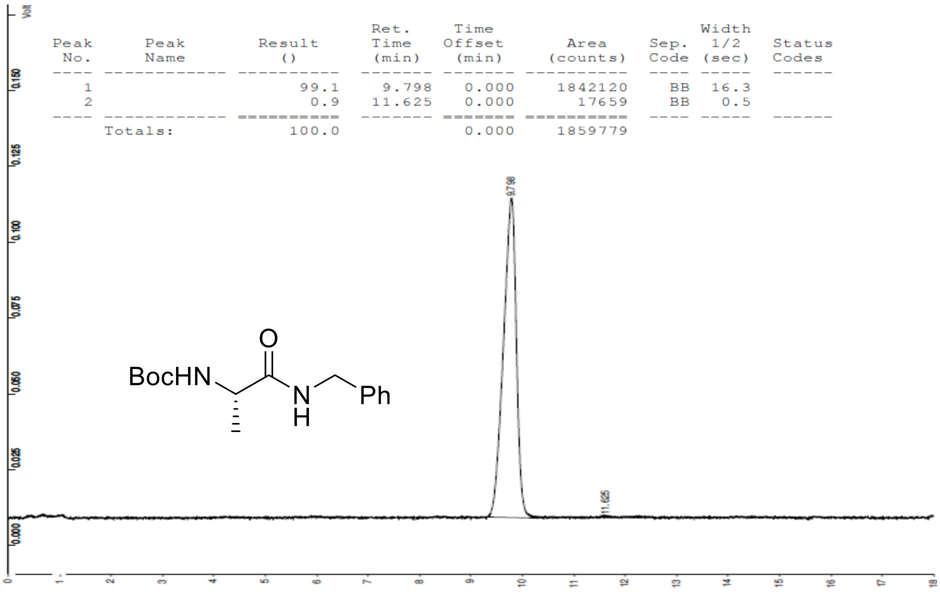

Peak No.	Peak Name	Result ()	Ret. Time (min)	Time Offset (min)	Area (counts)	Sep. Code	Width 1/2 (sec)	Status Codes
1		99.1	9.798	0.000	1842120	BB	16.3	
2		0.9	11.625	0.000	17659	BB	0.5	
	Totals:	100.0		0.000	1859779			

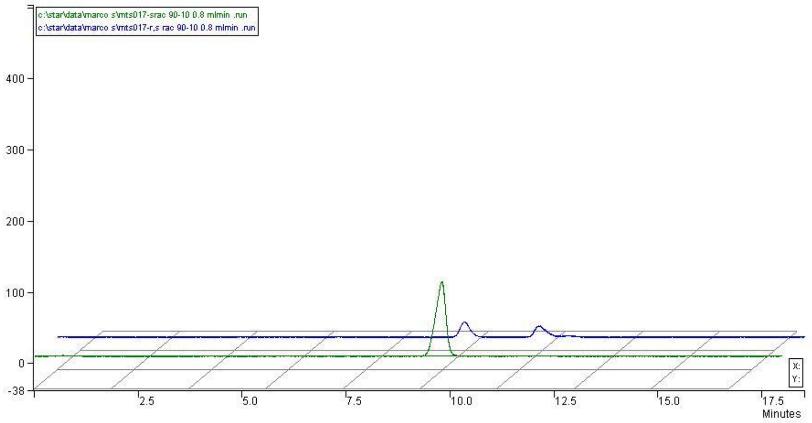

tert-butyl (S)-2-(benzylcarbamoyl)pyrrolidine-1-carboxylate (_39_)

conditions: OD1 column, 90:10 hexane/^{i}PrOH, flow 0.8 mL/min, 218 nm

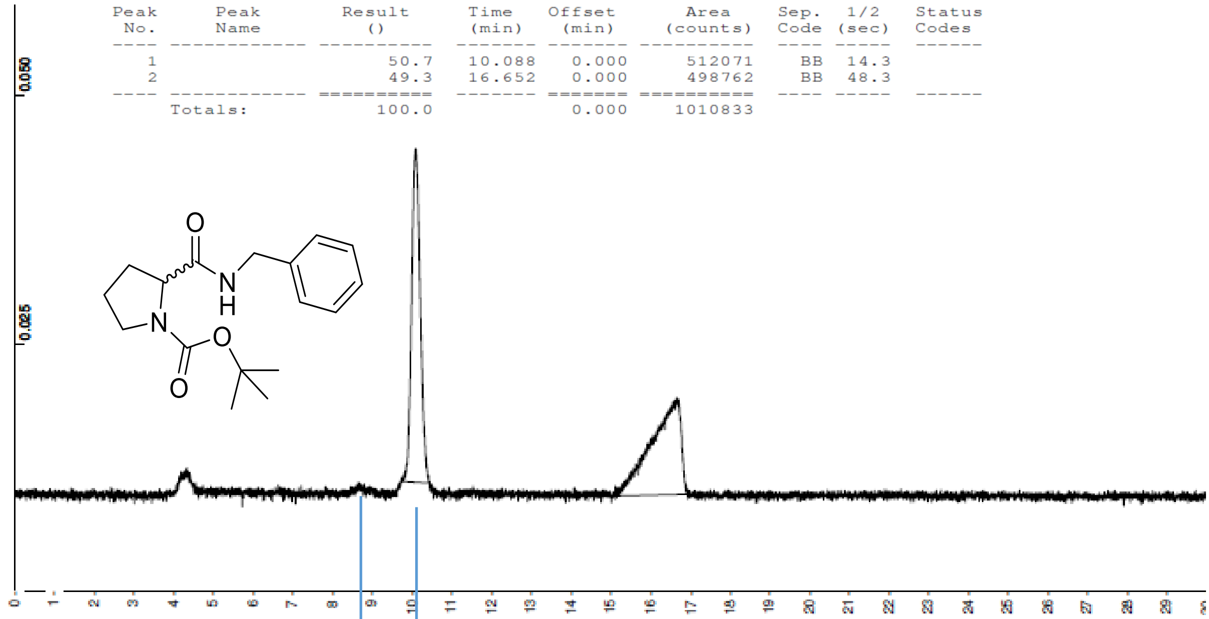

Peak No.	Peak Name	Result ()	Time (min)	Offset (min)	Area (counts)	Sep. Code	1/2 (sec)	Status Codes
1		50.7	10.088	0.000	512071	BB	14.3	
2		49.3	16.652	0.000	498762	BB	48.3	
	Totals:	100.0		0.000	1010833			

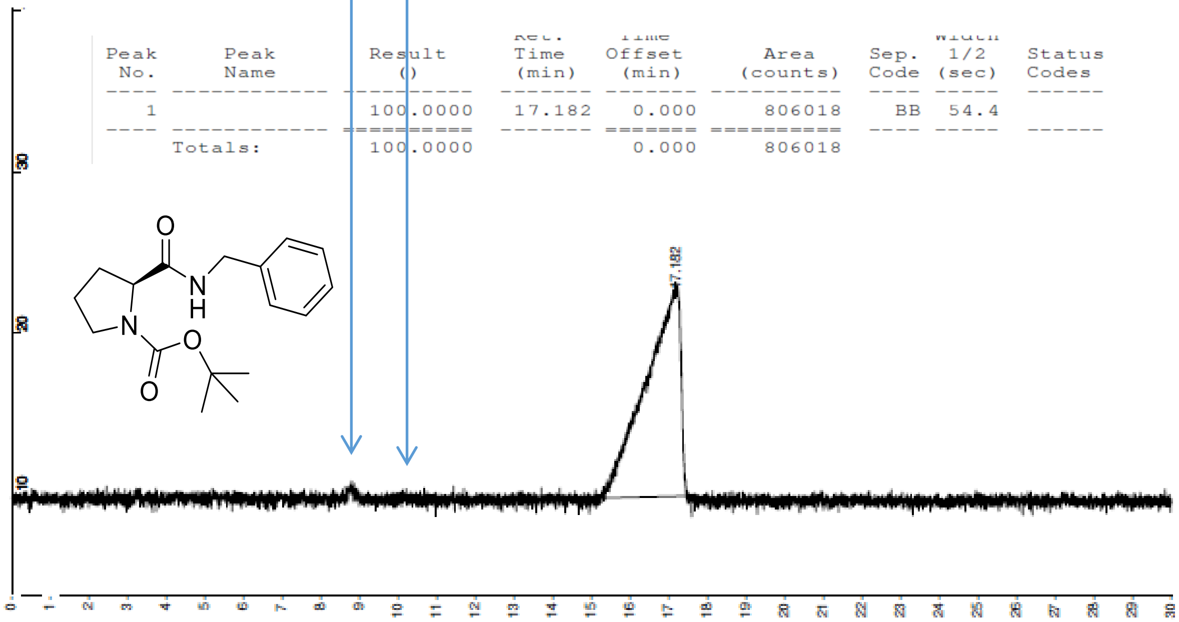

Peak No.	Peak Name	Result ()	Time (min)	Offset (min)	Area (counts)	Sep. Code	1/2 (sec)	Status Codes
1		100.0000	17.182	0.000	806018	BB	54.4	
	Totals:	100.0000		0.000	806018			

***(S)*-3,5-dibenzyl-2,2-dimethylimidazolidin-4-one (61)**

Conditions: 25cm Chiralpak AD-H, 20%ETOH/heptane [0.1%isopropylamine],1.0 ml/min, 235 nm

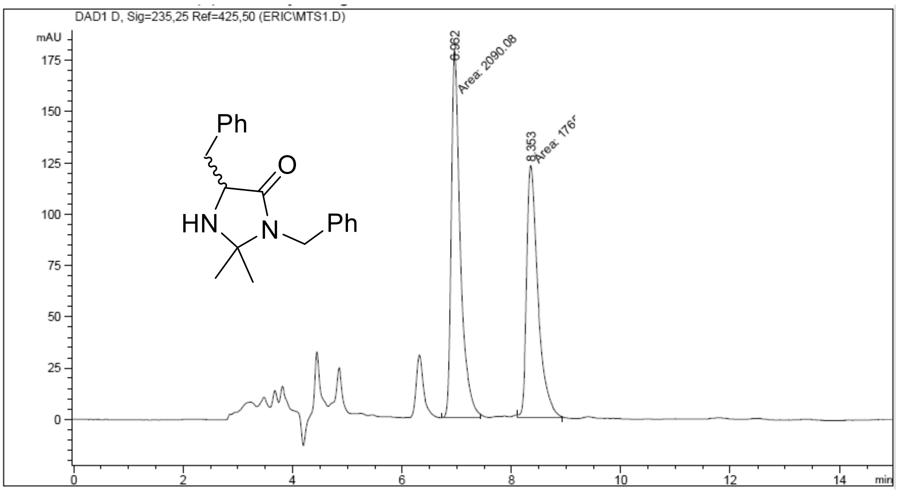

Peak #	RetTime [min]	Type	Width [min]	Area [mAU*s]	Height [mAU]	Area %
1	6.962	MM	0.1940	2090.07886	179.51640	54.2084
2	8.353	MM	0.2391	1765.55469	123.07912	45.7916

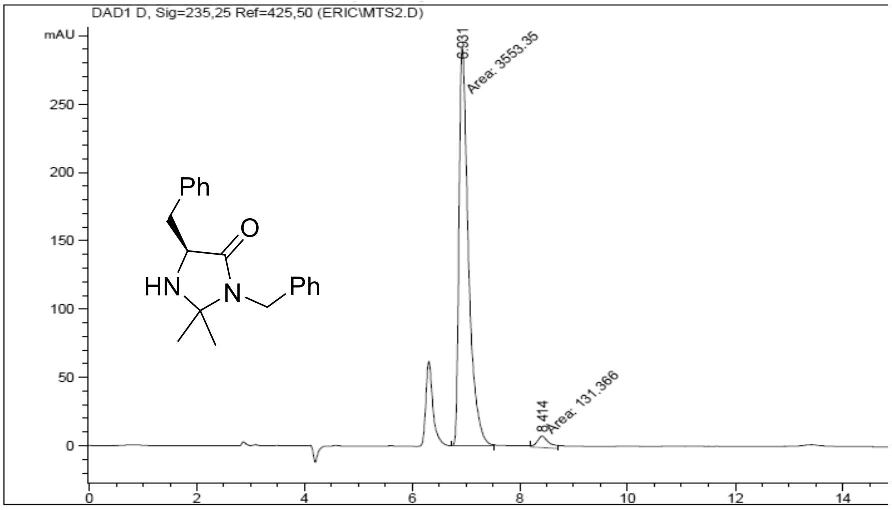

Peak #	RetTime [min]	Type	Width [min]	Area [mAU*s]	Height [mAU]	Area %
1	6.931	MM	0.2030	3553.35083	291.75360	96.4349
2	8.414	MM	0.2583	131.36555	8.47591	3.5651

(*2S,5S*)-3,5-dibenzyl-2-(*tert*-butyl)imidazolidin-4-one (62 B)

Conditions: 25cm Chiralpak AD-H, 20%ETOH/heptane [0.1%isopropylamine],1.0 ml /min, 235nm

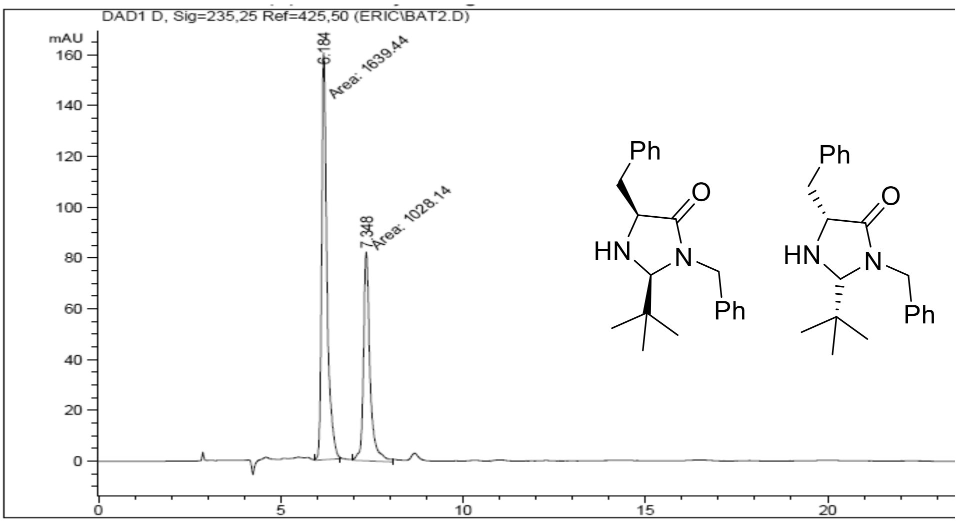

Peak #	RetTime [min]	Type	Width [min]	Area [mAU*s]	Height [mAU]	Area %
1	6.184	MM	0.1701	1639.43750	160.65372	61.4578
2	7.348	MM	0.2084	1028.14441	82.20559	38.5422

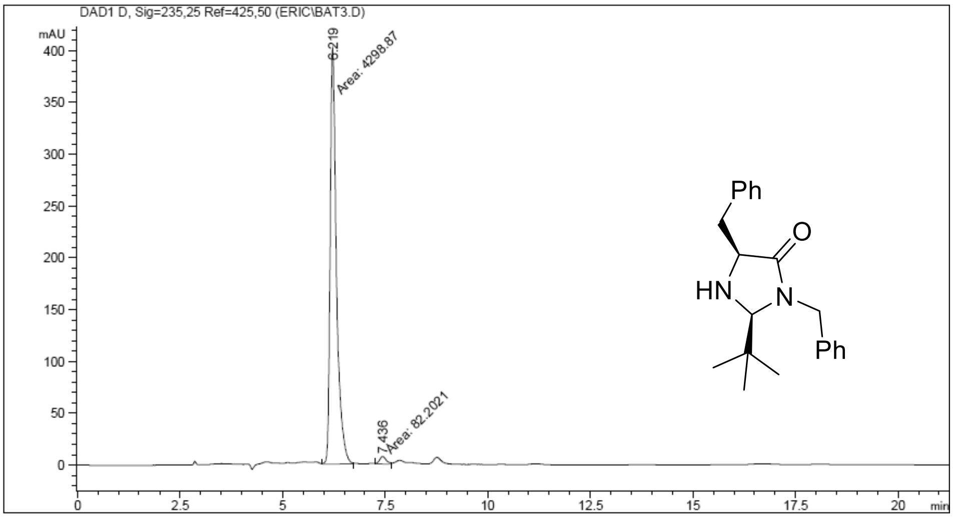

Peak #	RetTime [min]	Type	Width [min]	Area [mAU*s]	Height [mAU]	Area %
1	6.219	MM	0.1781	4298.87061	402.39188	98.1237
2	7.436	MM	0.1897	82.20207	7.22232	1.8763

(*2R,5S*)-3,5-dibenzyl-2-(*tert*-butyl)imidazolidin-4-one (62 A)

Conditions: 25cm Chiralpak AD-H, 20%ETOH/heptane [0.1%isopropylamine],1.0 ml /min, 235nm

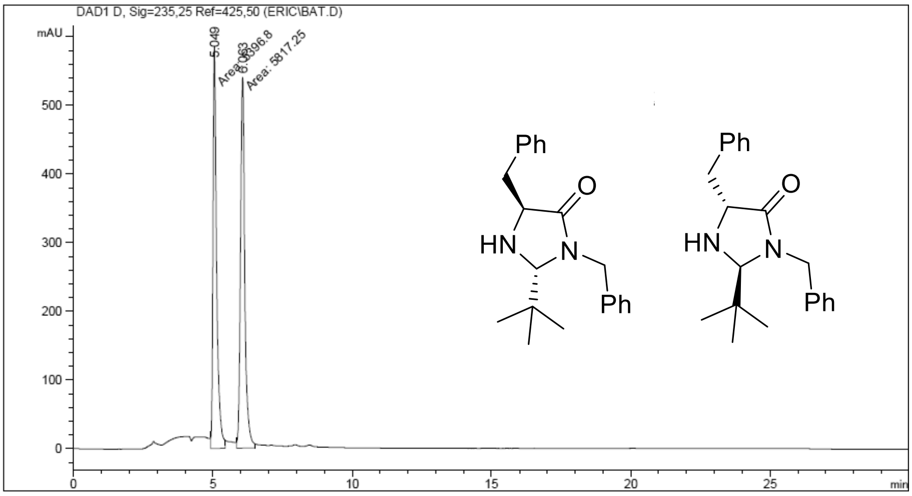

Peak #	RetTime [min]	Type	Width [min]	Area [mAU*s]	Height [mAU]	Area %
1	5.049	FM	0.1529	5396.80029	588.11823	48.1253
2	6.063	FM	0.1790	5817.25342	541.78381	51.8747

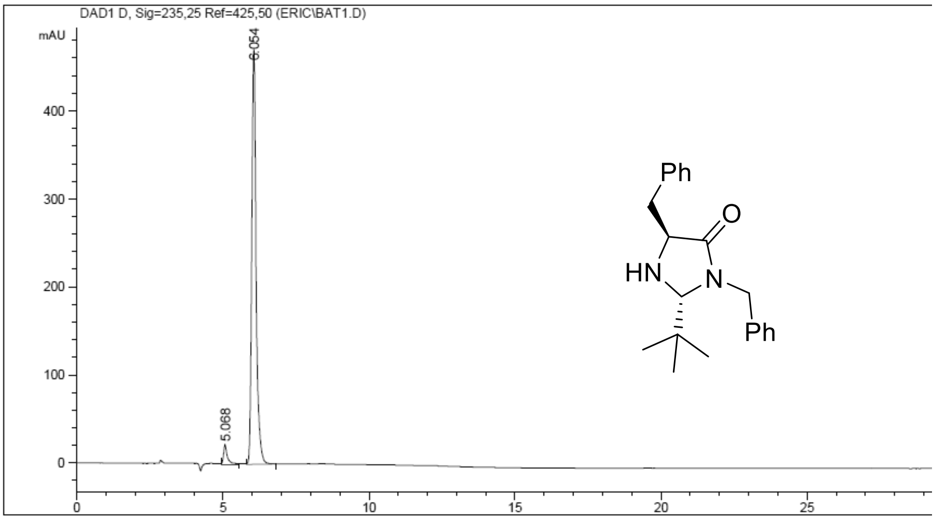

Peak #	RetTime [min]	Type	Width [min]	Area [mAU*s]	Height [mAU]	Area %
1	5.068	BB	0.1425	226.31715	23.05938	4.4387
2	6.054	BB	0.1565	4872.43213	471.67538	95.5613

***Tert*-butyl pentanoyl-L-valinate (66)**

Conditions OD-1, 97:3 hexane/propanol, 0.8 ml/min, 218 nm

Commercial starting material [99% ee]

Peak No.	Peak Name	Result ()	Time (min)	Offset (min)	Area (counts)	Sep. Code	1/2 (sec)	Status Codes
1		47.2559	6.595	0.000	2030089	BB	24.9	
2		52.7441	28.475	0.000	2265863	BB	97.6	
	Totals:	100.0000		0.000	4295952			

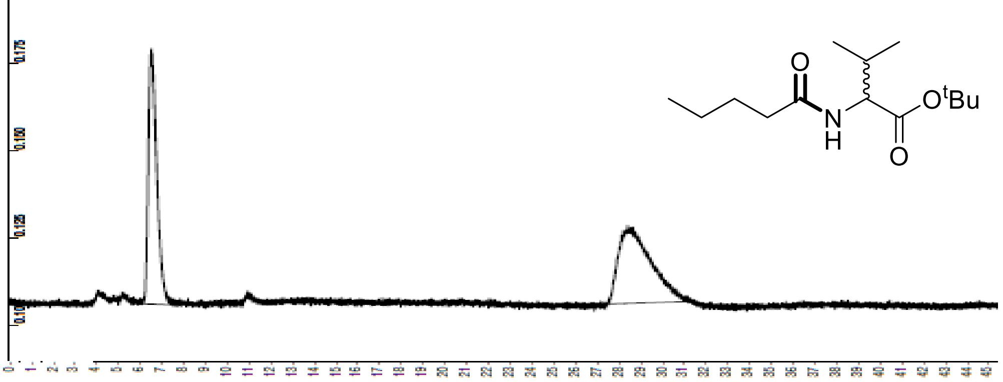

Peak No.	Peak Name	Result ()	Time (min)	Offset (min)	Area (counts)	Sep. Code	1/2 (sec)	Status Codes
1		0.7599	6.892	0.000	21923	BB	0.8	
2		99.2401	29.558	0.000	2863111	BB	113.8	
	Totals:	100.0000		0.000	2885034			

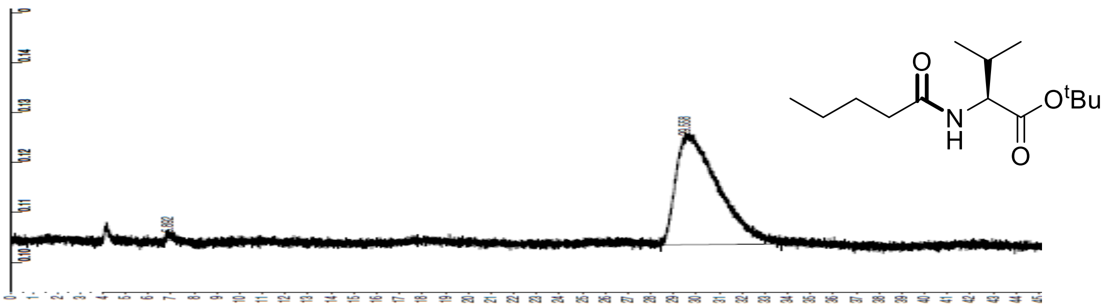

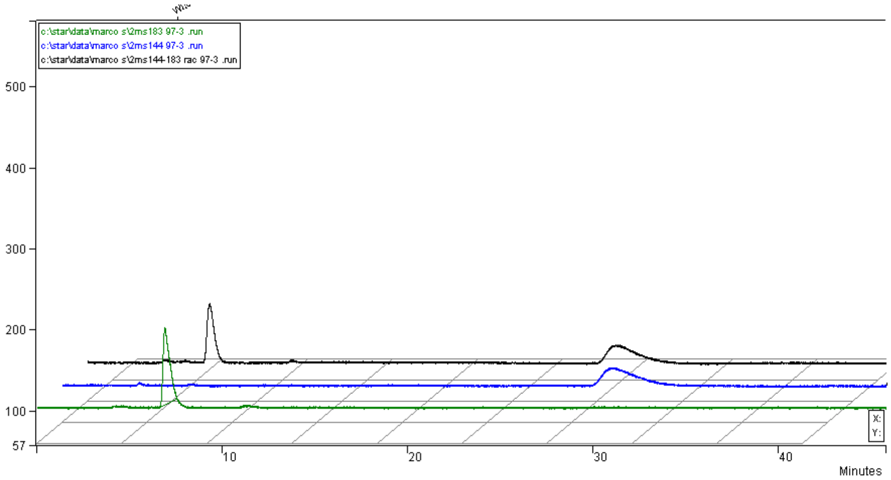

(*R*)-5-(piperidine-1-carbonyl)pyrrolidin-2-one (73, Fasoracetam)

Conditions: Chiralpak IA, 40%EtOH/heptane[0.1%isopropylamine],1.0 ml/min, 235nm

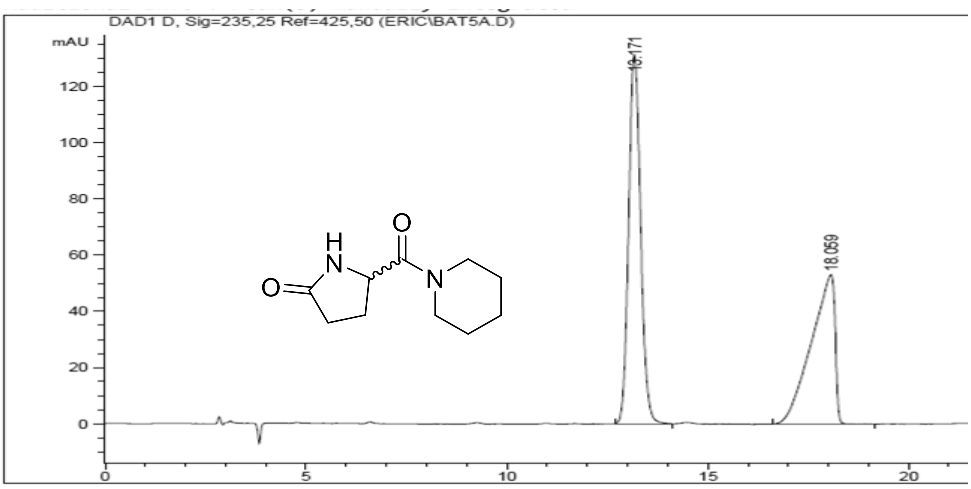

Peak #	RetTime [min]	Type	Width [min]	Area [mAU*s]	Height [mAU]	Area %
1	13.171	BB	0.3227	2730.39404	131.24663	55.5072
2	18.059	BB	0.5648	2188.59839	53.11140	44.4928

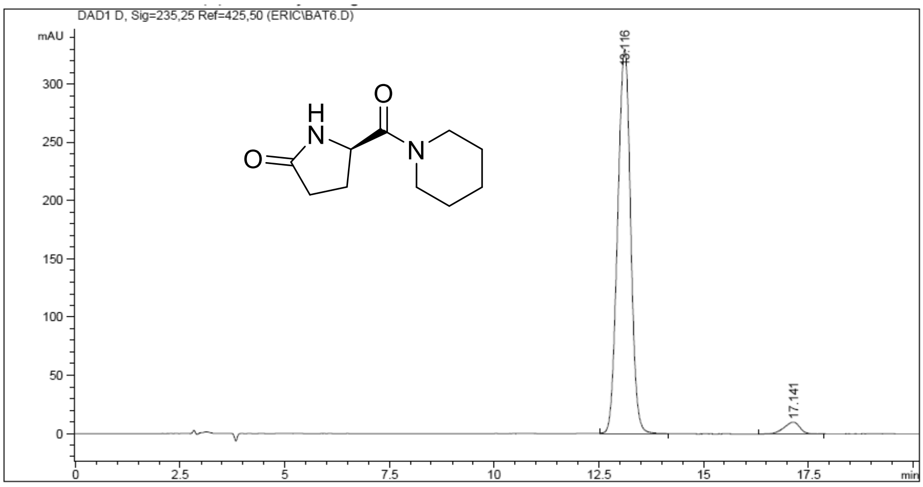

Peak #	RetTime [min]	Type	Width [min]	Area [mAU*s]	Height [mAU]	Area %
1	13.116	BB	0.3395	7113.08105	329.97043	96.4002
2	17.141	BB	0.4005	265.61682	10.17583	3.5998

^{1}H and ^{13}C NMR spectra for enantiopurity measurements

9.a NMR’s of dipeptide diastereoisomers (overlayed NMR’s)

benzyl (*tert*-butoxycarbonyl)-L-phenylalanyl-L-valinate (40)

Overlay of spectra of Boc-L-Phe-L-Val-OBn (40) and Boc-D-Phe-L-Val-OBn

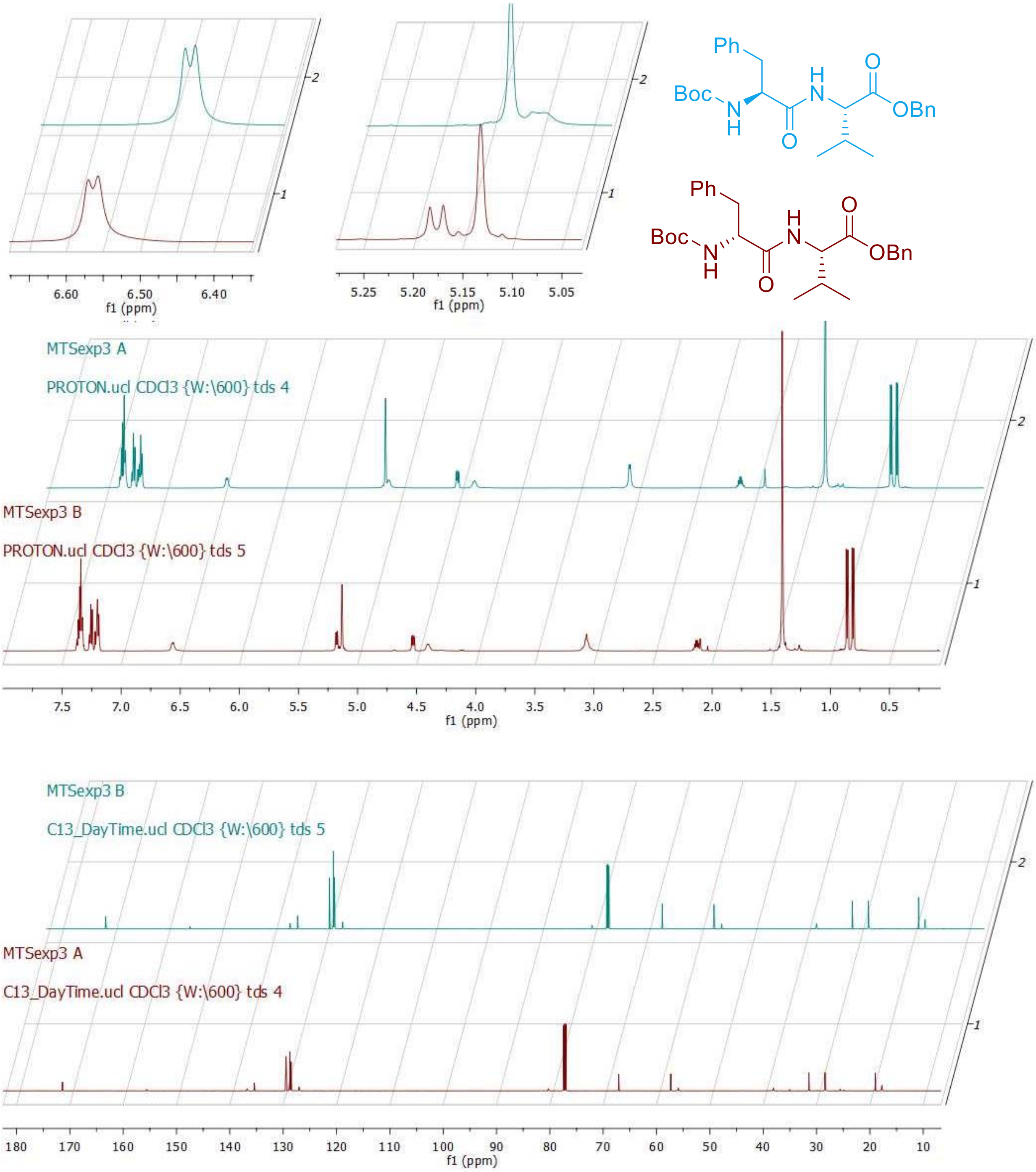

tert-butyl (tert-butoxycarbonyl)-D-alanyl-L-valinate (41)

Overlay of spectra of Boc-D-Ala-L-Val-O^{t}Bu (41) and Boc-DL-Ala-L-Val-O^{t}Bu

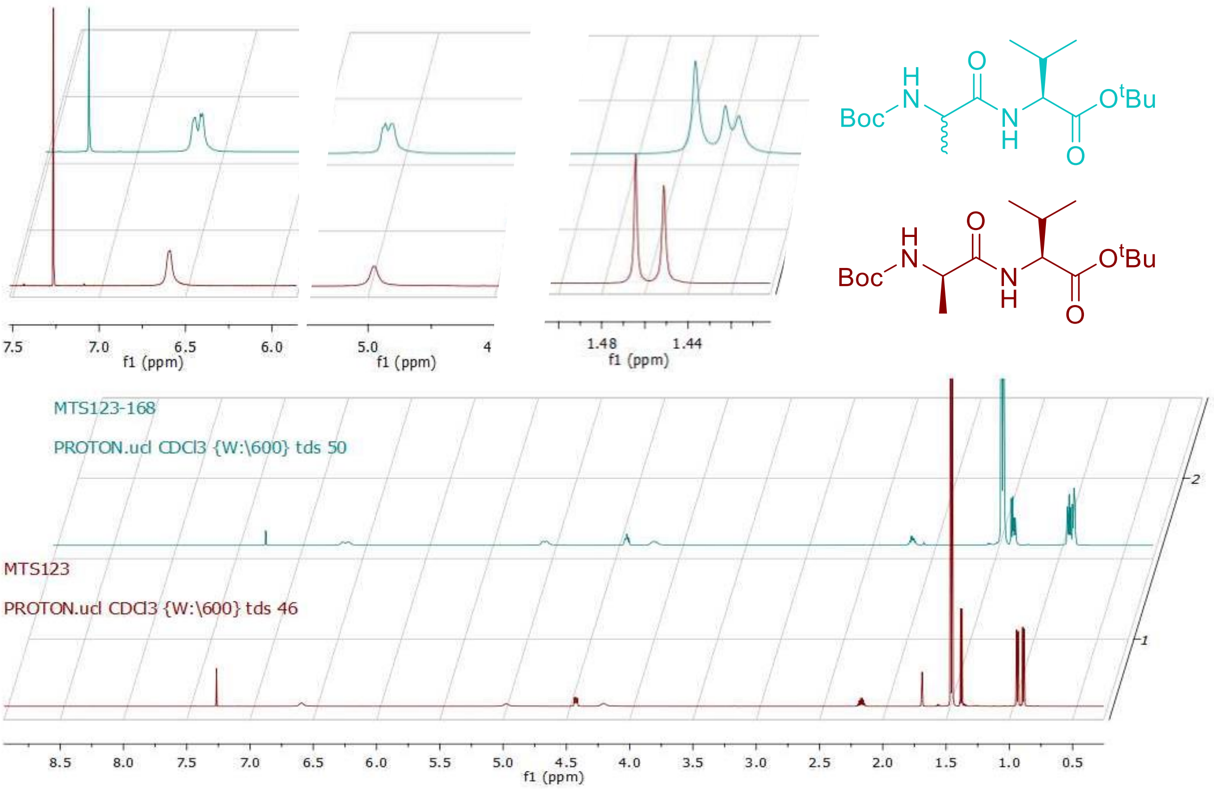

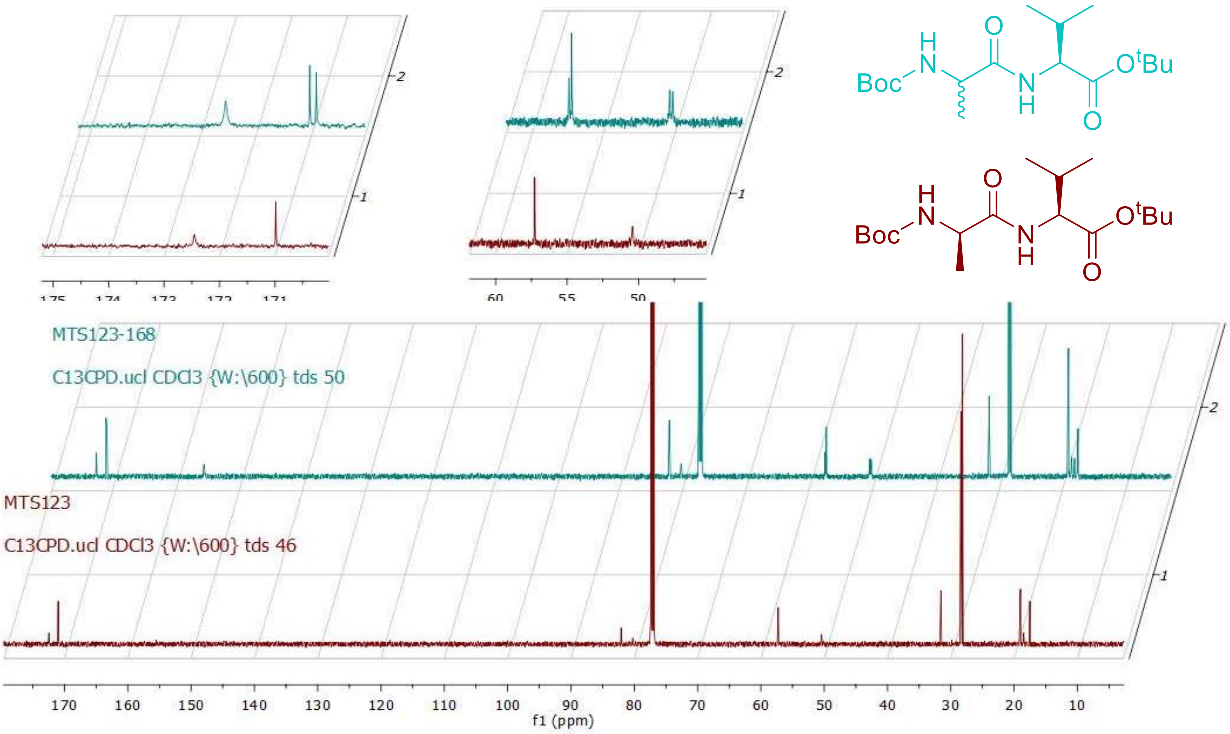

tert-butyl (tert-butoxycarbonyl)-D-alanyl-L-phenylalaninate (42)

Overlay of spectra of Boc-D-Ala-L-Phe-O^{t}Bu (42) and Boc-DL-Ala-L-Phe-O^{t}Bu

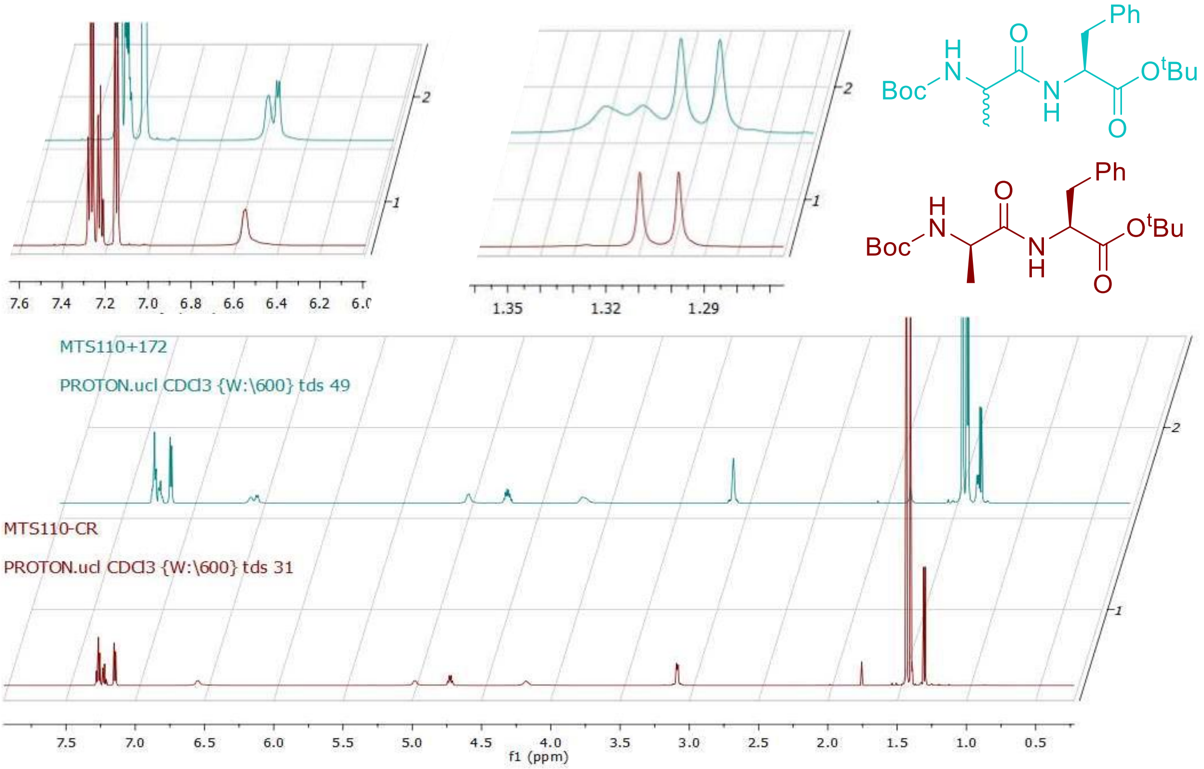

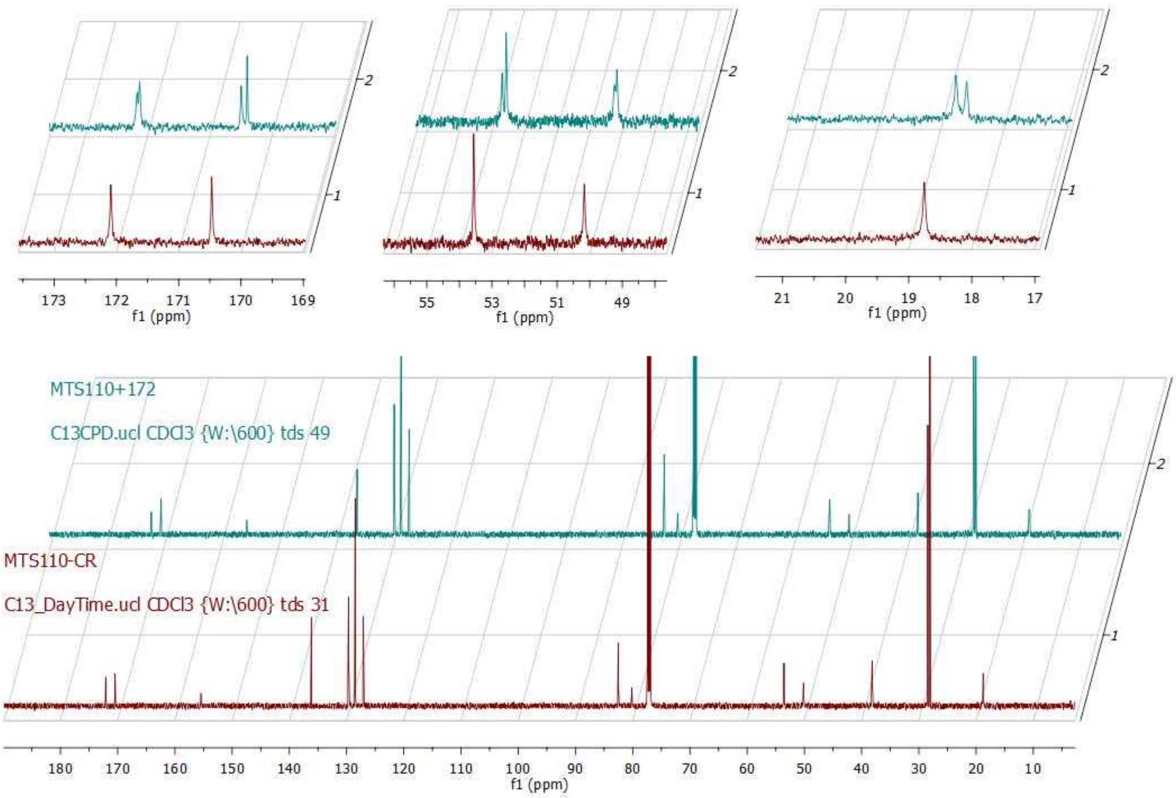

9.b NMR's of Marfey's derived amides (overlayed NMR's)

(*S*)-2-amino-N-benzyl-3-(1H-indol-3-yl)propanamide (46)

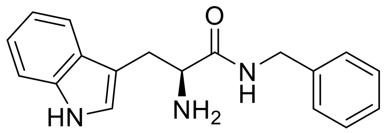

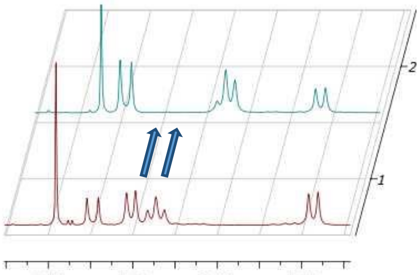

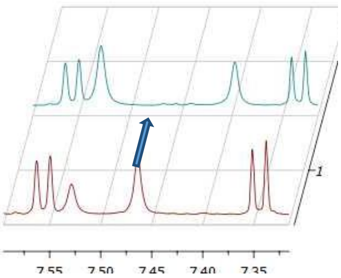

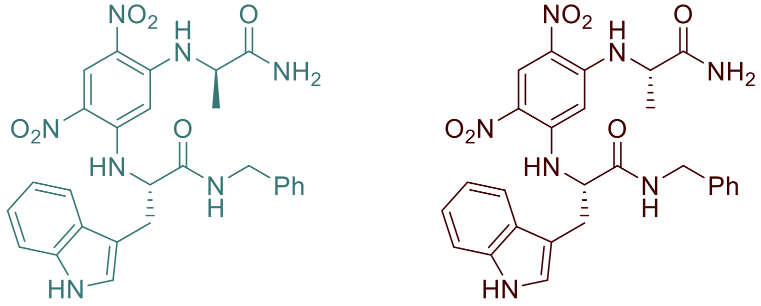

46 derivatised with L- and D-Marfey

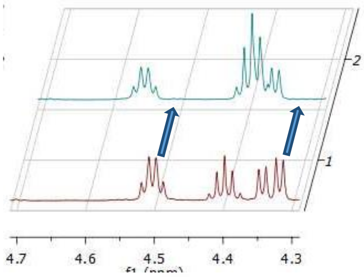

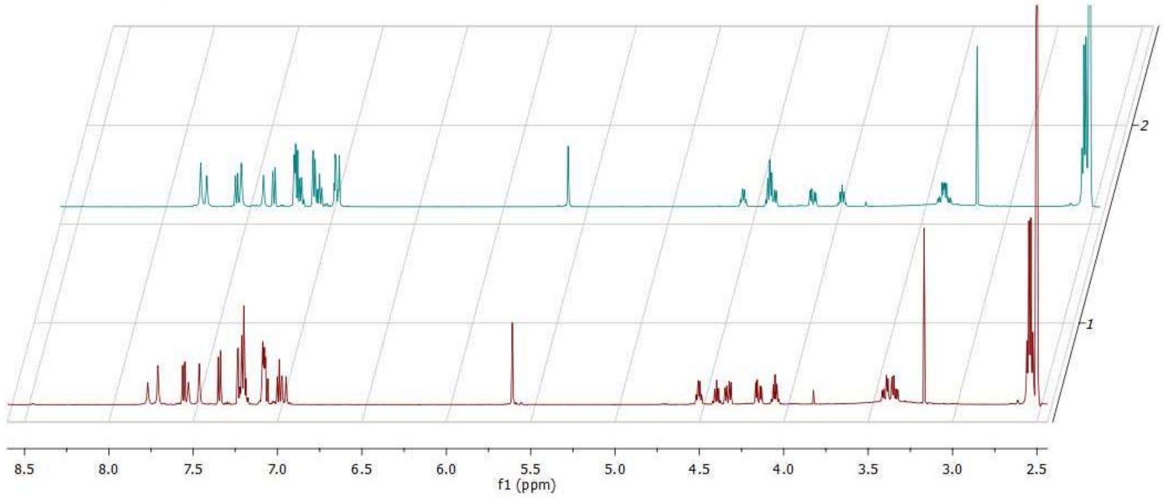

(S)-2-Amino-N-benzyl-3-methylbutanamide (47)

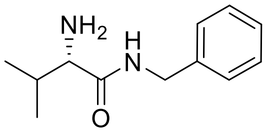

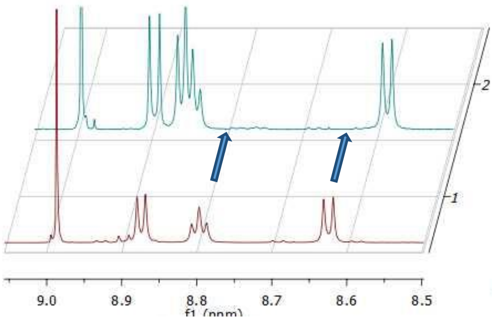

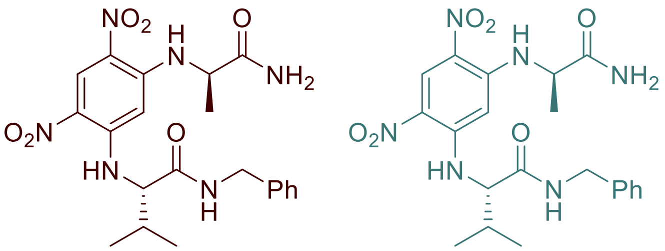

47 derivatised with L- and D-Marfey

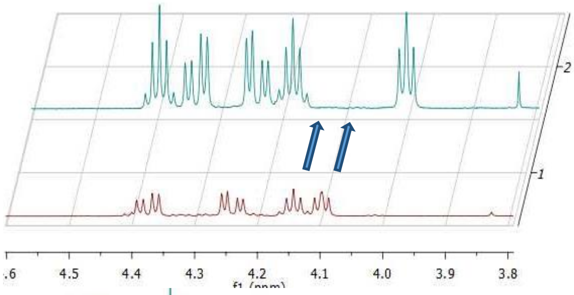

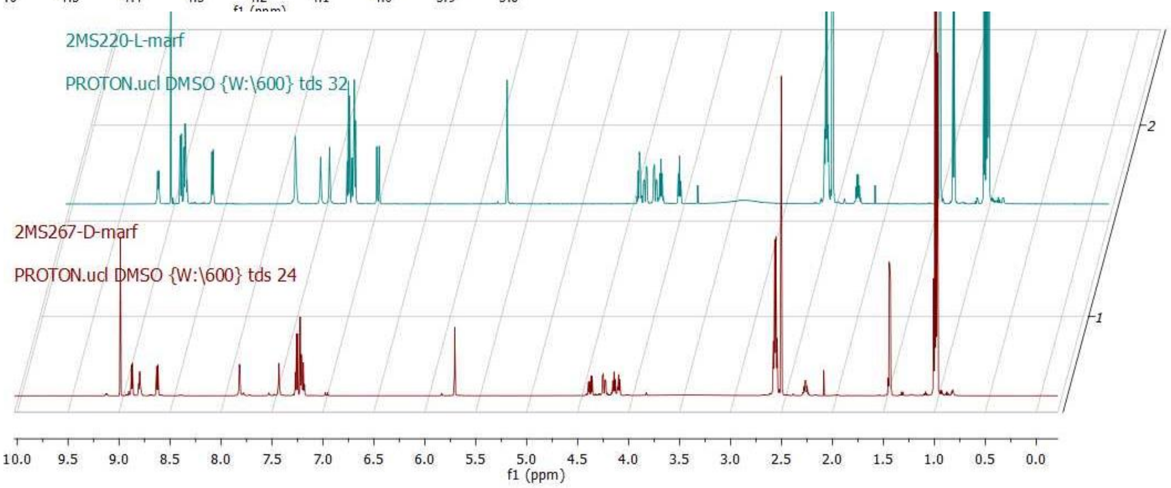

(S)-2-Amino-*N*-benzyl-3-methylbutanamide (47, using 30 mol% $B(OCH_2CF_3)_3$)

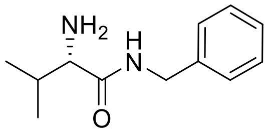

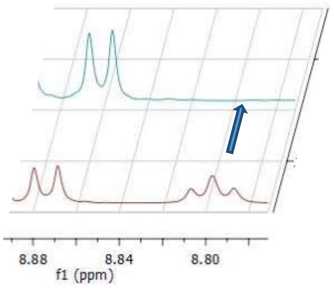

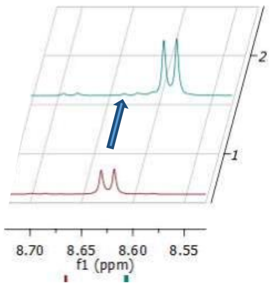

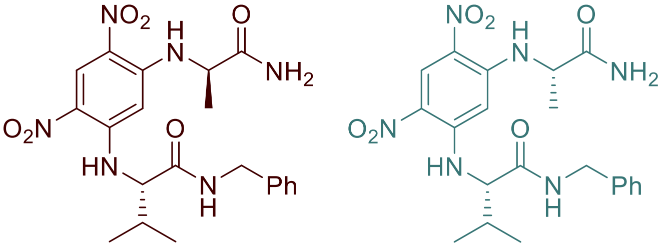

47 derivatised with L- and D-Marfey

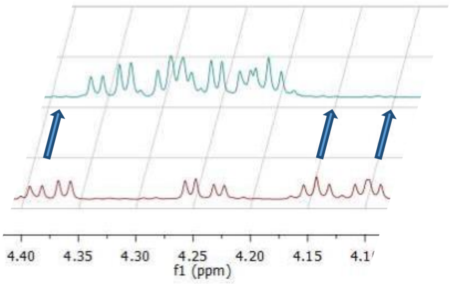

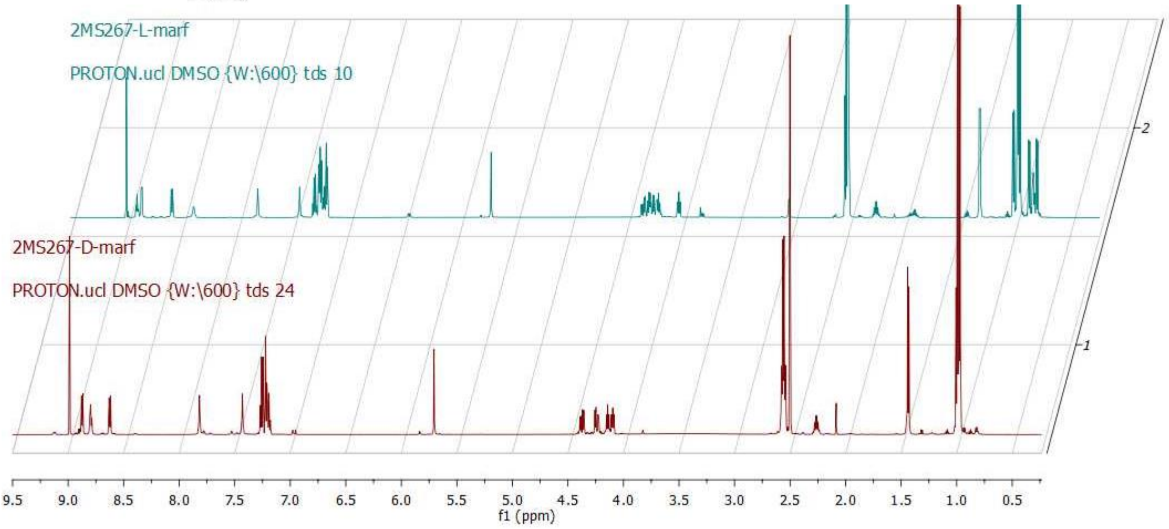

(S)-2-amino-N-benzyl-4-(methylthio)butanamide (49)

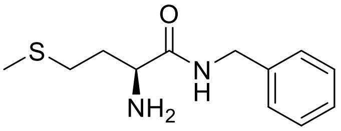

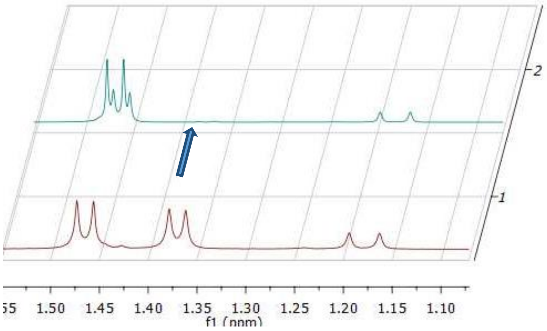

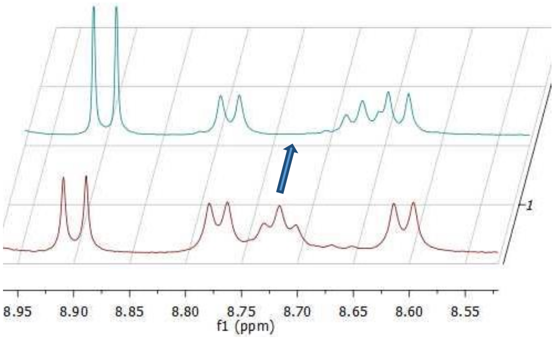

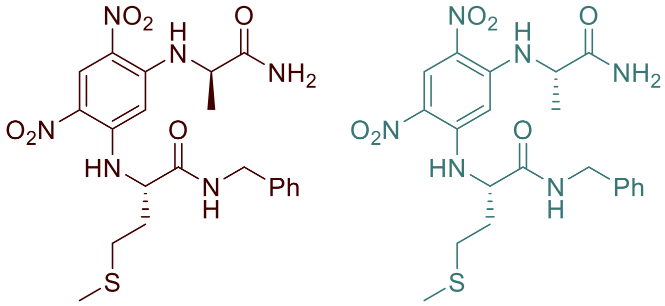

49 derivatised with L- and D-Marfey

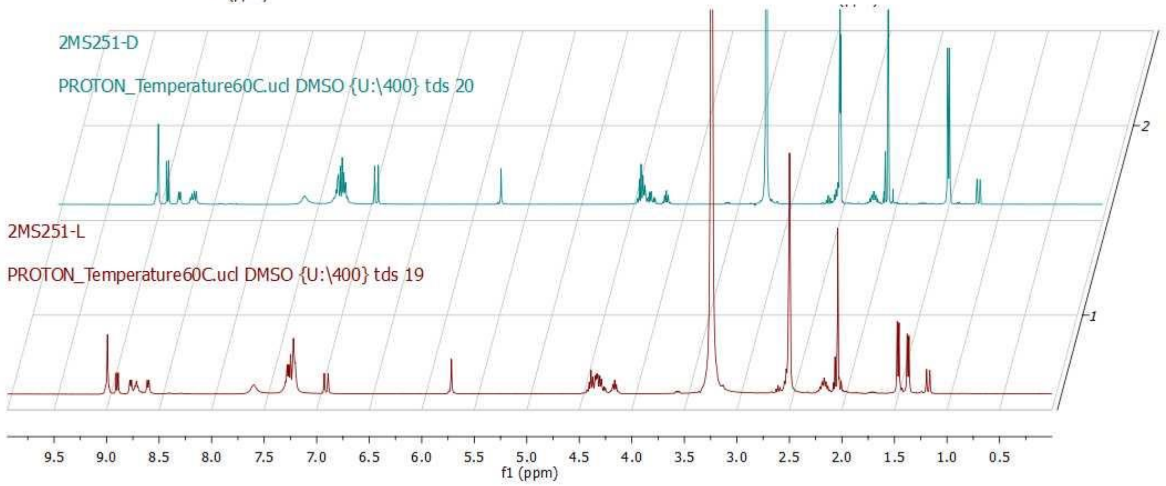

(S)-2-amino-N-benzyl-4-methylpentanamide (50)

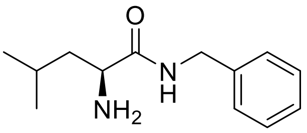

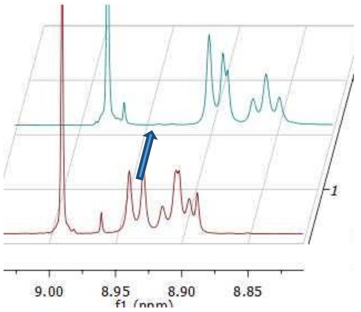

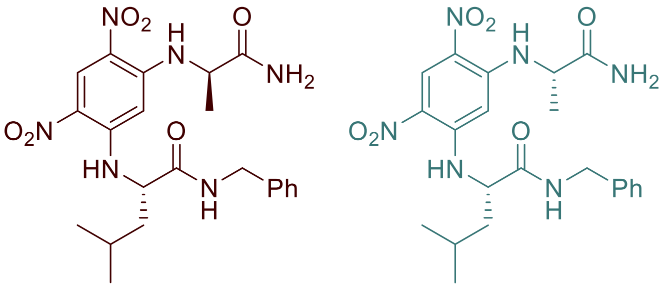

50 derivatised with L- and D-Marfey

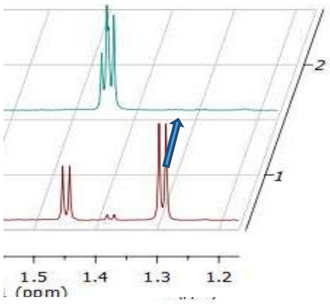

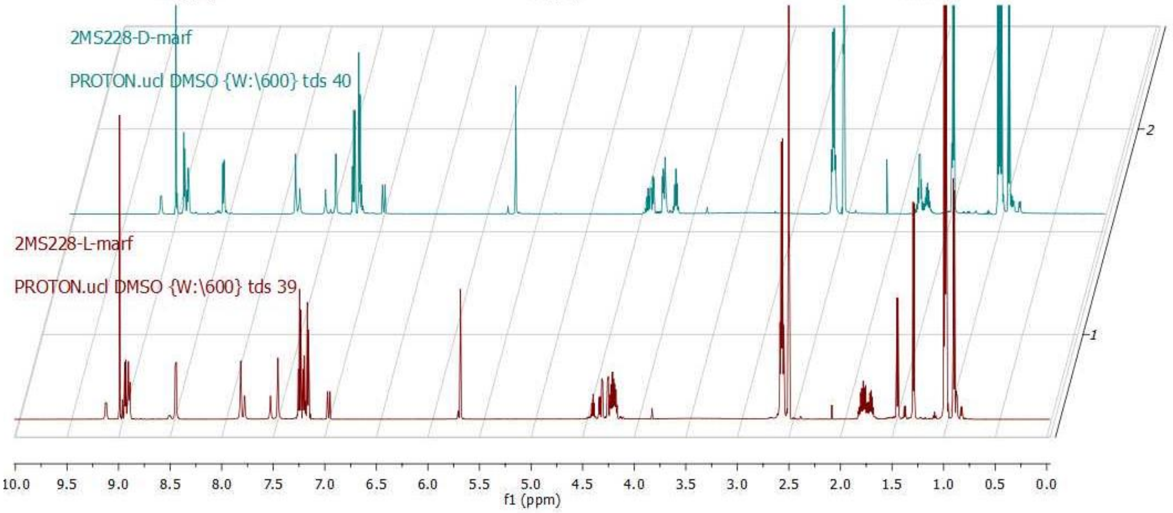

(*S*)-2-amino-N-benzyl-3-phenylpropanamide (51)

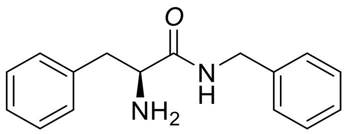

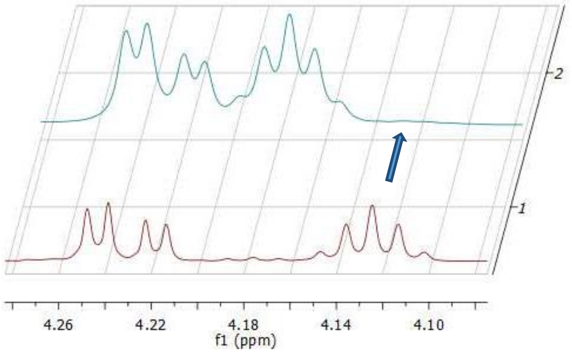

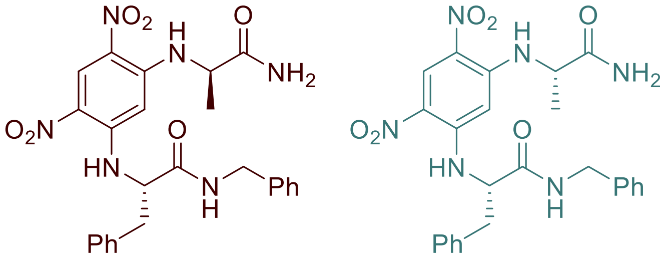

51 derivatised with L- and D-Marfey

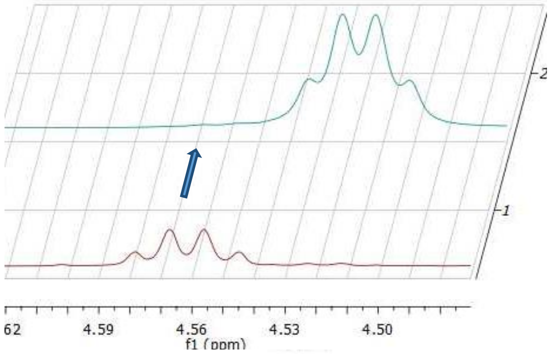

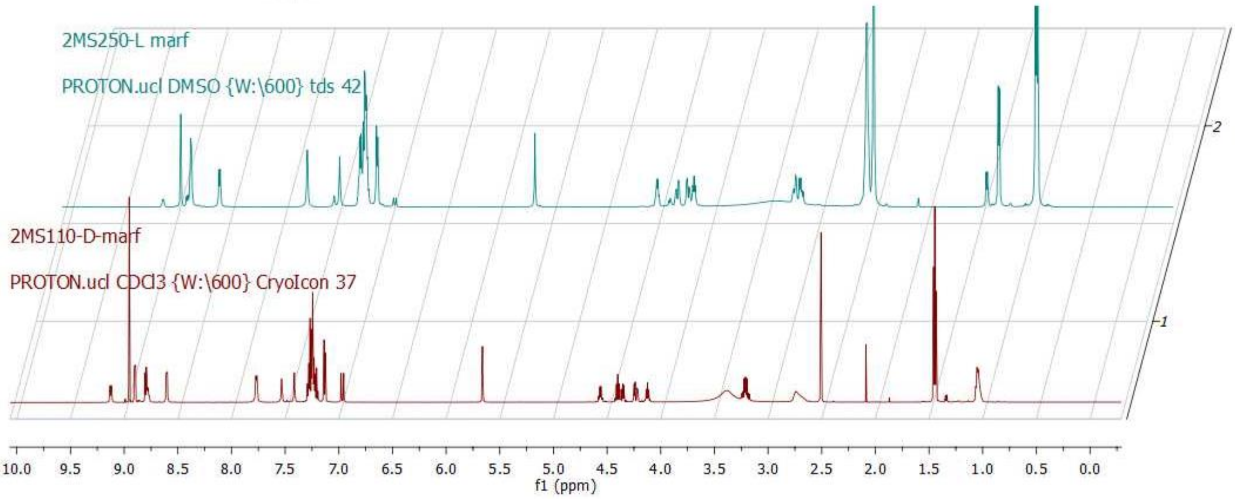

(S)-2-amino-N-benzylbutanamide (53)

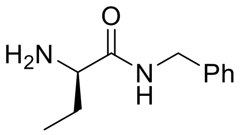

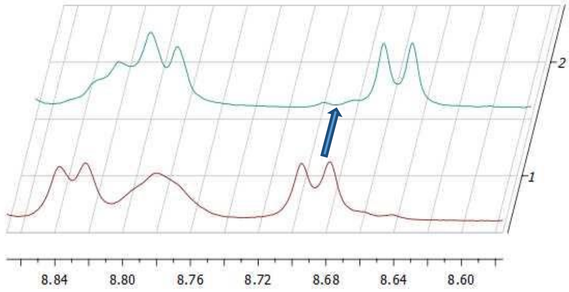

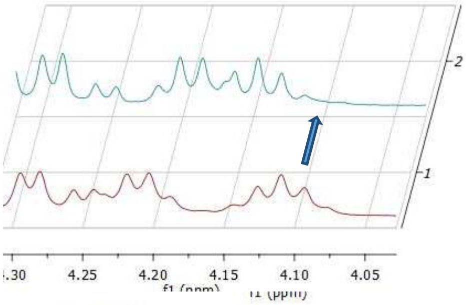

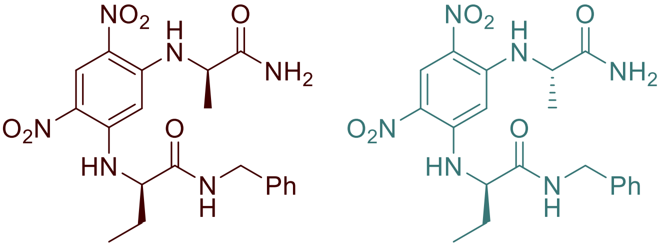

53 derivatised with L- and D-Marfey

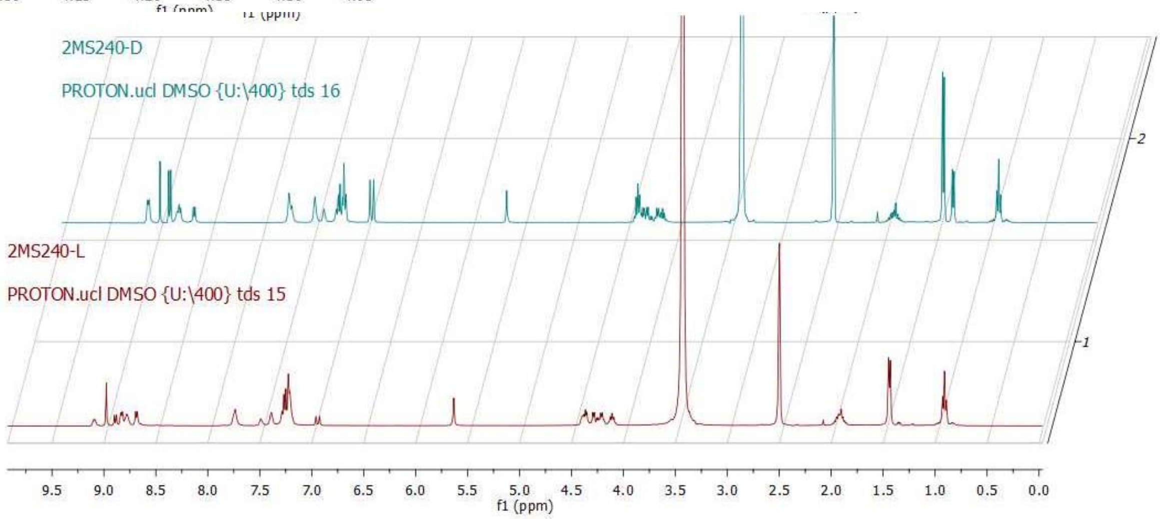

(*S*)-*N*-benzylpyrrolidine-2-carboxamide (54)

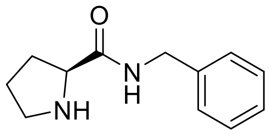

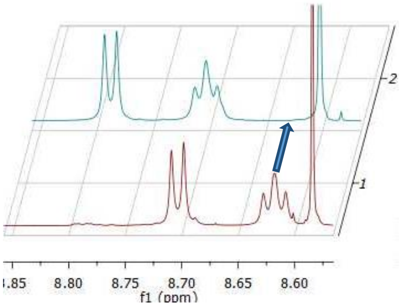

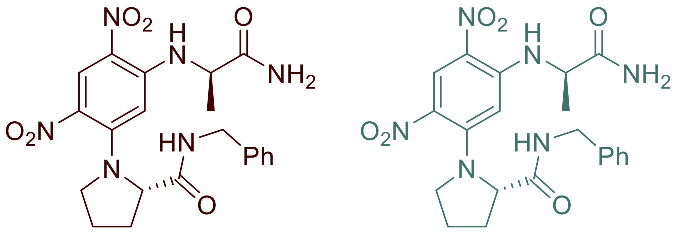

54 derivatised with L- and D-Marfey

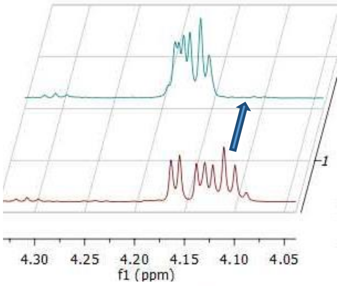

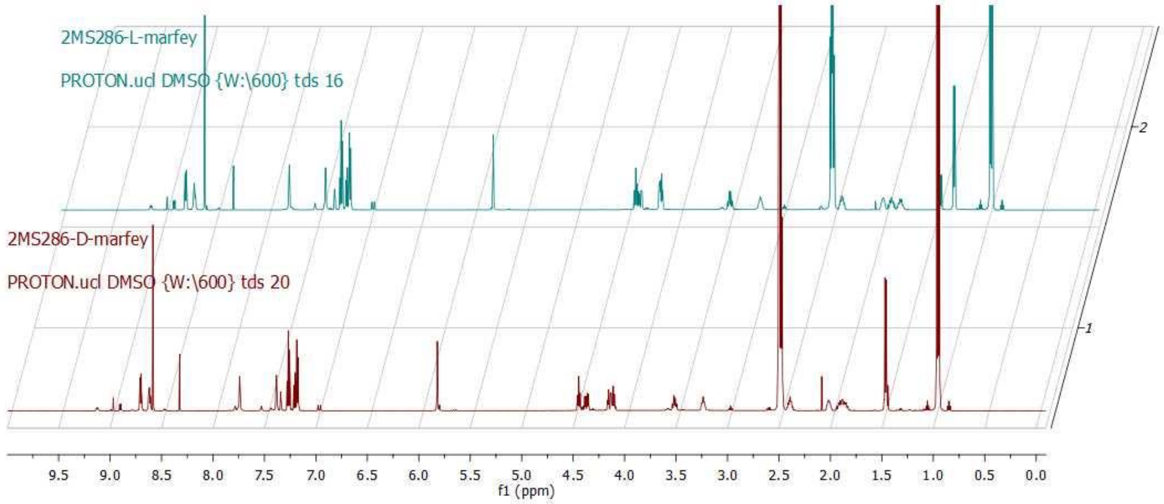

***(S)*-2-amino-N-benzylbutanamide (56)**

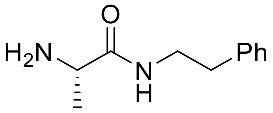

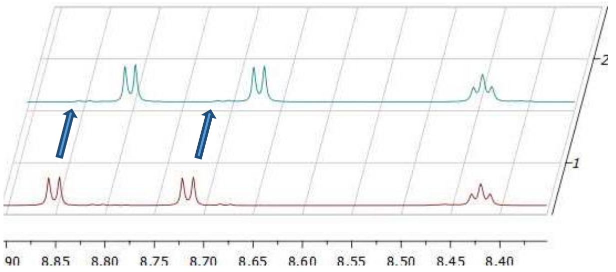

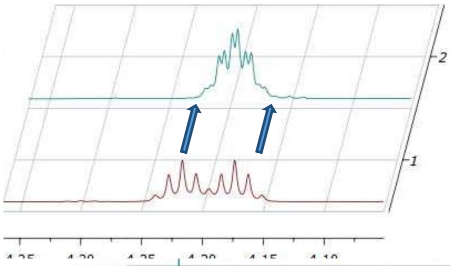

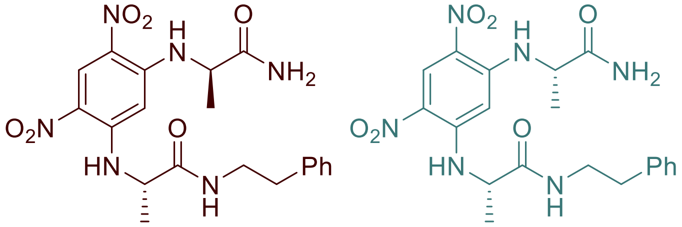

56 derivatised with L- and D-Marfey

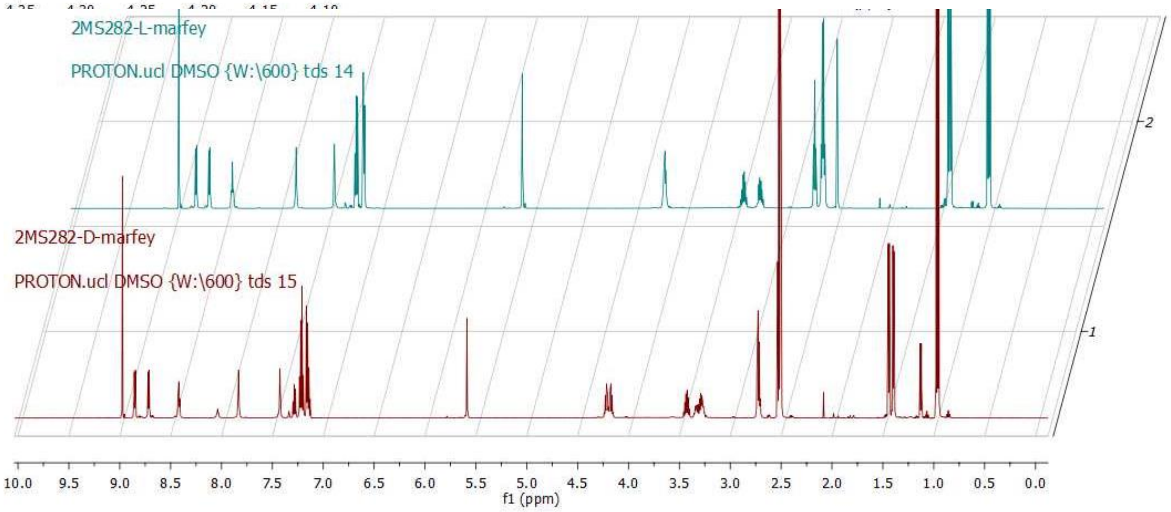

(S)-2-amino-N-hexyl-3-phenylpropanamide (58)

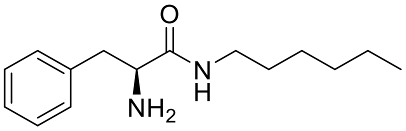

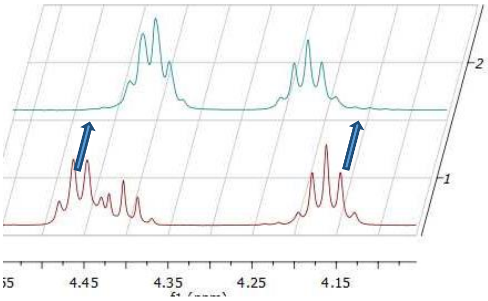

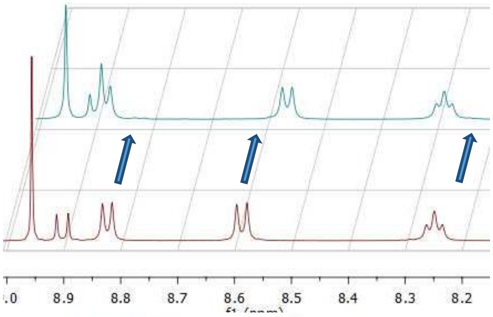

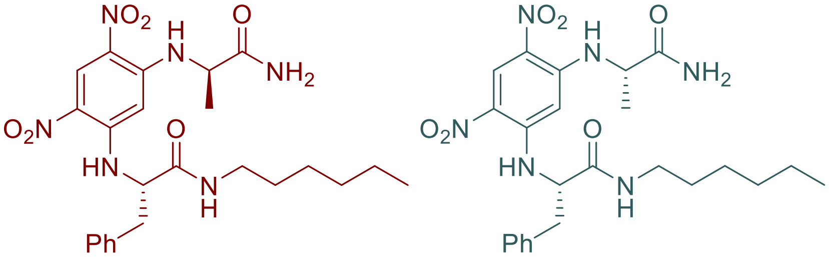

58 derivatised with L- and D-Marfey

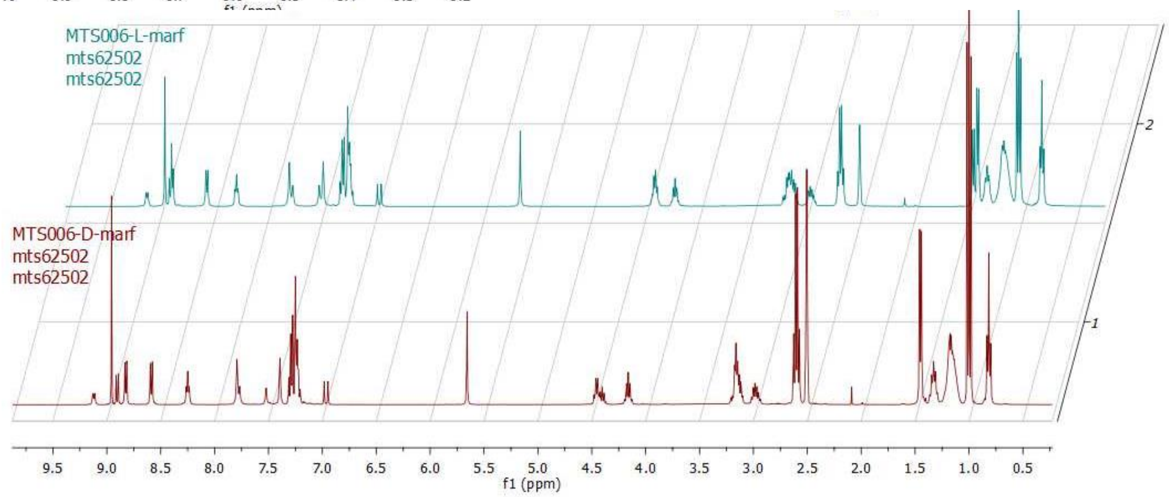

tert-Butyl (*S*)-prolylglycinate (59)

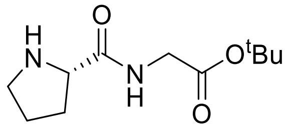

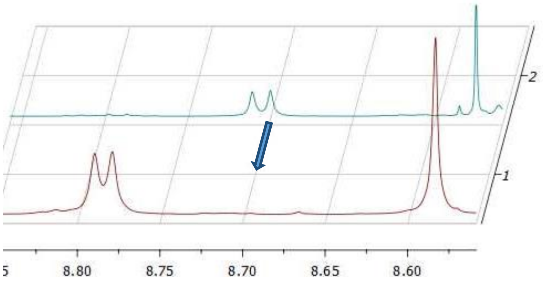

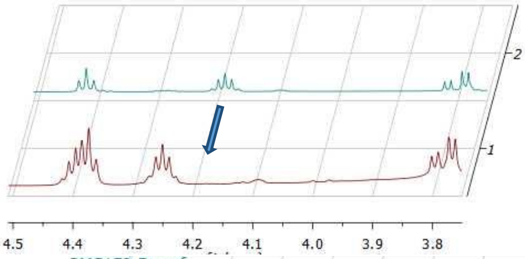

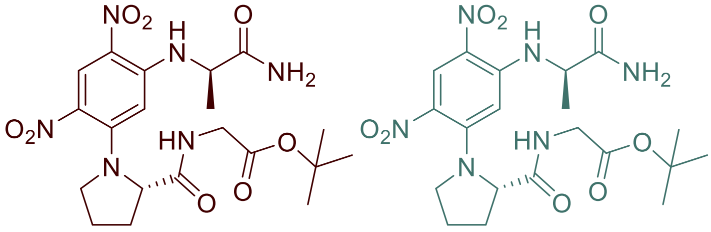

59 derivatised with L- and D-Marfey

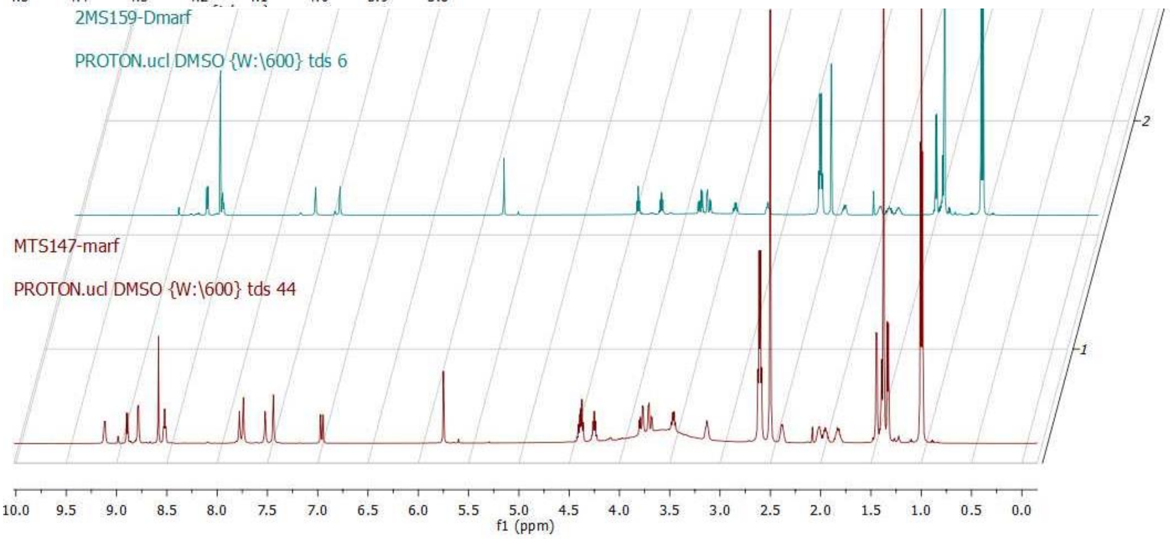

(*R*)-3-amino-1-(3-(trifluoromethyl)-5,6-dihydro-[1,2,4]triazolo[4,3-a]pyrazin-7(8H)-yl)-4-(2,4,5-trifluorophenyl)butan-1-one (Sitagliptin, 72)

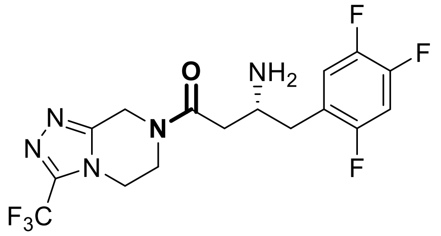

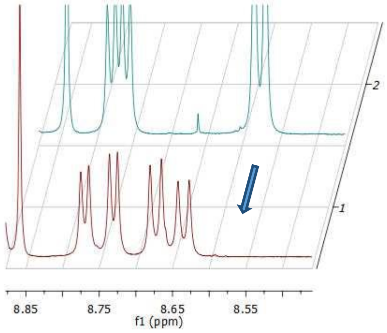

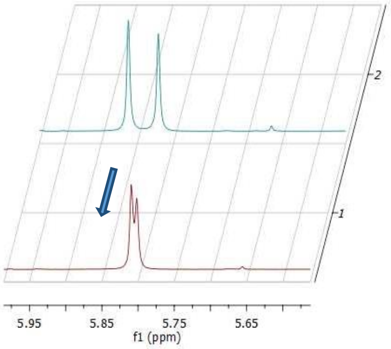

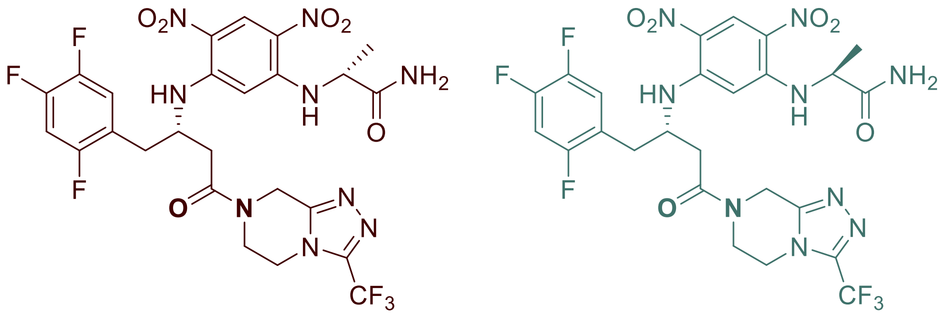

72 derivatised with L- and D-Marfey

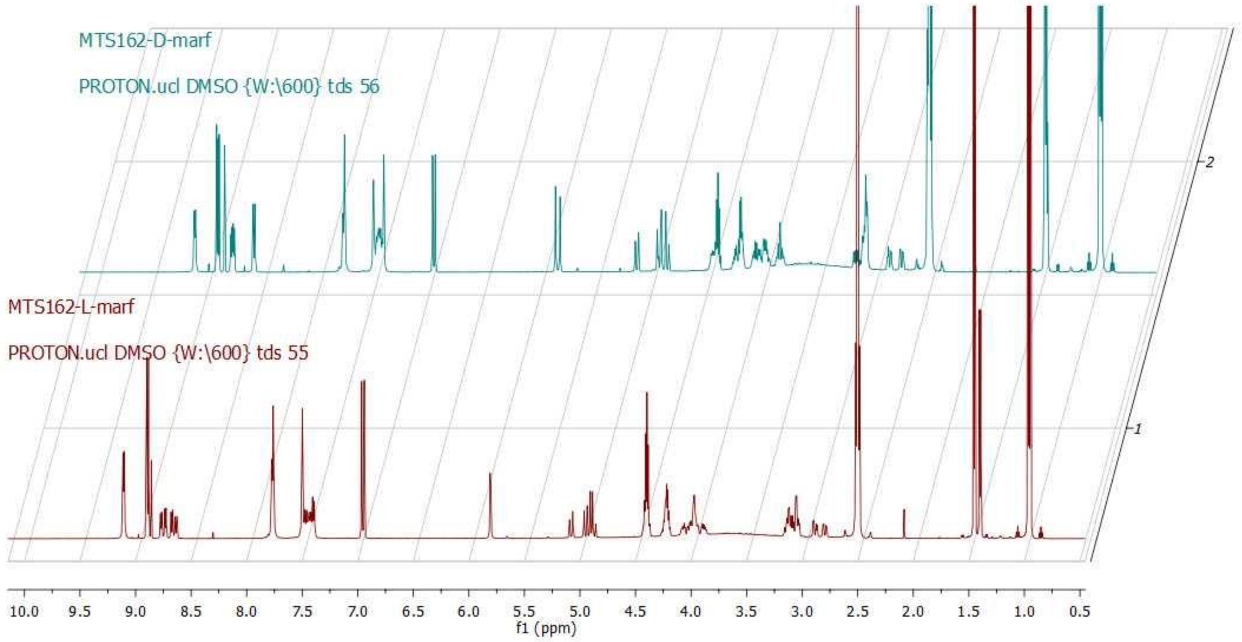

9.c Chiral shift reagent interaction for enantiopurity measurement

(*R*)-5-(4-Methylpiperazine-1-carbonyl)pyrrolidin-2-one (60)

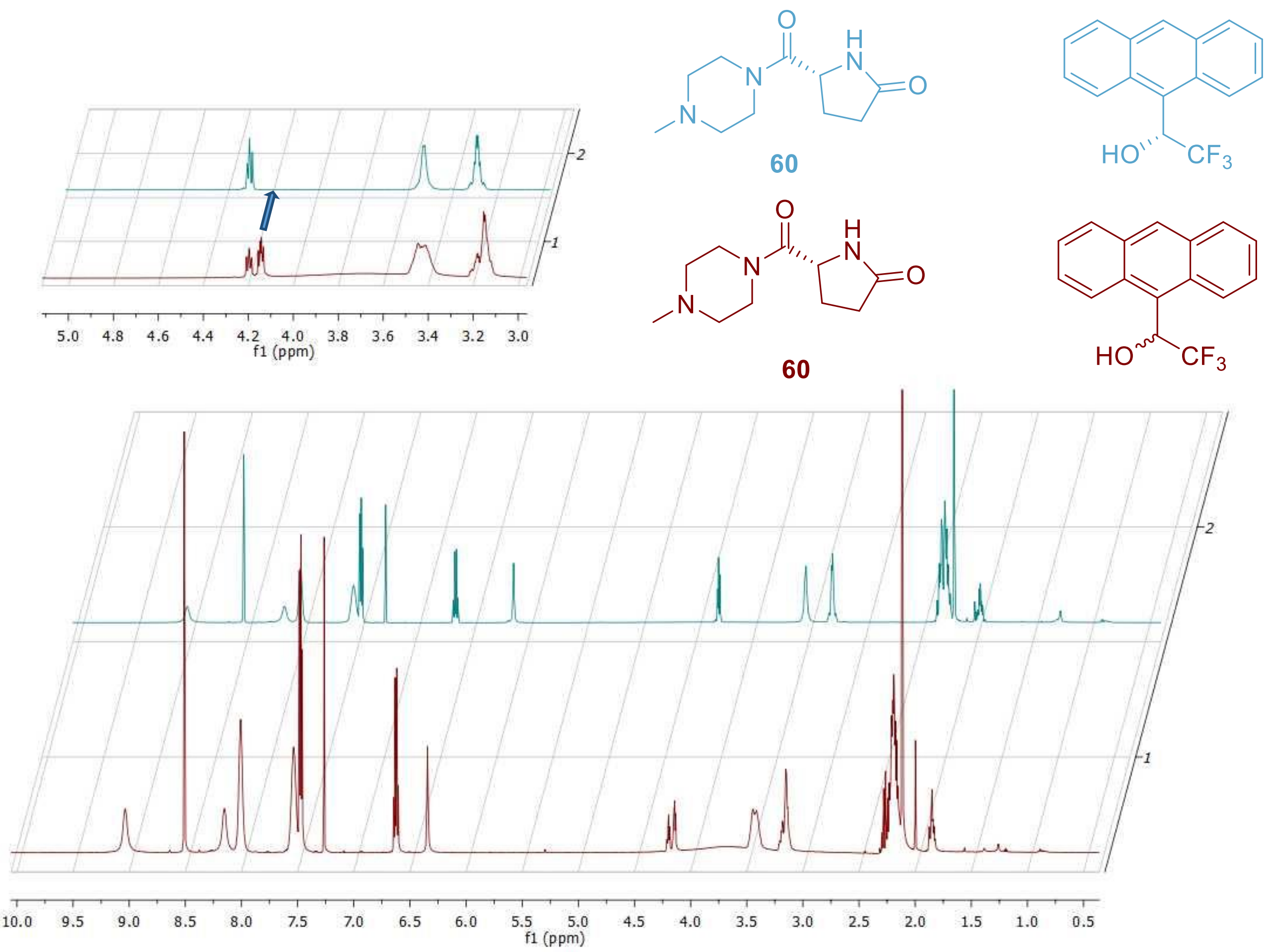

Supplement: http://advances.sciencemag.org/cgi/content/full/3/9/e1701028/DC1 [file 1701028_SM.pdf]
